# An evolutionarily conserved constellation of functional *cis*-elements programs the virus-responsive fate of the human (epi)genome

**DOI:** 10.1093/nar/gkaf207

**Published:** 2025-03-25

**Authors:** Marianna A Koutsi, Marialena Pouliou, Dimitris Chatzopoulos, Lydia Champezou, Konstantinos Zagkas, Marili Vasilogianni, Alexandra G Kouroukli, Marios Agelopoulos

**Affiliations:** Center of Basic Research, Biomedical Research Foundation, Academy of Athens, Athens 11527, Greece; Center of Basic Research, Biomedical Research Foundation, Academy of Athens, Athens 11527, Greece; Center of Basic Research, Biomedical Research Foundation, Academy of Athens, Athens 11527, Greece; Center of Basic Research, Biomedical Research Foundation, Academy of Athens, Athens 11527, Greece; Center of Basic Research, Biomedical Research Foundation, Academy of Athens, Athens 11527, Greece; Center of Basic Research, Biomedical Research Foundation, Academy of Athens, Athens 11527, Greece; Center of Basic Research, Biomedical Research Foundation, Academy of Athens, Athens 11527, Greece; Center of Basic Research, Biomedical Research Foundation, Academy of Athens, Athens 11527, Greece

## Abstract

Human health depends on perplexing defensive cellular responses against microbial pathogens like *Viruses*. Despite the major effort undertaken, the (epi)genomic mechanisms that human cells utilize to tailor defensive gene expression programs against microbial attacks have remained inadequately understood, mainly due to a significant lack of recording of the *in vivo* functional *cis*-regulatory modules (CRMs) of the human genome. Here, we introduce the virus-responsive fate of the human (epi)genome as characterized in naïve and infected cells by functional *genomics*, computational biology, DNA evolution, and DNA Grammar and Syntax investigations. We discovered that multitudes of novel functional virus-responsive CRMs (vrCRMs) compose typical enhancers (tEs), super-enhancers (SEs), repetitive-DNA enhancers (rDEs), and stand-alone functional genomic stretches that grant human cells regulatory underpinnings for layering basal immunity and eliminating illogical/harmful defensive responses under homeostasis, yet stimulating virus-responsive genes and transposable elements (TEs) upon infection. Moreover, extensive epigenomic reprogramming of previously unknown SE landscapes marks the transition from naïve to antiviral human cell states and involves the functions of the antimicrobial transcription factors (TFs), including interferon response factor 3 (IRF3) and nuclear factor-κB (NF-κB), as well as coactivators and transcriptional apparatus, along with intensive modifications/alterations in histone marks and chromatin accessibility. Considering the polyphyletic evolutionary fingerprints of the composite DNA sequences of the vrCRMs assessed by TFs-STARR-seq, ranging from the animal to microbial kingdoms, the conserved features of antimicrobial TFs and chromatin complexes, and their pluripotent stimulus-induced activation, these findings shed light on how mammalian (epi)genomes evolved their functions to interpret the exogenous stress inflicted and program defensive transcriptional responses against microbial agents. Crucially, many known human short variants, e.g. single-nucleotide polymorphisms (SNPs), insertions, deletions etc., and quantitative trait loci (QTLs) linked to autoimmune diseases, such as multiple sclerosis (MS), systemic lupus erythematosus (SLE), Crohn’s disease (CD) etc., were mapped within or vastly proximal (±2.5 kb) to the novel *in vivo* functional SEs and vrCRMs discovered, thus underscoring the impact of their (mal)functions on human physiology and disease development. Hence, we delved into the virus-responsive fate of the human (epi)genome and illuminated its architecture, function, evolutionary origins, and its significance for cellular homeostasis. These results allow us to chart the “Human hyper-Atlas of virus-infection”, an integrated “molecular *in silico*” encyclopedia situated in the UCSC Genome Browser that benefits our mechanistic understanding of human infectious/(auto)immune diseases development and can facilitate the generation of *in vivo* preclinical animal models, drug design, and evolution of therapeutic applications.

## Graphical Abstract

**Graphical Abstract:**
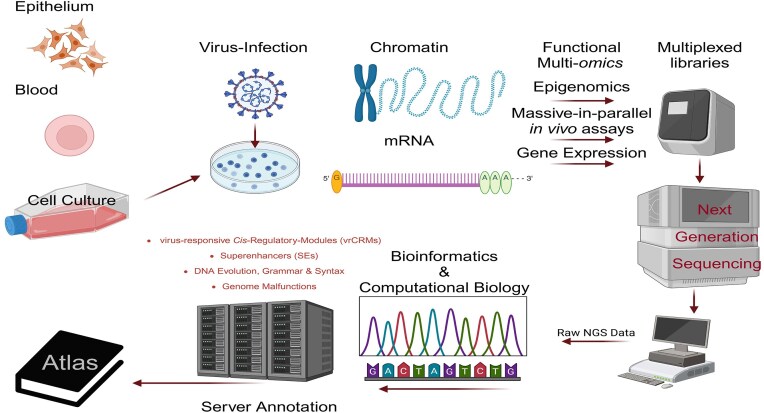

## Introduction

An integral part of human homeostasis substantially relies on the capability of cells to mount defensive responses tailored to fight microbial pathogens, an essential function that depends on their capacity to encode stimulus-induced gene expression programs. Infection of human cells by *Viruses* stimulates the concerted operation of pattern recognition receptors (PRRs), signal transduction pathways, and transcriptional regulators that mechanistically converge on *cis*-acting genomic elements that fine-tune the “ON” and “OFF” transcriptional states [1] of virus-responsive differentially expressed genes (vrDEGs). Advanced studies on mammalian cells exposed to *Viruses*, nucleic acid analogs of viral genomes [e.g. (poly (I:C)], or other immunogenic stimuli [2–7] have provided insights on defensive/immune gene expression regulation, typically with an emphasis on transcriptional regulators, signaling cascades, target-genes, or chromatin loci of interest. However, a comprehensive assessment of the *in vivo* functional *cis*-regulatory modules (CRMs) of the human (epi)genome is lacking [8–10], and a definitive anatomy of its virus-responsive fate remains elusive. In particular, the full spectrum of human functional typical enhancers (tEs), Super-enhancers (SEs), repetitive-DNA enhancers (rDEs) etc., has not been entirely elucidated in cell-type-specific, temporal, quantitative, and qualitative contexts, while its modularity, evolutionary conservation, and relevance for (auto)immune diseases are still unresolved. For instance, SEs contribute to cellular identity and responses and their malfunctions escort human disease manifestation [11–13]. However, their role in the establishment of mammalian virus-stimulated gene expression programs is remarkably overlooked.

A major challenge in understanding human defensive transcriptional responses is our inadequate knowledge of *cis*-acting elements per se. These are dispersed across ∼98% of the human genome and encompass a wide lexicon of transcription factor binding motifs (TFBMs) recognized with divergent legibility by a plethora of transcription factors (>1500 TFs) in distinct tissues [14–16]. Furthermore, *cis*-acting elements, when chromatinized, exhibit nearly unpredictable, mechanistic, and functional underpinnings/prerequisites. Thus, despite the revolution of *multi*-*omics* applications both at the population- and single-cell levels, the topographic fine mapping of the human functional virus-responsive CRMs (vrCRMs) at the chromosomal scale is missing. Hence, the (epi)genomic mechanisms that human cells utilize to tailor efficacious transcriptional responses against microbial pathogens, including *Viruses*, have remained unresolved, thus hindering research on human (auto)immune diseases. Apparently, well-known genes with unknown or non-characterized *cis*-acting elements are often inadequately attributed to genomic loci that host short variants, e.g. single-nucleotide polymorphisms (SNPs), and/or quantitative trait loci (QTLs) connected to pathogenic phenotypes, e.g. (auto)immune, and vice versa. To this end, our holistic functional *multi*-omics strategy was applied to fill these critical gaps of knowledge by massively characterizing human vrCRMs and illuminating how they are (i) architecturally stated, (ii) epigenetically profiled, (iii) mechanistically ruled, (iv) evolutionary inherited, (v) situated within their chromatin microenvironment of residence across human chromosomes, and (vi) associated in *cis* with DNA sequence variations linked to (auto)immune diseases.

Herein, we sought to obtain a comprehensive blueprint of the regulatory principles of virus-stimulated (epi)genomic/transcriptional response. We addressed the mechanisms that regulate the divergence of transcriptional activation rates of human vrDEGs, the principles of the human (epi)genome (re)programming, on the means of its architectural and functional compartmentalization, the commission of previously unidentified vrCRMs, the evolutionary origins of their composite DNA sequences, and significant *in cis* associations with autoimmune linked SNPs and QTLs. We exhaustively assayed the human transcriptome and (epi)genome in epithelial cells and B-lymphocytes prior to and upon virus-infection, through an integrative workflow of functional genomics (transcriptomics, epigenomics, and massive-in-parallel*in vivo* functional characterization by TFs-ChIP-STARR-seq), computational biology (bioinformatics, algorithms, and statistical analyses), DNA evolution, DNA Grammar and Syntax investigations (genome scanning, phylogenomics, TFs *in silico* footprinting), and analyses of (auto)immune diseases-associated genomic variations/malfunctions. Additional human and mouse cell systems of diverse origins, e.g. lung cells, fibroblasts, organoids, etc., were also investigated. Our results identify that the human (epi)genome grants cells with regulatory underpinnings critical to layer basal immunity and eradicate illogical-harmful defensive responses under homeostasis, yet to stimulate accurate antiviral transcriptional responses, upon infection. We also show that extensive transcriptome and epigenome reprogramming hallmarks the transition from naïve to antiviral human cell states. These phenomena depend on expanded chromosomal entities that harbor thousands of previously unidentified, unsupervised, or overlooked virus-responsive tEs, SEs, and rDEs that are associated *in cis* with vrDEGs and exhibit remarkable stimulus-specificity, characteristic epigenomic states, and functional fitness, *in vivo*. Using massive-in-parallel TFs-ChIP-STARR-seq coupled with *epigenomics* profiling, we succeeded in authenticating >3300 functional vrCRMs that reside within tEs, SEs, rDEs, and stand-alone functional genomic stretches (hereinafter referred to as “safGs”) of the human genome and are bound by interferon response factor 3 (IRF3), or/and nuclear factor-κB (NF-κB). Phylogenetic analyses of the discovered vrCRMs in nonrepetitive and repetitive DNA constituents [e.g. simple tandem repeats (STRs), dispersed repeats (DRs), or chimeras of those [c(T:D)Rs]] and DNA Grammar and Syntax assessments have allowed us to address substantial evolutionary hierarchies and to distinguish hundreds of functional IRF3- and/or NF-κB-bound homotypic clusters of TFs binding sites (HCTFBSs). Genome scanning across ∼100 species pervasively traced IRF3-HCTFBSs “clustered” fingerprints within the genomes of human relatives, other complex and unicellular eukaryotes, and additional organisms of the microbial kingdoms, including human infectious pathogens, e.g. parasites and *Viruses*. Crucially, we identified that mechanisms of horizontal transmission of viral DNA elements might have committed to the evolutionary (re)shaping of such microbial-responsive (epi)genomic fates across mammals. Moreover, ChIP-seq and RNA-seq experiments in human and mouse cells and mapping and expression profiling of virus-responsive differentially transcribed transposable elements (vrDTTEs) suggest the functional conservation of the human vrCRMs and a role of TEs in defensive cellular processes within the animal kingdom. Lastly, these functional vrCRMs often neighbor or co-localize with short variants, e.g. SNPs, and QTLs linked to complex traits/phenotypes, including (auto)immune diseases, thus highlighting novel biologically meaningful associations for human pathophysiology. These findings (i) profile the functional conductance of the virus-responsive fate of the human (epi)genome in the mechanisms of cellular homeostasis and disease development, (ii) illuminate previously unknown structural and evolutionary insights of the human genome, and (iii) shed light on the perplexing natural conflicts and “symbiotic” interactions between hosts and pathogens that are amenably linked to defensive gene expression. They also allow us to chart the “Human hyper-Atlas of virus-infection”, an integrated “molecular *in silico*” encyclopedia situated in the UCSC Genome Browser.

## Materials and methods

A list of consumables, including antibodies, culture media etc., used in the study is provided in Supplementary Table S6.

### Cell culture

Human and mouse cells were cultured according to the manufacturer’s instructions under optimum conditions. Cell cultures were established in laboratory incubators (37°C, 5% CO_2_). HeLa cells (ATCC, CCL-2^™^), MRC-5 cells (ATCC, CCL-171™), A549 cells (ATCC, CCL-185^™^), and NIH/3T3 cells (ATCC, CRL-1658^™^) were grown in Dulbecco's Modified Eagle's Medium (DMEM) medium (Sigma–Aldrich). For MRC-5 cells, the medium was replaced at a ratio of 6/10 (v/v) every 2 days. Namalwa cells (ATCC, CRL-1432^™^) and Tohoku Hospital Pediatrics-1 cells (THP-1) (ATCC, TIB-202^™^) were grown in RPMI 1640 medium (Sigma–Aldrich). For THP-1, the culture medium was supplemented with 1% Pen-Strep (Gibco), 20% (v/v) heat-inactivated fetal bovine serum (FBS) (Gibco, Biowest), and 2-mercaptoethanol to a final concentration of 0.05 mM. For the rest of the cultures, the media were supplemented with 1% Pen-Strep (Gibco) and 10% (v/v) heat-inactivated Fetal Bovine Serum (FBS) (Gibco, Biowest).

### Virus infection

Cell cultures were grown at 85%–95% confluence and subjected to mock-infection (0 h) or Sendai virus infection (SVI) (Cantell strain, Charles River lab., 2000 HA units/ml) for 3, 6, or 7 h, prior to proceeding to downstream examinations. Sendai virus was supplied directly in the culture medium at a ratio of 1/10 (v/v). We supply the cultures of HeLa, Namalwa, MRC-5, NIH/3T3, A549 and THP-1 with ∼180–200 Hemagglutination Units (HAU)/ml of Sendai virus. This is equal to multiplicity of infection (MOI) 2 for HeLa, MOI 2 for Namalwa, MOI 4 for MRC-5, MOI 2 for NIH/3T3, MOI 2 for A549, and MOI 2 for THP-1.

### *Omics* assays

#### RNA-seq experiments, multiplexed-library preparation, and NGS-run

The culture medium was removed and cells were collected and washed twice with ice-cold 1× Phosphate buffered saline (PBS). Total RNA was extracted from cells using the TRI reagent (T9424, Sigma–Aldrich) and treated with DNase I (04716728001, Roche) for 30 min at 37°C to remove any DNA remnants, followed by purification with the RNeasy Mini Kit (74104, QIAGEN). Alternatively, the Monarch Total RNA Miniprep Kit (T2010S, NEB) was used, which includes treatment with DNase I for 15 min at 37°C. RNA quality was evaluated with the 2100 Bioanalyzer system with DNA 7500 kit (Agilent Technologies). RNA sequencing libraries were generated using the TruSeq RNA Library Prep Kit v2, Set A and B (RS-122-2001 and RS-122-2002) for Illumina following the manufacturer’s instructions [17] or the NEBNext Ultra II Directional RNA Library Prep Kit (E7760L) for Illumina. Samples were subjected to beads-purification using either AMPure XP beads (A63881, Beckman Coulter) or NEBNext Sample Purification Beads (E7767L, NEB), and the quality of the multiplexed libraries was evaluated at the 2100 Bioanalyzer system with DNA 7500 kit (Agilent Technologies). Next-generation sequencing (NGS) was conducted on an Illumina NextSeq 500 platform at the Greek Genome Center at BRFAA, generating 75 bp single-end reads. The RNA-seq experiments were carried out in biological replicates.

#### DNaseI-seq experiments, multiplexed-library preparation, and NGS-run

DNaseI-seq assays were conducted based on a published protocol [18] with modifications. Intact nuclei were isolated from 15 to 20 × 10^6^ cells, by sequential incubation in extraction buffers. In detail, the culture medium was removed and cells were collected and washed twice with ice-cold 1× PBS. Cell pellets were diluted in 200 μl 1× Buffer A [15 mM Tris pH 8.0, 15 mM NaCl, 60 mM KCl, 1 mM EDTA, and 0.5 mM spermidine] by gentle pipetting using cut tips, followed by the addition of an equal volume of 1× Buffer A enriched in NP-40, and incubation for 4 min on ice. For HeLa (HL), 1% final (v/v) NP-40 and for Namalwa (NM) 0.05% final (v/v) NP-40 were utilized. Cells were centrifuged at 2000 rpm for 1 min, at 4°C followed by aspiration and dilution in 100 μl of 1× DNaseI incubation buffer (04716728001, Roche) by gentle finger flick. Next, an aliquot of 3–5 μl was processed to Trypan-blue staining, a critical control step, to monitor the efficiency of the nuclei isolation under a light microscope. Nuclei were digested by the addition of 80 units of DNase I (04716728001, Roche) for 3 min at room temperature, followed by quenching of the reaction by the addition of an equal volume of 2× Stop-Buffer (50 mM Tris-Cl, pH 8.0, 100 mM NaCl, 0.1% Sodium Dodecyl Sulfate (SDS), 100 mM EDTA), gentle finger flick, incubation for 10 min at room temperature, addition of 20 μg of RNaseI, and 30-min incubation at room temperature. Next, the digested chromatin filaments were subjected to Proteinase K treatment (100 μg enzyme, 0.50% SDS, and 400 μl total volume of the digestion reaction) (Proteinase K, recombinant, PCR Grade, 3115836001, Sigma–Aldrich) overnight at 55°C, followed by phenol–chloroform extraction and ethanol-based precipitation of the isolated DNA, agarose gel (2%) electrophoresis, and extraction of the digested DNA fragments, ranging from 100–300 bp, by following standard procedures. Multiplexed DNA libraries were prepared according to a published protocol [19] and sequenced on an Illumina NextSeq 500, NovaSeq 6000, at the Greek Genome Center at BRFAA, and the HiSeq 2000 at EMBL Core Genomics Facility, generating 75 bp or 100 bp single-end reads.

#### ChIP-seq experiments, multiplexed-library preparation, and NGS-run

ChIP-seq assays on human and mouse cells were carried out based on published protocols [3, 20, 21] with modifications. For HeLa, Namalwa, A549, THP-1, and NIH/3T3, cells were fixed in 1% formaldehyde (F8775, Sigma–Aldrich), for 30 min at room temperature, followed by quenching of the reaction by 0.125 M glycine. Chromatin was isolated by sequential incubation in extraction buffers, followed by sonication. In detail, 45–60 × 10^6^ cells were collected and washed twice with ice-cold 1× PBS. Cell pellets were diluted in 15 ml Buffer I (10 mM Tris-Cl, pH 8.0, 10 mM EDTA, and 0.25% Triton X-100) and incubated for 10 min on ice, followed by centrifugation at 3500 rpm for 5 min at 4°C and aspiration. Pellets were diluted in 15 ml Buffer II (10 mM Tris-Cl, pH 8.0, 200 mM NaCl, and 1 mM EDTA) and incubated for 10 min on ice, followed by centrifugation at 3500 rpm for 5 min at 4°C and aspiration. Pellets were diluted in sonication Buffer (10 mM Tris-Cl, pH 8.0, 1 mM EDTA), and chromatin filaments were sheared to a range of 200–2000 bp using a Covaris S220 Sonicator, followed by two sequential steps of purification by centrifugation for 8 min at 4°C as follows: first, a low-speed round (2000 rpm) and upon supernatant isolation a second high-speed round (12 000 rpm), to avoid trapping of chromatin filaments by the cell debris, during this process. Chromatin was precleared by incubation with magnetic protein G beads (10004D, Thermo Fisher Scientific) (10 μl per sample) in 1× Reaction Buffer [1% Triton X-100, 10 mM Tris-Cl, pH 8.0, 140 mM NaCl, 0.07% DOC (sodium deoxycholate)] for 45 min at 4°C with rotation. Magnetic protein G beads were removed, and the precleared chromatin was recovered with the help of a magnetic particle concentrator (MPC) and incubated with the antibody of interest (4 μg for histone modifications and 8 μg for TFs, coactivators, and RNA pol II) in 1× Reaction Buffer overnight at 4°C with rotation. Magnetic protein G beads (8 μl/μg of antibody) were added to the reaction and coupled with the antibody for 2 h at 4°C with rotation, followed by an additional step of incubation for 1 h at room temperature with rotation. Immobilized beads–antibody–chromatin complexes were recovered and washed three times, by the addition of 1 ml of 1× Wash Buffer (1% Triton X-100, 10 mM Tris, pH 8.0, 250 mM NaCl, 0.14% DOC, and 0.1% SDS), gentle finger flick, incubation for 4 min on ice, and aspiration, with the help of an MPC. A final washing step was carried out at room temperature. Washed immobilized beads–antibody–chromatin complexes were subjected to Proteinase K treatment (50 μg enzyme, 0.5% SDS, and 100 μl total volume of the digestion reaction) (Proteinase K, recombinant, PCR Grade, 3115836001, Sigma–Aldrich) for 3 h at 55°C followed by reverse cross-linking overnight at 67°C. ChIPed DNA fragments were extracted using AMPure XP beads (A63881, Beckman Coulter). Multiplexed-DNA-libraries were prepared according to a published protocol [19] with modifications. More specifically, the NEBNext Multiplex Oligos for Illumina (Index Primers Set 1 and 2) (E7335S and E7500S, Illumina) were used to generate compatible ends to the DNA fragments. Samples were sequenced in a 75 bp single-end mode using the NextSeq 500 sequencer or in a 100 bp paired-end fashion using the NovaSeq 6000 sequencer at the Greek Genome Center at BRFAA.

### ChIP-STARR-seq experiments, recombined-plasmids bacterial libraries construction and transfection in HeLa cells, STARR-cDNA-multiplexed-library preparation, and NGS-run

ChIP-STARR-seq assays were conducted based on the original STARR-seq protocol [22] with modifications. The strategy of recombination of the ChIPed fragments rather than randomly sonicated genomic DNA or customized oligo pools eradicated the limitations of massive-in-parallel episomal plasmid examination studies. The latter can be dictated by, e.g. the necessity for enormous library complexity [23], the cytoplasmic-DNA-driven noncanonical induction of IFN/immune pathways [24], the stress/damage induced in cells during transfection, etc. An oligo-inserted Sp1-binding site (5′-CCCGCCC-3′) was cloned in the original STARR-vector [22], to facilitate DNA–DNA communication, as previously described [25], between the promoter and the recombination site of ChIPed elements that is located at the 3′ prime of the GFP reporter gene, immediately downstream of the translational stop codon and immediately upstream of the polyA signal, thus increasing the sensitivity of the assay. The 1xSp1-STARR-seq vector was linearized with restriction enzymes AgeI-HF (NEB, R3552) and SalI-HF (NEB, R3138), prior to agarose-gel electrophoresis, extraction, and two-step purification [NucleoSpin (740609.50S, MACHEREY-NAGEL) and MinElute PCR Purification Kit (28004, QIAGEN)]. IRF3- and p65-ChIPed-NGS-sequenced DNA fragments, derived from HL ChIP-seq experiments (SVI, 6 h), were subjected to end-repair, A-tailing, NebNext-adaptor molecular-indexing, and limited PCR amplification with STARR-recombination primers [22]. This process led to the bidirectional tagging of the DNA fragments with 15 nt compatible with the AgeI and SalI recombination sites of 1xSp1-STARR-seq vector. Recombination reactions were conducted by the InFusion HD Cloning Kit (639650, Takara Bio) and transformed into NEB 10-beta ultra-efficient bacteria (NEB 10-beta Competent *E*.*coli*, C3019H). To increase the yield of the process, multiple reactions were set up simultaneously following a maximum ratio of 1/10 (v/v) of recombination mix/competent cells, to limit toxicity, thus increasing the transformation efficiency and consequently the complexity of the libraries generated. Bacteria were recovered and then grown in 1 l of LB medium enriched in ampicillin for <12 h at 32°C. One milliliter of the culture was grown in LB plates, following plasmid isolation and digestion, to estimate the recombination efficiency. Approximately 3–5 × 10^6^ unique clones comprise the IRF3- and p65-STARR-libraries that were extracted from the bacterial cultures using the Nucleobond Extra Midi Plasmid Isolation kit (740410.50, MACHEREY-NAGEL) and transfected in 40 × 10^6^ HeLa cells by Lipofectamine 2000 (11668-027, Thermo Fisher Scientific). Twenty-four hours post transfection, the cell culture was equally split and either infected with Sendai virus (SVI, 6 h), or mock-infected, and utilized as control (SVI, 0 h). RNA was isolated using the TRI reagent (T9424, Sigma–Aldrich), followed by polyA selection with Dynabeads Oligo (dT)_25_ (61005, Thermo Fisher Scientific) and DNaseI treatment (DNaseI recombinant, 04716728001, Roche). Complementary DNA (cDNA) was generated using SuperScript III Reverse Transcriptase (18 080 044, Thermo Fisher Scientific). A STARR-vector-specific primer [22] was used to amplify cDNA derived exclusively from the reverse-transcription of STARR-transcripts instead of total cell transcripts, followed by nested-PCR-amplification using an exons-specific primer [22] that flanks an intervening synthetic intron of the 1xSp1-STARR-seq vector, to diminish the amplification of plasmid DNA, and subsequently the contamination of the multiplexed library. IRF3- and p65-STARR-libraries were isolated post-transfection from ∼1 × 10^6^ HeLa cells using the QIAprep Spin Miniprep Kit (27104, QIAGEN) and subjected to nested-PCR-amplification, followed by the generation of input-post-transfection-STARR-libraries utilized as controls to estimate the recovery ratio of the experiments. Samples were subjected to NGS using single-end High Output V2 kit (75 cycles) (FC-404-2005, illumina) with illumina NextSeq 500 sequencer at the Greek Genome Center at BRFAA. ChIP-STARR-seq libraries transfection and downstream processes were conducted in technical replicates.

### *Cis*-Hotspots (CHs) mapping

*Cis*-Hotspots (CHs) were mapped by epigenome-wide, multilayered, sequential intersections of NGS peaks. In detail, the hierarchical intersection of DNaseI-seq, H3K27ac-, MED1-, and RNA pol II-ChIP-seq datasets led to the identification of chromatin loci marked by the entire repertoire of the above epigenetic factors/marks in naïve and virus-infected HeLa and Namalwa cells. CHs (co)-occupied by IRF3, p65, and CBP were identified by the intersection of the corresponding peaks obtained from the ChIP-seq assays (SVI, 6 h).

### QRTMs on chromatin microenvironments proximal to TSSs (±2 kb), virus-inducible epigenomics signals, and SHAe

Heatmaps that depict the RPKM normalized signal distribution and aggregation plots that depict the mean signal enrichment across selected genomic loci were generated by deeptools package with “computeMatrix” and reference-point mode. The reference points utilized for the analyses are (i) for QRTMs on TSSs (±2 kb); the +1 bp with –beforeRegionStartLength and –afterRegionStartLength:2000, (ii) for QRTMs on virus-inducible peaks; the center of the peak, (iii) for QRTMs on SHAe; the center of the element. plotProfile and plotHeatmap from deeptools package were used to visualize signal density [26].

### SEs and tEs authentication

ROSE algorithm [27, 28] was employed to identify tEs and SEs in naïve and virus-infected HeLa and Namalwa cells. ROSE algorithm was employed through Python 2.7.18, R (4.1 version), and SAMtools (1.10 version) and implemented locally in Ubuntu Windows subsystem. The ROSE algorithm scripts and the hg19 gene annotation file are downloaded from http://younglab.wi.mit.edu/super_enhancer_code.html. The hg19 gene annotation provides the necessary information for the TSSs utilized during the stitching procedure described below. The analyses were conducted on BAM files derived from paired-end or single-end ChIP-seq data. H3K27ac- and MED1-ChIP-seq total peaks initially identified from the ChIP-seq analysis in naïve (SVI, 0 h) and virus-infected (SVI, 6 h) cells are subjected to ROSE analysis. BAM files are properly sorted and indexed (alignment coverage) for the H3K27ac- and MED1-ChIP-seq experiments and the corresponding input sample that is used as background. ROSE algorithm is implemented separately for each set of BAM, peaks, and input files that correspond to the same experiment, by the application of the ROSE_main.py script. For this step, the stitching distance for the peaks is selected to 12.5 kb (-s 12.5 kb) to assemble constituent enhancers from H3K27ac or MED1 peaks in close proximity. The peaks surrounding TSSs (±2 kb) are excluded from the stitching procedure (-t 2000), unless residing within a constituent enhancer upon stitching. The ROSE algorithm then uses these stitched regions to quantify the signal both for the markers/factors of interest (e.g. H3K27ac and MED1) and the input signal for each stitched region. The ROSE algorithm ranks the stitched regions based on the difference of the signals’ intensities between the marker (H3K27ac or MED1) and the input. The resulting value is computed in RPM (reads per million). The stitched regions are ranked in ascending order based on the above process. A cut-off (inflection point) is then automatically computed and applied by the ROSE algorithm. This step is used by the ROSE algorithm to distinguish tEs from SEs. ROSE algorithm outputs the displayed rankplots, and the coordinates of tEs and SEs.

### Chromosomal ideograms

e-Karyotypes that illustrate the genomic loci of interest across human chromosomes (hg19) were generated by the RIdeogram package [29] with minor in-house modifications. Gene density was specified from the human genome annotation (GENCODE) through the GFFex function, and the human karyotype was utilized to stipulate the exact genomic coordinates of each displayed item. SEs, SHAe, and vruDEGs were plotted to their exact chromosomal positions and illustrated by diverse colors, symbols, and distances through the ideogram function. Selected vruDEGs names were manually typed upon e-karyotypes construction.

### Assignment of SEs to genes

*In cis* attribution/assignment of SEs to genes was performed based on (i) the genomic proximity and (ii) the gene expression characteristics, according to published strategies [30]. The geneMapper function of the ROSE package was utilized to map embedded, closest, and proximal genes. Bidirectional scanning was applied within a distance of ± 0.5 Mb from the center of each SE. Expressed genes were selected from the processed data of RNA-seq based on an average expression between the two RNA-seq replicates ≥10. The list of expressed genes was then intersected with vruDEGs and defined the spectrum of those that are SEs-associated (±0.5 Mb).

### Proximity assessments between SHAe and vruDEGs

A published workflow [31] was mainly followed. IRF3- and p65-SHAe proximity was evaluated first by determining the absolute distance of each element to the nearest Transcription Start Site (TSS) of the 466 (common and cell-type-specific) vruDEGs in HeLa. The expected backgrounds were determined based on the distances from the nearest TSSs of the 466 HeLa vruDEGs of randomly sampled cohorts of equal number (1949 for IRF3- and 1601 for p65-SHAe), including (i) STARR-active non-virus-inducible IRF3- and p65-ChIP-STARR elements, and (ii) elements from the rest of the human genome. The background from the whole human genome was determined using Bedtools “makeWindowsbed” function in order to bin the human genome in 300 bp same-size regions. Next, hg19 ENCODE blacklisted regions along with IRF3- or p65-SHAe and IRF3-, p65-ChIP-peaks (HL; SVI, 6 h) were subtracted from the 300 bp binned human genome. The distances of these millions of regions of the human genome from the closest TSSs of HeLa vruDEGs were then computed and utilized as one of the two background sets. The sampling was repeated 10 000 times for both background sets, and the average numbers of elements were used as the expected values that then were compared with the observed numbers of IRF3- and p65-SHAe. Statistical significance was assessed for the first 10 kb by the application of binomial tests. For each of the 10 000 background samples, the Wilcoxon rank sum test was employed to compare the number of elements between the spectrums of IRF3- and p65-SHAe and the spectrums of the background sets. A Wilcoxon *P*-value ≤ 0.05 was considered a “success,” while a *P*-value > 0.05 was considered a “failure.” The hypothesized probability of success for the binomial test was assumed equal to 0.5. The *P*-value in the binomial test is ≤ 2.2e^−16^, thus indicating that IRF3 and p65-SHAe preferentially reside more proximal to the TSSs of HeLa vruDEGs compared to the genomic elements listed in the background expectation sets.

### TFBMs scanning for identification of IR- and κBR-HCTFBSs-SHAe, and DNA grammar, syntax, and orientation assessments

Nonoverlapping TFBSs for IRF3 and p65 were identified using the MAST tool, accessed through MEME-Suite [32]. The MEME-compatible Position Frequency Matrix (PFM) was obtained from JASPAR 2022 CORE Nonredundant vertebrates database (http://jaspar.genereg.net) and used as the bait input motif for screening, according to default parameters (MA1418.1 for IRF3, MA0050.3 for IRF1, MA0105.4 for NFKB1, MA0778.1 for NFKB2, MA0101.1 for REL, MA0107.1 for RELA, and MA1117.1 for RELB). Following similar published strategies [33], IRF3-SHAe encompassing at least 3 IRF3/1 nonoverlapping perfect binding sites within 400 bp of DNA sequence or >10 5′-AAAG-3′ IRF3 “half” sites are considered as IR-HCTFBSs. Similarly, p65-SHAe were considered κBR-HCTFBSs when encompassing (i) 3 or more NFKB1/2 or REL/A/B perfect nonoverlapping binding sites within a maximum of 400 bp, (ii) 4–14 degenerate NF-κB-binding site (5′-GGGNNNNNCC-3′) [34, 35] within a maximum of 1300 bp, variably distributed and spaced by inter-motif distances, and (iii) NF-κB “half” sites (5′-GGRR-3′) [36]. DNA syntax and orientation analyses of IRF3 and NF-κB perfect binding sites were conducted as previously described [37]. Comparisons of TFBMs between “pre-printed” (SVI, 0 and 6 h) and “newly established” (SVI, 6 h only) CHs are described in the Supplementary File.

### SHAe repeatome identification, sankey plots, and sunburst charts

RepeatMasker annotations [38–40] and the Simple Tandem Repeats catalog deposited in Tandem Repeat Finder (TRF) [41] corresponding to the hg19 genome were downloaded from the UCSC Main Table Browser. These annotations were utilized to identify SHAe overlapping with repetitive DNA. The SHAe repeatome has emerged from the results obtained from both of the above analyses. In detail, SHAe overlapping tandem repeats listed in TRF or simple repeats and low complexity regions charted in RepeatMasker are gated in TRs, while SHAe overlapping repeats from Class I and II transposons listed in RepeatMasker are categorized as DRs. SHAe that are structured as hybrids encompassing both TRs and DRs are characterized c(T:D)Rs. Sankey plots were generated using the SankeyMATIC web tool (https://sankeymatic.com/ developed by Steve Bogart). Sunburst charts for IR- and κBR-HCTFBSs were constructed using Microsoft Excel.

### Evolutionary conservation of SHAe

The PhyloP basewise conservation score [42, 43] was derived from Multiz alignment of 100 vertebrate species (precomputed phyloP100way) deposited in the UCSC Genome Browser and used to calculate the phyloP score of each SHAe. ComputeMatrix from deeptools was employed to scale each SHAe to 500 bp (scale-regions mode, –regionBodyLength 500), and phyloP score was computed for 1 bp bin (–binSize 1). The average phyloP score for each SHAe was derived from the mean values of each bin. PhyloP average scores were presented as density plots between the different categories of SHAe and the statistical significance of the difference in values was calculated through the Wilcoxon rank-sum two-sided test.

### Evolutionary history of SHAe across 100 vertebrates

An in-house Python-designed bot was used to run automated tasks online, thus facilitating the efficient screening of 3367 SHAe (IRF3 and p65) for sequence conservation using Multiz 100-way Vertebrates Alignment, phyloP basewise score, and RepeatMasker respective UCSC tracks over the UCSC Genome Browser. Manual curation was conducted to classify the 219 common IRF3/p65-SHAe as follows: 182 only in IRF3-SHAe, and 37 in both IRF3-SHAe and p65-SHAe, due to the limited overlap of their composite sequences. The bot begins by accessing the UCSC Genome Browser site for hg19 genome assembly. A text file with the genomic coordinates of interest is provided, enabling each coordinate to be entered in the search area. The resulting snapshot was then automatically saved/stored locally. Stringent manual analysis classified the SHAe according to their evolutionary history as (i) enriched in primates (e-primates), (ia) a subgroup of elements enriched in human and higher-primates (HHPe-primates) SHAe, (ii) enriched in mammals (e-mammals), and (iii) evolutionarily traced beyond mammals (b-mammals), according to the criteria described above. The entire cohort can be accessed at https://github.com/magelo-lab/3367-unique-IRF3-and-p65-SHAe.git.

### Evolutionary history of SHAe across 241 placental mammals

An in-house Python-designed bot was utilized to run automated tasks online, thus facilitating the efficient screening of 3367 SHAe (IRF3 and p65) for sequence conservation. This analysis utilizes the Cactus 241-way Placental Mammals Alignment, phyloP basewise score, and RepeatMasker, from the UCSC Genome Browser. The coordinates of SHAe were converted to the human reference genome GRCh38/hg38, using the UCSC liftOver tool (http://www.genome.ucsc.edu/cgi-bin/hgLiftOver) [43], with a minimum ratio of bases that must remap to 0.95. The bot initiated the process by accessing the UCSC Genome Browser site for hg38 genome assembly. The resulting snapshot was then automatically saved locally.

### Genomes scanning by IRF3-“cIR-motif” and IRHADs mapping

HOMER2 software tool (v4.11) and the *de novo* function were employed to identify the most enriched 25 bp length motif (-len 25) within the 482 IR-HCTFBSs. The process computed a “cIR-motif” that encompasses two perfect IRF3-binding sites (“RRAARGGAAAGGAAAGGAAAGGAAA”) that was validated for its specificity and sensitivity in probing “IRF3-clustered binding.” The background set consists of the DNA sequences of the non-IR-HCTFBSs. The Letter Probability Matrix (LPM) was archived and further utilized for downstream analyses. The “cIR-motif” was utilized as the “bait” in PWMScan tool [44] scanning of the entire genomes of diverse organisms with default parameters (*P*-value < 1e^-05^ and nonoverlapping matches). Available genomes stored in PWMScan and additional genomes retrieved from NCBI RefSeq (https://ftp.ncbi.nlm.nih.gov/genomes/refseq/) in fna.gz format and manually uploaded in PWMScan were entirely assessed. The percentage of genome coverage was calculated as follows: The number of “cIR-motif” instances multiplied by 25, and the result was divided by the exact length of each genome obtained from the NCBI Assembly Database. IRHADs mapping in human (hg19) and mouse (mm9) genomes was conducted by “cluster intervals” function from bx-python (https://github.com/bxlab/bx-python) using a minimum of three instances spaced by <75 bp as a selection criterion.

### Synteny analysis between human and mouse genomes

ChIP-seq peaks and SHAe were aligned to human (hg19) and mouse (mm9) genomes by utilizing the UCSC liftOver tool (http://www.genome.ucsc.edu/cgi-bin/hgLiftOver) [43] with “-minMatch = 0.5” and the appropriate UCSC Pair-wise Chain Alignment. The regions that successfully lift over to the human or mouse genome were considered as orthologous between the two species.

### TEs families expression quantification

To quantify the expression levels of TE families of the human genome prior to and upon virus-infection (SVI, 0 and 6 h), we processed our *transcriptomics* datasets by re-aligning the reads from fastq files without excluding from the downstream analyses the multimapped reads, a critical step that is not followed when standard RNA-seq data processing is conducted [45, 46]. RNA STAR aligner (Galaxy Version 2.7.8a + galaxy1) [47] was utilized to map the reads in the human genome (hg19) with the following parameters: –alignSJDBoverhangMin 1, –winAnchorMultimapNmax 100 –outFilterMultimapNmax 100, –outFilterMismatchNmax 10, –outSAMmultNmax -1. TEs families’ expression analysis was performed through atena R package (version 1.4.0) [48] (https://github.com/functionalgenomics/atena). The TEtranscripts expression quantification method was conducted and assessed annotations from hg19 UCSC RepeatMasker by annotaTEs function and parsefun = rmskidentity. Next, TEtransciptsParam function with aggregate by “repFamily” was applied to sum the counts derived from the individual members of the same TE family. TEs families with a sum of counts ≥8 were subjected to further differential expression analyses. vruDTTEs families were determined using edgeR (version 3.34.0) with *P*-value normalization method as follows: Benjamini and Hochberg and Normalization method:TMM with a Fold Change cut-off ≥ 1.4 and the statistical significance threshold of *P*-value ≤ 0.05. Volcano plots illustrate statistically significant vruDTTEs families were generated through an in-house ggplot2-based script in R. The same process was conducted for downregulated DTTEs.

### Scanning for SNPs across SHAe genomic loci

A curated collection of single-nucleotide polymorphisms (SNPs) associated with human autoimmune diseases, including Celiac disease (CED), Crohn’s disease (CD), juvenile idiopathic arthritis (JIA), multiple sclerosis (MS), primary biliary cirrhosis (PBC), psoriasis (PSO), rheumatoid arthritis (RA), systemic lupus erythematosus (SLE), systemic scleroderma/sclerosis (SSC), ulcerative colitis (ULC), among others, was retrieved from Gokuladhas *et al.* [49]. To ensure precision, we adhered to stringent criteria, selectively focusing on SNPs residing within or in close proximity to the SHAe (±2.5 kb). The published inclusion criteria for SNPs demand a robust association, specifying a significance level of *P* ≤ 5 × 10^−6^. The chromosomal positions of these SNPs were reported in alignment with the human reference genome GRCh38/hg38 assembly. To ensure compatibility, the coordinates of SNPs were then converted to the human reference genome GRCh37/hg19, using the UCSC liftOver tool (http://www.genome.ucsc.edu/cgi-bin/hgLiftOver) [43], employing a minimum ratio of bases that must remap to 0.95. Following this conversion, the successfully lifted-over coordinates of SNPs were intersected with the coordinates of the SHAe, previously extended bidirectionally by 2500 bp from both edges. Linkage disequilibrium (LD) calculations, represented by the squared correlation (*r*^2^) between variants within European ancestry populations, were conducted employing the LDMatrix tool [50] provided by the NIH National Cancer Institute (https://ldlink.nih.gov/?tab=home), based on the GRCh37 genome build.

### Boxplots construction

The boxplots were generated by the application of ggplot2 R package. Each boxplot illustrates the interquartile range, their upper and lower limits represent the 75th and 25th percentile, respectively, and the whiskers extend to the minimum (lowest) and maximum (highest) nonoutlier values. The central horizontal line depicts the median of the values, and the outliers are shown as dark black dots. The red dot depicts the value of the mean.

Additional methodologies for *Bioinformatics* analyses, Algorithms, Statistics, Computational Biology Tools etc., are described in the Supplementary Information File.

## Results

### Human gene expression and epigenetics states profiling delineate regulatory fingerprints of defensive cellular functions within virus-responsive chromatin microenvironments

**Figure 1.**
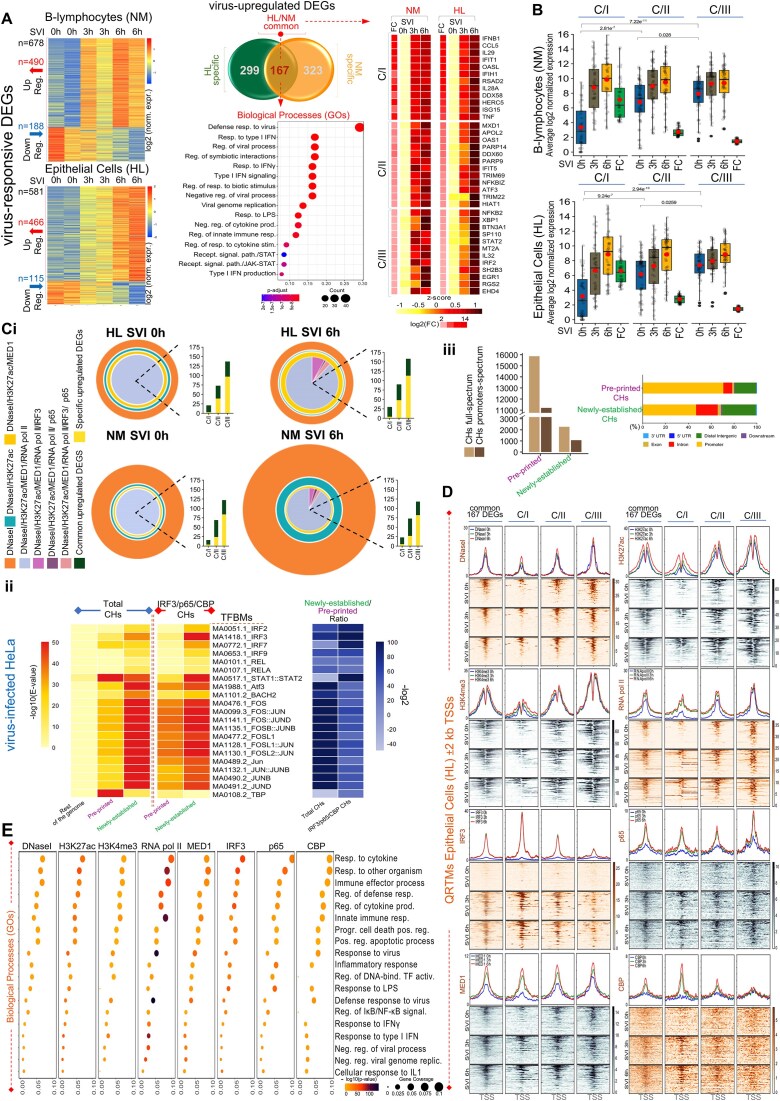
The human epigenome grants cells with regulatory underpinnings fundamental for virus-stimulated gene expression programs establishment. (**A**) Delineation of common and cell-type-specific virus-stimulated gene expression programs in human epithelial cells (HL) and B-lymphocytes (NM); Left upper and lower panels: RNA-seq heatmaps depict the transcriptional changes acquired in NM and HL [SVI; Naïve (mock-infected) (0 h) and virus-infected cells (3 and 6 h)] and revealed 678 vrDEGs in NM (490 upregulated and 188 downregulated) and 581 in HL (466 upregulated and 115 downregulated). Central upper panels: Intersection of the NM and HL vruDEGs records common (*n* = 167) and cell-type-specific spectrums (*n* = 299 in HL; *n* = 323 in NM). Central lower panel: GOs (clusterProfiler), sharply highlight the specificity of the 167 common vruDEGs for antiviral/defensive cellular processes. Right panels: Heatmaps of gene expression depicting the kinetics of transcriptional upregulation of striking examples of common C/I-III vruDEGs (e.g. secreted effectors, PRRs, and TFs). Additional data are described in Supplementary Figs S1–S3 and Supplementary Table S1. (**B**) “ON” and “OFF” transcriptional states profiling of common C/I-III vruDEGs; Box plots depict the distribution of the average log_2_ normalized expression values of each vruDEG per time point and the average of log_2_ (FC) as computed in biological replicates. The boxplots illustrate the interquartile range, their upper and lower limits represent the 75th and 25th percentile, respectively, and the whiskers extend to the minimum (lowest) and maximum (highest) non-outlier values. The central horizontal line depicts the median of the values, and the outliers are shown as dark black dots. The red dot depicts the value of the mean. Two-sided Wilcoxon rank-sum test was applied to the total common vruDEGs of each cluster (C/I: 43 in NM and 47 in HL; C/II: 63 in NM and 64 in HL; C/III: 61 in NM and 56 in HL) (Supplementary Table S1). The results depict statistically significant differences in basal levels of expression between C/I and C/II in NM (*P*-value = 2.81e^−7^), and in HL (*P*-value = 9.24e^−7^); C/I and C/III in NM (*P*-value = 7.22e^−11^), and in HL (*P*-value = 2.94e^−10^); C/II and C/III in NM (*P*-value = 0.028) and in HL (*P*-value = 0.0259). These results unveil significant heterogeneity in basal levels of vruDEGs’ expression and underscore that higher levels predominantly correlate with medium or minimal transcriptional induction upon virus-infection. Additional data for common- and cell-type-specific clusters of vruDEGs are described in Supplementary Figs S1–S3 and Supplementary Table S1. (**C** and **D**) Massive whole-epigenome- and regional-scale *epigenomics* studies in naïve and virus-infected human cells; Shown are chromatin states accessibility profiling by DNaseI-seq, and mapping of histone modifications, antimicrobial TFs, coactivators, and transcriptional apparatus by ChIP-seq. (**C**) The *in vivo* reconstitution of CHs in human cells: (i) concentric cycles demonstrate the epigenome-wide, sequential, overlaid intersections of the NGS-peaks obtained from DNaseI-seq, H3K27ac-, MED1-, and RNA pol II-ChIP-seq assays in HL and NM (SVI, 0 and 6 h). In virus-infected cells, IRF3, p65, and CBP incorporation within CHs is also highlighted. Genomic localization assessments mapped common and cell-type-specific vruDEGs *in cis* proximity (±2 kb) to established CHs. Bar graphs depict the number of CHs-associated vruDEGs and highlight that in naïve cells, C/II and C/III predominantly reside proximal to CHs, while upon virus-infection all clusters become enriched for CHs-associated vruDEGs, in HL and NM, in compatibility with the corresponding transcriptional states, as exchanged *in vivo* (additional numerical data for CHs are illustrated Supplementary Fig. S9 and Supplementary Table S2). (ii and iii) DNA Grammar assessments highlight an enriched lexicon of TFBMs legible by antimicrobial TFs in total and in IRF3-, p65-, and CBP-ChIPed, “pre-printed” and “newly established” CHs. Genomic localization assessments mapped the “pre-printed” CHs more excessively within promoter regions compared to “newly established” CHs. (**D**) QRTMs [Aggregation plots (signals’ average) and heatmaps (signals’ coverage)] applied within the TSS-proximal (±2 kb) chromatin microenvironments of residence of C/I-III common vruDEGs in HL (SVI, 0, 3, and 6 h). In naïve cells, C/II and C/III reside within “epigenetically triggered” whereas C/I in epigenetically fingerprinted chromatin microenvironments that lack hallmarks of transcriptional activation in line with their enhanced and constrained basal levels of expression, respectively. Upon virus-infection, C/II and C/III vruDEGs maintain or slightly enhance their “archetype” epigenetic features, which become divergently (co)-occupied by antimicrobial TFs and CBP, whereas C/I gradually acquire “open” chromatin, MED1, H3K27ac, H3K4me3, and RNA pol II accompanied by robust binding of antimicrobial TFs and CBP. QRTMs in NM verified the above epigenetic states (Supplementary Fig. S9). QRTMs for cell-type-specific genes are illustrated in Supplementary Fig. S12. (**E**) Virus-stimulated epigenomic fingerprints hallmark the transition from naïve-to-antiviral states in human cells and correlate with defensive cellular responses; Comparative GOs highlight tremendous specificity of the virus-stimulated *epigenomics* signals obtained from the full spectrum of DNaseI- and ChIP-seq assays for antiviral/defensive cellular processes in HL. The same analysis in NM is illustrated in Supplementary Fig. S13.

**Figure 2.**
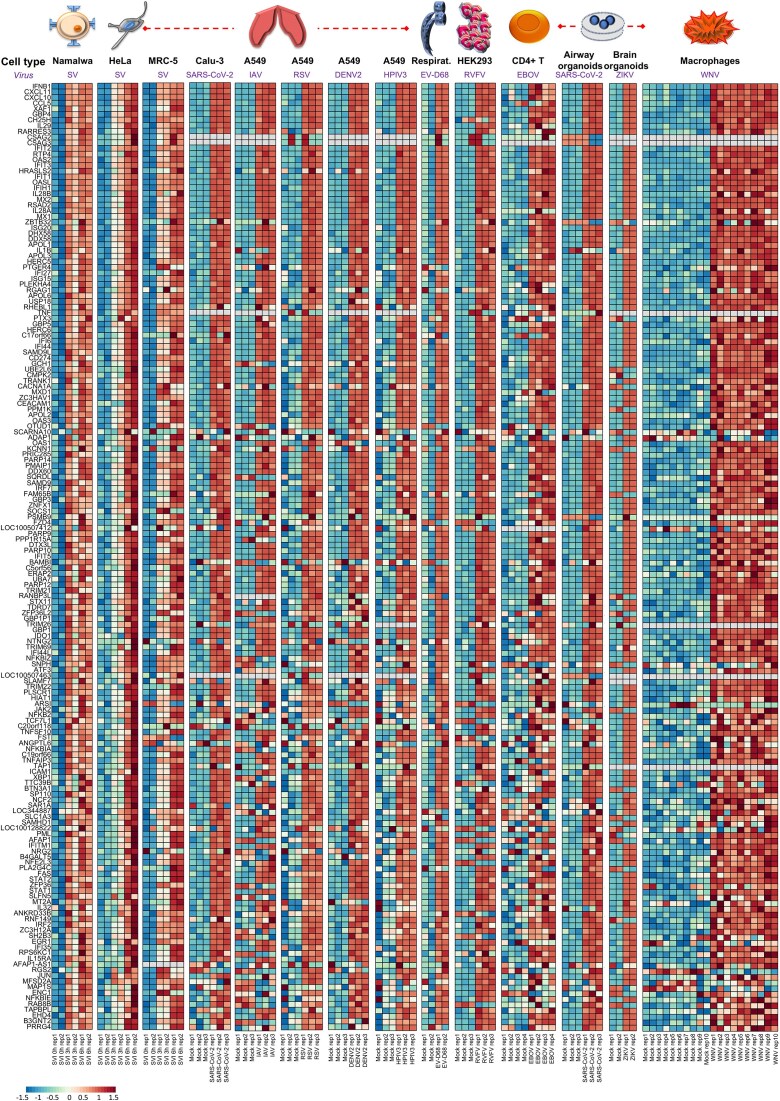
Common-Cores of human vruDEGs hallmark the establishment of defensive virus-stimulated gene expression programs in diverse human cell-types infected with distinct *Viruses*: Viral-stimulated gene expression programs mounting in human cells of epithelium, blood, lung, respiratory tract, and renal/kidney origins, and in human organoids, are comparatively investigated. Computational analyses were applied on the datasets derived from HL, NM, and MRC-5 experiments and publicly available RNA-seq datasets retrieved from Human Calu-3, male lung epithelial adenocarcinoma cells, naïve or infected with Severe Acute Respiratory Syndrome Coronavirus 2 (SARS-CoV-2; 0 and 24 h); Human A549, male lung epithelial cells, naïve or infected with: (i) Influenza A Virus (IAV; 0 and 6 h), (ii) Respiratory Syncytial Virus (RSV; 0 and 24 h), (iii) Dengue Virus 2 (DENV2; 0 and 24 h), and (iv) parainfluenza virus type 3 (HPIV3; 0 and 24 h); Human cell cultures established from upper respiratory tract specimens, naïve, or infected with enterovirus (EV-D68; 0 and 48 h); Human HEK293 female embryonic kidney cells, naïve or infected with Rift Valley Fever Virus (RVFV; 0 and 48 h); Human CD4^+^ T cells naïve or infected with Ebola Virus (EBOV; 0 and 24 h); Human Pluripotent Stem Cells (HPSCs)-derived Airway organoids naïve or infected with SARS-CoV-2 (0 h, 2-day post infection); HPSCs-derived brain organoids naïve or infected with Zika Virus (ZIKV 0 h, 3-day post infection); and Primary Human macrophages isolated from healthy donors naïve or infected with West Nile Virus (WNV; 0 and 24 h). Heatmaps depict the relative expression of the 167 common vruDEGs of NM and HL in the various human cell systems investigated prior to and upon infection with distinct *Viruses*. The results depict substantial correlation between the gene expression profiles assembled and delineate the mounting of broadly applied profiles and fates of virus-stimulated defensive/antiviral transcriptional responses within human tissues. Additional data are described in Supplementary Figs S3 and S4, and Supplementary Table S1.

To supervise how distinct human cell types mount virus-stimulated transcriptional responses, we assayed epithelial cells (HeLa; HL) and B-lymphocytes (Namalwa; NM) by functional multi-omics (RNA-seq, ChIP-seq, and DNaseI-seq), both under homeostasis (naïve) and upon sequential infection with Sendai virus (SVI; NM, HL; 0, 3, and 6 h), an agonist of IRF, NF-κB, and ATF/Jun signaling pathways [4, 51, 52] (Figs 1 and 2; Supplementary Figs S1–S14, and Supplementary Tables S1 and 2). RNA-seq assays, applied in two biological replicates, were coupled with computational analyses and identified 678 vrDEGs in NM and 581 in HL [Fold Change (FC); SVI^6 h/0 h^≥ 2, *P*-value < 0.05, (Fig. 1A and Supplementary Table S1)]. Among them, a core of 167 common (NM and HL) and two spectrums of cell-type-specific (NM, *n* = 323; HL, *n* = 299) upregulated vrDEGs (vruDEGs) were distinguished in acquiring outstanding transcriptional alterations 6 h upon infection (SVI^6 h/0 h^), in both or one cell type, respectively (Fig. 1A; Supplementary Figs S1A and S2; Supplementary Table S1). Computational assessments highlighted that 3 h upon infection (SVI^3 h/0 h^), in both cell types, ∼50% of the common vruDEGs were stimulated and maintained in the “ON” transcriptional state until 6 h upon infection (Supplementary Table S1). Such overlapping patterns were also defined in cell-type-specific vruDEGs over the course of infection (∼50% NM, ∼25% HL). These kinetics profile a step-wise *in vivo* assembly of the human defensive gene expression programs that rely on regulatory molecular mechanisms rather than nonspecific cellular responses caused by random transcriptional amplifications. Gene Ontology enrichment analysis (GOs), KEGG, and Reactome assessments addressed the immoderate specificity of the 167 common vruDEGs for defense response to virus, symbiotic interactions, IFN-signaling, additional antimicrobial cellular processes [e.g. against bacterial Lipopolysaccharide (LPS)] etc., (Fig. 1A and Supplementary Fig. S1B). Cell-type-specific vruDEGs commit to IFN-signaling, immune system functions etc., in NM, and to cell death, senescence etc., in HL (Supplementary Fig. S2A and B). In line with these gene expression characteristics, it is known that B-lymphocytes are key particles of immune mechanisms, while epithelia structure layers and barriers, regulate the number of dying and dividing cells, and they also execute defensive responses [53–56].

Hallmarks of defensive cellular responses such as PRRs, antimicrobial TFs, and secreted effectors (e.g. MDA5, IRFs, and IFNs), are encoded by the virus-stimulated transcriptomes shaped in NM and HL (Fig. 1A, and Supplementary Figs S1 and S2). In mammals, such genes frequently execute divergent rates of stimulus-induced transcriptional upregulation in the same cell type in diverse species or different cell types of the same organism [5, 6, 56–58]. The extent of this transcriptional heterogeneity, its significance for cellular physiology, and its causal (epi)genomic regulatory mechanisms have remained poorly understood in the context of human virus-stimulated response. To resolve these paradoxes, we first applied rigorous transcriptomics analyses and, based on their FC, we gated the 167 common vruDEGs in three clusters (C) (NM, HL; SVI, 0, 3, and 6 h) (Fig. 1A; Supplementary Fig. S1A and Supplementary Table S1). We identified that C/I vruDEGs [highly inducible (FC ≥ 16)] predominantly encode for secreted effectors (e.g. type I IFNs) while they nearly lack TFs and signaling components, whereas C/II [moderately-inducible (4 ≤ FC < 16)] and C/III [mildly-inducible (2 ≤ FC < 4)], primarily encode for TFs (e.g. IRFs), signaling components (e.g. IKB-α/ϵ), and viral receptors/sensors (e.g. ICAM), and they nearly abolish secreted effectors. We hypothesized that this heterogeneity in transcriptional responsiveness of the vruDEGs can be mechanistically determined *in vivo* either from variabilities in their *de novo* activation rates acquired upon virus-infection or by diversifications in their basal levels of expression established under homeostasis, or both. To address this, we computed the average normalized expression of the common vruDEGs per time point in both biological replicates (NM, HL; SVI, 0, 3, and 6 h) (Fig. 1B and Supplementary Table S1). Predominantly, C/I members (e.g. *IFNs*) exhibit low or abolish basal expression and acquire robust upregulation upon virus-infection; while a few execute enhanced basal levels followed by tremendous upregulation (e.g. *ISG15*). Contrastingly, numerous C/II and C/III members execute elevated/robust basal levels of expression and acquire an impulse of upregulation upon virus-infection (e.g. *NFKBIZ*), while a few establish minimal basal levels followed by slight upregulation e.g. *TRIM69* (Fig. 1A and B; Supplementary Fig. S1A and Supplementary Table S1). These mechanistic tendencies are transmuted *in vivo* as high (C/I), moderate (C/II), and mild (C/III) FCs of transcriptional upregulation and hallmark the heterogeneous yet precise assembly of the virus-stimulated transcriptomes, *in vivo*. Importantly, similar patterns/kinetics were recorded within the clusters (C/I-III) of cell-type-specific vruDEGs (Supplementary Figs S2A,B and S3A; Supplementary Table S1).

We extended those investigations to additional human and mouse systems of diverse origins exposed to SV or other *Viruses*, or nucleic acid analogs of viral genomes [(poly (I:C)]. Initially, we assayed human male normal diploid fetal lung MRC-5 cells by RNA-seq in two biological replicates (SVI, 0, 3, and 6 h) (Fig. 2; Supplementary Figs S3B–G  and S4B, and Supplementary Table S1). The results validated the above findings since the vast majority of the 167 common vruDEGs of NM and HL are shared with MRC-5 vruDEGs, both at 3 h (∼89%; 76/85) and 6 h (∼87%; 146/167) upon SVI. Comparisons between MRC-5 vruDEGs and HL and NM cell-type-specific vruDEGs uncovered additional spectrums of shared genes (Supplementary Table S1). GOs, KEGG, and Reactome analyses highlighted pathways/functions related to defense response to virus/external stimulus, cytokine-mediated signaling, etc. Next, we gated the 146 MRC-5 common vruDEGs in clusters and captured significant heterogeneity in transcriptional responsiveness and diversification in the patterns/kinetics of stimulation between vruDEGs of C/I and C/II-III (e.g. secreted effectors compared to regulatory molecules), as also recorded in NM and HL (Supplementary Fig. S3B and C, and Supplementary Table S1). The findings derived from HL, NM, and MRC-5 indicate the operation of common/shared regulatory mechanisms of virus-stimulated gene expression in distinct human cell types, thus implying functional consequences for organisms’ physiology. In accordance, the aberrant expression of secreted effectors in the absence of microbial challenges can lead to autoimmune malfunctions [3, 59, 60]. Thus, in naïve cells, the restricted basal levels of transcription of their coding genes sound mandatory. Oppositely, PRRs, signaling molecules and TFs also contribute to vital cellular processes without following the above mode of regulation. These diversifications reminisce integral parts of constitutive- and innate-immunity mechanisms. These are customized by the host cells to prevent unnecessary defense responses, fight microbial infections, and limit self-damage, on a finer scale [61]. Interestingly, many vruDEGs encode for molecules implicated in constitutive immunity, including restriction factors (e.g. *SAMHD1*, *IFI6*, *IFITM1*, *IFIT1*, *ISG15*, *STAT1*, etc.) [62, 63], and execute enhanced basal levels of expression in naïve NM and/or HL, and/or MRC-5 cells (Supplementary Fig. S3G). Next, we proved that our findings in human virus-stimulated defensive transcriptional response are not SV-specific, sex-determined, and/or cancerous-states-driven but instead are broadly applied in diverse male and female human cell types/tissues and organoids systems [lung, kidney, blood e.g. primary macrophages, peripheral blood mononuclear cells (PBMCs), and upper respiratory tract cells; human brain-, airway-, and ileum-organoids [64–75]] prior to and upon infection with a wide range of distinct *Viruses* that cause human diseases such as Influenza (IAV), Dengue fever (DENV2), COVID-19 (SARS-CoV-2), gastroenteritis (HAstV1) etc. (Supplementary Text S1; Fig. 2; Supplementary Figs S4–S6A; Supplementary Table S1). Comparative computational assessments applied both on bulk- and single-cell-RNA-seq published datasets [64–75], vruDEGs’ catalogs evaluations, GOs, and cellular pathways/functions investigations validated our findings and illuminated that (i) similar defensive transcriptional responses are assembled in a given human cell type/tissue upon infection with distinct viruses, and (ii) diverse human cell types/tissues execute similar transcriptional defensive responses regarding dozens of markers of the 167 common vruDEGs, when infected with the same *Virus*. Moreover, we investigated another mammalian species by conducting RNA-seq assays in mouse NIH/3T3 fibroblasts [naïve or infected with SV, in two biological replicates (NIH/3T3; SVI, 0 and 7 h)] (Supplementary Fig. S7 and Supplementary Table S1). The results confirmed substantial overlaps between human and mouse transcriptomes, pathways, and defensive/immune cellular functions. Last, computational assessments of RNA-seq datasets retrieved from naïve or poly (I:C)-stimulated human dermal fibroblasts [5] and mouse embryonic fibroblasts (MEFs) [76] validated these findings (Supplementary Fig. S6B–D and Supplementary Table S1).

Together, our transcriptomics investigations (i) define a wealth of human vruDEGs that assemble broad transcriptional activation signatures in distinct cell types upon infection with the same or different *Viruses*, (ii) track fingerprints of regulatory mechanisms applied beyond the context of human antiviral/defensive response, which might pervasively operate in mammals against microbial infections, and (iii) evidence that human cells of very distinct developmental origins exchange their states, from naïve to antiviral, by activating common (Fig. 2) and cell-type-specific gene expression alterations (Supplementary Figs S2 and S3A, and Supplementary Table S1). These are tailored to the emergence of the microbial challenge, the unique identity, physiology, functional traits, and the significance of each cell type for organisms’ homeostasis, and they are also adjusted to the cell fate decisions, as specified *in vivo*. These paramount phenomena can be supervised by organism-specific, cell-intrinsic, or/and stimulus-instructed rules and dependencies.

These modes of vruDEGs transcriptional fitness might be encrypted in segments of the human (epi)genome that harbor functional vrCRMs specialized in integrating and interpreting the exogenous/microbial stress inflicted and converting it to antiviral gene expression programs. To address such puzzling regulatory interrelationships, we exhaustively assayed the human epigenome and delineated both its “archetype” landscape and its acquired virus-inducible alterations (NM, HL; SVI, 0, 3, and 6 h) (Fig. 1; Supplementary Figs S8–S14 and Supplementary Table S2). Chromatin states accessibility profiling by DNaseI-seq assays, and mapping of histone modifications (acetylation and methylation of H3: H3K27ac and H3K4me3) antimicrobial TFs (IRF3 and NF-κB), coactivators [mediator complex subunit 1 (MED1) and CREB-binding protein (CBP)], and transcriptional apparatus [RNA polymerase II (RNA pol II)] by ChIP-seq assays were massively conducted and computationally assessed both at the whole-epigenome and regional scales. We first called and sequentially overlaid the NGS-peaks from DNaseI, H3K27ac, MED1, and RNA pol II assays in an epigenome-wide manner. We mapped the *in vivo* reconstitution of thousands of *Cis*-Hotspots (CHs) that resemble structures known to accumulate TFs [77] and/or regulatory proteins and chromatin marks (“Materials and methods” section; Fig. 1C and Supplementary Figs S8 and S9; Supplementary Table S2). Integrative Genomics Viewer analyses (IGVs) validated the enrichment of CHs for the above epigenetic features/factors connected to active transcription (Supplementary Fig. S8 and Supplementary Table S2). Concisely, in naïve cells, CHs markedly display *in cis* proximity to numerous C/II and C/III but a few C/I members (common and cell-type-specific), which primarily execute elevated basal levels of expression (∼75% HL; ∼88% NM) (Fig. 1C and Supplementary Table S1), consistent with the local formation of “epigenetically triggered” chromatin states that host stimulus-independent RNA pol II recruitment [58]. Upon virus-infection, additional CHs are established proximal to C/I-III members, and, frequently, are (co)-occupied by IRF3, p65, and CBP (Fig. 1C; Supplementary Fig. S9A and B, and Supplementary Table S2), consistent with the transcriptional stimulation of these vruDEGs upon virus infection. These factors are incorporated both within “pre-printed” (SVI, 0 and 6 h) and “newly established” (SVI only 6 h) CHs, thus implying that the stress inflicted by virus-infection is interpreted within chromatin microenvironments conveyed both via epigenetic memory [78] or become assembled from the ground state, in infected cells (Supplementary Figs S8 and S9B).

We, next, investigated the DNA Grammar characteristics of the composite sequences of CHs with an emphasis on TFBMs legible by antimicrobial TFs (expressed in NM and HL). We compared (i) the total “pre-printed” and “newly established” CHs’ cohorts, (ii) subclasses of those that feature IRF3- and/or p65-, and/or CBP-binding/recruitment, according to our ChIP-seq experiments, and (iii) equal numbers of randomly sampled loci corresponding to DNA sequences of equal length tracked from the rest of the human genome [excluding (i) and (ii), internal control]. The results revealed that in infected cells within the total cohorts, compared to “pre-printed,” the “newly established” CHs are predominantly enriched for TFBMs recognized by families/members of (i) IRFs and RELs in HL and NM, and (ii) JUN/FOS and ATF3 in HL only, and NFKB2 in NM only. Interestingly, STAT1::STAT2 TFBMs profiles are nearly balanced within CHs’ classes and TBP TFBMs are prevalent within “pre-printed” CHs compared to “newly established,” in both NM and HL examinations (Fig. 1C and Supplementary Fig. S9C). Most importantly, the profiles derived from the rest of the genomic sequences examined do not follow those of CHs’ datasets, thus underscoring the specificity of the results. Furthermore, we gained further biological insights regarding the *in vivo*-binding of DNA sequences by assessing the sub-populations of the IRF3- and/or p65-, and/or CBP-targeted-CHs. In principle, the above diversifications/enrichments are broadly recovered between the sub-populations of “newly established” and “pre-printed” CHs (Fig. 1C and Supplementary Fig. S9C). Crucially, a sharp reduction of the ratio of the TFBMs abundancies between the sub-populations of the two classes of CHs is highlighted in this analysis, thus implying that both within the “newly established” and the “pre-printed” CHs, TFBMs targeted *in vivo* by the repertoire of TFs examined, are embedded. Next, we also unraveled the diversification in TBP TFBMs by conducting genome localization assessments. The results highlight that in both cell types, rather than “newly established” the vast proportion of “pre-printed” CHs reside across DNA-sequences within or flanking the promoter regions (a set of which integrate TATA-boxes). This is evident even when the total TSSs of the genome or only those of vruDEGs were examined (ChIPseeker analyses; Fig. 1Ciii; Supplementary Fig. S9Ciii). Crucially, according to our results and the ENCODE classification [14], the discovered CHs inhabit genomic loci that host candidate *cis*-regulatory elements (cCREs), as identified by the intersection of the corresponding genomic coordinates and verified statistically (two-tailed Fisher’s exact test; Supplementary Table S2). These findings imply functional consequences of CHs *in vivo* reconstitution for human antiviral gene expression programs establishment.

TSS-proximal loci harbor *cis*-elements, such as promoters and enhancers, and can integrate signals that are directly (e.g. by TFs binding) or indirectly (e.g. by DNA–DNA communication) delivered. In eukaryotes, they also reconstitute local chromatin architectures crucial for microbial-responsive DEGs’ transcriptional regulation, such as those instructed by their composite DNA sequences (e.g. by the TATA-box, by DNA-induced allosteric conformations) [3, 5, 79]. We applied High-resolution Quantitative Regional Topographic Mapping analyses (hereinafter referred to as QRTMs) to probe *in silico* the TSS-proximal (±2 kb) chromatin microenvironments of residence of the common 167 vruDEGs (NM, HL; SVI, 0, 3, and 6 h) [Fig. 1D; Supplementary Figs S9D and S10 (IGVs); Supplementary Text S2]. The results displayed in aggregation plots and heatmaps highlight that (i) in naïve HL and NM, the TSS-proximal loci of common C/I vruDEGs primarily exhibit restricted chromatin accessibility, poor levels of H3K27ac, low levels of H3K4me3, and limited stimulus-independent RNA pol II recruitment. Oppositely, the TSS-proximal loci of C/II and, more intensively, C/III predominantly reside within “open” chromatin microenvironments marked by H3K27ac, H3K4me3, and stimulus-independent RNA pol II binding. These epigenetically divergent local chromatin microenvironments are mechanistically compatible with the evolution of constrained (C/I) or enhanced (C/II and more intensively C/III) basal levels of transcription that characterize numerous of their residents under homeostasis (Fig. 1D and Supplementary Fig. S9D). (ii) In virus-infected cells, C/I vruDEGs slightly acquire “open” chromatin architectures enriched in H3K27ac, H3K4me3, and RNA pol II. Oppositely, C/II and, more intensively, C/III maintain or modestly enhance their “archetype” epigenetic states (Fig. 1D and Supplementary Fig. S9D). (iii) In naïve cells, IRF3 and NFκB are nearly abolished from C/I-III. Upon virus-infection IRF3 binds robustly across C/I loci, weakly on C/II, and is slightly (HL) or not recruited (NM) on C/III. Alternatively, p65 is distributed between C/I-III, albeit with lower affinity compared to IRF3. This is consistent with its capabilities to bind to “open” chromatin configurations [3], to target genes pre-occupied by RNA pol II prior to stimulation [4], and to facilitate chromatin opening on inducible enhancers [80]. CBP is slightly or not recruited in naïve cells to C/I-III, yet in virus-infected cells it is intensively mapped on C/I genes, and slightly on C/II in HL only (Fig. 1D and Supplementary Fig. S9D). (iv) In naïve cells, MED1 is recruited on C/II, more intensively on C/III, but is nearly abolished from C/I. Upon virus-infection, it *de novo* occupies C/I and enhances its pre-association with C/II and C/III. Overall, MED1 follows the profiles of “open” chromatin, H3K27ac, H3K4me3, and RNA pol II, and overlays with the footprint of IRF3 (Fig. 1D and Supplementary Fig. S9D). Consistent with the corroborated functions of MED1 in antimicrobial transcription and RNA pol II/transcriptional regulators recruitment [4, 81–83], and its interactions with complexes that modify nucleosomes and impose chromatin opening [84], it (co)-founds the epigenetic and transcriptional states of vruDEGs in naïve (C/II and C/III) and virus-infected (C/I-III) human cells. Hence, prior to virus-infection, MED1’s restricted recruitment to C/I vruDEGs members can limit, but oppositely, its intensive recruitment to many C/II-III members can enhance their baseline of expression, respectively. These *epigenomics* profiles (i) were verified statistically by comparable analyses of correlations of signals’ strength and gene expression levels (Supplementary Fig. S11), and correlations of signals’ intensities (Supplementary Fig. S12A and B), and (ii) were validated by QRTMs in cell-type-specific vruDEGs (Supplementary Fig. S12C). Accordingly, the heterogeneity in transcriptional responsiveness of human vruDEGs is under robust epigenetic supervision that mechanistically relies on the function of *in vivo* reconstituted CHs/chromatin microenvironments.

We next examined the exchange of the epigenomic fingerprints between naïve and virus-infected cells (Fig. 1E; Supplementary Figs S13 and S14, and Supplementary Table S2). The virus-inducible epigenomics signals (enriched FC; SVI^6 h/0 h^) derived from DNaseI-seq assays, and ChIP-seq assays against H3K27ac, H3K4me3, IRF3, NF-κB, MED1, CBP, and RNA pol II were traced/assessed to evaluate if their enhancements over the time of infection correspond to integral parts of global epigenome (re)programming settings or regional-random episodes. First, the results highlighted both their precise on-genome distribution and gradual enrichment upon virus-infection (Supplementary Fig. S13A and C). Regardless of the factor/marker and the cell type examined, they remarkably correlate with defense cellular responses (GOs), (co)-localize at CHs that neighbor vruDEGs (SVI, 6 h), and mark endogenous sequences enriched in DNA Grammar characteristics legible by microbial-activated TFs e.g. IRFs, RELA, STATs, and FOS/JUN (Fig. 1E; Supplementary Figs S13B and S14). Such epigenomic interrelationships sound anticipated when DNA-attached cellular particles like TFs and RNA pol II are examined. However, analogous patterns of exchange are followed by coactivators [that lack DNA-binding domains (DBDs)] and chromatin modifications and accessibility (that become printed *in trans* by the function of modifying/remodeling complexes with the assistance of TFs). Therefore, the infectious stimulus triggers global epigenome reprogramming that is pervasively but not illogically diffused across human chromatin landscapes. These results underscore that human cells follow a regulatory logic to interpret microbial attacks and to achieve the optimum functional outcome. Under such stressful environmental conditions, the desired derivative of cellular response is the generation of balanced transcriptional regulatory output that is then converted to defensive gene expression programs, *in vivo*. We further explored these phenomena in additional human and mouse systems by conducting remarkably sensitive/specific IRF3- and H3K27ac-ChIP-seq experiments prior to and upon virus-infection (SVI, 0 and 6 h), in human lung cells (A549), and mouse fibroblasts (NIH/3T3). Additionally, human monocytes (THP-1) were investigated in H3K27ac-ChIP-seq prior to and upon virus-infection (SVI, 0 and 6 h). The profiles of genomic distribution, virus-inducible enrichment, antimicrobial TFBMs legibility, and defensive/antiviral functional specificity substantially validate our findings regarding virus-stimulated epigenomic reprogramming in diverse tissues in mammals (Supplementary Figs S13D and S14C; GitHub Repository; Supplementary Tables S2 and S5).

### SEs’ “decommission”, “maintenance”, and “genesis” hallmark the “naïve-to-antiviral” exchange of human cell states

**Figure 3.**
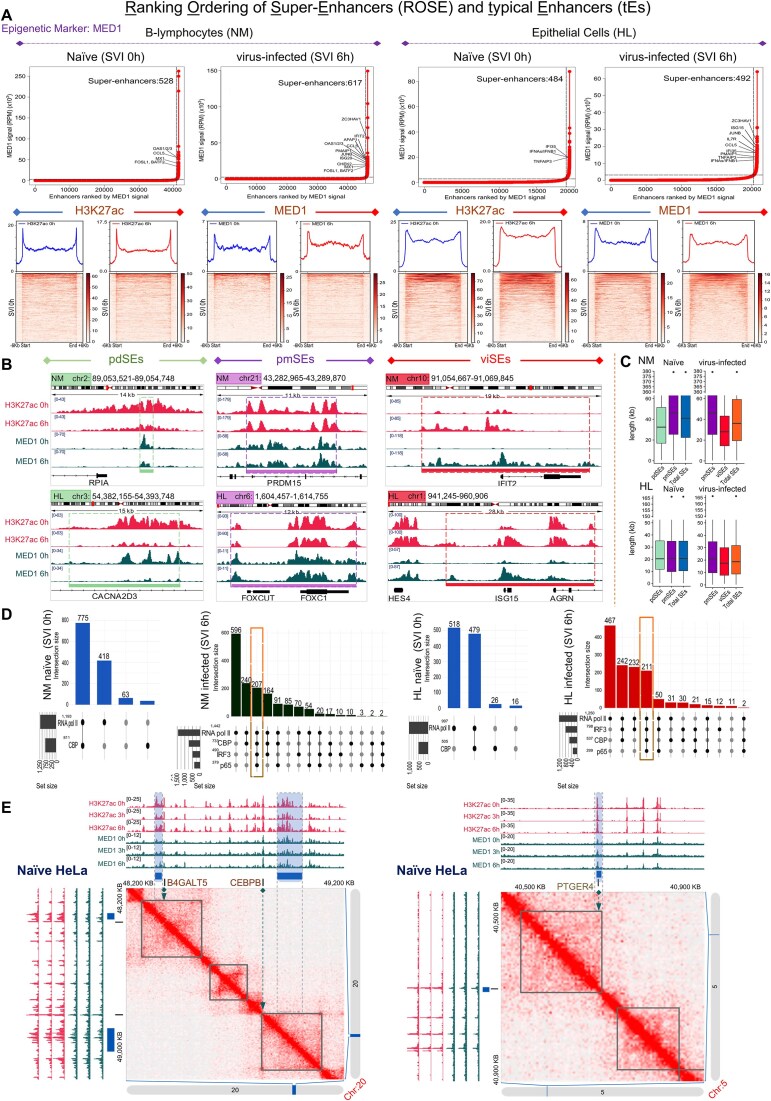
SEs’ “decommission”, “maintenance”, and “genesis” hallmark the “naïve-to-antiviral” exchange of human cell states. (**A**) Upper panels: Ranking plots from ROSE-analyses applied on MED1-ChIP-seq experiments depict the *in vivo* reconstitution of SEs and tEs, in naïve and virus-infected epithelial cells (HL) and B-lymphocytes (NM) (SVI, 0 and 6 h). Striking examples of vruDEGs residing *in cis* proximity to SEs are highlighted. ROSE-analyses applied to H3K27ac-ChIP-seq experiments are illustrated in Supplementary Fig. S15. Bottom panels: QRTMs [Aggregation plots (signals’ average) and heatmaps (signals’ coverage)] illustrate prolonged epigenomic entities corresponding to *in vivo* reconstituted SEs epigenetically marked by H3K27ac and MED1 in naïve and virus-infected (SVI, 0 and 6 h) NM (left panels) and HL (right panels). (**B**) High-resolution IGVs snapshots of *genomics* tracks illustrate MED1- and H3K27ac-ChIP-seq signals intensities and distribution across striking examples of pdSEs’, pmSEs’, and viSEs’ genomic loci of residence, in NM (upper panels) and HL (bottom panels). Horizontal lines and rectangles demarcate SEs genomic coordinates, as mapped by the ROSE algorithm. (**C**) Upper and lower panels: SEs median lengths [total and individual classes (pdSEs, pmSEs, and viSEs)]; Naïve cells: ∼40 kb in NM and ∼20.5 kb in HL. Infected cells: ∼36 kb in NM and ∼18.6 kb in HL. Median lengths are also plotted for each cell type and state. Boxplots are constructed as detailed in “Materials and methods” section and Fig. 1 legend. (**D**) Upset plots demonstrate the (co)-occupation of IRF3, p65, CBP, and RNA pol II across SEs genomic coordinates. Naïve cells (SVI, 0 h); CBP: 811 (∼62%) in NM, 505 (∼48%) in HL. RNA pol II: 1193 (∼92%) in NM, 997 SEs (∼96%) in HL; virus-infected cells (SVI, 6 h); CBP: 733 (∼46%) in NM, 537 (∼40%) in HL. RNA pol II: 1442 (∼92%) in NM and 1250 (∼94%) in HL. IRF3: 490 (∼31%) in NM and, 798 (∼60%) in HL. p65: 379 (∼24%) in NM, and 299 (∼22%) in HL. In NM and HL, 26.3% and ∼25.6% of SEs, respectively, are co-occupied by IRF3 and p65. Rectangles indicate the spectrum of “fully-equipped” SEs with the above markers examined (>200 SEs, in both cell types). (**E**) 3D investigations for SEs *in vivo* reconstitution proximal to vruDEGs in naïve HeLa cells captured several pairs of vruDEGs and SEs to coinhabit (neighbor, reside proximal, or entirely coincide) within chromosomal domains in naïve HL cells (e.g. chr20: B4GALT5/SE; chr20: CEBPB/SE; chr5: PTGER4/SE). Rectangles indicate SEs. Additional data are described in Supplementary Fig. S22 and Supplementary Table S3.

**Figure 4.**
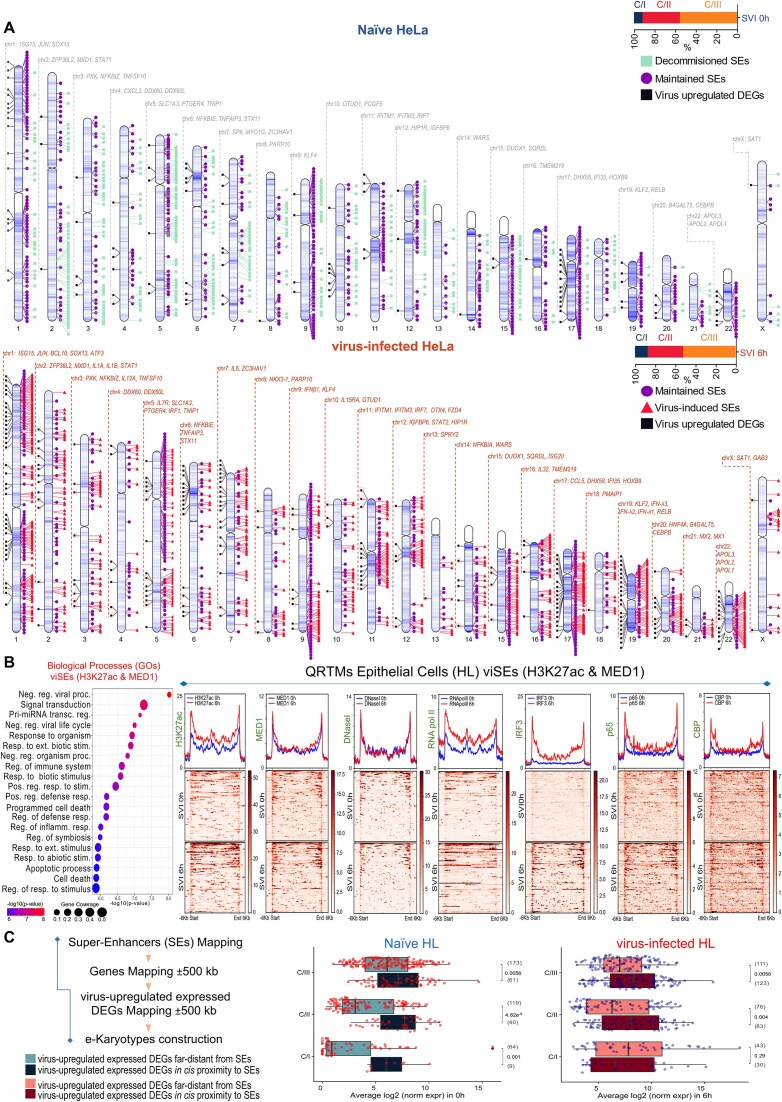
SEs genomic localization highlights extensive epigenome reprogramming phenomena that match the gene expression characteristics as steady-stated prior to and exchanged upon virus-infection in HL. (**A**) Chromosomal Ideograms (e-karyotypes) illustrate SEs *in vivo* reconstitution in naïve (upper panel) and virus-infected (bottom panel) HL (SVI, 0 and 6 h). Horizontal lines within chromosomes depict gene density. Squares label pdSEs, circles label pmSEs, and arrows/triangles label viSEs (on the right). Boxes (on the left) label vruDEGs expressed at the cell state of examination that reside within ±0.5 Mb from SEs. Striking examples of SE-associated-expressed vruDEGs are highlighted above each human chromosome. Upper and bottom right bar graphs: The percentage of allocation (%) of the 110 and 236 SEs-associated expressed vruDEGs within C/I-III in naïve and virus-infected HL, respectively. The anatomical patterns of SEs assembly match the gene expression programs as established, *in vivo* (SVI, 0 and 6 h). The same analysis for NM is illustrated in Supplementary Fig. S16. (**B**) viSEs double-marked by H3K27ac and MED1 in HL; Left panel: GOs depict substantial specificity for negative regulation of viral processes, signal transduction, immune system processes etc. Right panel: QRTMs [Aggregation plots (signals’ average) and heatmaps (signals’ coverage)] illustrate the *epigenomics* signals in naïve (SVI, 0 h, blue line) and virus-infected cells (SVI, 6 h; red line) corresponding to H3K27ac, MED1, DNaseI, RNA pol II, IRF3, p65, and CBP as distributed across the genomic coordinates. Predominantly, enhanced signals across expanded *epigenomics* entities that follow a virus-inducible mode of enrichment are captured for the markers/factors examined. The incorporation of antimicrobial TFs, coactivators, and transcriptional apparatus further indicates the functional conductance of viSEs in defensive cellular mechanisms. (**C**) SEs genomic localization and transcriptional regulation of linearly associated vruDEGs in naïve and infected HL. Left panel: A comprehensive description of the workflow applied. Flowchart showing the steps followed for the assignment of SEs to genes leading to the construction of chromosomal Ideograms (e-karyotypes) in naïve and infected HL. Numerous neighboring, overlapping, or entirely embedded expressed vruDEGs genes of C/I-III clusters were mapped in *cis* association with at least one SE in naïve and infected HL. Right panel: Boxplots depict the average log_2_ normalized expression values of SEs-associated and SEs-far-distant vruDEGs allocated in C/I-III in naïve (SVI, 0 h) and virus-infected cells (SVI, 6 h) as computed in biological replicates. Boxplots are constructed as detailed in “Materials and methods” section and Fig. 1 legend. Two-sided Wilcoxon rank-sum test computed the statistical significance in the divergence of expression levels between the SEs-associated and SEs-far-distant vruDEGs of each cluster [naïve HL: C/I *P*-value = 0.001, C/II *P*-value = 4.82e^−9^, C/III *P*-value = 0.0056; virus-infected HL: C/I *P*-value = 0.29 (nonsignificant), C/II *P*-value = 0.004, C/III *P*-value = 0.0058]. Numbers next to boxes highlight the number of SEs-associated or SEs-far-distant expressed vruDEGs, respectively. Significantly, higher diversification in basal expression levels between SEs-associated and SEs-far-distant vruDEGs for C/I-III in naïve cells, is recorded. Importantly, C/II and C/III are enriched in SEs-associated-expressed vruDEGs compared to C/I. In virus-infected cells these diversifications are more balanced. The same statistical analysis validates similar findings in B-lymphocytes (NM), as illustrated in Supplementary Fig. S16.

Noncoding genome is crowded by CRMs that structure minimal (tEs), expanded (SEs), or concatemerized (rDEs) domains, that, when (over)loaded with transcriptional complexes, gain increased potential to control gene expression [8]. SEs span in length and exhibit chromatin architecture marked by proximal or overlapping CRMs, excessive histone labeling (e.g. H3K27ac), and intense recruitment of co-activators (e.g. MED1), TFs, and RNA pol II [83, 85–87]. We attended SEs and tEs *in vivo* reconstitution and elucidated broad epigenome reprogramming phenomena at the whole-chromosomal era (NM, HL; SVI, 0 and 6 h) (Figs 3 and 4; Supplementary Figs S15–S22; Supplementary Table S3). We capitalized on our voluminous MED1- and H3K27ac-ChIP-seq datasets and applied the ROSE algorithm (Ranking Ordering of Super-Enhancers), a computational tool that accumulates stringent multi-layered criteria [27, 28]. In naïve cells, ROSE analyses ranked 1292 unique SEs in NM and 1039 in HL, whereas in virus-infected cells 1571 in NM and 1324 in HL that span median lengths of tens of kbs (Fig. 3A and C; Supplementary Fig. S15A and Supplementary Table S3). QRTMs applied across SEs genomic coordinates detected excessive signals of H3K27ac and MED1, in both cell types and states, that are superiorly fingerprinted in the linear dimension across expanded (epi)genomic entities (Fig. 3A), a hallmark of SEs’ assembly. Moreover, the intersection of SEs’ genomic coordinates and ChIP-seq peaks captured remarkable (co)-occupation by CBP, RNA pol II, IRF3, and p65 (Upset plots in Fig. 3D). Intriguingly, >200 SEs are “fully-equipped” with the above factors/marks and exhibit substantial specificity for cytokine pathways, defense-related processes etc., in each cell type (GOs) (Supplementary Fig. S17A). These findings underscore SEs’ functional fitness and commission in human antimicrobial gene expression mechanisms.

We, next monitored the anatomical patterns of SEs *in vivo* reconstitution prior to and upon virus-infection and distinguished three classes: (i) preassembled-decommissioned (pdSEs; structured only in naïve cells; ∼434 NM; ∼449 HL), (ii) preassembled-maintained (pmSEs; structured both in naïve and virus-infected cells; ∼858 NM; ∼590 HL), and (iii) virus-induced (viSEs; structured exclusively in virus-infected cells; ∼713 NM; ∼734 HL) (Figs 3B and 4A; Supplementary Fig. S16A and Supplementary Table S3). We evaluated these architectural states by computing the FC (SVI^6 h/0 h^) of the H3K27ac- and MED1-ChIP-seq signals and investigating SEs’ subclasses that feature single or double epigenetic marking. The results showed that during the naïve-to-antiviral cell states transition the vast majority of pdSEs genomic coordinates diminish the strength of these epigenetic hallmarks, as expected. In contrast, those of pmSEs and viSEs maintain a balance within their population or predominantly acquire remarkable enhancements, respectively. These patterns are applied regardless of the epigenetic factor (H3K27ac or MED1) or the marking combination (single or double) utilized to classify SEs (Supplementary Fig. S17B). Chromosomal Ideograms [29] (e-karyotypes) concisely demonstrate, at the whole-epigenome scale, that during the naïve-to-antiviral cell states transition, pdSEs are abolished, pmSEs remain structured, and viSEs are reconstituted from the ground state (Fig. 4A; Supplementary Fig. S16A and Supplementary Table S3). Hence, we elucidated that virus-infection stimulates extensive reprogramming of the human epigenome that modifies its “archetype” anatomical, architectural, and functional compartmentalization across expanded chromosomal entities that harbor SEs. Next, QRTMs elegantly accessed the epigenetic states and functional features of viSEs assembly and validated H3K27ac, MED1, DNaseI, RNA pol II, CBP, IRF3, and p65 (co)-occupancies prior to and upon virus-infection (Fig. 4B; Supplementary Figs S15B and S16B). In addition, statistically significant overlap of viSEs with “newly established” CHs was recorded [two-tailed Fisher’s exact test; HL (*P*= 5.25e^−221^), NM (*P*= 3.53e^−189^)] (Supplementary Table S3). GOs of SEs applied in cell-state contexts strengthened the above findings and highlighted, among others, defensive/immune cellular functions/pathways e.g. immune response, negative regulation of viral life cycle, etc., (Supplementary Text S3; Fig. 4B; Supplementary Figs S15A, S16B, and S17C; Supplementary Table S3). These findings illuminate that robust reprogramming of epigenomic entities that harbor SEs accompanies the mechanistic interpretation of the stress inflicted by virus-infection and its conversion to vital gene expression programs, in human cells. Apart from SEs, multitudes of tEs were also elucidated in both cell types and states (NM, HL; SVI, 0, 3, and 6 h) (IGVs) (Supplementary Figs S18–S21 and Supplementary Table S3).

Next, we mechanistically investigated SEs genomic localization and vruDEGs transcriptional regulation. First, we mapped several vruDEGs, such as *ISG15*, *CCL5*, and*IFIT2*, being entirely embedded within SEs landscapes in naïve or/and infected cells; a critical observation (Supplementary Figs S18–S20). Then, in another layer of evaluation of these architectural and functional insights, we assessed publicly available 3D Hi-C data [88] and broadly captured several pairs of vruDEGs and SEs to co-inhabit (neighbor, reside proximal or entire coincide) within chromosomal domains in naïve HL cells (e.g. chr20: B4GALT5/SE, chr20: CEBPB/SE, chr5: PTGER4/SE, and chr6: BTN3A1/SE) (Fig. 3E and Supplementary Fig. S22B). Importantly, according to our RNA-seq- and ROSE-data these vruDEGs predominantly establish enhanced basal levels of expression *in cis* proximity to SEs (Supplementary Tables S1 and S3). Apparently, SEs can reside close or far distant from their regulated genes, e.g. *MYC* [89], and this might hinder the attribution process. Therefore, we eradicated such deficiencies without affecting the resolution of analysis [30] by utilizing each SE as the reference stretch of the genome, and defined ± 0.5 Mb as a decent flanking linear distance of DNA sequence across which bidirectional screening for residential genes, including vruDEGs, was carried out by utilizing as selection criterion their absolute expression levels at the state of examination (naïve or infected cells). Hundreds of expressed vruDEGs were mapped to neighbor, overlap, or entirely coincide with SEs, under naïve and/or antiviral states, within a ± 0.5 Mb distance (Fig. 4A and C; Supplementary Figs S16A, C, and S22A). In naïve cells, 138 NM and 110 HL SEs-associated expressed vruDEGs were identified (proximal to pdSEs and pmSEs). These catalogs predominantly include C/II (*n* = 40) and C/III (*n* = 61), rather than C/I (*n* = 9) vruDEGs in HL, and NM [C/I (*n* = 8), C/II (*n* = 41), and C/III (*n* = 89)]. In virus-infected cells, the extensive epigenome reprogramming evokes the decommission of pdSEs and the generation of viSEs from the ground state, yet pmSEs remain stable. Strikingly, in virus-infected cells SEs-associated expressed vruDEGs (proximal to pmSEs and viSEs) are allocated among all three clusters in HL [C/I (*n* = 30), C/II (*n* = 83), and C/III (*n* = 123)] and NM [C/I (*n* = 66), C/II (*n* = 85), and C/III (*n* = 130)] (Supplementary Table S3). Importantly, two-sided Wilcoxon rank-sum test validated statistically significant diversifications in expression between SE-associated- and SEs-far-distant-vruDEGs, in naïve cells, that were minimized/eliminated in virus-infected cells (Fig. 4C; Supplementary Figs S16C and S22A), presumably due to the nuclear translocation and function of the antimicrobial TFs and the *in vivo* reconstitution of additional SEs. These findings illuminate that many human vruDEGs are *in cis* associated with SEs, and thus they are granted in utilizing the transcriptional regulatory output that SEs manufacture. Finally, we investigated SEs *in vivo* reconstitution and vruDEGs kinetics of expression (Supplementary Fig. S22C). We classified vruDEGs according to their temporal patterns of association with SEs as Group I (GI), associated in naïve and infected cells, and Group II (GII), nonassociated in naïve but associated in infected cells. Both spectrums exhibit substantial specificity for immune/antiviral cellular processes, thus underscoring the resolution and validity of the analyses conducted based on this classification. Dotplots depict that in NM and HL, GI members broadly establish dominant patterns of basal expression, lesser extents of transcriptional enhancements, and consequently, lower transcriptional inductions over the time of infection compared to GII. Importantly, the profiles of RNA pol II-ChIP-seq assays were utilized to calculate the signal intensities of the transcriptional apparatus occupancy in the TSSs proximal loci and substantially validated the above results (Supplementary Fig. S22C). Together these findings force our understanding regarding SEs-association and vruDEGs transcriptional fitness.

Hence, a clear correlation between SEs *in cis* proximity and fine-tuning of the transcriptional “ON” and “OFF” states of vruDEGs is recorded. Herein, we infer that human SEs (i) follow precise anatomical patterns of *in vivo* reconstitution that match the gene expression profiles of their *in cis* associated vruDEGs as steady-stated (in naïve cells) and exchanged (in infected cells). (ii) They are also (co)-occupied by coactivators, RNA pol II, and antimicrobial TFs, and (iii) they exhibit distinguished architectural and epigenomic states, and feature functional fitness in delivering transcriptional regulatory output proximal to vruDEGs. (iv) They are markedly correlated with defensive cellular processes. Remarkably, hundreds of short variants, e.g. SNPs, insertions, deletions etc., and QTLs associated with human (auto)immune diseases were mapped within SEs’ genomic coordinates, in naïve and infected cells, as further described in the “Atlas” section. Therefore, we discovered thousands of previously unknown human SEs that on their own, comprise previously unsupervised functional entities of the virus-responsive fate of the epithelial and B-lymphocyte epigenomes, and we delineated their “decommission”, “maintenance” and “genesis,” *in vivo*, during the emergence of the microbial challenge (Fig. 4A and Supplementary Fig. S16A).

### Massive-in-parallel *in vivo* functional authentication by TFs-ChIP-STARR-seq unravels “bona fide” spectrums of IRF3- and/or NF-κB-targeted vrCRMs

**Figure 5.**
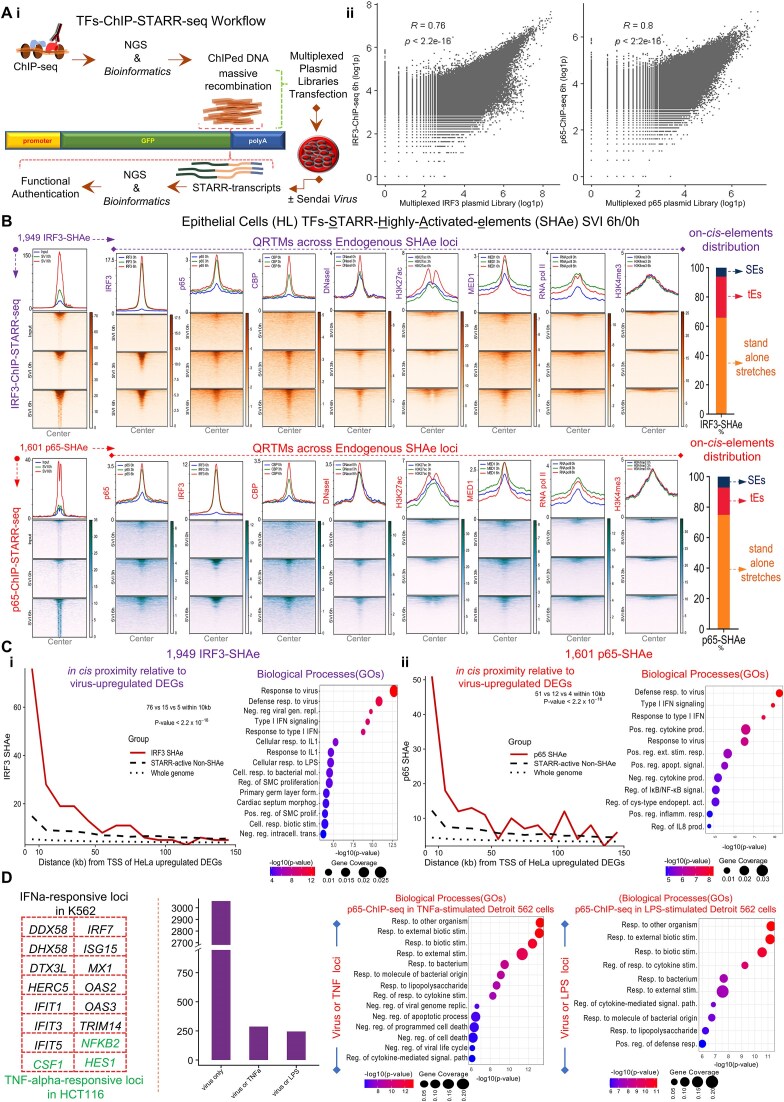
Massive-in-parallel *in vivo* functional authentication by TFs-ChIP-STARR-seq unravels “bona fide” IRF3- and NF-κB-targeted vrCRMs. (**A**) (i) The principles of TFs-ChIP-STARR-seq methodology: STARR-seq is based on the capability of functional *cis*-elements to activate the transcription of an intervening reporter-gene (e.g. GFP) when they are located several kbs downstream (3′ prime) of a promoter sequence, thus becoming self-transcribed as 5′-GFP:*cis*-element:poly (A)-3′ mRNA hybrids. TFs-ChIP-STARR-seq examination platforms are established in naïve and virus-infected HL cells. In brief, upon recombination of NGSed ChIPed DNA fragments in STARR-vectors, the generated multiplexed plasmid libraries are back-transfected in HL cells that are then subjected to virus-infection (SVI, 6 h) or remain uninfected (mock-infected; SVI, 0 h). Total mRNA is isolated and processed to generate STARR-transcripts-specific-cDNA-libraries for NGS (“Materials and methods” section). (ii) Genome-wide correlation analyses of the mapped NGS reads of each ChIP-seq experiment that was utilized to generate the respective input multiplexed-STARR-plasmid library for the IRF3-ChIP-STARR-seq (left) and the p65-ChIP-STARR-seq (right) assays. In these analyses, the human genome was fragmented *in silico* into 5000 bp bins, and the log1p (natural log + 1) reads per bin are displayed. A strong statistically significant positive correlation is demarcated, in both assays (Pearson correlation coefficient: 5Aii left: *R* = 0.76, *P*-value < 2.2e^−16^; 5Aii right: *R* = 0.8, *P*-value < 2.2e^−16^). (**B**) Left upper and lower panels: Aggregation plots (signals’ average) and heatmaps (signals’ coverage) illustrate the ChIP-STARR-seq RNA-seq signals identified in naïve (SVI, 0 h, green line) and virus-infected HL (SVI, 6 h, red line). Blue line corresponds to the signals derived from direct NGS of the multiplexed-STARR-plasmid library. In both IRF3- and p65-ChIP-STARR-seq assays, aggressive transcriptional upregulation is detected upon virus-infection, that indicates the function of vrCRMs. Computational processing resulted in the *in vivo* functional authentication of 1949 IRF3- and 1601 p65-SHAe. Central upper and lower panels: QRTMs meta-profiling of epigenomics signals across endogenous SHAe loci of residence illustrate that during the transition from naïve (SVI, 0 h) to antiviral (SVI, 3 and 6 h) cell states, IRF3- and p65-SHAe exhibit gradual enrichment of H3K27ac labeling, antimicrobial TFs binding, recruitment of coactivators and RNA pol II, yet stability of H3K4me3 labeling; whereas “open” chromatin is gradually enriched across IRF3-SHAe yet pre-established and enriched across p65-SHAe. Right upper and lower panels: Genomic localization assessments (% distribution) map IRF3- (upper bar graph) and p65-SHAe (lower bar graph) within human SEs, tEs, and “safGs”, thus validating their functional fitness/conductance for antiviral cellular processes. (**C**) (i) and (ii) left panels: IRF3- and p65-SHAe neighbor *in cis* vruDEGs: Binomial tests followed by Wilcoxon rank sum test (within the first 10 kb) verified statistically significant *in cis* proximity of IRF3- and p65-SHAe to the vruDEGs (continuous lines), compared to STARR-active-non-SHAe (dashed lines) or randomly sampled loci of equal length tracked from the rest of the human genome (square dotted lines) (*P*-value < 2.2e^−16^, for both Ci and Cii). These results show that the virus-responsive fate of the human epigenome harbors *in vivo* functional vrCRMs embedded within chromatin microenvironments linearly associated with the vruDEGs. Striking examples are illustrated in Fig. 6 and the entire catalog of SHAe-associated vruDEGs is charted in Supplementary Fig. S23B. (i) and (ii) right panels: GOs validate that IRF3- and p65-SHAe are circuited in the cellular mechanism of defensive transcriptional responses; Defense response to virus, negative regulation of viral genome replication, type I IFN signaling, etc., are highlighted among other cellular functions. (**D**) Integrative analyses of the SHAe genomic coordinates in alternative human systems challenged with immunogenic or inflammatory stimuli. The results highlight that several SHAe loci work as IFN-α responsive *cis*-elements in K562 cells. In addition, extended fractions of SHAe genomic coordinates host p65-binding in Detroit 562 cells upon TNF-a- or LPS-stimulation, and exhibit outstanding specificity for defensive and immune cellular responses, such as those against other organisms, LPS, cytokines, etc. For these analyses, the genomic coordinates of SHAe were extended bidirectionally (±2.5 kb) to avoid exclusion of biologically meaningful coincidences.

**Figure 6.**
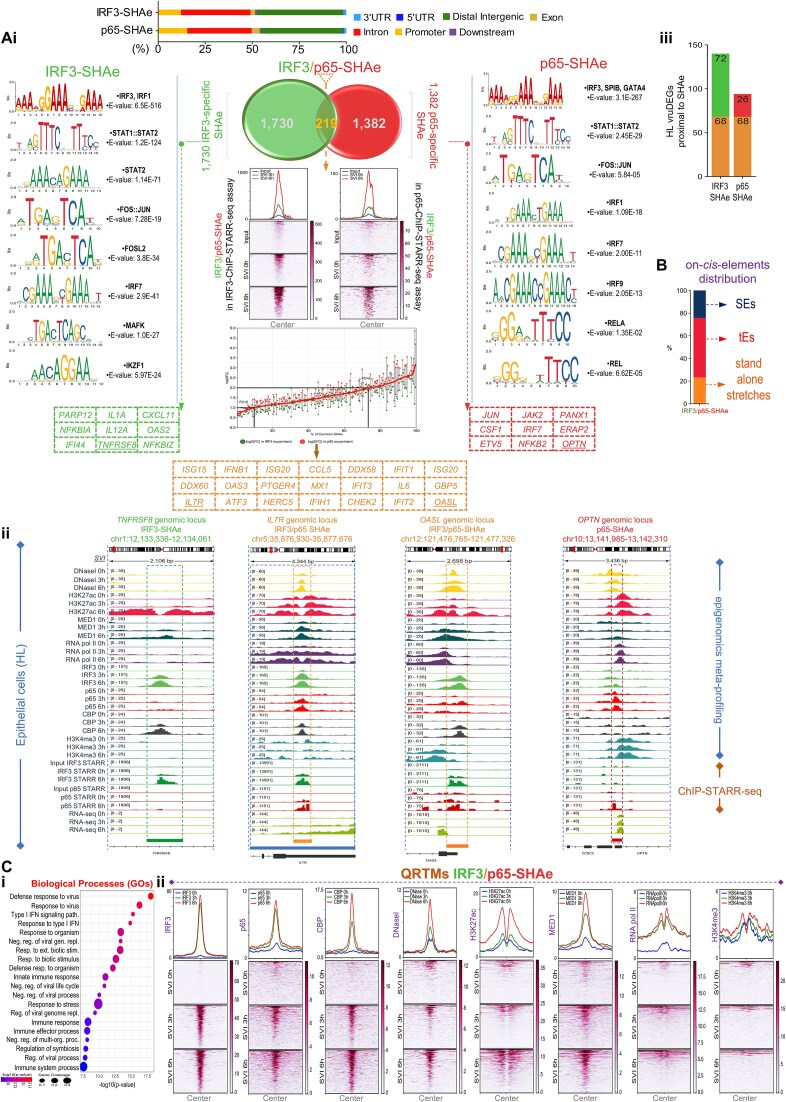
Antimicrobial TFs cooperate at the SHAe level. (**A**) (i) Central upper panel: Genomic localization assessments capture similar patterns of distribution between IRF3- and p65-SHAe within intra- and inter-genic regions. Upper left and right panels: Analyses of the lexicon of TFBSs of SHAe elucidate DNA grammar characteristics legible, among others, by members of the microbial-activated families of TFs e.g. IRFs, RELA, STATs, FOS/JUN, etc. Central middle panel: Diagram depicts a spectrum of 219 IRF3/p65-SHAe (common) within the cohorts of IRF3- and p65-SHAe, that emerged upon intersection of the respective genomic coordinates. Below: Aggregation plots (signals’ average) and heatmaps (signals’ coverage) illustrate the ChIP-STARR-seq signals captured in naïve (SVI, 0 h; green line) and virus-infected HL (SVI, 6 h; red line) upon assessments of IRF3/p65-SHAe in individual IRF3-ChIP-STARR-seq (left panel) and p65-ChIP-STARR-seq (right panel) assays. Blue line corresponds to the signals derived from direct NGS of the multiplexed-STARR-plasmid library. In both IRF3- and p65-ChIP-STARR-seq assays, aggressive upregulation is depicted upon virus-infection for IRF3/p65-SHAe. Below: Scatter plot depicts that the vast majority of IRF3/p65-SHAe (∼90%) executes strong (FC ≥ 2) virus-stimulated upregulation of STARR-transcripts (average value of FCs derived from the individual assays). Tables summarize striking examples of SHAe-associated vruDEGs (GREAT analysis; The entire catalog is charted in Supplementary Fig. S23B). (ii) Topographic maps of human SHAe endogenous loci. High-resolution IGVs snapshots of *genomics* tracks illustrate the epigenetic and transcriptional states of *in vivo* reconstituted IRF3-SHAe (*TNFRSF8* locus), p65-SHAe (*OPTN* locus), and IRF3/p65-SHAe (*IL7R* and *OASL* loci) in HL (SVI, 0, 3, and 6 h). *IL7R* locus is also discussed in Fig. 10. CHs assembly, histone modifications, and distinct patterns of antimicrobial TFs binding and CBP recruitment verify the specificity of the results. (iii) Bar graph depicts the allocation (absolute number) of SHAe-associated vruDEGs. (**B**) Bar graph depicts the distribution (%) of IRF3/p65-SHAe within SEs, tEs, and “safGs.” (**C**) (i) GOs validate statistically significant correlations of IRF3/p65-SHAe with defensive cellular functions, e.g. response to virus, type I IFN signaling, response to organism. (ii) QRTMs meta-profiling of *epigenomics* signals across the endogenous IRF3/p65-SHAe loci: During the transition from naïve (SVI, 0 h) to antiviral (SVI, 3 and 6 h) cell states IRF3/p65-SHAe exhibit gradual aggressive enrichment of “open” chromatin, and MED1 recruitment, H3K27ac labeling, strong occupation by antimicrobial TFs, CBP, and RNA pol II, while a slight change in H3K4me3 labeling is also detected. Hence, IRF3-, p65-, and IRF3/p65-SHAe work as virus-inducible transcriptional enhancers, *in vivo*.

Our findings addressed that the human epigenome grants cells critical underpinnings for defensive gene expression both under homeostasis and during the adaptation to the environmental/microbial stress inflicted by virus-infection. Resolving such perplexing interrelations at the mechanistic level requires the functional authentication of the *cis*-acting elements, *in vivo*. We elegantly accessed “in-operation” “bona fide” vrCRMs by assaying the DNA fragments isolated from IRF3- and p65-ChIP-seq experiments, described above, *in vivo*, *in parallel*, and *en masse*,via STARR-seq methodology (Self-Transcribing Active Regulatory Region Sequencing) [22–24, 90–93], following a rigorous workflow (“Materials and methods” section; Supplementary Text S4; Figs 5 and 6; Supplementary Figs S23 and S24; Supplementary Table S4). The ChIPed fragments examined correspond to the spectrum of genomic loci that are bound by IRF3- and/or p65 in HL cells 6 h upon SVI, including TSSs, tEs, SEs etc. Thousands of virus-inducible STARR-highly-activated-elements (SHAe) were identified (FC; unnormalized counts SVI^6 h/0 h^ ≥ 1.44; *P*-value < 0.05). Among them, multitudes of novel and spectrums of well-characterized virus- or IFN-inducible human enhancers (utilized as internal positive controls of our results) were captured (e.g. −20 kb *IFNβ* distal; −20 kb *OASL* distal; −2.5 kb *ZC3HAV1*; −100 bp *ISG15*) [2, 94–96], including several IFN-α-response loci previously identified by cap-STARR-seq assays in K562 cells (e.g. *IFIT3* and *OAS2*) [90], as further detailed below. Hence, despite the different cell culture systems utilized, stimuli applied, and arbitrary sources of experimental material harvested (genomic DNA [22], ChIPed DNA [23], and synthetic oligos [90, 97]), our results validate that we have precisely established TFs-ChIP-STARR-seq examination platforms in naïve and virus-infected human epithelial cells (HL), suitable for the functional authentication of vrCRMs, *in vivo*.

IRF3-ChIP-STARR-seq assays yielded 1949 *in vivo* functional SHAe (Fig. 5B and Supplementary Table S4), which execute virus-inducible STARR-transcripts-upregulation following variable strengths (FC SVI^6 h/0 h^: ∼16% 1.4 ≤ FC < 2, ∼82% 2 ≤ FC < 10, and ∼1.2% FC ≥ 10) (Supplementary Fig. S23A). This diversity ensures that our massive-in-parallel examination is not dictated by modest, leaky, or stochastic transcriptional activation episodes of the reporter plasmids. To decode the mechanism of operation of IRF3-SHAe, we first meta-profiled the epigenetic characteristics of their endogenous chromatin loci of residence (Fig. 5B). QRTMs in naïve cells depicted slightly “open” chromatin structures, labeled by low levels of histone modifications, MED1, and CBP, that nearly abolish TFs and transcriptional apparatus. This profile is replaced in virus-infected cells by “open” chromatin architectures, marked by IRF3 binding, MED1, CBP, and H3K27ac enrichment, yet at a lesser extent p65, RNA pol II, and H3K4me3. Importantly, IRF3 follows an analog mode of TF-TFBSs recognition across SHAe endogenous sequences (Supplementary Fig. S23A). Genomic localization assessments mapped 120 IRF3-SHAe within 113 unique SEs (33 in pmSEs and 87 in viSEs), 546 within tEs, and 1283 within “safGs” (HL; SVI, 6 h) (Fig. 5B and Supplementary Table S4). GOs of the 1949 *in vivo* functional IRF3-SHAe uncovered remarkable correlations with defensive cellular processes (e.g. response to virus, negative regulation of viral genome replication) (Fig. 5Ci), and analysis of the lexicon of TFBSs illuminated statistically significant specificity for microbial-activated TFs e.g. IRF3/1, RELA, STAT1/2, and FOS:JUN (Fig. 6Ai). Moreover, 141 vruDEGs (∼30%), neighbor, reside proximal, or entirely coincide with at least one IRF3-SHAe [∼42% of C/I (e.g. *ISG15*, *OASL*), ∼28% of C/II (e.g. *NFKBIZ*, *IL12A*), and ∼28% of C/III (e.g. *TNFAIP3*, *PRDM6*)] (Supplementary Fig. S23B and Supplementary Table S4). Intriguingly, the binomial test verified statistically significant proximity of vruDEGs with IRF3-SHAe but not with STARR-active-non-SHAe fragments, or with randomly sampled loci of equal length tracked from the rest of the human genome (Fig. 5Ci). The above examinations coupled with IGVs resolved that IRF3-SHAe work as virus-responsive enhancers, *in vivo* (Fig. 6Aii).

p65-ChIP-STARR-seq assays yielded 1601 *in vivo* functional SHAe (Fig. 5B and Supplementary Table S4), of which ∼2.37% executes 1.4 ≤ FC < 2, ∼81.57% 2 ≤ FC < 10, and ∼16% FC ≥ 10 virus-inducible upregulation of the STARR-transcripts (Supplementary Fig. S23A). QRTMs-based epigenetic meta-profiling of their endogenous chromatin loci of residence predominantly depicted in naïve cells “open” chromatin structures, labeled by H3K27ac and H3K4me3 modifications, and RNA pol II and MED1 occupancy. In virus-infected cells, these loci feature increased chromatin accessibility, p65 and IRF3 binding, CBP recruitment, and H3K27ac modification, yet at lesser extents, MED1, and H3K4me3 (Fig. 5B). Strikingly, p65 follows a semi-analog mode of TFBSs recognition, across SHAe endogenous sequences (Supplementary Fig. S23A). Furthermore, 115 p65-SHAe reside within 107 unique SEs (33 in pmSEs and 82 in viSEs), 287 within tEs, and 1199 within “safGs” (HL; SVI, 6 h) (Fig. 5B and Supplementary Table S4). GOs highlighted substantial correlation with antiviral cellular processes (e.g. defense response to virus, type I IFN signaling) (Fig. 5Cii), whereas the lexicon of TFBSs is legible by antimicrobial TFs, e.g. RELA, IRF3/1, STAT1/2, IRF7, and FOS:JUN (Fig. 6Ai). Moreover, 94 vruDEGs (∼20%), neighbor, reside proximal, or entirely coincide with at least one p65-SHAe [∼31% of C/I genes (e.g. *IFIH1* and *IFITs*), ∼ 19% of C/II (e.g. *DDX60*, *IRFs*, and *IL7R*), and ∼17% of C/III (e.g. *NFKB2* and *JUN*)] (Supplementary Fig. S23B and Supplementary Table S4). Intriguingly, the binomial test verified statistically significant proximity of p65-SHAe and vruDEGs but not with STARR-active-non-SHAe fragments, or with randomly sampled loci of equal length tracked from the rest of the human genome (Fig. 5Cii). Finally, IGVs validated that, like IRF3-SHAe, p65-SHAe are epigenetically programmed to work as virus-responsive enhancers, *in vivo* (Fig. 6Aii).

We further monitored the SHAe genomic loci of residence in additional human tissues challenged with SV or alternative immunogenic and inflammatory stimuli (Fig. 5D; Supplementary Fig. S23C and Supplementary Table S4). We identified >1100 SHAe loci of residence in human lung A549 cells and >1400 in B-lympocytes NM that are marked by outstanding H3K27ac modification and/or IRF3-binding upon virus-infection (SVI, 6 h), and exhibit substantial specificity for defensive/antiviral and immune cellular functions (GOs). Moreover, a wealth of SHAe loci of residence (e.g. *ISG15*, *IFITs, OASs, IRF7*) are also activated in K562 human lymphoblasts upon IFN-α stimulation, as showed by the intersection of our ChIP-STARR-seq datasets with the promoter-centered cap-STARR-seq published results [90]. For instance, the tumor necrosis factor α (TNF-a)-responsive loci that host *NFKB2*, *CSF1*, and *HES1* that were previously characterized as NF-κB targets in human colon HCT116 cells [98] were also retrieved, and are further detailed below in the “DNA evolution” section. In addition, we assessed publicly available p65-ChIP-seq datasets [2] from assays applied to human nasopharyngeal epithelial Detroit 562 cells, naïve or stimulated with TNF-a or bacterial LPS. Intersection of our ChIP-STARR-seq and p65-ChIP-seq datasets retrieved hundreds of common instances, that exhibit significant specificity for defensive/antiviral and immune cellular functions (GOs). Hence, this survey of integrative analyses deliver data that broadly verify/expand our insights regarding SHAe *in vivo* operation in distinct human tissues upon challenging with alternative stressful microbial (viral or bacterial) or immuno-inflammatory stimuli. These results postulate that apart from the virus-responsive fate the discovered SHAe may functionally integrate into alternative microbial-responsive fates of the human epigenome established upon other infections.

It is renowned that IRF3 and p65 co-regulate many mammalian microbial-inducible genes [4]. We validated that our individual massive-in-parallel TFs-ChIP-STARR-seq assays meet the required specificity and sensitivity to recover such functional epigenomic interrelationships at the SHAe level. Intersection of SHAe’s genomic coordinates captured 219 common IRF3/p65-SHAe (∼11.2% of the IRF3-SHAe; ∼13.7% of the p65-SHAe) (Fig. 6Ai,ii and Supplementary Fig. S23B). These predominantly (∼90%) execute robust virus-stimulated upregulation of STARR-transcripts (FC ≥ 2), as computed by the average value of the individual FCs (Fig. 6Ai). QRTMs-based epigenetic meta-profiling depicted highly aggressive virus-inducible profiles of IRF3, p65, CBP, H3K27ac, MED1, H3K4me3, and RNA pol II across IRF3/p65-SHAe (Fig. 6Cii), compared to those of IRF3- and p65-SHAe (Fig. 5B). This result underscores the cooperation of IRF3 and p65 on IRF3/p65-SHAe. Genomic localization assessments, GOs, evaluations of *in cis* proximity with vruDEGs, and IGVs confirm that IRF3/p65-SHAe work as virus-responsive enhancers, *in vivo* (Fig. 6A, B, and Ci; Supplementary Fig. S23B). For example, 68 vruDEGs that correspond to known (e.g. *IFNβ* and *OASL*) [2, 4, 6] and novel IRF3-NF-κB co-targets (*ATF3*, *CHEK2*, *DDX58*, *KLF4*) neighbor, reside proximal or entirely coincide with at least one IRF3/p65-SHAe (Supplementary Fig. S23B and Supplementary Table S4).

Next, we compared the signals’ intensities of ChIP-STARR-seq with those of DNaseI-seq and ChIP-seq assays across the SHAe endogenous loci of residence and depicted sharp linear associations/modes. Importantly, TFs, coactivators, RNA pol II, histone marks, and chromatin accessibility signals’ intensities exert statistically significant correlations with those of SHAe-STARR-seq (Supplementary Text S5 and Supplementary Fig. S24), thus underscoring that the functional conductance of those vrCRMs is underlined/supervised by epigenomic mechanisms. Therefore, the above functional, epigenetics, mechanistic, topological, and statistical findings authenticate that human “bona fide” IRF3-, p65-, and IRF3/p65-SHAe work as virus-inducible enhancers, *in vivo*. The vast majority of those vrCRMs comprise previously unidentified architectural and functional composites of the virus-responsive fate of the human (epi)genome.

Remarkably, dozens of SNPs, and other short variants such as insertions, deletions etc., and QTLs associated with human (auto)immune diseases reside within or vastly proximal (±2.5 kb) to SHAe’s genomic coordinates, as further described below in the “Atlas” section. Intriguingly, lift-over analyses highlighted 882 out of the 1949 IRF3-SHAe (∼45%) and 805 out of the 1601 p65-SHAe (∼50%) being orthologous in mice (Supplementary Fig. S23D). In light of these findings, and considering the published notion that IRF and NF-κB families may have co-evolved during the transition from uni- to multi-cellular organisms [99], we oriented our investigation towards monitoring the evolutionary history of SHAe.

### Human genome evolution, DNA grammar and syntax, and phylo*genomics* investigations trace variable evolutionary hierarchies and diverse architectural and functional features of human SHAe

**Figure 7.**
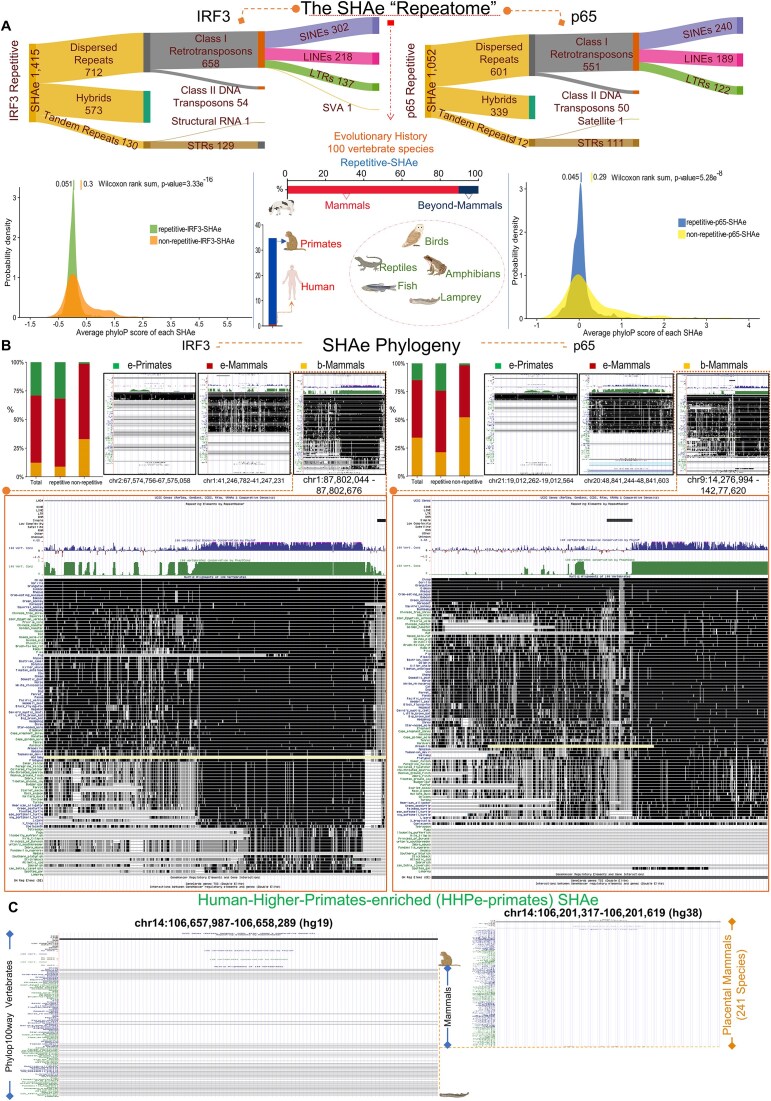
Tracing the evolutionary origins of SHAe. (**A**) The phylogeny of the human SHAe Repeatome; Upper left and right panels: Sankey plots depict the distribution of the 1415 Repetitive-IRF3-SHAe (left) and the 1052 Repetitive-p65-SHAe (right) in between the diverse classes of repetitive DNA. DRs, TRs, and hybrids of those including c(T:D)Rs composite the initial nodes (entities) of the diagrams that are then subclassified in additional linked nodes including class I retrotransposons (LINEs, SINEs, and LTRs, class II DNA transposons, STRs etc.). In both Repeatomes, DRs are the predominant class followed by hybrids and STRs. Central bottom panels: Monitoring of the evolutionary history of repetitive-SHAe within the cohort of the 100 vertebrates examined traced the vast majority (∼90%) of those upon the emergence of mammalian species (e-mammals), whereas a limited spectrum displays a more prolonged evolutionary history and captured in the genomes of nonmammalian vertebrates (b-mammals). Left and right bottom panels: Density plots depict statistically significant differences between the evolutionary conservation of repetitive- versus nonrepetitive- IRF3- and p65-SHAe, as computed by the average PhyloP score of each element [two-sided Wilcoxon rank sum test (*P*-value = 3.33e^−16^ for IRF3-SHAe and *P*-value = 5.28e^−8^ for p65-SHAe)]. In principle, repetitive-SHAe are “newly acquired” in the human genome compared to nonrepetitive. (**B**) Upper panels: Bar graphs illustrate the evolutionary distribution of human IRF3- and p65-repetitive-SHAe between the classes of e-primates, e-mammals, and b-mammals accompanied by representative examples of DNA sequence conservation within the cohort of 100 vertebrates. These patterns validate the evolutionary hierarchies of IRF3- and p65-Repeatomes. (**C**) The evolutionary history of human higher primates-enriched SHAe (HHPe-primates); Side-by-side monitoring of the conservation of vrCRMs within the 100 vertebrates’ (left panels) and the 241 placental mammals’ (right panels). The entire DNA sequence of this SHAe is not traced in the genomes of other organisms in both cohorts and is also classified in G3 cCREs. Part of Fig. 7 was created in BioRender: Agelopoulos, M. (2025) https://BioRender.com/x93p313

**Figure 8.**
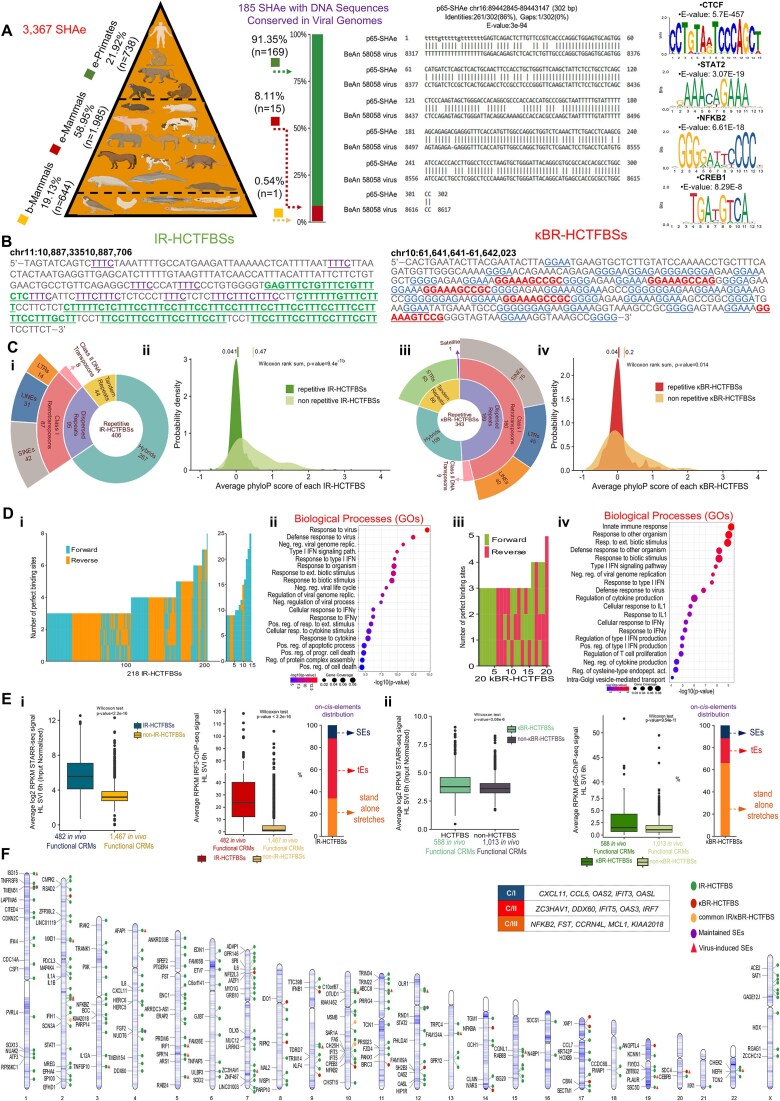
DNA Grammar and Syntax assessments and phylogenomics investigations mapped the evolution of hundreds of homotypic clusters of TF-binding sites (HCTFBSs) within composite sequences of the IRF3- and p65-SHAe. (**A**) The pyramid of the >180 human SHAe that encompass extended composite DNA sequences (>150 bp) highly conserved in one or multiple viral genomes. Central panels: Analyses of the distribution among the classes of SHAe. Right panels: A striking example of pairwise alignment of the DNA sequence of the SHAe and BeAn 58058 virus genome. TFBMs analyses applied exclusively on the segment of SHAe that exhibits conservation with viral genome(s) highlights that CTCF, STAT2, NFKB2, and CREB1 TFBMs are among the top-ranked. (**B**) Striking examples of IR- (left) and κBR-HCTFBSs (right) composite DNA sequences which illustrate the density of perfect, degenerate, and half TFBSs of IRF3 and NF-κB, respectively. Magenta (IRF3) and blue (NF-κB/Rel) letters label half sites, green letters perfect IRF3 binding sites, and red letters NF-κB/Rel-binding sites. (**C**) (i) and (iii): Sunburst charts illustrate the distribution of HCTFBSs-SHAe within distinct classes of repetitive DNA. In principle, IR-HCTFBSs (*n* = 406) and to a lesser extent κBR-HCTFBSs (*n* = 343) reside predominantly within the repetitive-SHAe spectrums, consistent with their concatemerized structures. IR-HCTFBSs predominantly bear hybrid sequences, while within κBR-HCTFBSs, the DRs are vastly represented. (ii) and (iv): Monitoring of the evolutionary history underscores that repetitive-IR-HCTFBSs (*n* = 406) and repetitive-κBR-HCTFBSs (*n* = 343) are “newly acquired” compared to nonrepetitive-IR-HCTFBSs (*n* = 76) and nonrepetitive-κBR-HCTFBSs (*n* = 245), respectively. Statistically significant differences of PhyloP scores between the spectrums of repetitive and nonrepetitive HCTFBSs were identified [two-sided Wilcoxon rank sum test (*P*-value = 9.4e^−15^ for IR-HCTFBSs and *P*-value = 0.014 for κBR-HCTFBSs)]. This analysis is complemented in Supplementary Fig. S28. (**D**) (i) and (iii): DNA Grammar and Syntax analysis on the composite sequences shows that the core elements of both IR-HCTFBSs and κBR-HCTFBSs exhibit “Head-to-Tail” and to a lesser extent “Head-to-Head” orientation of their embedded perfect TFBSs, a feature that reminisces TRs expansion within the genomes. (ii) and (iv): GOs on IR-HCTFBSs and κBR-HCTFBSs show substantial specificity for defensive and immune-response cellular processes. (**E**) (i) and (ii): Left and middle panels: IR-HCTFBSs and κBR-HCTFBSs host robust/stronger virus-inducible binding of IRF3 and p65, respectively, and execute stronger/wealthier virus-inducible STARR-transcripts upregulation *in vivo* compared to non-IR- and non-κBR-HCTFBSs, respectively [two-sided Wilcoxon rank sum tests (Di left: *P*-value < 2.2e^−16^; Di middle: *P*-value < 2.2e^−16^; Dii left: *P*-value = 3.08e^−6^; Dii, middle: *P*-value = 9.54e^−11^)]. The Boxplots are constructed as detailed in materials and methods and Fig. 1 legend. Right panels: Genomic localization assessments map the on-*cis*-elements distribution (%) of HCTFBSs: IR-HCTFBSs: 58 are embedded within 54 unique SEs (10 elements in pmSEs and 48 elements in viSEs), 260 within tEs, and 164 “safGs”. κBR-HCTFBSs: 69 are embedded within 66 unique SEs (15 elements in pmSEs, and 54 elements in viSEs), 131 within tEs, as verified by the ROSE algorithm, and 388 within “safGs”. (**F**) Chromosomal Ideograms (e-karyotypes) illustrate the broad *in vivo* reconstitution of functional virus-responsive IR-HCTFBSs, κBR-HCTFBSs, and IR//κBR-HCTFBSs in virus-infected HL (SVI, 6 h), across chromosomal telomeres, intra-chromosomal domains, and centromeres. Significant linear on-genome associations are marked between HCTFBSs and proximal vruDEGs (GREAT analysis) and SEs. Green circles: IR-HCTFBSs; Red circles: κBR-HCTFBSs; Orange circles: IR//κBR- HCTFBSs; Magenta circles: pmSEs; Red arrows/triangles: viSEs. The above findings validate the functional conductance of clustered *cis*-elements of diverse phylogeny that are recognized by IRF3 and/or NF-κB in virus-stimulated gene expression in human cells. Part of Fig. 8 was created in BioRender: Agelopoulos, M. (2025) https://BioRender.com/d37w614

**Figure 9.**
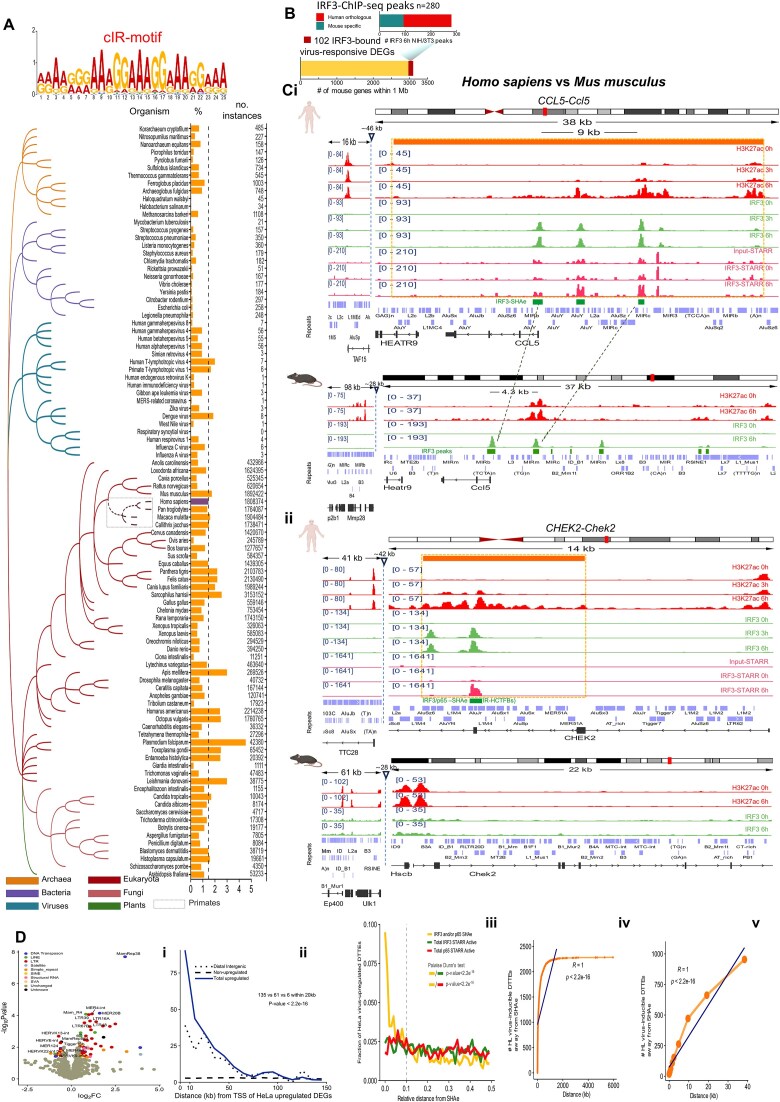
Tracing IRF3 HCTFBSs fingerprints in evolutionary recent, old, and ancient genomes, and characterization of vruDTTEs. (**A**) HOMER2 software tool (v4.11) and the *de novo* function computed the most enriched motif of an adjusted 25 bp length within the 482 IR-HCTFBSs which is termed “cIR-motif,” encompasses two perfect IRF3-binding sites (“RRAARGGAAAGGAAAGGAAAGGAAA”), and efficiently probes *in silico* “IRF3-clustered binding.” Lower panel: The 97 genomes investigated (retrieved either as default assemblies (PWMScan) or manually curated from the NCBI RefSeq database) belong to organisms with divergent evolutionary origins: (i) Metazoans: vertebrates (e.g. primates and mammals) and invertebrates (e.g. worms, insects, and mollusk), (ii) Plants: *Arabidopsis thaliana*, (iii) unicellular eukaryotes (e.g. fungi and protozoa), (iv) Prokaryotes (bacteria and archaea), and (v) and Human infectious *Viruses*. Percentage (%) of genome coverage (bar graph) and number of “cIR-motif” instances (right lane) are displayed. (**B**) Highlighted in dark are the 102 IRF3-target genes orthologous in mice and human (HL vruDEGs; SVI, 6 h). Upper bar graph: The spectrum of 102 IRF3-target genes is associated with 280 virus-inducible IRF3-ChIP-seq signals in NIH/3T3 (SVI, 6 h), 187 (66%) of which are orthologous in human according to lift-over analysis. Bottom bar plot: The 102 IRF3-target genes within the full spectrum of mouse genes that reside *in cis* proximity (1 Mb; GREAT analysis) to virus-inducible IRF3-ChIP-seq signals. (**C**) Genome innovation episodes mark the evolution of human vrCRMs: (i) The *CCL5*-*Ccl5* paradigm: Three distinguished IRF3-SHAe compose a repertoire of vrCRMs that regulate the human *CCL5* gene and reside within the modular architecture of its viSE (rectangle and horizontal line). All three of those are IR-HCTFBSs, one TSS-proximal (+237 bp), one TSS-distal (-9687 bp), and one intervening (-3866 bp) that was captured by the “cIR-motif.” The orthologous mouse locus bears two mIR-HCTFBSs (-68 bp, -4341 bp). The segment of the human intervening sequence that is missing from the mouse genome hosts the -3 866 IR-HCTFBSs, is classified in higher Primates, and harbors multiple insertions of TEs, including SINEs (Alu, MIR) and ERVs (LTR35A, LTR8). This genome innovation episode might happen *via* horizontal transmission of viral DNA and grants human cells an additional vrCRM that might facilitate the *in vivo r*econstitution of the viSE. (ii) Human *CHEK2* harbors an IR-HCTFBS that is located ∼11.3 kb downstream of the TSS (GREAT analysis), inhabits the viSE (rectangle and horizontal line) of the gene, and coincides with its third exon (all classified in e-primates). It is structured by TR and Alu elements, and has viral origins. This genome innovation episode leads to the generation of another vrCRM, presumably the “birth” of a viSE, and the encoding of additional amino acids that lead to a new isoform of the human protein (exonization). (**D**) Dissecting the human virus-stimulating TEs-encoded transcriptome; (i) TEtranscripts tool analyses identified 60 distinct families of vruDTTEs in virus-infected HL (SVI, 6 h) e.g. MER107, LTR26, and MER57B1. (ii) Binomial test followed by Wilcoxon rank sum test within the first 20 kb highlight statistically significant enrichment of *in cis* proximity between vruDTTEs and vruDEGs, in HL, when total (continuous line) or distal intergenic copies (dotted line) were assessed, compared to an equal number of nonvirus-upregulated TEs (dashed line) (*P*-value < 2.2e^−16^). (iii) vruDTTEs inhabit genomic loci more proximal to SHAe (yellow line) compared to the total STARR-Active elements (IRF3; green line, p65; red line) [Dunn’s test in 0.1 cutoff in the respective relative distances (*P*-value < 2.2e^−16^)]. (iv) Bidirectional demarcation of vruDTTEs across ∼6 Mb flanking distance was conducted by step-wise increase (doubling) of the flanking sequences and utilizing the center of each SHAe as the reference point of the genome. This analysis highlights linearly increased inhabitance across ∼300 kb, which is validated in (v) by increasing the resolution of analysis within ± 40 kb flanking distances (Spearman correlation coefficient, *R* = 1, *P*< 2.2e^−16^), and mapped 222 SHAe topologically associated with core antiviral genes vruDTTEs (Supplementary Table S5). Part of Fig. 9 was created in BioRender: Agelopoulos, M. (2025) https://BioRender.com/k72m711.

We investigated SHAe biology from an evolutionary angle. We attended critical aspects and delivered novel insights regarding (i) their evolutionary roadmaps, (ii) the structural features of their composite DNA sequences, (iii) what they encode for, apart from transcriptional regulatory output, and (iv) whether they evolutionarily (re)shape transcriptional responses against microbial stimuli, like other human *cis*-elements [59] (Figs 7–9; Supplementary Figs S25–S30 and Supplementary Table S5). The *Homo sapiens* genome was subjected to evolutionarily shaping for millions of years (MY) [100], and it pervasively encompasses repetitive segments that account for 30%–40% of its length [101, 102]. We first processed SHAe endogenous sequences by Tandem Repeats Finder (TRF) and RepeatMasker [38–41] and revealed that 1415 IRF3-SHAe (∼72%) encompass repetitive DNA stretches of TRs (STRs; *n* = 129, structural RNAs; *n* = 1), DRs; *n* = 712; class II DNA transposons, class I retrotransposons (LINEs, SINEs, and LTRs, e.g. ERVs), or hybrids c(T:D)Rs; *n* = 573. The remaining 534 IRF3-SHAe (∼28%) are nonrepetitive. Analogously, 1052 out of the 1601 (∼66%) p65-SHAe encompass TRs (STRs; *n* = 111, satellite; *n* = 1), DRs; *n* = 601, and c(T:D)Rs; *n* = 339, while the remaining 549 (∼34%) are nonrepetitive (Fig. 7A and Supplementary Table S5). QRTMs meta-profiled the chromatin states of the endogenous loci of repetitive- and non-repetitive-SHAe and highlighted divergent modes of epigenetic marking and regulation (Supplementary Fig. S25A and B).

Next, we employed phylop100way basewise ranking and scored the DNA sequence conservation of the total 3367 unique SHAe (IRF3 and p65). We addressed substantial hierarchies throughout hundreds (∼300–500) of MY of evolution [37, 103–107], a time window that spans the emergence of 100 representative vertebrate species on Earth. Density plots depict the average conservation and illuminate that repetitive-SHAe are principally “newly acquired” in the human genome compared to nonrepetitive-SHAe that record a more prolonged evolutionary history (Fig. 7A, lower left and right panels). In addition, we carried out a stringent phylogenetics approach by accumulating (i) the average phyloP score for each SHAe, (ii) the length of the conserved sequences >100 bp, (iii) a sequence similarity score >60%, and (iv) the sequence alignment across multiple evolutionary relative organisms (GitHub Repository). The emerged evolutionary roadmaps record that ∼90% of the repetitive-SHAe are enriched in mammals (e-mammals) while ∼10% are traced beyond mammals (b-mammals), including ancestor species of vertebrates, e.g. birds, reptiles, amphibians, fish, lamprey etc. Intriguingly, within e-mammals-repetitive-SHAe, a spectrum of ∼34.74% is enriched in primates (e-primates) (Fig. 7A and B, and Supplementary Table S5). In sharp contrast, nonrepetitive-SHAe exhibit alternative evolutionary roots, since ∼62% are e-mammals and ∼38% are b-mammals. The latter are traced within multiple vertebrate genomes. Of the e-mammals-nonrepetitive-SHAe, the cohort of e-primates corresponds only to ∼2.5% (Supplementary Fig. S25C and Supplementary Table S5). Notably, we term these elements “enriched” instead of “specific” to emphasize that they were assessed within this cohort of 100 vertebrate genomes and, thus, any possibility of inhabiting genomes of other organisms that have not yet been (entirely) sequenced cannot be excluded.

We further monitored the evolutionary fingerprints of those SHAe across >200 genomes of placental mammals described in the study of Andrews *et al.* [108] that incorporates 0.92 × 10^6^ cCREs from hundreds of human cells/tissues stored in ENCODE [14]. The results extensively validated the evolutionary roadmaps emerged from the above analyses in the vertebrates’ cohort (Supplementary Table S5 and GitHub Repository). In synopsis, Andrews *et al.* traced a limited spectrum of cCREs (∼4.4%) beyond the mammalian clade, within the cohort of 100 vertebrate genomes, similar to our results for SHAe (∼19.1%). Importantly, statistically significant inhabitance of cCREs within human SEs in virus-infected HL (SVI, 6 h; pmSEs and viSEs) was addressed (Supplementary Fig. S25D). Moreover, 1078 common SHAe/cCREs were mapped and validated regarding their evolutionary classification. Together the above findings broadly verify the evolutionary roadmaps and deliver proofs for the *in vivo* function of cCREs in human epithelial cells upon virus infection (SVI, 6 h) (Supplementary Text S6, Supplementary Fig. S25D, and Supplementary Table S5).

Intriguingly, within the cohort of 100 vertebrates (phylop100way) a set of SHAe was recorded only in the human genome, which was then further monitored within the 241 placental mammals [108] (Fig. 7C and Supplementary Fig. S26A; GitHub Repository). A spectrum of those SHAe exhibit conservation in human relatives such as chimpanzee, monkey and lemur but still any remarkable downstream conservation in other mammals was missing. We term this small spectrum Human-Higher-Primates-enriched SHAe (HHPe-primates). Interestingly, some of those elements exhibit outstanding conservation with the genomes of human infectious *Viruses* (BeAn 58058 virus, Baboon cytomegalovirus OCOM4-37, Zika virus, and Human endogenous retrovirus K113) (Supplementary Fig. S26B and C, and Supplementary Table S5) but not with dozens of vertebrates’ genomes. These results suggest that apart from the vertical transmission, evolutionary mechanisms of horizontal transmission might have facilitated the inhabitance of such traces of ancient DNA into the genomes of mammals or primates (e.g. germline infections). We further capitalized on these findings and aligned/blasted the 3367 unique SHAe with >10000 viral genomes archived in NCBI (taxid:10239) (Fig. 8A; Supplementary Fig. S26B and C and GitHub Repository). We captured a spectrum of >180 human SHAe (>5% of the total) that encompasses extended DNA sequences (>150 bp) highly conserved with one or multiple viral genomes. We expanded this investigation and illuminated an unprecedented phenomenon: as shown in the pyramid of Fig. 8A, these SHAe, predominantly, are e-primates, followed by e-mammals, whereas b-mammals are nearly absent (only 1 SHAe mapped). Proportionally, although the e-primates-SHAe population corresponds to less than half of e-mammals and is nearly equal to b-mammals, they bear ∼10 and ∼150 times more viral-conserved stretches within their composite sequences, respectively. These results underscore that horizontal transmission might have facilitated the evolution of the virus-responsive fate of the human (epi)genome. Among the additional viral species captured, many human-specific such as Zika virus, human endogenous retroviruses K115, HCML-ARV, are listed (Supplementary Table S5). Next, we asked what these stretches deliver within the human genome and applied TFBMs analyses exclusively on the viral-DNA conserved-composite of the identified SHAe (>180). CTCF TFBMs are predominantly recovered accompanied by others legible by defense-related TFs e.g. NFKB2, STATs, CREB etc., (Fig. 8A). Thus, insertions of viral-sequences within human DNA that might happen via horizontal transmission episodes (or delivery from evolutionary relatives to which they were initially horizontally transmitted, e.g. other primates) contribute to the shaping of the virus-responsive fate of the human (epi)genome by conveying at least new TFBMs or even intact composite elements within human *in vivo* functional vrCRMs. These findings highlight putative functional associations between host–pathogens interactions with regulatory consequences for defensive gene expression that rely on the *in vivo* operation of vrCRMs.

Moreover, we investigated for inhabitance of viral DNA within the genomes of non-human vertebrate species, attempting to map candidate species-specific virus-responsive CRMs (ssvrCRMs) endowed with DNA stretches conserved in viral genomes (Supplementary Fig. S27A and Supplementary Table S5). To strengthen the likelihood of mapping *in vivo* functional ssvrCRMs, we capitalized on our IRF3-ChIP-seq experiments in mouse fibroblasts (NIH/3T3; SVI, 0 and 6 h). We lifted-over the genomic coordinates of ∼5500 virus-inducible mouse IRF3 peaks in the human genome [UCSC liftover [43]; minmap threshold = 0.5] and subtracted the orthologous sequences, thus we fished-out ∼2200 nonorthologous IRF3-bound mouse elements. These are not conserved in humans and share enhanced epigenomic potential to operate during mouse antiviral cellular response. We applied stringent criteria for the selection of ssvrCRMs (Supplementary Materials and Methods). Striking examples correspond to *cis*-elements residing proximal or within the *Ly6e* [109] and *Apobec3* [110] loci, respectively. Both these genes encode for proteins implicated in cellular functions connected to virus-infection and are classified in vruDEGs in NIH/3T3 (RNA-seq data; SVI, 0 and 7 h). Importantly, apart from the IRF3-binding on the viral-conserved DNA segment, these loci exhibit increased H3K27ac labeling, thus further underscoring their *in vivo* functional potential. We further monitored/validated their evolutionary history in 30 vertebrate species (30-Way Multiz Alignment - mm9) and identified remarkable nonattendance in the human and other vertebrates’ genomes (Supplementary Fig. S27A), for both the IRF3 peak and its viral-conserved composite segment. Hence, we delivered mouse ssvrCRMs endowed with DNA segments conserved in murine infectious *Viruses*.

We next dissected the DNA Grammar and Syntax of SHAe endogenous sequences. We marked another pervasive feature of the genomes according to which embedded TFBSs, recognized by the same TF or its family members, are arranged *in cis* and assemble HCTFBSs known to contribute to the mechanisms of transcriptional control [37, 98, 111–115]. We rigorously assessed the 1949 IRF3-SHAe from HeLa with the MAST tool by utilizing the IRF3/1 motif (JASPAR 2022) and scored “clustered-elements” [98, 116] encompassing (i) 3 or more perfect nonoverlapping IRF3/1-binding sites (each one harbors two “half” sites; 5′-GAAAG-3′ or/and 5′-AAAG-3′) [117] within a maximum of 400 bp of DNA sequence (*n* = 218, core elements), or (ii) <3 perfect IRF3/1 binding sites but 10–43 nonoverlapping “half” sites within a maximum of 1 kb (*n* = 264), variably distributed and spaced by inter-motif distances (Fig. 8B; Supplementary Fig. S28A and B, and Supplementary Table S5). The discovered 482 IRF3-repetitive-HCTFBSs (IR-HCTFBSs) predominantly (∼84%) reside within the repetitive-IRF3-SHAe spectrum, especially in hybrids, and these are in principle “newly acquired,” compared to those (∼16%) belonging to the nonrepetitive DNA segments (Fig. 8Ci, Cii). Dissection of TFBSs orientation within the 218 core IR-HCTFBSs primarily retrieved “Head-to-Tail” yet rarely “Head-to-Head” syntax (Fig. 8Di), an architecture that reminisces the anatomical pattern of *in cis* expansion of TRs as repetitive units of the same orientation that can deliver TFBSs across the genomes [37, 118, 119]. This distinguished genomic syntax encrypts a delegated TF–DNA interrelationship since IRF3-ChIP-seq and IRF3-ChIP-STARR-seq evaluations (HL; SVI, 6 h) showed that IR-HCTFBSs exhibit stronger TF-binding and STARR-transcripts upregulation, respectively, compared to non-IR-HCTFBS-SHAe (Fig. 8Ei). This cohort of IR-HCTFBSs includes dozens of previously unknown and many well-characterized vrCRMs (Fig. 8F and Supplementary Table S5). GOs uncovered robust specificity for antiviral cellular processes (Fig. 8Dii), and genomic localization assessments mapped 84 proximal vruDEGs, including key antiviral players, e.g. *IFITs* and *IFNβ*, and highlighted remarkable distribution across SEs, tEs, and “safGs” (Fig. 8Ei, F, and Supplementary Table S5). Importantly, we also monitored the assembly of IR-HCTFBSs across the coordinates obtained from HeLa, in lung A549 cells and B-lymphocytes NM, and identified ∼240 and ∼190 common instances, respectively, that are bound by IRF3 6h upon virus-infection according to our IRF3-ChIP-seq assays.

We followed an enriched workflow and identified 588 NF-κB-repetitive-HCTFBSs (κBR-HCTFBSs) based on perfect, degenerate, and “half” NF-κB sites (Supplementary Text S7; Fig. 8B; Supplementary Fig. S28A and B, and Supplementary Table S5) [34, 36], that in contrast to IR-HCTFBSs, they are nearly balanced between the repetitive (∼58.3%), predominantly reside in DRs- and non-repetitive (∼41.6%) p65-SHAe spectrums (Fig. 8Ciii). Therefore, repetitive-κBR-HCTFBSs are “newly acquired” compared to nonrepetitive, but this evolutionary distance is shorter compared to the one between IR-HCTFBSs classes (Fig. 8Cii,iv). The core κBR-HCTFBSs predominantly exhibit “Head-to-Tail” rather than “Head-to-Head” orientation (Fig. 8Diii). The cohort of 588 κBR-HCTFBSs is robustly related to antiviral defense cellular processes (GOs), and mechanistically, more intensively targeted by p65, and more efficient in delivering virus-inducible upregulation of STARR-transcripts *in vivo*, compared to nonrepetitive-p65-SHAe (Fig. 8Div and Eii). Genomic localization assessments mapped 70 vruDEGs associated *in cis* with κBR-HCTFBSs, corresponding to novel and well-characterized p65 targets (*IFIH1* and *OASL*) (Supplementary Table S5). The latter include also *NFKB2*, *CSF1*, and *HES1* loci mentioned above, that have been described to host clusters of targeted-NF-κB-binding sites, in TNF-a induced HCT116 cells, a result that further underscores our findings [98]. Moreover, multitudes of κBR-HCTFBSs are embedded within SEs, tEs, and “safGs” (Fig. 8Eii,F and Supplementary Table S5). Lastly, IR//κBR-HCTFBSs were also defined and inhabit both genomic loci of known antiviral/immune-response genes (e.g. *IFIH1*and*ISG15*) and novel IRF3- and NF-κB-targets (Fig. 8F; Supplementary Fig. S28B and Supplementary Table S5). Intriguingly, IR-, κBR-, and IR//κBR-HCTFBSs do not exhibit statistically significant evolutionary distances (Supplementary Fig. S28A). Hence, here we deliver the *in vivo* reconstitution of novel functional SHAe-HCTFBSs, of distinguished DNA Grammar and Syntax, stimulus specificity, and transcriptional fitness. These are targeted by antimicrobial TFs, are constituents of tEs, SEs, and “safGs,” and work as vrCRMs, *in vivo*.

Then, we traced the evolutionary footprint of IR-HCTFBSs by scanning the genomes of ∼100 species that evolved over billions of years, ranging from human relatives to microbia, e.g. *Viruses* (Fig. 9A). HOMER2 tool applied on the 482 IR-HCTFBSs computed *de novo* an *in silico* bait termed clustered-IRF3-motif (“cIR-motif”) that is made up of 25 bp, it harbors two perfect IRF3 TFBSs, and it is incredibly sensitive/specific in probing IRF3-TFBMs “clustered” organization (Fig. 9A). Importantly, the same workflow failed to generate an *in silico* bait motif for κBR-HCTFBSs, and thus, they were excluded from further analysis. PWMScan [44, 120] traced ∼1.8 × 10^6^ “cIR-motif” instances across the human genome (hg19), which equals ∼1.45% of its entire length; an extended portion, when considering that its protein-coding segment is ∼2%. Importantly, these loci markedly recover the IR-HCTFBSs (∼72%, *n* = 347), the IRF3-SHAe (∼32.88%, *n* = 640), and the IRF3-ChIP-peaks (SVI, 6 h) in HL (∼20.5%, *n* = 1972), NM (∼22.5%, *n* = 878), and A549 cells (∼40%, *n* = 393) (Supplementary Fig. S28C). In principle, complex multicellular eukaryotes such as nonhuman primates, mammals, plants etc., follow the *H. sapiens* “mode-of-enrichment” (number of instances and % of genome coverage). Many nonmammalian metazoans, such as the fruit fly *Drosophila melanogaster*, Gallus gallus, *Caenorhabditis elegans*, Zebrafish, etc., exhibit medium or low enrichment in both parameters, but this is notably lower compared to other multicellular organisms (e.g. Octopus vulgaris and Apis mellifera). Intriguingly, rather than many of their evolutionary relatives, or prokaryotes like bacteria and archaea, human eukaryotic parasites’ genomes, such as those of *Plasmodium falciparum*, *Entamoeba histolytica* etc., exhibit analogous increased modes with those of the *H. sapiens* and nonhuman primates’ genomes. Regarding *Viruses*, their small genomes limit the likelihood for *in vivo* assembly of expanded clustered TFBSs, but even so, human herpesviruses, renowned for infecting germlines and endogenizing/engulfing parts of or their entire genomes into the host genome [121, 122] carry multiple “cIR-motif” instances. Hence, in principle vertical evolutionary hierarchies mark the inheritance of IRF3-TFBSs clustered organization from ancestors into the human genome, yet these may have been bypassed in episodes of horizontal transmission, e.g. when the donor organism is a human (or mammalian) parasite, capable of infecting the germline. This is made more puzzling when considering the virtually infinite ways by which molecular phenomena connected to DNA replication, stability, and innovation in collaboration with the evolutionary forces can edit the genomes throughout the MY of evolution.

Moreover, we discovered that IRF3 clustered TFBSs can further segregate and constitute prolonged *cis*-elements. We applied “Cluster Intervals” function (≥3 “cIR-motif” instances interspaced by <75 bp are assigned as constituents of a unique/given *cis*-element) and mapped ∼30 000 and ∼110 000 IR-HCTFBSs-Aggregated-Domains (IRHADs) across the human (hg19) and the mouse genome (mm9), respectively. We biochemically validated these findings *in vivo*, by assessing our IRF3-ChIP-seq peaks (SVI, 6 h) in HL, NM, A549, and NIH/3T3 cells and gained substantial recoveries. Notably, a wealth of human IRHADs overlap with SHAe. Hence, in both mammalian species, such condensed TF-TFBSs are assembled in virus-infected cells (Supplementary Text S8, Supplementary Fig. S28D, and Supplementary Table S5). Collectively, the homotypic mode-of-clustering of IRF3-TFBSs is pervasively fingerprinted within the genomes of recent, old, and ancient species, and can give rise to functional *cis*-elements of intricate DNA Grammar and Syntax, throughout evolution. Given the plethora of cell types that compose the mammalian bodies [123], such *in cis* aggregated domains can be alternatively utilized/targeted by the plethora of TFs and regulate diverse genes in distinct tissues.

Last, we emphasized the innovation of human genome architecture and function that arose via new SHAe acquisitions. We centered to 102 HL vruDEGs that share orthologous genes in mice, and in both species are IRF3-targets (NIH/3T3 IRF3-ChIP-seq; SVI, 6 h; HL IRF3-ChIP-seq; SVI, 6 h) (Fig. 9B), while several of those are “key players” of antiviral response e.g. *Ccl5*, *Isg15*, *Ifnβ*, *Ifit_s_*, and captured upregulated in our NIH/3T3 RNA-seq assays (SVI, 0 and 7 h) (Supplementary Table S1). We identified that 37 IRF3-bound mouse IR-HCTFBSs (mIR-HCTFBSs) are associated *in cis* with these loci (Supplementary Table S5). Comparative investigations uncovered intriguing episodes related to new SHAe acquisitions. For example, (i) IR-HCTFBSs display either similar or distinct patterns of residence relative to the TSSs of orthologous mouse and human genes: Two IR-HCTFBSs, a TSS-proximal (+237) and a TSS-distal (-9687) are topologically associated with the human *CCL5* gene (Fig. 9Ci). The orthologous mouse locus bears one mIR-HCTFBSs proximal to TSS (-68), thus reminiscing the human architecture, and one distal (-4341), but their intervening sequence spans half of the length compared to the respective one across the human *CCL5* locus. Sequence alignment and Repeatmasker analyses identified that the segment of the human intervening sequence does not correspond to the mouse genome, is nearly absent from rat and golden hamster genomes, but is traced in higher Primates and in other mammals (phylop100way, 241 placental mammals) [108]. It harbors multiple insertions of TEs, including SINEs (Alu, MIR) and ERVs (LTR35A, LTR8). Intriguingly, this human sequence “endogenizes/engulfs” an additional IRF3-SHAe that belongs to IR-HCTFBSs, within the modular architecture of *CCL5* viSE, described above. This acquisition expands the repertoire of functional vrCRMs of the human *CCL5* locus. Towards, this genome innovation episode, grants human cells an additional vrCRM, might facilitate the “birth” of a SE upon virus infection and might influence the fine-tuning of a human antiviral gene, thus enforcing regulatory underpinnings on this locus; (ii) SHAe can encode for new amino acids; Human *CHEK2* harbors an IR-HCTFBS structured by TR and Alu elements, that is located ∼11.3 kb downstream of its TSS (Fig. 9Cii; Supplementary Fig. S27B; Supplementary Table S5), inhabits the viSE of the gene, coincides with its third exon, and exhibits substantial conservation with viral genomes. This IR-HCTFBS, the human exon, and the viSE are classified in e-primates and they are absent from the mouse genome (phylop100way). This genome innovation episode presumably leads to the generation of a vrCRM, the “birth” of a viSE, and the addition of amino acids in an isoform of the human protein (exonization) [124]. Importantly, it has been shown that immune pathways adaptation is connected to the evolution of cCREs and exons [108]. In line, intersection of SHAe with G3 exons (primate-specific) uncovered several common instances within vruDEGs (e.g. *IFIT3*, *IFITM3*, and *APOL2*) and beyond (e.g. *ACVR1*, *RAN*, and *ACSF3*) (Supplementary Fig. S27B); (iii) SHAe can encompass new targeted-IRF3- and NFκB-TFBSs as shown by comparative analyses of the human and mouse *IFIH1/Ifih1* TSSs-proximal genomic loci, SHAe and ChIP-seq investigations (Supplementary Text S9 and Supplementary Fig. S28E). Together, the above findings imply that antiviral cellular response was evolutionarily supervised, and it is connected to the (re)shaping of the virus-responsive fates of the mammalian (epi)genomes.

### Virus-responsive transposable elements transcription, a new era in stress-induced/defensive gene expression

Our results show that SHAe are enriched in ancient DNA segments, such as TEs, which are known to become transcribed in human cells upon infection by diverse *Viruses*, like SV [125, 126]. However *in vivo* evidence regarding TEs transcriptional regulation is elusive. We processed our RNA-seq datasets by TEtranscripts tool and identified multiple families of vru-differentially-transcribed-TEs (vruDTTEs) in NM (*n* = 58) and HL (*n* = 60), such as MER107, LTR26, and MER57B1 (Fig. 9Di; Supplementary Fig. S29A and B). Next, by increasing the resolution of the analyses, we monitored each TE copy and captured 2284 HL and 3991 NM vruDTTEs (e.g. ERVs, Alu, MIR, and LINE L1). Those vruDTTEs are largely shared in NM and HL, are associated *in cis* (neighboring, proximal, and overlapping) to vruDEGs, and exhibit remarkable specificity for antiviral defense cellular processes (Supplementary Fig. S29C). Intriguingly, the binomial test verified statistically significant enrichment in proximity between vruDTTEs and vruDEGs, compared to an equal number of nonvirus-upregulated TEs, in both HL and NM (Fig. 9Dii and Supplementary Fig. S29E). This profile was maintained even when intergenic vruDTTEs were assessed in isolation, depicting statistically significant enrichment within 10–20 kb distal from vruDEGs. These findings imply that regulatory mechanisms of antiviral transcription are applied both within and outside of the genomic coordinates that host vruDEGs and supervise the assembly of an alternative defensive transcriptome—apart from the one that encodes protein-coding mRNAs—that is encoded from vruDTTEs (Supplementary Text S10, Supplementary Fig. S29B–D, and Supplementary Table S5).

To investigate the regulatory underpinnings of TEs-transcriptome assembly, we first examined vruDTTEs’ inhabitance within tEs, SEs, “safGs,” SHAe, and cCREs and captured remarkable coincidences (Supplementary Fig. S29F and G). In HL, 178 vruDTTEs (SVI, 6 h) are embedded within 68 unique SEs (22 in pmSEs and 156 in viSEs), and 24 of those are endogenous Retroviruses (ERVs), remnants of ancient Retroviruses (within 17 unique SEs). 203 vruDTTEs reside within tEs, and 1903 in “safGs.” Analogous results were retrieved in NM (Supplementary Fig. S29F and Supplementary Table S5). Many of those vruDTTEs reside within viSEs associated with key antiviral genes (*CCL5*, *IRF1, ISG15*, *ISG20*, *IL7R* etc.), and are transcribed within their architectures (IGVs) (Supplementary Fig. S29G). Thus, the function of SEs in the virus-stimulated TEs-transcriptome assembly is illuminated in line with published results showing that ERVs become transcriptionally active *in vivo* by hijacking transcriptional condensates from SE-driven pluripotency genes [127]. Moreover, vruDTTEs reside more proximal to SHAe compared to the total STARR-Active elements (Dunn’s test; 0.1 cutoff in relative distance) (Fig. 9Diii), and they exhibit remarkable inhabitance within ±40 kb from their center (Supplementary Text S11, Fig. 9Diii–v, Supplementary Fig. S29G, and Supplementary Table S5). Finally, 422 vruDTTEs were uniquely- and 9 multiply-mapped among the G1, G2, and G3 categories of cCREs [108], and this reciprocally provides additional functional evaluation for their *in vivo* operation.

Moreover, in MRC-5 cells, we recorded substantial overlaps of vruDTTEs with NM and HL, remarkable correlations to defensive/immune responses, and predominant inhabitance within introns (Supplementary Fig. S30A–D and Supplementary Table S5). Crucially, we also delineated the mouse vruDTTEs-encoded transcriptome in NIH/3T3 cells (SVI, 0 and 7 h), which, similar to those encoded in human cells, exhibits statistically significant specificity for defensive/immune responses (Supplementary Fig. S30E and Supplementary Table S5). Accordingly, it is reasonable to argue for a conserved role of TEs-encoded transcriptomes in shaping mammalian cellular responses against pathogenic stimuli. Furthermore, activation, maintenance, or acceleration of defensive gene expression can be induced by non-microbial stimuli, e.g. autocrine or paracrine signals and chemotherapies. In line with our results, in IFNγ-induced HL cells, ERVs host STAT-binding and H3K27ac histone labeling and work as enhancers of innate immunity [31]. Moreover, TEs-transcripts commit to immune-response in mouse hematopoietic stem cells under the stressful conditions of chemotherapy through the cytoplasmic IFIH1, and the IRF3 and NF-κB pathways activation [128]. Hence, distinct modalities of vruDTTEs are programmed, *in vivo*: (i) they work as vrCRMs regulated by IRF3, and/or p65, and encode transcriptional regulatory output. (ii) They become (self)-transcribed and encode TEs-RNAs. Thus, the spectrum of vruDTTEs is an integral part of the virus-responsive fate of the human (epi)genome, and its encoded TEs-transcriptome is a derivative of the antiviral transcriptional response of human cells.

### The “Human hyper-Atlas of virus-infection”, an integrative “molecular *in silico*” encyclopedia

We capitalized on this *voluminous* knowledge and charted in high resolution the functional and mechanistic insights discovered upon multi-layered integration/overlaying of the topographic maps, which were made from the analyses of hundreds of millions of sequencing reads of nucleic acids obtained from our *multi*-omics, functional, genome-wide study. The “Atlas” among others hardwires: (i) the epigenomic topographies and features of the discovered virus-responsive *cis-*acting elements and chromatin microenvironments, (ii) the transcriptional regulators’ distribution and the RNA pol II “travelling,” and (iii) the genomic residence and the evolutionary history of vrCRMs, vruDEGs, vruDTTEs, and SNPs. The first version (VoI 1) of the Atlas (Supplementary Fig. S33) is annotated in the UCSC genome browser (“Human hyper-Atlas of Virus-infection”; https://genome.ucsc.edu/s/magelo%40BRFAA/The%20Human%20hyper-Atlas%20of%20virus-infection), and it is complemented by data stored in our GitHub Repository.

A main scope of the Atlas is the elucidation of associations between functional *cis*-acting elements and human diseases. More than 4300 unique autoimmune-associated SNPs [49, 129] were assessed regarding their residence within or vastly proximal (±2.5 kb) to the functional SEs and SHAe discovered. This stringent criterion for *in cis* proximity increases the likelihood of mapping biologically meaningful rather than random coincidences. Within SEs, we mapped ∼300 and ∼380 SNPs in naïve and infected NM, while ∼80 and ∼150 in HL, respectively. The percentage of the SNPs recovered within SEs’ genomic coordinates is remarkably enriched, given that SEs inhabit a limited proportion of the total genome length [two-tailed Fisher’s exact test; HL pmSEs (*P-*value = 4.59e^−14^), HL viSEs (*P-*value = 1.97e^−34^), NM pmSEs (*P*-value = 6.08e^−130^), and NM viSEs (*P*-value =3.67e^−33^)]. Several SEs-encompassed-SNPs are linked to autoimmune diseases such as MS, CD, RA, SLE etc., and exhibit immoderate specificity for defensive/immune processes in NM prior to and upon virus infection and in HL cells only upon virus-infection (Supplementary Table S5). Interestingly, we increased GOs’ resolution, and revealed that in NM both pm- and vi-SNPs-linked-SEs are correlated with defensive/immune functions, whereas in HL only vi-SNPs-linked-SEs exhibit such specificity; in line with the unique identity, physiology, functional traits, and the distinguished commission of B-lymphocytes and epithelial cells in organisms’ homeostasis and autoimmune phenotypes development.

**Figure 10.**
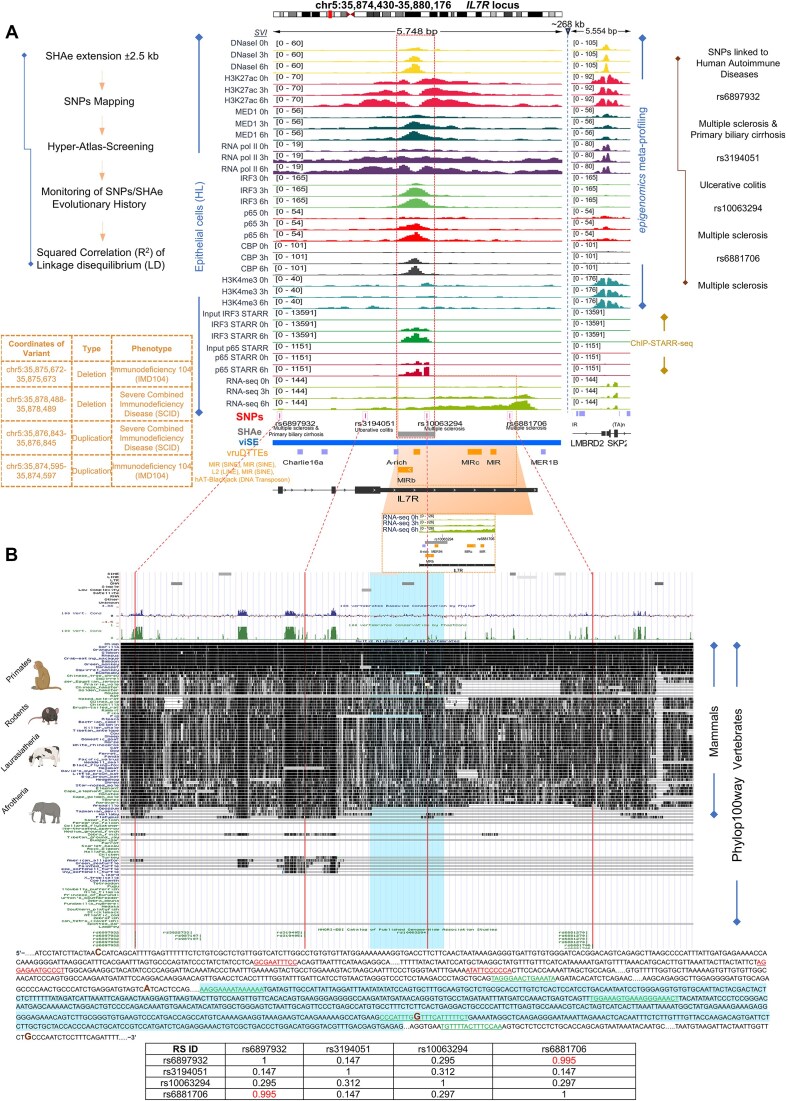
Integrative analyses of SHAe loci that are enriched in SNPs and other short variants linked to human (auto)immune diseases. (**A**) Upper left panel: A description of the workflow. Upper center panel: The transcriptional and epigenomics states of the IRF3/p65-SHAe and its flanking ±2.5 kb sequence. These genomic coordinates are encompassed within the *IL7R* genomic locus and are constituents of the viSE of the gene (horizontal line). A CH is assembled prior to (SVI, 0 h) and becomes enhanced upon virus-infection (SVI 3 and 6 h) and is accompanied by the binding of the antimicrobial TFs (IRF3 and p65) and the recruitment of CBP, thus leading to the *in vivo* reconstitution of functional vrCRMs. RNA pol II travels across the gene body and transcribes its coding region, which apart from protein-coding sequences harbors multiple vruDTTEs (orange lines) that generate TEs-encoded RNAs (lower orange rectangle). Several autoimmune-disease-associated SNPs are mapped within the above genomic coordinates, and the majority of those are linked to MS. Interestingly, rs10063294 inhabits the IRF3/p65-SHAe sequence within an IRF3-binding site, as shown in (B) (sequence panel). Upper right panel: A catalog of the SNPs and their associated Human autoimmune diseases. Middle left panel: A chart of striking examples of characterized deletions and duplications that reside within the *IL7R* locus, accompanied by their associated phenotypes’ descriptions. The entire analysis is charted in GitHub Repository. (**B**) Upper panel: The evolutionary origins of the SHAe and its *in cis* associated SNPs within the cohort of 100 representative vertebrate species (phylop100way); In principle, the chr5:35874430–35880176 locus of the human genome is not traced beyond mammalian species, and its sequence conservation is significantly increased from lower mammals such as Aardvark, to primates. Middle panel: The sequence architecture of the locus [TFBSs for IRF1/3 (green letters) and NF-κB (red letters); IRF3/p65-SHAe (blue shadow); SNPs (brown letters)]. Lower panel: Linkage disequilibrium (LD) analyses performed by the calculation of the squared correlation (*r*^2^) between SNPs. A pair-wised approach was conducted for the total of SNPs and the results highlighted significant correlations with the rs6881706–rs6897932 pair that is classified at the top of the ranking, reaching an absolute correlation. Both SNPs are associated with MS. Part of Fig. 10 was created in BioRender: Agelopoulos, M. (2025) https://BioRender.com/b54k071.

Next, we mapped 56 SNPs within or adjacent to 40 SHAe (Fig. 10; Supplementary Figs S31 and S32; GitHub Repository; Supplementary Table S5). Linkage disequilibrium (LD) analyses were carried out when multiple SNPs reside within or close to a unique SHAe by the calculation of the squared correlation (*r*^2^) [50] between the pairs of variants of interest; the results depicted robust associations that in many cases reach the absolute *r*^2^= 1 (Fig. 10; Supplementary Figs S31 and S32). In this regard, multiple SNPs that are lined up across the extended coordinates screened may inhabit/attend the same functional *cis*-acting element, like a SEs or a SHAe. Having constructed the “Atlas”, we gained elegant/in-depth access to the transcriptional and (epi)genomic states, the evolutionary history, and the regulatory principles of those SEs- and SHAe-SNPs-linked loci. A striking example derives from the *IL7R* locus where several known SNPs linked predominantly to MS reside proximal or within the SHAe, that inhabits the viSE of the gene (Fig. 10). This vrCRM hosts a CH, encompasses a vruDTTE, and harbors multiple NF-κB and IRF3/1 TFBSs, one of which engulfs the rs10063294 SNP. This SHAe and its flanking sequences are primarily conserved in mammals and meet their higher DNA sequence conservation in primates. Additional striking examples include SHAe and SNPs that neighbor, reside proximal, or entirely coincide with known immune-related vruDEGs such as *IRF7* (LD analysis: *r*^2^= 1 in several pairs of SNPs, Supplementary Fig. S31), *DDX58*, *IFITM3*, *JAK2* etc. (Supplementary Figs S31 and S32; GitHub Repository; Supplementary Table S5). Another striking example is derived from the *IFITM3* which encodes for an antiviral protein that inhibits viral infection of the host [130] and its TSS-proximal sequences encompass rs35218683 that is linked to MS, resides adjacent to the SHAe that lies within/vastly proximal to the pmSE of the gene. This locus (5′ prime UTR) also hosts rs34481144 that is linked to the severity of Influenza infection [130]. This SHAe exhibits its higher conservation in mammals and primates, and is nearly absent from other vertebrates. Both our results and published studies, show that the SHAe hosts IRF3-binding which in the case of rs34481144 sequence alteration decreases and causes substantial downstream effects on the expression of *IFITM3* and its neighboring genes. Interestingly, LD analysis computed *r*^2^= 0.612 for those two variants. Hence, our holistic workflow resolved the transcriptional and (epi)genomic states of autoimmune-disease-associated functional chromatin microenvironments and the evolutionary history of their encompassed SHAe and SNPs, and illuminated critical mechanistic insights (Supplementary Text S12; Fig. 10; Supplementary Figs S31 and S32).

In addition, the recent advanced study of Aracena *et al.* [129] illuminated that epigenetic variation influences the divergence of transcriptional response upon influenza A infection in macrophages and provided a plethora of genomic loci that are marked by epigenomic features/variations, QTLs etc. We defined that >1000 SHAe overlap with these genomic loci, a result that confirms their functional conductance in human antiviral cellular response. QTLs were mapped to correspond to SHAe endogenous loci associated with human autoimmune diseases, such as the one proximal to the *SVIL* locus that is linked to CD and inflammatory bowel disease (IBD) (GitHub Repository, Supplementary Table S5). At the mechanistic level, our ChIP-seq data highlights remarkable epigenetic (co)-regulation of these SHAe/QTLs loci by antimicrobial TFs (IRF3 and NF-κB) and coactivators (MED1 and CBP), as illustrated by the upset plot in Supplementary Fig. S30F. Notably, ∼100 of those SHAe/QTL reside within SEs, in virus-infected HeLa cells. GOs of the SHAe/QTLs highlighted statistically significant correlations with defensive cellular functions such as response to virus and type I IFN signaling pathway (Supplementary Table S5).

Finally, the comparative intersection of the SHAe, cCREs, and QTLs cohorts revealed >400 virus-responsive *cis*-elements that are functional *in vivo* (SHAe/cCREs) and exhibit remarkable specificity for defensive cellular processes (Supplementary Table S5). Those include among others the *CCL5* (+237) TSS-proximal element, the *OASL* (-20 kb) distal element etc. (Supplementary Table S5).

Finally, we screened the ClinVar database and mapped >1450 short variants (SNPs, deletions, duplications etc.) within the extended coordinates of the SHAe, a significant portion when considering that the vast majority of those are linked to at least one (auto)immune phenotype. These DNA sequence alterations were merged with those derived from our investigations described above (56 SNPs within 40 SHAe) and identified a spectrum of 55 SHAe to inhabit >1509 SNPs and other short variants. The Disease Enrichment Analysis on this SHAe cohort highlights outstanding specificity for (auto)immune-related diseases, such as primary immunodeficiency disease, asthma, viral infectious disease etc. Most importantly dozens of the vruDEGs identified (*DDX58*, *NFKB2*, and*IL7R*) to associate with these diseases are also associated *in cis* with those 55 SHAe (Supplementary Fig. S30G, GitHub Repository), thus illuminating the commitment of these vrCRMs in cellular physiology. Together, these multi-layered functional evaluations prove the commission of the discovered SHAe in the mechanisms of human physiology that becomes dysregulated in cases of (epi)genomic variations/abnormalities and/or microbial attacks. Hence, we evaluated the DNA sequence/(epi)genome alterations-instructed molecular mechanisms of human autoimmune diseases development under the lens of vrCRMs *in vivo* operation and dysregulation. Such discoveries delineate the assembly and function of the virus-responsive fate of the human (epi)genome and underline the utility of the “Human hyper-Atlas of Virus-infection” as a valuable resource/repository and its applicability to additional systems where mammalian antimicrobial response is investigated.

## Discussion

Herein, we investigated from the ground state a vital yet unresolved mechanism of human homeostasis: the capability of cells to execute defensive transcriptional responses when challenged by *Viruses*. First, we showed that human cells shape heterogeneous transcriptional activation patterns of vruDEGs (C/I-III), and this mechanistically relies on robust epigenomic supervision. CHs are reconstituted *in vivo* and the majority of those are endowed with cCREs according to ENCODE [14]. For example, the “very sensitive” TSSs-proximal DNA stretches of vruDEGs are architecturally stated within virus-responsive chromatin microenvironments of residence conveyed either by epigenetic memory and/or assembled from the ground state in infected cells. Based on our data, diverse patterns of CHs formation, (co)-occupation of transcriptional regulators (MED1 and CBP) and antimicrobial TFs (IRF3 and NF-κB), and nucleosome modifications (H3K27ac and H3K4me3) are shaped *in vivo* in compatibility to the divergence in transcriptional responsiveness/stimulation that characterize the identified distinct clusters of vruDEGs. Strikingly, CHs *in vivo* reconstitution characterizes both naïve and antiviral states and is dominant across TSSs vicinities, thus also indicating a broader role of these chromatin microenvironments in gene expression regulation under naïve cellular states, in the absence of infections. Hence, the transcriptional regulation of human antiviral response relies on virus-responsive chromatin microenvironments and they are acquired both by epigenetic memory and *de novo* assembly. Apparently, the 3D shape of human chromatin facilitates DNA–DNA communication [88, 131] between distal *cis*-elements and this can deliver on TSSs transcriptional regulatory output that originated far distant from the gene(s) that utilizes it. Hence, embedded, proximal or distal vrCRMs, even those located in other chromosomes, in collaboration with the corresponding TSSs-proximal chromatin architectures, can accurately convert the manufactured transcriptional regulatory output to fine-tuned antiviral gene expression programs, under precise epigenomic supervision.

Next, we showed that extensive epigenome reprogramming hallmarks the transition from naïve to antiviral human cell states. Expanded entities that harbor previously unknown virus-responsive tEs and SEs were found circuited in the mechanisms of antiviral transcriptional control. Importantly, the patterns of assembly of SEs match vruDEGs’ expression profiles as those become steady-stated under homeostasis and modified under the emergence of the microbial challenge. Crucially, many human vruDEGs reside close to or “live” underneath SEs as “permanent residents” within pmSEs, or as cell-state “exchanged visitors” within viSEs. These vruDEGs are, by default, privileged in utilizing the transcriptional regulatory output that SEs manufacture. These findings further strengthen/extend the critical role of *cis*-acting elements surfacing in stimulus-induced defensive gene expression, as described in alternative systems e.g. upon LPS stimulation [123]. This type of (epi)genomic response indicates a functional conductance of expanded chromosomal domains rather than restricted genomic coordinates in regulating human antiviral/defensive processes. Classification of vruDEGs based on SEs proximity recapitulate their actual *in vivo* transcriptional profiles. Notably, SEs were previously unknown in this arena of virus-responsive transcriptional regulation of human cells. These novel SEs integrate IRF3, p65, MED1, CBP, and RNA pol II inputs, in line with the known commission of antimicrobial TFs [80] and chromatin complexes in epigenome reprogramming. Moreover, the majority of the SEs discovered (∼70%–90%) host CHs and this further underscores their functional principles (Supplementary Table S5). The above mechanistic and structural insights illuminate the capability of human cells to architecturally and functionally compartmentalize their (epi)genome on a finer scale to achieve the desired outcome, which, in cases of microbial attacks is the accurate shaping of defensive gene expression programs.

The TFs-ChIP-STARR-seq assays in human epithelial cells allowed us to evaluate these cellular features at the mechanistic/functional level in spite of the limitations that govern massive-in-parallel reporter examinations [24]. These functional assays for IRF3 and NF-κB were missing from the field and forced our mechanistic and functional understanding several steps ahead. Thousands of previously unknown IRF3- and/or NF-κB-bound SHAe were mapped within or outside vruDEGs, tEs, SEs, and rDEs and are hallmarked by coactivators, RNA pol II, epigenetic features of transcriptional activation, and substantial specificity for virus-stimulated defensive cellular responses, thus working as vrCRMs, *in vivo*. A survey of examinations including epigenomic profiling, genomic localization assessments, GOs, TFBSs analyses, binomial-test-based statistical evaluations of *in cis* proximity, and IGVs confirmed that SHAe exhibit: (i) extensive/extraordinary residence/inhabitance within SEs, tEs, rDEs and “safGs”, (ii) substantial correlation with antiviral/defensive cellular processes, (iii) enhanced legibility by antimicrobial TFs, (iv) excessive histone labeling and antimicrobial TFs, coactivators and RNA pol II recruitment, and (v) outstanding *in cis* associations/proximity with vruDEGs across the human genome. Definitely, we do not state that IRF3-, p65-, and IRF3/p65-SHAe are the only functional vrCRMs of the human genome, but a descent bona fide spectrum of those. The initial observation that ∼50% of SHAe are orthologous in the mouse genome was considered together with the published notion that IRF and NF-κB families may co-evolved during the transition from uni- to multi-cellular organisms [99]. DNA evolution investigations illuminated that the repetitive-SHAe are “evolutionarily younger” compared to the non-repetitive that display prolonged evolutionary history. This may imply the operation of evolutionary forces and mechanisms of natural selection acting on the shaping of the virus-responsive fate of the human (epi)genome. The recent publication of Andrews *et al.* enabled us (i) to validate the evolutionary hierarchies of the SHAe, (ii) to provide proofs of *in vivo* function for over 1000 SHAe/cCREs in human virus-infected epithelial cells, and (iii) to identify that a series of the SHAe overlaps with primate-specific exons, including a set that encodes for antiviral regulators, in human virus-infected epithelial cells. These findings underscore the regulatory role of SHAe in the evolution of defensive/immune mechanisms against pathogens both at the functional (epi)genomic and protein levels. They also expand the observation that primates evolved these mechanisms to adapt on a finer scale [108] and highlight that in humans this involves the function of the discovered SHAe.

Among the cohort of SHAe, a wealth of HCTFBSs bound by IRF3 and/or p65 was discovered. The delegated DNA Grammar and Syntax of these clustered vrCRMs can compensate for their functional fitness [132], might limit “leaky or noisy” transcription, and can facilitate signal integration and transcriptional response. Notably, in human pancreatic ductal adenocarcinoma cells (PDAC), the TF ZEB1 binds clustered elements predominantly nearby chromosomal telomeres. ZEB1 clustered elements operate as transcriptional silencers critical for the maintenance of cellular identity [37]. Alternatively, IR-, κBR-, and IR//κBR-HCTFBSs vrCRMs are broadly distributed across telomeres, intra-chromosomal domains, and centromeres (Fig. 8E and F), and work as transcriptional enhancers. Moreover, IR-HCTFBSs fingerprint is pervasive throughout evolution, and marks genomes ranging from the animal to microbial kingdoms, including human infectious parasites and *Viruses*. The *in silico* identification and *in vivo* biochemical validation of IRHADs in human and mouse cells sharply underscore the above findings. Having probed mammalian chromatin from distinct organisms and cell types (epithelia, blood, lung, and fibroblasts) against IRF3 in ChIP-seq assays we gained precise insights in IRHADs *in vivo* reconstitution, across mammals. Accordingly, *cis*-elements of “clustered” organization, divergent phylogeny, and diverse genomic localization are recognized by miscellaneous TFs (e.g. IRFs and ZEB1) and regulate vital cellular processes. In light of these results, it becomes further understood that the human “Repeatome”: (i) is supervised by epigenome-instructed, sequence-defined and stimulus-applied rules, and (ii) is an integral part of the mechanisms of transcriptional control; thus, should no longer be “stereotypically” assigned as concatemerized DNA segments of unknown function [133]. This is also supported by the comparisons of our datasets with those of Aracena *et al.* [129] that demarcated a wealth of SHAe/QTLs that are composed of STRs and work as virus-responsive enhancers.

Furthermore, we mapped Mobile elements (vruDTTEs) to inhabit the virus-responsive fate of the human (epi)genome and upon virus-infection to encode an alternative transcriptome concurrently to the one originated from protein-coding vruDEGs. These findings indicate that vruDTTEs: (i) (re)shape the antiviral transcriptional response, (ii) are functionally supervised by vrCRMs, whereas in many cases are composites of those, thus executing both transcriptional regulatory and self-transcribed capacities, (iii) exhibit a broad pattern of residence within tEs, SEs, rDEs, SHAe, and cCREs. Given that deleterious TEs’ insertions cannot be maintained within populations [134] due to the function of the mechanisms of natural selection; it becomes evident that the antiviral response of human cells is privileged by the functional conductance of TEs.

Moreover, we investigated SEs and vrCRMs/SHAe function under an autoimmune-disease-oriented angle. We conducted a high-throughput screening for SNPs by applying two stringent criteria: (i) the *in vivo* function of the *cis*-elements and (ii) their limited linear distance (±2.5 kb) from the SNPs examined. This rationale delivered enormous specificity and captured biologically meaningful correlations/associations. These results authenticated the functional conductance of the identified *cis*-elements under homeostasis and disease development. They also underscore the utility of the “Atlas” for basic and translational research.

Herein, we filled significant gaps of knowledge. We delved into the virus-responsive fate of the human (epi)genome and conducted several steps ahead in understanding its composition, architecture, function, evolutionary origins, and its significance for cellular homeostasis. We defined that it harbors a plethora of novel functional virus-responsive tEs, SEs, rDEs, “safGs,” and vruDTTEs. These grant human cells basal immunity and eradicate illogical-harmful defensive responses under homeostasis, yet stimulating antiviral gene expression programs upon virus infection. The polyphyletic evolutionary history of the vrCRMs, the conserved features of antimicrobial TFs and chromatin complexes that regulate their function, and their pluripotent stimulus-induced activation, when considered together with the genome innovation/acquisition episodes, exemplified above, the pervasive evolutionary footprints of clustered IRF3 TFBSs from human to pathogens, e.g. parasites and *Viruses*, and the pattern of residence of the vrCRMs within autoimmune-related SNPs/QTLs-hosted genomic loci allow us to envision that (i) the composites of the virus-responsive fate of the human (epi)genome might be functionally committed in cellular responses triggered by other stressful/infectious stimuli and integrated into alternative microbial-responsive (epi)genomic fates established, as suggested by our data (ii) apart from the vertical inheritance, horizontal transmission that can deliver DNA stretches or expanded genomic segments between unrelated species, or both, might have facilitated (as suggested by the discovery of HHPe-SHAe that carry segments conserved in viral genomes) the shaping of vrCRMs within the human DNA which was edited and subjected to natural selection throughout MY of evolution. These findings shed light on the perplexing natural conflicts and interactions between hosts and pathogens at the time of “symbiosis.” Definitely, such phenomena are amenably linked to defensive gene expression regulation, and, as shown, are efficiently programmed *in vivo* by thousands of novel human functional vrCRMs of recent, old, and ancient (microbial) origins/fingerprints, when crucial prerequisites related to stimulus-specificity, DNA Grammar and Syntax, genomic localization, and (epi)genome architecture are applied (Supplementary Fig. S33).

## Supplementary Material

gkaf207_Supplemental_Files

## Data Availability

All data are available in the main text or the Supplementary Materials. Raw and processed NGS data are deposited in the NCBI repository Gene Expression Omnibus (GEO), series GSE229445. The evolutionary history analysis of 3367 SHAe is deposited in our Github repository at https://github.com/magelo-lab/3367-unique-IRF3-and-p65-SHAe.git, and in Zenodo at https://doi.org/10.5281/zenodo.14917834. The “Human hyper-Atlas of Virus-infection” is deposited in UCSC Genome Browser at https://genome.ucsc.edu/s/magelo%40BRFAA/The%20Human%20hyper-Atlas%20of%20virus-infection. The unprocessed data generated in this study are available from the lead contact. This paper does not report original code. Any additional information needed to reanalyze the data reported in this paper is available from the lead contact; Marios Agelopoulos Ph.D; Corresponding author; email: magelo@bioacademy.gr
