# Supplementary material for: An evolutionarily conserved constellation of functional cis-elements programs the virus-responsive fate of the human (epi)genome
Source: Nucleic Acids Res. 2025 Mar 25;53(6):gkaf207. doi: 10.1093/nar/gkaf207 (PMC11934927; doi:10.1093/nar/gkaf207)

An evolutionarily conserved constellation of functional *cis*-elements programs the virus-responsive fate of the human (epi)genome

Marianna A. Koutsi[†,1], Marialena Pouliou[†,1], Dimitris Chatzopoulos[‡,1], Lydia Champezou[‡,1], Konstantinos Zagkas[1], Marili Vasilogianni[1], Alexandra G. Kouroukli[1], Marios Agelopoulos[*,1]

[1]Center of Basic Research, Biomedical Research Foundation, Academy of Athens, Athens, 11527, Greece

* To whom correspondence should be addressed: Marios Agelopoulos, PhD; Tel: +30 210 6597454; Fax: +30 210 6597545; Email: magelo@bioacademy.gr

† Join first authors

‡ Join second authors

"The authors wish it to be known that, in their opinion, the first authors should be regarded as joint First Authors".

Supplementary Text

Supplementary Text §1 Related to Figure 2; Supplementary Figures S4-S7; Supplementary Table S1

We next assessed publicly available bulk-RNA-seq datasets retrieved from studies utilizing multitudes of distinct human cells and *Viruses* (Figure 2; Supplementary Figure S4; Supplementary Table S1). Upon rigorous *transcriptomics* analyses, intersections of the datasets, and GOs validation we revealed sharp correlations with the expression profiles described in NM and HL. We centered our investigation on the 167 common vruDEGs of NM and HL and emerged broadly applied patterns of virus-stimulated gene expression programs assembly, in diverse types of human cells when infected by alternative *Viruses* (Figure 2; Supplementary Figure S4; Supplementary Table S1). In addition, a wealth of vruDEGs that encode for constitutive immunity molecules and are expressed in naïve NM and HL were found to establish enhanced basal levels of transcription prior to virus-infection in the rest of the cell types examined (Supplementary Figure S4A). These generic profiles of the evolution of defensive gene expression programs in distinct human cells are accompanied by cell-type-characteristic transcriptional responses markedly linked to diverse functions and pathways, as shown by Reactome analyses (Supplementary Table S1). We interrogated these findings at the single cell (sc) *transcriptomics* level. Publicly available scRNA-seq datasets were retrieved from studies in human Peripheral Blood Mononuclear Cells (PBMCs) [naïve or infected with SV (0h, 24h); naïve or infected with Influenza A virus (IAV 0h, 12 h)], and human ileum-derived organoids [naïve or infected with Human Astrovirus-1 (HAstV1 0h, 16h)] (Supplementary Figures S5, S6A; Supplementary Table S1). We followed the above workflow and applied rigorous *transcriptomics* analyses (supplementary materials and methods). We identified that diverse cell types of PBMCs execute similar transcriptional responses regarding dozens of marker genes of antiviral and defensive functions, when infected with the same *Virus* (SVI or IAV). Similar transcriptional responses were also evident for the same cell type of PBMCs in distinct infections (SVI vs IAV). Shared profiles of virus-stimulated transcriptional responses were also recapitulated in the ileum-derived organoids/HAstV1 system of investigation (Supplementary Figure S6A). We dissected more these phenomena in another mammalian species by assaying mouse NIH/3T3 fibroblasts in RNA-seq experiments (NIH/3T3; SVI; 0h, 7h)] (Supplementary Figure S7; Supplementary Table S1) and identified substantial overlap between human and mouse transcriptomes, assembled upon virus-infection, shared defensive and immune response pathways and functions (GOs). Finally, we examined analogous questions in publicly available RNA-seq datasets retrieved from human and mouse cell culture systems where, poly(I:C) was utilized for stimulation. *Transcriptomics* analyses in naïve and stimulated [poly(I:C); 0h, 6h] human dermal fibroblasts and mouse embryonic fibroblasts (MEFs) coupled with GOs, sharply confirmed similar correlations (Supplementary Figure S6B-D; Supplementary Table S1).

Supplementary Text §2 Related to Figure 1D, E; Supplementary Figures S8, S10, S12-S14; GitHub Figure 1; Supplementary Table S2

[1] QRTMs were conducted by computing the regional *epigenomics* signals and constructing aggregation plots and heatmaps. In many cases QRTMs are coupled with IGVs panels.

[2] The investigation of the virus-inducible *epigenomics* signals were done by genomic localization assessments, GOs, QRTMs, and TFBSs analyses.

Supplementary Text §3 Related to Figure 4B; Supplementary Figures S15A, S16B and S17C; Supplementary Table S3

GOs of SEs applied in cell-state contexts strengthened our findings when evaluating the full spectrum of SEs or subclasses that feature single or combined epigenetic marking by H3K27ac, MED1, IRF3, and p65 (Figure 4B; Supplementary Figures S16B and S17C; Supplementary Table S3). For instance, in naïve states, immune response, B cell activation, etc., in NM, and negative regulation of apoptosis and programmed cell death, etc., in HL, are highlighted. In antiviral states, immune responses, antiviral processes, and cytokine-mediate signaling in NM, and negative regulation of viral life cycle, immune system processes, response to stimuli etc., in HL, are among others distinguished (Supplementary Figure S15A).

Supplementary Text §4 Related to Figure 5Ai and ii

These ChIPed DNA elements were directly recombined to STARR-plasmids, without any intermediate selection step, and backtransfected in HL cells followed by mock or virus-infection (SVI 0h, 6h) (Figure 5Ai). Rigorous computational evaluations validated the efficiency of recombination of the TFs-ChIPed fragments in multiplexed STARR-plasmid libraries (Figure 5Aii), their capability for virus-inducible transcriptional upregulation of the 5'-GFP:*cis*-element:polyA-3' mRNA hybrids, and the recovery of functional elements.

Supplementary Text §5 Related to Supplementary Figure S24

An extended spectrum of the SHAe exhibit strong binding by IRF3 or/and p65 *in vivo*, yet fractions of mild or weak targets are also evident (Supplementary Figure S24). The latter can also account for *cis*-elements that assemble 3D contacts with strong-targets through DNA-DNA communication. Importantly, TFs, co-activators, RNA pol II, histone marks, and chromatin accessibility signals' intensities exert statistically significant correlations with those of SHAe-STARR-seq (Supplementary Figures S24). These alignments account for IRF3-, p65- and IRF3/p65-SHAe.

Supplementary Text §6 Related to Supplementary Figure S25D; Supplementary Table S5

We capitalized more on the cohort described in Andrews et al. that incorporates $0.92*10^6$ candidate *cis*-regulatory elements (cCREs) from hundreds of cells and tissue types, that were retrieved from ENCODE. Three groups of cCREs were distinguished as follows: G1; Highly conserved in mammals, G2; Actively evolving, with at least 90% sequence alignment to primates' genomes, and G3; primate-specific. We computationally assessed these datasets and intersected them with the genomic coordinates of the total 3,367 SHAe. We mapped 1,078 SHAe (unique) within G1 (n=723), G2 (n=186), and G3 spectrums (n=125). The remaining 44 were mapped in more than one group (Supplementary Figure S25D). We increased the resolution of analysis and showed that 71 G1-, 26

G2-, 13 G3-, and 8 G1,2,3-SHAe neighbor vruDEGs, approximately in one-to-one correlation (Supplementary Table S5).

Supplementary Text §7 Related to Figure 8B; Supplementary Figure S28B

The NFκB-Repetitive-HCTFBSs (κBR-HCTFBSs) were identified based on (a) perfect NFκB binding sites (n=20; core elements), (b) degenerate NFκB motifs (5'-GGGNNNNNCC-3') (n=146) and (c) NFκB "half" sites, 5'-GGRR-3' (n=422); spaced by variable inter-motif distances (Figure 8B; Supplementary Figure S28B).

Supplementary Text §8 Related to Figure 9B; Supplementary Figure S28D; Supplementary Table S5

We mapped more than 30,000 IR-HCTFBSs-Aggregated-Domains (IRHADs) across the human genome (hg19) and approximately 110,000 across the mouse genome (mm9), and then we biochemically validated these findings in both mammalian species. We assessed the IRF3-ChIP-seq peaks in HL (SVI 6h) and captured 224 targeted IRHADs (SVI 6h) of which 130 (58%) belong to IRF3-SHAe and reside proximal or distal to vruDEGs. Of the 110,000 mouse IRHADs, 664 are targeted by IRF3. In both species, IGVs resolved such condensed TF-TFBSs assemblies in virus-infected cells (Supplementary Figure S28D).

Supplementary Text §9 Related to Supplementary Figure S28E

The human *IFIH1* gene locus harbors an IR//κBR-HCTFBS across its TSS-proximal region that is conserved in the mouse genome (Supplementary Figure S28E). High-resolution comparative analyses of the sequence architecture in both species show that the human IR//κBR-HCTFBS is enriched in IRF3 and NFκB TFBSs that do not correspond to the mouse genome. Our data defined that these TFBSs are targeted by the antimicrobial TFs in HL cells upon virus-infection and presumably grant functional fitness to the IR//κBR-HCTFBS.

Supplementary Text §10 Related to Supplementary Figure S29A, C, D; Supplementary Table S5

We authenticated 2,284 vruDTTEs in HL and 3,991 in NM (mainly ERVs, Alu, MIR, LINE L1), while ~600 of those are common in both cell types (Supplementary Figure S29A, B, D). These vruDTTEs are associated *in cis* (neighboring, proximal, overlapping) to 148 and 172 vruDEGs, in HL and NM respectively (Supplementary Table S5), and predominantly reside within introns rather than distal intergenic and promoter regions (Supplementary Figure S29C). GOs highlighted substantial specificity for antiviral defense cellular processes (e.g., response to virus and to type I IFN) both for common and total vruDTTEs of each cell type (Supplementary Figure S29C, D). Importantly, this specificity was maintained even when apart from the exonic, the intronic, the 5' and 3' prime UTR, and the promoter vruDTTEs that inhabit vruDEGs loci -thus expected to become co-transcribed and to exhibit antiviral specificities- were excluded from the analyses. Thus, intergenic vruDTTEs show analogous defensive specificities.

Supplementary Text §11 Related to Figure 9Diii, Div, Dv; Supplementary Figure S29G; Supplementary Table S5

Genomic localization assessments defined that vruDTTEs inhabit genomic loci more proximal to SHAe compared to the total STARR-Active elements, as confirmed by Dunn's test (0.1 cutoff in relative distance) (Figure 9Diii). Next, we mapped vruDTTEs that are embedded within or located adjacent to SHAe (±~200 bp) (Supplementary Figure S29G), and are associated *in cis* with key vruDEGs (Supplementary Table S5). This was further underpinned *via* bi-directional mapping of vruDTTEs utilizing the center of each SHAe, as the reference stretch of the genome. The results recorded a gradual increase of vruDTTEs within 6 Mb distance and a strong linear association between SHAe and vruDTTEs within ~±300 kb. The latter is more aggressive within ±40 kb (Figure 9Div, Dv). Many core vruDEGs reside within these genomic coordinates (Supplementary Table S5).

Supplementary Text §12 Related to Figure 10; Supplementary Figures S31-S32; GitHub Figures 3-6; Supplementary Table S5

To achieve precision, from the total SNPs only those that reside within ±2.5 kb linear distance from the SHAe were processed in downstream evaluation. Linkage disequilibrium (LD) analyses were carried out in cases where multiple SNPs were mapped within or close to a unique SHAe. This was conducted by the calculation of the squared correlation (r^2) between the pairs of variants of interest, within European ancestry populations. The LDMatrix tool was applied (https://ldlink.nih.gov/?tab=home). Given that SHAe are captured by assaying sonicated ChIPed DNA fragments (Figure 5A), it is reasonable to argue that they can correspond to more elongated *cis*-acting elements, *in vivo*.

Supplementary Materials and Methods

Bioinformatics analysis, Algorithms, Statistics, and Computational Biology Tools

RNA-seq Data Analysis

The primary RNA-seq data obtained from NGS were analyzed through the Galaxy platform (https://usegalaxy.org/) (135). Initial quality Control (QC) procedures were conducted on raw sequencing reads via the FastQC application (Galaxy Version 0.72+galaxy1) to ascertain the integrity and fidelity of the data. Subsequent alignment of processed sequencing reads to the human (hg19) or mouse (mm9) reference genomes was conducted by the HISAT2 alignment tool (Galaxy Version 2.2.1+galaxy0) under "single-end" and "unstranded" options (136) for HL and NM and "single-end", "stranded" and "reverse" options for MRC-5 and NIH/3T3 RNA-seq analyses. To evaluate the quality of the RNA samples and the enrichment of RNA-sequencing reads within gene bodies, the Transcript Integrity Number (TIN) tool (Galaxy Version 2.6.4.1) and the Gene Body Coverage (BAM) tool (Galaxy Version 2.6.4.3), were employed from RSeQC package, respectively (137). The quantification of the sequencing reads mapped into genes was conducted by the htseq-count tool (Galaxy Version 0.9.1+galaxy1), in "union" mode, with "unstranded", "minimum alignment quality 1" and "–nonunique

All" options (138) for HL and NM cells and in "union" mode, with "stranded", "reverse", "minimum alignment quality 1" and "–nonunique All" options (138) for MRC-5 and NIH/3T3 cells, delivering a quantitative measure of gene expression, forming the foundational dataset for subsequent differential gene expression analyses.

Differential Gene Expression Analysis

The decisive point of this analysis lies in the application of the edgeR algorithm, a statistical framework. Differential Gene Expression analysis was conducted using the edgeR algorithm (implemented in RNA-seq 2G web portal and Galaxy online platform, Galaxy Version 3.34.0) (139, 140). The analysis was performed between distinct time points, corresponding to control (SVI 0h; naïve cells) and virus-infected samples (SVI 3h, SVI 6h, or SVI 7h). To ensure the credibility of the results obtained from pair-wised comparisons of gene expression levels between naïve and virus-infected samples, stringent cutoffs of total counts, derived additively from control and virus-infected samples, were implemented. Specifically, counts ≥20 for HL, NM, and MRC-5 cells and counts ≥14 for NIH/3T3 cells were applied for each processed gene. Normalization of gene counts was achieved using the median of ratios method, among all samples of the individual different time points (control and virus-infected samples). This normalization approach is crucial for addressing the variability in sequencing depth and any technical limitations across different time points, ensuring more accurate and unbiased comparisons of the gene expression levels.

Computational Analysis of DEGs

In line with published computational strategies (6), the identification of vrDEGs was based on two criteria: First, a stringent p-value threshold, ensuring the significance of the observed changes, and second, statistically significant differential expression values (FC), between virus-infected and naïve cells, or between poly(I:C)-stimulated cells and naïve cells. Accordingly, $|FC| \geq 2$ ($|\log2(FC)| \geq 1$) and p-value <0.05 were applied in RNA-seq datasets from HL, NM, MRC-5, Calu-3, A549, HEK293, $CD4^+$ T cells, respiratory tissues, airway and brain organoids, macrophages, MEFs, and human dermal fibroblasts. $|FC| \geq 1.5$ ($|\log2(FC)| \geq 0.58$) and p-value <0.05 were applied in RNA-seq datasets from NIH/3T3 cells. This processing ensures for the identification of substantial and consistent changes in gene expression, and excludes any potential "transcriptional noise". Additional steps include the quantification of signal intensity using the bamCoverage tool (Galaxy Version 3.3.2.0.0), under the "normalize to reads per kilobase per million (RPKM)" option (26), which converted BAM files to coverage bigwig files and delivered the normalized RPKM. Visualization of *transcriptomics* signals was conducted by Integrated Genomics Viewer (IGV) that provides high-resolution snapshots into the exact genomic coordinates where transcription occurs (141). The same process was followed for computational analyses of publicly available datasets, when applicable.

scRNA-seq analyses on publicly available Datasets

The filtered feature-by-barcode count matrices (.h5) from Aso et al. (73) and Triana et al. (75) were downloaded from GEO (GSE218199 and GSE171620) and processed using Seurat v5.0.0 (142). For

each study, the datasets from naïve and virus-infected cells were merged, and only high-quality cells were selected. In both studies, cells with at least 3 transcribed genes were selected. Additional filters were applied to keep cells with 200-5000 (73) and 600-5000 (75) unique features and less than 10% mitochondrial reads. The resulting-filtered Seurat objects were normalized with the "NormalizeData" function using 'LogNormalize' and scale.factor=10.000 parameters and the top 2,000 variable genes were detected with the "FindVariableFeatures" function and using the "vst" method. Next, the "ScaleData" function was applied and principal component analysis (PCA) was performed using default parameters. UMAP non-linear dimensionality reduction was performed with "dims=1:30" and the result was evaluated to determine if the integration process across the 2 different conditions is necessary. In cases where this was necessary, the SCTransform was performed using "SCTransform" function for the Seurat object of each condition, individually, with "vst.flavor=v2" parameter for Aso et al. datasets (73). The 2 different datasets were subjected to "SelectIntegrationFeatures" in order to identify the 3000 highly variable genes for the integration process. Then, "PrepSCTIntegration" and "FindIntegrationAnchors" were conducted using "normalization.method=SCT" and data were integrated with "IntegrateData" function and "normalization.method=SCT". The 2 different datasets from naïve and infected cell states in Triana et al. (75) were integrated using the 2000 highly variable genes and "normalization.method=LogNormalize". Next, PCA and UMAP with "dims=1:30" were conducted in the integrated datasets. Cell clusters were identified by employing "FindNeighbors" with "dims=1:30" and "FindClusters" functions. Cell-type annotation for the PBMCs cell clusters from Aso et al. (73) was automatically performed based on the reference PBMCs datasets from Hao et al. (143) (https://satijalab.org/seurat/articles/multimodal_reference_mapping.html#a-multimodal-pbmc-reference-dataset using "FindTransferAnchors" function with "normalization.method = SCT, reference.reduction = spca, dims = 1:50" parameters and "MapQuery" function). For the ileum organoids cell clusters, the cell-type specific marker genes normalized expression (75) was utilized to manually determine the cell types. For the PBMCs infected with Influenza A virus (74), pre-processing included only the selection of the naïve and the virus-infected cells, since the Seurat object was directly available from GEO (GSE164922) in .rds format. Differential gene expression analysis for the studies of PBMCs infected with Sendai and Influenza A between the infected and naïve cells for each cell type separately was performed through "FindMarkers" function using the MAST test, while for the ileum organoids, the Wilcoxon rank-sum test (two-sided) was used. Genes detected in at least 10% of either <u>v</u>irus-<u>i</u>nfected or <u>n</u>aïve cell populations were used for differential expression analysis ("min.pct=0.1") and DEGs were selected according to a $FC^{vi/n} \geq 1.18$ ($|log2(FC)| \geq 0.25$) and p-value <0.05. For heatmaps, the average log-normalized expression for each cell type in naïve and virus-infected cell states individually was computed through the "AverageExpression" function.

GOs, KEGG, and Reactome of DEGs

GOs, KEGG, and Reactome pathways/functions analyses on vruDEGs were performed through clusterProfiler R package (144) utilizing enrichGO and enrichKEGG functions, and ReactomePA R package, respectively. Biological terms with p-adjusted <0.05 were considered statistically significant.

The results were subjected to semantic reduction by REVIGO with default parameters and "medium size" result list (145), and illustrated as dot plots in R (ggplot2 package).

DNaseI-seq and ChIP-seq Data Analysis

The primary DNaseI-seq and ChIP-seq data obtained from NGS were analyzed through the Galaxy online platform (https://usegalaxy.org/) (135). QC steps were employed on raw sequencing reads by the FastQC application (Galaxy Version 0.72+galaxy1). Adaptors' contaminants and low-quality reads were trimmed, and thus excluded from the downstream analyses by the application of Trim Galore (Galaxy Version 0.6.7+galaxy0). Trimmed sequencing reads were mapped to the reference human (hg19) or mouse (mm9) genome by the application of Bowtie2 tool (Galaxy Version 2.4.2+galaxy0), with "very sensitive end to end" parameters (146). Duplicate reads were eliminated using the RmDup tool (Galaxy Version 2.0.1) of the SAMtools package (147). Samples were normalized to the same sequencing depth using the Downsample SAM/BAM (Galaxy Version 2.18.2.1) tool from Picard. Peaks were called by MACS2 callpeak algorithm (Galaxy Version 2.1.1.20160309.6) following the parameters as outlined: (a) band width=150 bp, (b) lower mfold=5, (c) upper mfold=50, and (d) q value=0.05 (148). For ChIP-seq analysis, the input data were utilized as controls. Peaks detected in chrY and unplaced contigs (chrUn, chrM) and in ENCODE hg19 or mm9 blacklisted regions were excluded from the downstream analyses (149). Reads that mapped within peaks were tallied using bedtools' MultiCovBed tool (Galaxy Version 2.30.0) (150).

On-genome Distribution, Annotation, GOs, Classification of DNaseI-seq, ChIP-seq Peaks, and SHAe-CRMs

Mapping of signals' genomic localization (promoters, exons, introns, distal intergenic regions, etc.) was employed through ChIPseeker (Galaxy Version 1.18.0+galaxy1) (151) with default parameters. Bar plots were generated by the application of the ggplot2 R package. DNaseI-seq and ChIP-seq peaks, and SHAe were annotated to the coordinates of the human (hg19) or mouse (mm9) genome and assigned to the most proximal TSSs by the Genomic Regions Enrichment of Annotations Tool (GREAT) (version 4.0.4), by utilizing whole-genome as background, and "single nearest gene" parameter (152). GOs were conducted through GREAT and Biological Processes with p-value <0.05 for binomial and hypergeometric tests were considered statistically significant. Dot plots were generated by the application of the ggplot2 R package.

Analyses of enriched/virus-inducible DNaseI-seq and ChIP-seq peaks between naïve and virus-infected cells

Virus-inducible peaks were defined according to their FC between naïve (SVI 0h) and virus-infected cells (SVI 6h) [$SVI^{6h/0h}$ cutoff ≥2 for DNaseI-seq and ChIP-seq peaks, (except from: H3K4me3 ≥1.5 in HL, IRF3 ≥1.5 in NIH/3T3 cells, IRF3 ≥1.5 in A549 cells and H3K27ac ≥1.5 in THP-1 cells)], and filtered based on a minimum number of reads at 6h upon SVI ranged from 15 to 40. Signals' intensity was quantified using the bamCoverage tool from deeptools package (Galaxy Version 3.3.2.0.0) with "normalize to reads per kilobase per million (RPKM)" and "average fragment size=150 bp" options

(26), which converted BAM files to coverage bigwig files. IGV analysis displays the signals across their genomic regions of residence (141).

TFBMs Enrichment Analysis

MEME-ChIP (32) and AME (Analysis of Motif Enrichment) (153), were applied using the motifs retrieved from JASPAR 2022 CORE non-redundant vertebrate database (http://jaspar.genereg.net). MEME-ChIP was applied using the options “DNA -mod zoops or mod anr -minw 4 -maxw 15” and AME with -scoring mean -fisher method -hit-lo-fraction 0.25 -control shuffle. TFBMs were evaluated both for their enrichment (E-value threshold) and their statistical significance (p-value <0.05).

TFBMs enrichment analysis of Chromatin Hotspots

First, the underlying DNA sequences of pre-printed and newly-established CHs were retrieved through getfastabed tool from bedtools. Considering the higher number of pre-printed CHs relative to newly-established in both cell types, we proceeded in random sampling of an equal number of pre-printed CHs (100 subsamplings) utilizing seqinr R package, based on a published workflow (154). Such analyses necessitate an internal control including regions from the rest of the genome. These were charted by first binning the hg19 in length-matched bins with the median length of pre-printed CHs utilizing bedtools “makeWindowsbed” tools. Next, hg19 ENCODE blacklisted regions and the total spectrums of pre-printed and newly-established CHs were excluded from the above pool of loci, by applying the “subtractbed” tool from bedtools. The underlying DNA sequences of interest from the rest of the genome were extracted by utilizing “getfastabed”; given the large size of the fasta file, Biostrings R package was utilized for 100 random subsamplings Next, AME (Analysis of Motif Enrichment) (153) was performed locally for each of the subsampled set of regions utilizing the motifs retrieved from JASPAR 2022 CORE non-redundant vertebrate database (http://jaspar.genereg.net) with parameters: --scoring avg --method fisher --hit-lo-fraction 0.25 --evalue-report-threshold 10.0 --control --shuffle-- --kmer 2, utilizing the median E-value for the selected TFBMs when required.

Classification SEs

[a] According to their anatomical pattern of assembly: Common SEs between naïve and virus-infected cells are termed pmSEs; SEs exclusively structured in naïve cells are termed pdSEs; SEs exclusively structured in virus-infected cells are termed viSEs. [b] According to cell states: In naïve states, the total of pdSEs and pmSEs is classified, while in antiviral states, the total of pmSEs and viSEs is classified. [c] According to single or double-epigenomic labeling: Intersection of H3K27ac- and MED1-ROSE-datasets was employed to distinguish single- or double-marked SEs.

QRTMs on SEs

Heatmaps depicting the RPKM normalized signal distribution and aggregation plots for the mean signal enrichment across SEs genomic loci were generated by deeptools package with “computeMatrix” in scale-regions mode. plotProfile and plotHeatmap from deeptools package were used to visualize signal density (26).

Regional Epigenomic Analysis

Epigenomics signals and chromatin accessibility levels derived from ChIP-seq and DNaseI-seq experiments respectively, were quantified in TSS-proximal loci of the three clusters of common vruDEGs in HL and NM as follows: (a) ±300 bp for TFs and co-activators ChIP-seq, and DNaseI-seq, data and (b) ±600 bp for histone marks ChIP-seq data. Across these regions, the average signal density is enriched. ComputeMatrix in scale-regions mode was employed to quantify RPKM normalized signal distribution from each ChIP-seq or DNaseI-seq experiment prior to (SVI 0h) and upon (SVI 6h) virus-infection in the aforementioned loci with a binSize of 50. The average signal for each TSS-proximal locus was derived from the mean of signal values of each bin. Values are visualized as box plots through the ggplot2 R package and the statistical significance of the identified differences between the clusters of vruDEGs was evaluated/ calculated using the Kruskal-Wallis test. Upon rejection of the null hypothesis of no difference between the clusters of vruDEGs (p-value <0.05), the estimation of pair-wise significance by Dunn's test and Holm correction was followed.

Epigenetic Potential for Gene Expression Regulation

Epigenomics signals were quantified within the TSS-proximal loci (±2 kb) of the three clusters of vruDEGs and compared to the respective expression levels. RPKM normalized signal distribution (±2 kb around the TSSs of vruDEGs), from DNaseI-seq and ChIP-seq experiments was calculated by ComputeMatrix in scale-regions mode, with a binSize of 50, prior to (SVI 0h) and upon virus-infection (SVI 6h). The average signal of each TSS-proximal locus, for each sample, was then calculated as the mean of signal values of each bin. In addition, gene expression levels of vruDEGs were computed as the mean of the normalized counts of the two biological replicates prior to (SVI 0h) and upon virus-infection (SVI 6h). The association between epigenomic signals and expression levels was tested by the Spearman rank correlation test, calculating the correlation coefficient and p-value and the relationship between the numerical variables. The results were visualized as scatterplots through the ggplot2 R package.

Upset Plots

SEs genomic coordinates were intersected with RNA pol II- and CBP-ChIP-seq peaks in naïve cells (SVI 0h), and RNA pol II-, CBP-, IRF3-, and p65-ChIP-seq peaks, in virus-infected cells (SVI 6h). The number of the targeted SEs by the factors examined and their co-occupancies were computed and illustrated by ComplexUpset R package (https://github.com/krassowski/complex-upset) (155), an efficient tool for handling and displaying complex sets of overlapping data.

ChIP-STARR-seq Analysis

The primary ChIP-STARR-seq data, as obtained from the sequencing process, were analyzed by the application of the Galaxy online platform (https://usegalaxy.org/) (135). QC steps were employed on raw sequencing reads by the FastQC application (Galaxy Version 0.73). Adaptors' contaminants and low-quality reads, were trimmed, and thus excluded from the downstream analyses, by the application of Trim Galore (Galaxy Version 0.6.7). Trimmed reads were aligned to the reference genome (hg19)

with the Bowtie2 tool (Galaxy Version 2.4.2+galaxy0), with “very sensitive end to end” parameters (146). Samples were normalized to the same sequencing depth using the Downsample SAM/BAM (Galaxy Version 2.18.2.1) tool from Picard. Genome browser tracks for data visualization were generated using bamCoverage (Galaxy Version 3.5.1.0.0) for ChIP-STARR-seq and STARR-Input libraries with the following outlined parameters: (a) scaling/normalization method=Reads per Kilobase per Million (RPKM), (b) compute an exact scaling factor=YES, (c) custom length of extension reads=150, (d) center regions=YES (26). SAMtools “view” (Galaxy Version 1.13) was used to filter reads based on their proper mapping quality scores (MAPQ ≥10, -q=10) as previously described (93, 156) and the datasets derived from the technical replicates were combined through MergeSAMFiles from Picard. Peak calling was conducted with MACS2 callpeak (v2.1.2.2) (--bw 300, --qvalue 0.01, --keep-dup all). The sequences that correspond to specific genomic coordinates and derived from the merged STARR-Input library replicates were utilized as the input control during peak calling. Peaks derived from the merged ChIP-STARR-seq datasets (technical replicates) with FDR ≤0.01, enrichment over input ≥3, and average ≥25 counts, were considered statistically and biologically significant. A reference catalog of the total statistically significant peaks, as obtained from analyses in naïve and virus-infected datasets, was generated by merging and concatenating (bedtools “merge” -d=50) as previously described (93). Peaks identified in chrY, unplaced contigs (chrUn, chrM) and in ENCODE hg19 blacklisted regions (149) were excluded from the downstream analyses. Genome-wide correlation analyses of read distribution per replicate between STARR-Input library and the corresponding cloned ChIP-library were performed by multiBamSummary from deeptools using filtered, for mapping quality, reads. The human genome was binned into 5,000 bp bins and the reads per bin were calculated and transformed in natural log upon adding 1. The results were analyzed in R, computing pair-wise the Pearson correlation coefficient and p-value and visualized as scatterplots through the ggplot2 R package.

Identification of SHAe

The number of mapped reads obtained, from both technical replicates per cellular state (SVI; 0h, 6h), of each STARR-active element, was calculated by multicovbed tool, and used to compute its virus-inducible differential activation (FC; $SVI^{6h/0h}$) between naïve and infected cells, using edgeR (version 3.34.0) with p-value normalization method: Benjamini and Hochberg and Normalization method: TMM as previously described (23, 93, 156). To conduct rigorous evaluation and define SHAe, a cutoff FC $SVI^{6h/0h}$ ≥1.44, and p-value <0.05, were applied. In addition, an average of ≥40 counts for IRF3-ChIP-STARR-seq and ≥30 counts for p65-ChIP-STARR-seq was applied for the final selection. Furthermore, to enrich our rationale, a more stringent analysis was performed as previously described (23) and validated spectrums of SHAe captured by both pipelines (≥75% for IRF3-SHAe, ≥40% for p65-SHAe) thus suggesting that our study is not dominated by sequencing biases and inherent limitations. The final cohort of SHAe charted is composed of the full spectrum of unique SHAe identified by those two individual computational workflows. Importantly, the genomic loci that were represented by ≥3 fragments, in the STARR-Input library were evaluated in the downstream analysis, following previously published strategies (154). Virus-inducible differential activation of SHAe (FC;

SVI$^{6h/0h}$) was computed as described above, with the addition of a filter of 0.5 Counts Per Million (CPM) in at least two of the four datasets processed. SHAe that do not apply for the last criterion were excluded from the results.

STARR-seq signals computing derived from IR- and κBR-HCTFBSs and non-HCTFBSs-SHAe

Normalized STARR-seq signals were computed by the application of bamCoverage. Signals derived from merged replicates of ChIP-STARR-seq and STARR-Input library were assessed by the aforementioned parameters: (a) scaling/normalization method=Reads per Kilobase per Million (RPKM), (b) compute an exact scaling factor=YES, (c) custom length of extension reads=150, (d) center regions=YES. ChIP-STARR-seq signal was then normalized with STARR-Input-library coverage through bigwigCompare (-bs 50 --operation subtract --pseudocount 1). ComputeMatrix in scale-regions mode (--regionBodyLength 500) was employed to quantify RPKM signal (input subtracted) for each SHAe with a binSize of 50. The average signal for each SHAe was derived from the mean of signal values of each bin. Values were visualized as box plots through the ggplot2 R package and the statistical significance of the difference between IR-, κBR-HCTFBSs and non-HCTFBSs-SHAe was calculated by the Wilcoxon ran-sum two-sided test.

Phylogenetic Tree

97 genomes from distinct organisms were scanned with the "cIR-motif". The evolutionary relationship of the 97 organisms was assessed through the Taxonomy Database in NCBI. The Common Tree option was utilized to generate the phylogenetic tree. More specifically, the Common Tree illustrates a hierarchical view of the relationships between the taxa and their lineages, while the length of each branch represents the evolutionary time between two nodes. Then, the phylogenetic tree was archived in PHYLIP format and utilized by the interactive Tree Of Life (iTOL) for visualization purposes.

Identification of mouse and human Orthologous Genes in RNA-seq and ChIP-seq assays

Orthology data were obtained from Alliance of Genome Resources (version 5.3.0) and processed in order to retrieve the orthologous genes between human and mice. We associated the mouse genes neighboring IRF3-inducible-targeted loci in NIH/3T3 with their corresponding human orthologous gene. The mouse loci that neighbor orthologous HeLa vruDEGs were selected for downstream analysis.

TEs Copies Expression Quantification

To examine TEs copies expression, we utilized the RNA-seq reads, as aligned for "TEs families expression quantification" analysis with the RNA STAR aligner. The assignment of uniquely aligned reads to TE copies was conducted with featureCounts (Galaxy Version 2.0.1) using –the primary parameter. The required gtf annotation file for featureCounts was retrieved from TEtoolkits repository (https://hammelllab.labsites.cshl.edu/software/#TEtranscripts) and TEs copies that overlap gene exons were excluded from the quantification process. To identify the vruDTTEs copies between SVI

0h and SVI 6h, we used edgeR (version 3.34.0) with p-value normalization method: Benjamini and Hochberg and Normalization method: TMM. TEs copies with a sum of counts less than 10 were filtered out from the differential expression analysis. TEs copies with a cutoff FC $SVI^{6h/0h}$ ≥1.6, and p-value ≤0.05 were considered as vrDTTEs. The same process was conducted for downregulated DTTEs.

Intronic TE Copies

The DNA sequence of vruDTTEs copies that overlap intronic regions was assessed for polyA or polyT stretches at their ends in order to validate their autonomous mechanisms of expression. The presence of polyA or polyT sequences enables TEs-transcripts isolation by the polyA-beads-based selection of total RNA. The MAST algorithm was utilized to screen the DNA sequences of intronic vruDTTEs for a 15 bp polyA or polyT "motif". A percentage of ~13% (HL) and 15% (NM) of intronic vruDTTEs were identified to harbor DNA stretches of at least 15 bp at the end of their sequence, thus validating their capability for autonomous expression independently of the expression of the gene that they inhabit.

Evaluation of the Relative Distance between DTTEs and SHAe

To investigate the proximity of vruDTTEs to 3,367 SHAe in HeLa cells in comparison with the total IRF3- and p65-STARR-Active-elements utilized as background, we evaluate their relative distance through bedtools "reldist" function. The statistically significant proximity of vruDTTEs and SHAe compared to backgrounds was validated with Holm adjusted Dunn's test in 0.1 cutoff in the relative distance.

Sequence alignment of the human IR//κBR-HCTFBS *IFIH1* TSS-proximal locus with the orthologous mouse *Ifih1* TSS-proximal locus

The sequence alignment was based on lift-over analysis and conducted using ClustalW (Galaxy Version 2.1) with default parameters. The visualization was performed by MView through EMBL-EBI web-based sequence analysis tools (157).

Intersection of the genomics data with those described in Andrews et al. 2023

The intersections with Andrews et al. published data were conducted upon converting the coordinates of cCREs from hg38 to hg19 genome assembly.

Identification of SHAe with DNA sequences conserved in viral genomes

A comprehensive analysis was conducted on 3,367 composite sequences of SHAe to evaluate their conservation with viral genomes, using the bioinformatics tool Blast (Basic Local Alignment Search Tool) for nucleotides by NCBI (158). This tool specializes in identifying regions of nucleotide sequence similarity by comparing input sequences against well-curated reference databases. For this analysis, the Core Nucleotide Database (core_nt) and the RefSeq Genome Database (refseq_genomes) were utilized as reference datasets, offering comprehensive and high-quality

genomic resources. To focus exclusively on viral organisms, the taxonomic identifier taxid:10239, corresponding to *Viruses*, was applied. The use of BLASTn enabled the identification of local sequence alignments, allowing for a precise assessment of the degree of similarity between the SHAe sequences and the viral genomic content. By employing these specialized databases and the viral-specific taxonomic filter, a robust framework was established to uncover potential associations or evolutionary relationships between SHAe and viral genomes. The analysis was further refined by applying stringent criteria, selecting only those alignments that showed query coverage—defined as the percentage of the query sequence length included in alignments against the sequence match—and percentage identity ≥ 70%. This rigorous approach ensured the specificity and reliability of the results, facilitating the identification of conserved regions that may suggest a potential viral origin for the SHAe sequences.

Identification of viral DNA segments inhabitance within non-human-orthologous IRF3 virus-inducible peaks in NIH/3T3.

IRF3 virus-inducible peaks in NIH/3T3 mouse fibroblasts (mm9) upon SVI 6h were examined for their orthology in human (hg19) utilizing the UCSC liftOver tool (43) with "-minMatch = 0.5" and the appropriate UCSC Pair-wise Chain Alignment. Mouse IRF3-ChIPed DNA sequences that failed to lift-over in the human genome were considered as non-human-orthologous and were further examined for the inhabitance of viral DNA segments within their composite sequences utilizing BLASTn (158). The Core Nucleotide Database (core_nt) and the RefSeq Genome Database (refseq_genomes) were utilized as reference datasets, for "Viruses" (taxid:10239), as described above. We applied stringent criteria including the percentage of query coverage derived from BLASTn (40%-100%) and the length of sequences aligned to viral genome ≥ 100bp, and a 70% cutoff in percentage identity in at least one viral genome, to demarcate the ssvrCRMs.

Identification of short variants within or vastly proximal (±2.5 kb) to SHAe

To identify short variants clinically-related with human disease phenotypes within or vastly proximal (±2.5 kb) to SHAe, extended SHAe coordinates were intersected with the ClinVar database (ClinVar SNVs) (159), retrieved from UCSC Table Browser (hg19). Short variants including single nucleotide variants, deletions, insertions or short-length duplications were then filtered based on their associated phenotypes, focusing on those linked to immune-related diseases. Disease enrichment analysis for the closest vruDEGs (GREAT Analysis) of the SHAe that inhabit the genomic loci of the selected short variants was performed through clusterProfiler R package utilizing enrichDO function (144).

Schemes and Graphics

Parts of some figures have been created by BioRender.com

Supplementary Figures Legends

Supplementary Figure S1. Computational processing of the gene expression kinetics, FC-based clustering, and functional assignment of the 167 common vruDEGs in human Epithelial cells (HL) and B-lymphocytes (NM). (A) RNA-seq heatmaps depict the relative expression of the 167 common vruDEGs during the assembly of the common virus-stimulated gene expression programs in NM (left panels) and HL (right panels). These include highly, mildly, and moderately induced vruDEGs ranked based on to their FCs. Secreted effectors (e.g., *IFNβ*, *IFN-λ*) establish low basal expression and aggressive transcriptional changes compared to TFs (e.g., members of the STAT, NFKB, and IRF families), while PRRs execute high basal expression followed by variable virus-stimulated induction. In NM cells, the virus-stimulated transcriptional changes are acquired earlier (SVI 3h) than in HL. A spectrum of common vruDEGs is differentially classified between the clusters of NM and HL. **(B)** KEGG pathway and Reactome analyses illuminate the tremendous specificity of the common transcriptome shaped in NM and HL, respectively, for defensive cellular pathways and processes effective against diverse viral strains or other immunogenic stimuli. A detailed chart is provided in Supplementary Table S1.

Supplementary Figure S2. Computational processing of the gene expression kinetics, FC-based clustering, and functional assignment of the cell-type-specific vruDEGs in human Epithelial cells (HL) and B-lymphocytes (NM). (A) and **(B)** upper panels: RNA-seq heatmaps depict the relative expression of the 323 cell-type-specific vruDEGs in NM (e.g., IFN alpha family) and of the 299 in HL (e.g., *IL-8*, *IL1A*) during the assembly of the cell-type-specific virus-stimulated gene expression programs. In NM (left panels) and HL (right panels) virus-stimulated transcriptomes are composed of highly, mildly, and moderately vruDEGs ranked based on their FCs. Secreted effectors establish low basal expression and aggressive transcriptional changes compared to TFs, while PRRs execute high basal expression followed by variable virus-stimulated induction. In NM cells, the transcriptional changes are acquired earlier (SVI 3h) than in HL. **(A)** and **(B)** lower panels: GOs and Reactome analyses illuminate significant divergence of the cell-type-specific transcriptomes shaped in NM and HL, which are engaged to diverse cell fates decisions and pathways (e.g., cellular senescence in HL, IFN signaling in NM), and they are committed to alternative processes (e.g., response to virus in NM, gene expression and epigenome regulation in HL). These are substantially tailored to the unique identity, physiology, functional traits, and significance for organism homeostasis and to the stress inflicted under the emergence of the microbial challenge. A detailed chart is provided in Supplementary Table S1.

Supplementary Figure S3. Gene expression programs investigation in NM, HL, and MRC-5 cells prior to and upon SVI. (A) Transcriptional states profiling in cell-type-specific vruDEGs gated in NM and HL C/I, C/II, and C/III; Box plots depict the distribution of the average log_2 normalized expression values of each vruDEG per time point and the average $log_2(FC)$, as computed in biological replicates. Boxplots are constructed as detailed in materials and methods and Figure 1 Legend. Two-

sided Wilcoxon rank-sum test was applied to the total cell-type-specific vruDEGs of each cluster [C/I: 63 in NM and 26 in HL; C/II: 88 in NM and 95 in HL; C/III: 172 in NM and 178 in HL)]. The results depict statistically significant differences in basal levels of expression between C/I and C/II in NM (p-value= $3.73e^{-16}$), and in HL (p-value=$6.91e^{-5}$); C/I-C/III in NM (p-value<$2.2e^{-16}$), and in HL (p-value=$9.55e^{-9}$); C/II-C/III in NM (p-value=$2.46e^{-12}$), and in HL (p-value=$9.34e^{-10}$). These results unravel the significant heterogeneity of basal levels of expression of cell-type-specific vruDEGs in naïve cells and underscore that the high levels predominantly correlate with medium or minimal transcriptional induction upon virus-infection, in NM (left panel) and HL (right panel). **(B)** Computational processing of the gene expression kinetics, the FC-based clustering, and functional assignment of the 146 MRC-5 vruDEGs shared with the spectrum of 167 common vruDEGs in HL and NM. RNA-seq heatmaps depict the relative expression of the 146 MRC-5 vruDEGs. These include highly, mildly, and moderately induced vruDEGs ranked according to their FCs. Secreted effectors (e.g., *IFNβ*, *IFN-λ*) establish low basal expression and aggressive transcriptional changes compared to TFs (e.g., members of the STAT and IRF families), while PRRs execute high basal expression followed by variable virus-stimulated induction. A spectrum of these vruDEGs is differentially classified between the clusters of MRC-5, NM, and HL. **(C)** Transcriptional states profiling in the MRC-5 146 vruDEGs gated in C/I, C/II, and C/III; Same as in (A): Box plots depict statistically significant differences in basal levels of expression in MRC-5 between C/I and C/II (p-value=$8.99e^{-10}$), and C/I and C/III (p-value=$7.98e^{-7}$) but not C/II and C/III (p-value=0.33) (two-sided Wilcoxon rank sum test). High basal levels of expression predominantly correlate with medium or minimal transcriptional induction upon virus-infection. **(D)** KEGG pathway analyses illuminate the tremendous specificity of the 146 vruDEGs for defensive cellular processes/pathways effective against distinct *Viruses*. **(E)** GOs sharply highlight the specificity of the 146 vruDEGs for defensive cellular processes/pathways, e.g., defense response to virus, regulation of innate immune response, etc. **(F)** Reactome analysis illuminates the tremendous specificity of the 146 vruDEGs for defensive cellular pathways and antiviral cellular mechanisms, e.g., Interferon pathways, ISG15 antiviral mechanism, SARS-CoV-2 infection. A detailed chart is provided in Supplementary Table S1. **(G)** Constitutive immunity molecules encoded from vruDEGs, including restriction factors, are broadly expressed prior to and upon SVI in NM, HL, and MRC-5. These results underscore the commitment of basal levels of vruDEGs expression in establishing primary defense layers under homeostasis in human cells.

Supplementary Figure S4. Tracing functional fingerprints of ubiquitously applied mechanisms of defense-oriented gene expression; Gene expression investigations in diverse human systems infected with distinct *Viruses*. (A) Upper Tables: Several hundreds of vruDEGs are shared between the plethora of human cell systems listed, NM, and HL. These results imply the assembly of common gene expression signatures and the function of similar mechanisms that regulate defense-oriented gene expression in diverse cell types upon infection with distinct *Viruses*. A detailed analysis is provided in Supplementary Table S1. Lower tables: Constitutive immunity molecules encoded from vruDEGs, including restriction factors, are broadly expressed prior to and upon infection with distinct *Viruses* in the diverse human cell systems examined. These results

underscore the commitment of basal levels of vruDEGs expression in establishing primary defense layers under homeostasis in human cells. **(B)** GOs applied on the total vruDEGs of each system of human cells highlight substantial specificity for defensive cellular processes and immune functions, e.g., defense response to virus, cytokine pathways, etc. Selected striking ontologies are demonstrated.

Supplementary Figure S5. scRNA-seq investigations of the virus-stimulated gene expression programs establishment in human PBMCs. Publicly available scRNA-seq datasets were computationally processed; Heatmaps depicting the differential expression of the common vruDEGs of NM and HL at the single-cell level in distinct cell types of (i) Human PBMCs naïve or infected with SV and (ii) Human PBMCs naïve or infected with IAV. Only the vruDEGs that were captured expressed beyond the cell-number threshold applied in such scRNA-seq assays are shown. For each cell type, the average normalized expression of each gene is derived from the total cell population and is illustrated in the heatmap that demonstrates the relative expression of the gene in between the total cell types in naïve and infected cells. (i and ii) left panels; GOs applied on unique/individual cell-types complement these gene expression investigations and highlight striking examples of the top-ranking ontologies, that are predominantly associated with defensive and immune processes. (i and ii) right panels; Gated into blue boxes are striking examples of well-known vruDEGs that are captured to acquire nearly uniform transcriptional changes in between the same cell types, when infected with distinct *Viruses* (SV or IAV). These results indicate significant similarity of the patterns of virus-stimulated gene expression programs establishment in both analyses of PBMCs. These profiles are in line with those emerged from NM, HL, MRC-5 and the additional cell systems studied, thus confirming the broad application of regulatory mechanisms of defensive gene expression in human cells of distinct developmental origins.

Supplementary Figure S6. (A) scRNA-seq investigations of the virus-stimulated gene expression programs establishment in human ileum-derived organoids. Same as in Supplementary Figure S5; scRNA-seq investigations applied on publicly available scRNA-seq datasets highlight the virus-stimulated gene expression programs establishment in human ileum-derived organoids (HAstV1 infection; 0h, 16h). The results indicate substantial similarity with the patterns of virus-stimulated gene expression programs established in PBMCs (SV or IAV), NM, HL, MRC-5 (SV), and the additional cell systems infected with distinct *Viruses* studied, thus confirming the broad application of regulatory mechanisms of defensive gene expression in diverse human cells. **(B-D)** Gene expression programs investigations in mammalian cells stimulated with a nucleic acid analog of viral genomes trace functional fingerprints of ubiquitously applied mechanisms of defense-oriented gene expression (Computational assessments of publicly available RNA-seq datasets). **(B)** Upper Table: Several hundreds of poly(I:C)-induced DEGs in human dermal fibroblasts and mouse embryonic fibroblasts (MEFs) are shared with the vruDEGs of NM and HL. These results confirm the assembly of common gene expression signatures within the transcriptomes shaped in diverse cell types and organisms upon challenging with distinct stimuli (e.g., virus infection or poly(I:C)-stimulation), across distinct

mammalian species (mouse and human). A detailed analysis is provided in Supplementary Table S1. **(C)** Constitutive immunity molecules encoded by poly(I:C)-induced DEGs, including restriction factors, are broadly expressed prior to and upon stimulation in both systems examined, and are also encoded from vruDEGs in HL and NM. **(D)** GOs applied on the total poly(I:C)-induced DEGs of each system highlight substantial specificity for defensive cellular processes and immune functions, e.g., defense response to virus, cytokine pathways, etc. Selected striking ontologies are demonstrated.

Supplementary Figure S7. Interspecies transcriptomics investigations. (Ai-Aiv) NIH/3T3 mouse fibroblasts were assayed prior to and upon SVI (0h, 7h) in transcriptomics experiments. RNA-seq datasets revealed a wide spectrum of mouse and human orthologous genes being upregulated upon SVI in NIH/3T3 (mvruDEGs). Intersections of mvruDEGs with the NM and HL common (i) and cell-type-specific (ii and iii), and the MRC-5 vruDEGs (iv) uncover significant overlaps. GOs highlight significant specificity for virus-stimulated and innate immune responses in mouse cells, thus indicating the operation of similar regulatory mechanisms of defensive gene expression programs establishment in both mammalian species. Part of Supplementary Figure S7 was created in BioRender: Agelopoulos, M. (2025) https://BioRender.com/l05t872

Supplementary Figure S8. High-resolution analyses resolved the *epigenomics* states of CHs. IGVs snapshots of *genomics* tracks illustrate the *in vivo* reconstitution of CHs, at selected genomic loci in naïve and virus-infected NM and HL. Red (NM) and blue (HL) horizontal lines and rectangles distinguish the assembly of CHs between the cell types. Topographic maps of striking examples of common vruDEGs (*NFKB2*, *IL15RA, ZFP36L2*), cell-type-specific vruDEGs (*TNFSF13B*, *IL7R*), and intergenic loci that host CHs are illustrated. In principle, common vruDEGs are associated with CHs in both cell types, while cell-type-specific vruDEGs with cell-type-specifically *in vivo* reconstituted CHs. Importantly, the intergenic CHs assembled ~55 kb distal from *TNFAIP3* (as computed through GREAT) are not accompanied by RNA-seq signals, in both cell types, thus verifying the specificity of our workflow. These results underscore the functional conductance of CHs in defensive gene expression programs establishment.

Supplementary Figure S9. CHs *in vivo* reconstitution *in cis* proximity to common vruDEGs in human Epithelial cells (HL) and B-lymphocytes (NM). (A) Table summarizes the workflow of epigenome-wide, sequential, overlaid intersections applied on NGS-peaks obtained from DNaseI-seq and ChIP-seq assays, in naïve (SVI 0h) and virus-infected cells (SVI 6h). Thousands of complete CHs enriched for “open” chromatin, H3K27ac, MED1, and RNA pol II are identified in both cell types and states (red boxes). In virus-infected cells, the (co)-occupation of CHs by IRF3, p65, and CBP is assessed individually or in distinct combinations and leads to the identification of sharp epigenetic profiles, including few hundreds in which the above factors are coinciding (orange boxes). These results highlight that robust epigenome supervision marks CHs *in vivo* reconstitution. **(B)** Left Tables: Summarizes the percentages of CHs-associated vruDEGs (common and cell-type-specific) within C/I, C/II, and C/III, in naïve and virus-infected cells. The vast majority of CHs-associated vruDEGs are

classified in C/II and C/III while significantly less in C/I in naïve NM and HL. Upon virus-infection a more balanced association between CHs and vruDEGs is established among C/I, C/II, and C/III in both cell types. Right Tables: The table charts the percentage of "pre-printed" (SVI 0h and 6h) and "newly-established" (SVI only 6h) CHs (co)-occupied by IRF3, p65, CBP. **(C)** Same as in Figure 1C. **(i)** DNA Grammar assessments highlight an enriched lexicon of TFBMs legible by antimicrobial TFs in total and in IRF3-, p65-, and CBP-ChIPed, "pre-printed" and "newly established" CHs and (iii) Genomic localization assessments mapped the "pre-printed" CHs more excessively within promoter regions (ii) compared to "newly established" CHs. **(D)** Same as in Figure 1D. QRTMs within the TSS-proximal (±2 kb) chromatin microenvironments of residence of the common vruDEGs in NM; Aggregation plots (signals' average) and heatmaps (signals' coverage) illustrate the regional *epigenomics* signals as assembled in naïve and virus-infected NM within the genomic loci of residence of common vruDEGs (NM: SVI; 0h, 3h, 6h). C/II and C/III are "epigenetically triggered" for transcriptional activation in naïve cells in line with their enhanced basal levels of expression. C/I vruDEGs reside in epigenetically fingerprinted chromatin microenvironments lacking hallmarks of transcriptional activation, in line with their constrained levels of expression. Upon virus-infection, C/II and C/III vruDEGs maintain or slightly enhance their "archetype" epigenetic features, which become divergently (co)-occupied by antimicrobial TFs and CBP, whereas C/I gradually acquire "open" chromatin, MED1, H3K27ac, H3K4me3, and RNA pol II accompanied by robust binding of antimicrobial TFs and CBP.

Supplementary Figure S10. Topographic maps of the TSS-proximal (±2 kb) chromatin microenvironments of common vruDEGs in NM and HL. High-resolution IGVs snapshots of *genomics* tracks illustrate the epigenetic and transcriptional states as established around TSS of *CCL5* (C/I), *DDX60* (C/II) and *STAT2* (C/III) prior to (SVI 0h) and in the course of virus-infection (SVI; 3h, 6h), in both cell types. CHs assemblies, histone marks, TFs and co-activators recruitment, RNA pol II "traveling", and RNA-seq signals are highlighted. In principle, *DDX60* (C/II) and more intensively *STAT2* (C/III) chromatin microenvironments of residence are enriched in epigenetic marks/factors connected to active transcription in naïve cells and become gradually enhanced in virus-infected cells, while *CCL5* (C/I) acquires such characteristics predominantly in virus-infected cells, in both cell types. Such chromatin states divergences support distinct mechanisms of basal and virus-inducible gene expression between the clusters of common vruDEGs. Bottom IGVs snapshots illustrate in high resolution RNA-seq signals from the entire *DDX60* and *STAT2* genomic loci.

Supplementary Figure S11. Comparative analysis of epigenetic characteristics and transcriptional levels of vruDEGs (common and cell-type-specific) in B-lymphocytes (NM) and Epithelial cells (HL) prior to and upon virus-infection. (A-B) Scatterplots show the correlation between chromatin accessibility (DNaseI-seq), histone modifications (H3K27ac, H3K4me3), transcriptional apparatus occupancy (RNA pol II), binding of antimicrobial TFs [IRF3, NFκB (p65 subunit)] and co-activators recruitment [CBP, Mediator complex subunit 1 (MED1)], within the TSS-proximal loci (±2 kb) of C/I, C/II, and C/III vruDEGs and their corresponding expression levels in NM

(A) and HL (B). The scatterplots were constructed by the assessment of the average DNaseI- and ChIP-seq signals (RPKM normalized signal) around ±2 kb of the TSSs of vruDEGs (blue color; C/I, red color; C/II, yellow color; C/III) and the $\log_2$ normalized expression values of each DEG (mean of the normalized counts of the RNA-seq biological replicates). Spearman's rank correlation test calculated the correlation coefficient and the results are shown in each panel. The results depict statistically significant positive correlations between chromatin accessibility, epigenetic characteristics, TFs and co-activator recruitment within the TSS-proximal loci (±2 kb) of vruDEGs, and the expression levels established. These findings unveil that the divergence in vruDEGs stimulation (tremendous for C/I, moderate for C/II, and mild for C/III) is under robust epigenetic supervision. For antimicrobial TFs and CBP only the antiviral cell states were examined.

Supplementary Figure S12. (A-B) Box plots demonstrate the statistical validation of the results describing the molecular events within the TSS-proximal chromatin microenvironment of vruDEGs. *Epigenomics* signals derived from TFs, co-activators, and histone modifications ChIP-seq assays, and chromatin accessibility signals derived from DNaseI-seq assays, in NM (A) and HL (B), were quantified to RPKM through computeMatrix. The TSS-distance threshold was determined according to the signals' on-genome aggregation (±300 bp for TF and co-activators ChIP-seq, and DNaseI-seq, and ±600 bp for histone modifications ChIP-seq). The boxplots depict signal strength for each cluster of common vruDEGs in naïve and antiviral cell states, as derived from the average signal of the individual TSS-proximal loci examined. Boxplots are constructed as detailed in materials and methods and Figure 1 Legend. Statistically significant differences between the clusters were calculated using Kruskal-Wallis test. Then, on the approved examinations [Kruskal-Wallis (p-value<0.05)], we computed the pair-wise significance of the difference with Dunn's test and Holm correction. As shown, statistically significant differences (Dunn's test corrected p-value≤0.05) (*: p-value≤0.05, **: p-value≤0.01, ***: p-value≤0.001, ****: p-value≤0.0001) are identified in both cell types and states and validate the regulatory principles of the divergence in transcriptional responsiveness in between the distinct clusters of vruDEGs. **(C)** Same as in Figure 1D and Supplementary Figure S9D. QRTMs within the TSS-proximal (±2 kb) chromatin microenvironments of residence of the cell-type-specific vruDEGs in NM (left panels) and HL (right panels). Cell-type-specific QRTMs validate the epigenetic supervision in transcriptional regulation prior to and upon virus-infection in human cells and highlight CHs assembly and cell-type-specific fingerprints of TFs and CBP recruitment. Aggregation plots (signals' average) and heatmaps (signals' coverage) illustrate the regional *epigenomics* signals as assembled in naïve and virus-infected NM and HL within the genomic loci of residence for all three clusters of cell-type-specific vruDEGs (HL: SVI; 0h, 3h, 6h). In naïve cells, C/II and C/III are "epigenetically triggered" for transcriptional activation via "open" chromatin, histone modifications (H3K27ac, H3K4me3), RNA pol II and MED1, establishment, which facilitate their high basal levels of expression. In contrast, C/I vruDEGs reside in epigenetically fingerprinted chromatin microenvironments that nearly abolish the above hallmarks of transcriptional activation, consistent with their constrained levels of expression. Upon virus-infection, C/II and C/III vruDEGs maintain (DNaseI, H3K27ac, H3K4me3, and RNA pol II) or slightly enhance (MED1) their "archetype"

epigenetic features, while C/I maintain or minimally enhance (RNA pol II) the low levels of their pre-printed characteristics. The results highlight CHs assembly and cell-type-specific targeting by TFs and co-activators, as expected. Despite the limited enrichment in cases of microbial TFs, manual curation of well-known vruDEGs and individual assessments validate their cell-type-specific regulation by IRF3 (*IFN*-α family, *IFN*-ε, *IFN*-ω) and p65 (*IL1A*, *IL6*, *IL8*), etc.

Supplementary Figure S13. Virus-stimulated epigenomic fingerprints hallmark the transition from naïve-to-antiviral states in human and mouse cells and correlate with defensive responses. (A) Genomic localization assessments of virus-inducible *epigenomics* signals (NM, HL; $SVI^{6h/0h}$); TFs, co-activators, chromatin accessibility, and H3K27ac shape nearly balanced on-genome allocation whereas H3K4me3 and RNA pol II (to a lesser extent) are prevalently enriched across intragenic sequences and promoter regions, thus verifying the specificity of our workflow in capturing how epigenetic marks become diffused upon virus-infection in NM and HL. **(B)** Same as in Figure 1E. Comparative GOs highlight the tremendous specificity of the virus-stimulated *epigenomics* signals obtained from the full spectrum of assays for antiviral, immune and defense-oriented cellular processes in NM e.g., response to cytokine, response to organism, negative regulation of viral genome replication, etc. **(C)** QRTMs on virus-inducible signals centered ±2 kb; Aggregation plots (signals' average) and heatmaps (signals' coverage) illustrate the gradual enrichment of *epigenomics* signals as shaped during the transition from naïve to antiviral cell states in NM (upper panels) and HL (lower panels) (SVI; 0h, 3h, 6h). Regardless of the assay and the cell type examined, aggressive inducibility is captured. (D) IRF3- and H3K27ac-ChIP-seq in A549 human lung cells and NIH/3T3 mouse fibroblasts. Genomic localization assessments of virus-inducible IRF3- and H3K27ac-ChIP-seq signals highlight the distribution of the TF and H3K27ac across the genome in virus-infected cells (SVI 6h). Left panel: GOs on virus-inducible IRF3- and H3K27ac-ChIP-seq signals show substantial specificity for defense and immune-response cellular processes in virus-infected cells (SVI 6h). Right panel: QRTMs on IRF3- and H3K27ac-ChIP-seq virus-inducible signals in A549 and NIH/3T3 cells (centered ±2 kb); Aggregation plots (signals' average) and heatmaps (signals' coverage) illustrate the gradual enrichment of signals as shaped during the transition from naïve to antiviral cell states in A549 and NIH/3T3 cells (SVI; 0h, 6h). Part of Supplementary Figure S13 was created in BioRender: Agelopoulos, M. (2025) https://BioRender.com/d90s319

Supplementary Figure S14. Fingerprints of virus-infection are monographed within epigenetically marked chromosomal landscapes enriched in TFBSs legible by antimicrobial TFs. (A) and **(B)**: Analysis of the lexicon of TFBSs encompassed in DNA sequences across which DNaseI, MED1, H3K27ac, H3K4me3, RNA pol II, IRF3, p65, and CBP virus-inducible *epigenomics* signals (enriched in virus-infected cells compared to naïve cells $SVI^{6h/0h}$) in HL (A) and NM (B). Regardless of the assay and the cell type examined, endogenous sequences enriched in DNA grammar characteristics legible, among others, by members of the microbial-activated families of TFs e.g., IRFs, RELA, STATs, FOS/JUN, etc., and more rarely members of FOX family, are elucidated. These results highlight the extensive global epigenome reprogramming triggered by virus-infection in

human cells and underscore the regulatory logic followed for its interpretation. **(C)** Same as in A and B. Analysis of the lexicon of TFBSs encompassed in DNA sequences across which H3K27ac and IRF3 virus-inducible *epigenomics* signals (enriched in virus-infected cells compared to naïve cells $SVI^{6h/0h}$) in A549 human lung cells (left) and NIH/3T3 mouse fibroblasts cells (right). Part of Supplementary Figure S14 was created in BioRender: Agelopoulos, M. (2025) https://BioRender.com/w38l038

Supplementary Figure S15. (A) Same analysis as in Figure 3A. Upper panels: Ranking plots emerged from ROSE-analyses applied on H3K27ac-ChIP-seq experiments depict the *in vivo* reconstitution of SEs and tEs, in naïve (SVI 0h) and virus-infected (SVI 6h) Epithelial cells (HL) and B-lymphocytes (NM). Striking examples of vruDEGs residing *in cis* proximity to SEs are highlighted. Middle panels: Classification of SEs according to the ROSE MED1- and H3K27ac-ranking. A significant portion of SEs has been captured by both *epigenomics* markers as indicated by the assessments employed by the algorithm, thus validating the specificity of our workflow. Lower panels: GOs depict the specificity of the full spectrum of SEs for cellular functions in each cell type and state. Immune system processes in NM and negative regulation of apoptosis and programmed cell death, gene expression and biosynthesis in HL are among others highlighted. A more detailed analysis employed on SEs classes or subclasses based on their epigenetic marking is provided in Supplementary Figure S17A, C. **(B)** QRTMs in NM across the genomic coordinates of viSEs double-marked with H3K27ac and MED1, prior to and upon virus-infection. Aggregation plots (signals' average) and heatmaps (signals' coverage) illustrate the *epigenomics* signals that emerged in naïve (SVI 0h; blue line) and virus-infected cells (SVI 6h; red line) corresponding to H3K27ac, MED1, DNaseI, RNA pol II, IRF3, p65, and CBP as distributed across viSEs genomic coordinates. "Open" chromatin structures become enriched for the full spectrum of epigenetic markers/factors examined in a virus-inducible fashion, across expanded epigenomic entities; a hallmark of SEs assembly. The incorporation of antimicrobial TFs, co-activators, and transcriptional apparatus in those viSEs is indicative of their functional conductance in defensive cellular mechanisms.

Supplementary Figure S16. SEs genomic localization highlights extensive epigenome reprogramming phenomena that match the gene expression characteristics as steady-stated prior to and exchanged upon virus-infection in NM. (A) Chromosomal Ideograms (e-karyotypes) illustrate SEs *in vivo* reconstitution in naïve (upper panel) and virus-infected (bottom panel) NM. Horizontal blue lines within chromosomes depict gene density. Green squares label pdSEs, magenta circles label pmSEs, and red arrows/triangles label viSEs. Black boxes label vruDEGs expressed at the cell state of examination that reside within ±0.5 Mb from SEs (SEs-associated vruDEGs). Striking examples of SEs-associated vruDEGs are highlighted above each human chromosome. Upper right: bar graph depicts the percentage of allocation (%) of the 138 SEs-associated expressed vruDEGs in between C/I, C/II, and C/III in naïve NM. Bottom right: bar graph depicts the percentage of allocation (%) of the 281 SEs-associated expressed vruDEGs in between C/I, C/II, and C/III in virus-infected

NM. The anatomical patterns of SEs assembly match the gene expression programs as established in naïve and virus-infected NM cells. **(B)** Left panel: GOs applied on viSEs single-marked by MED1 in NM depict substantial specificity for response to biotic stimulus and organisms, type I IFN signaling, immune system processes, etc. Right panel: QRTMs across viSEs single-marked by MED1 genomic coordinates in NM: Aggregation plots (signals' average) and heatmaps (signals' coverage) illustrate the *epigenomics* signals that emerged in naïve (SVI 0h; blue line) and virus-infected cells (SVI 6h; red line) corresponding to H3K27ac, MED1, DNaseI, RNA pol II, IRF3, p65, and CBP as distributed across viSEs genomic coordinates. "Open" chromatin structures become enriched for the full spectrum of epigenetic markers/factors examined in a virus-inducible fashion and variable strengths across expanded epigenomic entities; a hallmark of SEs assembly. The incorporation of antimicrobial TFs, co-activators, and transcriptional apparatus in those viSEs is indicative of their functional conductance in defensive cellular mechanisms. **(C)** SEs genomic localization and transcriptional regulation of linearly associated vruDEGs in naïve and infected NM. The workflow is the same as in Figure 4C. Boxplots depict the average $\log_2$ normalized expression values of SEs-associated and SE-far-distant vruDEGs allocated in C/I, C/II, and C/III in naïve (SVI 0h) (blue) and virus-infected cells (SVI 6h) (red) as computed in biological replicates. Two-sided Wilcoxon rank-sum test computed the statistical significance in the divergence of expression levels between the SE-associated and SE-far-distant vruDEGs of each cluster [naïve NM: C/I p-value=$8.35e^{-5}$, C/II p-value=$5.44e^{-5}$, C/III p-value=$1.53e^{-7}$; virus-infected NM: C/I p-value=0.0016, C/II p-value=$5.12e^{-6}$, C/III p-value=0.0009]. Gray-colored numbers next to boxes highlight the number of SEs-associated or SEs-far-distant expressed vruDEGs, respectively. Significantly, higher diversification in basal expression levels between SEs-associated and SEs-far-distant vruDEGs for all three clusters in naïve cells, is recorded. C/II and C/III are enriched in SEs-associated expressed vruDEGs compared to C/I in naïve cells. In virus-infected cells, both the diversification in expression levels and the spectrums of SEs-associated and SEs-far-distant vruDEGs of C/I, C/II, and C/III are more balanced.

Supplementary Figure S17. (A,C) A survey of GOs employed on subclasses of SEs distinguished according to their single or double epigenetic marking. **(A)** Fully equipped SEs with CBP, RNA pol II, IRF3, and p65 in virus-infected NM (left panel) and HL (right panel). In NM, cellular response to cytokine and response to stress, while in HL, immune system processes and response to stress are dominant. **(B)** SEs architectural states validation. Shown are scatterplots depicting the Fold Change (FC; $SVI^{6h/0h}$) of the RPKM-normalized ChIP-seq signals derived from H3K27ac and MED1 assays in NM (upper panels) and HL (lower panels). The genomic coordinates that host SEs are ranked in ascending FCs. In naïve and virus-infected cells, the genomic coordinates of pdSEs, pmSEs and viSEs were examined and the results show patterns of epigenetic alterations that match to the SEs architectural states. In brief, the vast majority of pdSEs' genomic coordinates diminish the strength of the signals, those of pmSEs maintain a balance within their population and those of viSEs predominantly acquire significant enhancements, during the transition from naïve to antiviral states, in both NM and HL. **(C)** pmSEs double-marked by H3K27ac and MED1 are correlated with immune system processes in NM (upper left panel) and negative regulation of metabolic processes in HL

(upper right panel). pm and viSEs correlate, among others, with type I IFN signaling pathway (IRF3-targeted), regulation of immune system processes (p65-targeted), and response to cytokine (IRF3/p65-targeted), in NM (left panels). In HL, pm and viSEs correlate among others with negative regulation of metabolic processes (IRF3-targeted, p65-targeted, and IRF3/p65-targeted), and immune system processes (IRF3/p65-targeted) (right panels). These results underscore the significance of human SEs for vital cellular processes, including defensive responses.

Supplementary Figure S18. Topographic maps of human viSEs. High-resolution IGVs snapshots of *genomics* tracks illustrate the epigenetic and transcriptional states of *in vivo* reconstituted SEs in naïve and virus-infected Epithelial cells (HL) and B-lymphocytes (NM). Red (NM) and blue (HL) horizontal lines and rectangles distinguish the coordinates as ranked by the ROSE algorithm. Left panel: Shown is the genomic locus of *ZFP36* common vruDEG that hosts viSEs in both cell types. The entire sequence of the gene is embedded within the viSEs. Right panel: Shown is the genomic locus of *KLF4*, a HL-specific vruDEG that hosts a HL-specific viSE. In contrast, the same locus in NM cells resides within an “epigenetically repressed” chromatin microenvironment, that lacks chromatin accessibility, histone activation marks, TFs, etc. These profiles match the gene expression characteristics of *KLF4* as steady-stated in naïve cells and exchanged upon virus-infection, in both cell types, since this vruDEG is transcriptionally silenced in NM. Red (NM) and blue (HL) horizontal lines and rectangles distinguish the coordinates as ranked by the ROSE algorithm.

Supplementary Figure S19. Topographic maps of human viSEs. Same as in Supplementary Figure S18. Left panel: Shown is the genomic locus of *ISG15* common vruDEG that hosts a viSE, in HL. The entire gene sequence is embedded within the viSE. Robust epigenetic signals are also mapped in NM, a pattern that poises for the application of similar epigenomic mechanisms of transcriptional regulation. Right panel: Shown is the genomic locus of *CCL5* common vruDEG that hosts viSEs, in both cell types. The entire gene sequence is embedded within the viSEs. Red (NM) and blue (HL) horizontal lines and rectangles distinguish the coordinates as ranked by the ROSE algorithm.

Supplementary Figure S20. Topographic maps of the *IFIT* gene cluster. Same as in Supplementary Figures S18 and S19. Shown is the genomic locus of the IFIT cluster composed of *IFIT2*, *IFIT3*, *IFIT1B*, *IFIT1*, and *IFIT5* genes. Upper panel: In NM, *IFIT1B* is not expressed and the respective genomic coordinates are characterized by the absence of SEs. *IFIT2* is embedded entirely and *IFIT3* partially within viSEs, and both genes are vruDEGs in NM. *IFIT1* and *IFIT5* are highly expressed vruDEGs in NM (Table S1). Lower panels: In HL, several tEs are mapped across the IFIT cluster, such as in *IFIT2* and *IFIT3* that both are vruDEGs. *IFIT1B* is not expressed and the respective genomic coordinates are characterized by the absence of SEs. *IFIT1* and *IFIT5* are highly expressed vruDEGs. Red (NM) and green (HL) horizontal lines and rectangles distinguish the coordinates of SEs and tEs as mapped by the ROSE algorithm.

Supplementary Figure S21. Topographic maps of human tEs. High-resolution IGVs snapshots of *genomics* tracks illustrate the epigenetic and transcriptional states of *in vivo* reconstituted tEs in naïve and virus-infected Epithelial cells (HL) and B-lymphocytes (NM). TSS-proximal and TSS-distal tEs that neighbor vruDEGs are illustrated. Orange (NM) and Green (HL) horizontal lines distinguish tEs genomic coordinates as ranked by the ROSE algorithm. Genomic loci of housekeeping genes (*ACTB*, *ACTG1*), repressed genes (*AFM*, *MYOD1*) and manually curated tEs of vruDEGs (*DDX58*, *CXCL10*, *IFI44*, etc.) are used as controls. The distances for TSS-distal tEs were computed through GREAT.

Supplementary Figure S22. SEs genomic localization and transcriptional regulation of associated vruDEGs in naïve and infected HL and NM. (A) Percentages (%) of SEs-associated vruDEGs within C/I, C/II, and C/III for both naïve and virus-infected HL and NM are charted in the left rows of the table. Antiviral defense-related biological processes associated with the total spectrum of SEs in each cell type and state as derived from GOs are listed in the middle rows. Striking examples of SEs-associated and expressed genes, including vruDEGs, corresponding to biological processes, are shown in the right columns of the tables. **(B)** 3D investigations for SEs *in vivo* reconstitution proximal to vruDEGs in naïve HeLa cells captured several pairs of vruDEGs and SEs to co-inhabit (neighbor, reside proximal or entire coincide) within chromosomal domains in naïve HL cells (e.g., chr6: BTN3A1/SEs). Blue rectangles indicate SEs. Additional data are described in Supplementary Table S3. **(C)** Dotplots depict the average $\log_2$ normalized expression values in SVI 0h and SVI 6h for Group I (GI); associated in naïve and infected cells (blue-colored), and Group II (GII); non-associated in naïve but associated in infected cells (red-colored) for each gene. GI members broadly establish: dominant patterns of basal expression, lesser extents of transcriptional enhancements, and consequently, lower transcriptional inductions over the time of infection compared to GII. GOs highlight that both groups of genes exhibit substantial specificity for immune/antiviral cellular processes, thus underscoring the resolution and validity of the analyses conducted based on this classification. Lower panels; left and right: Statistically significant (p-value<0.05) diversification of RNA pol II occupancies are identified across the TSSs of GI and GII SEs-associated genes, in both HL and NM. The average RPKM signal across TSS-proximal loci (± 2kb) was computed. Two-sided Wilcoxon rank-sum test was applied for the GI-GII SEs-associated genes for each timepoint of infection (SVI 0h, 3h, 6h).

Supplementary Figure S23. (A) Graphical representation of the distribution of the values of FCs that correspond to the virus-inducible upregulation of STARR-transcripts by IRF3-SHAe (left) and p65-SHAe (right). Most SHAe execute strong (FC≥2) stimulation of the synthesis of 5'-GFP:*cis*-element:polyA-3' mRNA hybrids, *in vivo*. This verifies their capability to work as virus-inducible enhancers and validates the specificity of our workflow. IRF3 binds in an analog mode across the endogenous sequences of IRF3-SHAe, which, as shown, is dictated by the number of perfect TFBSs encompassed. p65 binds in a semi-analog mode across the endogenous sequences of p65-SHAe, which, as shown, is dictated by the number of perfect TFBSs encompassed until the threshold of 3 sites. The association between the number of perfect TFBSs and the binding strength was assessed

by Spearman's correlation coefficient (IRF3-SHAe: R=0.64; p-value<$2.2e^{-16}$; p65-SHAe: R=0.17; p-value=$6.1e^{-12}$). **(B)** The complete catalog of SHAe-associated vruDEGs in virus-infected HL (SVI 6h). **(C)** Integrative analyses of the SHAe genomic coordinates in alternative human cell types infected with SV for 6h. The results highlight that an extended spectrum of SHAe genomic coordinates is bound by IRF3 and marked by H3K27ac 6h upon SVI of B-lymphocytes (NM) and lung cells (A549) while exhibit outstanding specificity for antiviral and defensive cellular processes. The genomic coordinates of SHAe were extended bidirectionally (±2.5 kb) to avoid exclusion of biologically meaningful coincidences (±2.5 kb). **(D)** Lift-over analyses were conducted to investigate the corresponding genomic regions of the human 1,949 IRF3-SHAe and 1,601 p65-SHAe in the mouse genome. The results revealed that 882 out of the 1,949 IRF3-SHAe (~45%) (upper left panel) and 805 out of 1,601 p65-SHAe (~50%) (upper right panel) are orthologous in the mouse genome. Yellow and blue tables chart striking examples of human vruDEGs that are orthologous in mice and reside *in cis* proximity to the conserved SHAe regions. GOs applied on the 882 mouse orthologous IRF3-SHAe show substantial specificity for response to other organism and biotic stimulus, defense response to virus, and regulation of cytokine production (bottom left panel). GOs applied on the 805 mouse orthologous p65-SHAe highlight, among others, correlation with leukocyte differentiation, positive regulation of type I IFN production, and apoptotic signaling pathways (bottom right panel). These results indicate a significant conservation of IRF3- and p65-SHAe between the human and the mouse genomes, suggesting a potential functional relevance. Part of Supplementary Figure S23 was created in BioRender: Agelopoulos, M. (2025) https://BioRender.com/m72h605

Supplementary Figure S24. Correlations of *epigenomics*-signals vs SHAe-STARR-seq-signals: IRF3, p65, MED1, CBP, H3K27ac, H3K4me3, and RNA pol II occupancies, and chromatin accessibility were investigated in correlation to TFs-STARR-seq strength, in virus-infected HL cells (SVI 6h). **(A)** DNaseI- and ChIP-seq-signals vs IRF3-STARR-seq signals: The scatterplots depict the average signal intensities. The signals were quantified to RPKM (normalized signal distribution) through computeMatrix across each IRF3-SHAe endogenous locus by utilizing ±2 kb flanking distance from its center as the reference point. The scatterplots depict the correlation of the signals and their level of association as assessed by Spearman's rank correlation coefficient. Overall, strong positive correlations/linear associations are demarcated. IRF3 binding and IRF3-SHAe virus-induced activation are significantly correlated (R=0.82, p-value<$2.2e^{-16}$). Similar strong positive correlations were emerged from the rest of the examinations. **(B)** Same as in (A). DNaseI- and ChIP-seq-signals vs p65-STARR-seq signals. Overall, strong positive correlations/linear associations are demarcated. p65-binding and p65-SHAe virus-induced activation are significantly correlated (R=0.65, p-value<$2.2e^{-16}$). Similar strong positive correlations were identified for the rest of the examinations. **(C)** Same as in (A) and (B). DNaseI- and ChIP-seq signals vs IRF3-STARR-seq signals: The scatterplots depict the average signal intensities. The signals were quantified to RPKM (normalized signal distribution) through computeMatrix across each IRF3/p65-SHAe endogenous locus by utilizing ±2 kb flanking distance from its center as the reference point. The scatterplots depict the correlation of the signals and their level of association as assessed by Spearman's rank correlation coefficient. Overall,

strong positive correlations/linear associations are demarcated between IRF3 binding and IRF3/p65-SHAe virus-inducible activation (R=0.83, p-value<2.2e^{-16}) and p65 binding and IRF3/p65-SHAe virus-inducible activation (R=0.44, p-value=1.4e^{-11}). Similar strong positive correlations were emerged from the rest of the examinations. **(D)** Same as in (C). DNaseI- and ChIP-seq-signals vs p65-STARR-seq signals. Overall, strong positive correlations/linear associations are demarcated between p65 binding and IRF3/p65-SHAe virus-inducible activation (R=0.69, p-value<2.2e^{-16}) and IRF3 binding and IRF3/p65-SHAe virus-inducible activation (R=0.66, p-value<2.2e^{-16}). Similar strong positive correlations were emerged from the rest of the examinations.

Supplementary Figure S25. (A) Left panels: Aggregation plots (signals' average) and heatmaps (signals' coverage) illustrate the ChIP-STARR-seq signals generated from repetitive- (left) and non-repetitive (right) IRF3-SHAe in naïve (SVI 0h; green line) and virus-infected HL (SVI 6h; red line). Blue line corresponds to the signals derived from direct NGS of the STARR-Input library. Repetitive-IRF3-SHAe execute more aggressive upregulation of STARR-transcripts compared to non-repetitive-IRF3-SHAe. Right panels: QRTMs illustrate the regional *epigenomics* signals as assembled in naïve and virus-infected HL within the endogenous loci of residence of repetitive- and non-repetitive-IRF3-SHAe (HL: SVI; 0h, 3h, 6h). In principle, both classes of IRF3-SHAe are variably targeted in a virus-inducible fashion by IRF3, p65, and CBP. Interestingly, the epigenetic marks/factors of CHs ("open" chromatin, H3K27ac, MED1, and RNA pol II) are pre-printed in naïve cells and maintained in the course of virus-infection across the genomic coordinates of non-repetitive-IRF3-SHAe rather than repetitive-SHAe that primarily acquire these characteristics from the ground state upon virus-infection. H3K4me3 modification assembles stable patterns in both classes, yet more enriched in non-repetitive-IRF3-SHAe. Interestingly, non-repetitive-IRF3-SHAe exhibit stronger binding of p65 compared to repetitive-IRF3-SHAe, presumably due to the saturation of the sequence of repetitive-IRF3-SHAe with IRF3 TFBSs that can hinder the binding of other TFs. This can influence IRF3-SHAe activation capacities. **(B)** Left panels: Aggregation plots (signals' average) and heatmaps (signals' coverage) illustrate the ChIP-STARR-seq signals generated from repetitive- (left) and non-repetitive (right) p65-SHAe in naïve (SVI 0h; green line) and virus-infected HL (SVI 6h; red line). Blue line corresponds to the signals derived from direct NGS of the STARR-Input library. Repetitive-p65-SHAe and non-repetitive-p65-SHAe execute nearly equal rates of virus-inducible upregulation of STARR-transcripts. Right panels: QRTMs illustrate the regional *epigenomics* signals as assembled in naïve and virus-infected HL within the endogenous loci of residence of repetitive- and non-repetitive-p65-SHAe (HL: SVI; 0h, 3h, 6h). In principle, both classes of SHAe are variably targeted in a virus-inducible fashion by IRF3, and CBP. Interestingly, the epigenetic marks/factors of CHs ("open" chromatin, H3K27ac, MED1, and RNA pol II) are pre-printed in naïve cells and maintained in the course of virus-infection across the genomic coordinates of non-repetitive-p65-SHAe rather than repetitive-SHAe, which mostly acquire these characteristics from the ground state upon virus-infection, thus following the pattern, described above, for repetitive- and non-repetitive-IRF3-SHAe. H3K4me3 modification assembles stable patterns in both classes, yet those are more enriched in non-repetitive-p65-SHAe. Interestingly, non-repetitive-p65-SHAe exhibit slightly stronger binding for p65 and weaker for IRF3 compared to

repetitive-p65-SHAe. This can influence their activation capacities. The above [(A) and (B)] findings indicate common and distinct principles of epigenetically supervised transcriptional activation between the distinct classes of SHAe, and validate that the sequence architecture of a *cis*-element encrypts regulatory underpinnings with functional consequences. **(C)** The evolutionary history of non-repetitive-SHAe: Monitoring within the cohort of the 100 vertebrates classifies ~62% in e-Mammals and ~38% in b-Mammals which are traced within multiple vertebrate genomes. Of the e-Mammals-non-repetitive-SHAe, the cohort of e-Primates corresponds to ~2.5%. **(D)** Computational assessments of the results from Andrews et al.; Left panel: Intersection of cCREs genomic coordinates with pmSEs and viSEs revealed that 1,302 of those expanded (epi)genomic domains are inhabited by diverse groups of those *cis*-elements, in virus-infected HL (HL; SVI 6h) (~74.1% inhabited by multiple groups of cCREs; ~21.8% inhabited by G1; ~2.1% inhabited by G2, and ~1.8% inhabited by G3 cCREs). Right panel: Intersection of the 3,367 SHAe and cCREs genomic coordinates revealed 1,078 common *cis*-elements. These SHAe are differentially allocated in between the groups of cCREs, and predominantly classified to G1 (~67%; highly conserved in mammals), then to G2 (~17.2%; actively evolving) and G3 (~11.6%; primate-specific), whereas a limited spectrum (~4%) encompasses multiple groups of cCREs. These results validate our findings regarding the evolutionary hierarchies of SHAe. In addition, these findings illuminate the functional fitness of the common SHAe/cCREs in human Epithelial cells upon virus-infection (HL; SVI 6h). The intersections with Andrews et al. published data were conducted upon converting the coordinates of cCREs from hg38 over hg19 genome assembly. Part of Supplementary Figure S25 was created in BioRender: Agelopoulos, M. (2025) https://BioRender.com/i18v172

Supplementary Figure S26. **(A-C)** The evolutionary origins of Human-Higher-Primates-enriched SHAe (HHPe-primates); Side-by-side monitoring of the conservation of vrCRMs within the 100 vertebrates' (left panels) and the 241 placental mammals' (right panels) cohorts. **(A)** The entire DNA sequence of this SHAe is not traced in the genomes of other organisms in both cohorts. **(B)** Upper panels: Part of this human DNA sequence is significantly conserved in viral genomes (Supplementary Table S5), including the one of Zika virus. Robust conservation was revealed upon pairwise alignment of human and Zika virus sequences. Lower Panels: The entire DNA sequence of this SHAe exhibits extremely limited conservation with the genomes of some primates, mammals, and other vertebrates (left and right panels). **(C)** Upper Panels: Part of this human DNA sequence is significantly conserved in viral genomes (Supplementary Table S5), including the one of Human endogenous retrovirus K113 virus. Robust conservation was revealed upon pairwise alignment of human and Human endogenous retrovirus K113 sequences. Lower Panels: The entire DNA sequence of this SHAe is conserved in the genomes of some primates, but any significant conservation in other mammals or other vertebrates (left panel) is missing. Part of Supplementary Figure S26 was created in BioRender: Agelopoulos, M. (2025) https://BioRender.com/u09y070

Supplementary Figure S27. (A) The identification of ssvrCRMs in mice. Integrative analyses of DNA sequences corresponding to non-human orthologous IRF3-ChIP-seq peaks assembled in mouse cells

upon virus infection and genomes from murine infectious viruses uncovered the establishment of species-specific vrCRMs with increased functional potential and limited or abolished conservation in the genomes of other vertebrates. Striking examples are highlighted for loci that host two mouse genes that encode for proteins with antiviral function *(Ly6e and Apobec3)* and establish ssvrCRMs that harbor viral-DNA insertions, and the locus that hosts the *Rims2* (non-virus-stimulated gene) that also encompass viral-DNA insertions but does not harbor established ssvrCRMs. **(B)** Computational assessments of the results from Andrews et al.; Upper panel: A striking example of an e-Primates IRF3/p65-SHAe chr22:29,126,259-29,126,733 which harbors the 3rd exon of the vruDEG *CHEK2* and is also classified in G3 exons according to Andrews et al.; Robust conservation was revealed upon pairwise-alignment with multiple viral genomes including those of Baboon cytomegalovirus OCOM4-37 and BeAn 58058 virus. These results highlight that vrCRMs have viral origins and encode primate-specific amino acids of critical human antiviral regulators, thus underscoring the natural conflicts and "symbiotic" interactions between host and pathogens at the functional (epi)genomic and protein levels. Additional examples of vruDEGs that acquired SHAe-encoded exons (blue letters) or G3 exons (black letters) are charted in the table below.

Supplementary Figure S28. (A) Density plots comparatively depict the evolutionary conservation of IR-HCTFBSs, κBR-HCTFBSs, and IR//κBR-HCTFBSs, as computed by the average PhyloP score of each element. In principle, no statistically significant differences are recorded in between these distinct classes of HCTFBSs-SHAe as shown by Kruskal-Wallis test (p-value=0.053). **(B)** Topographic maps of HCTFBSs-SHAe: High-resolution IGVs snapshots of *genomics* tracks illustrate the epigenetic and transcriptional states of striking examples of HCTFBSs-SHAe**.** Left upper panel: The *ISG15* TSS-proximal IR//κBR-HCTFBS is meta-profiled within its endogenous genomic coordinates of residence which hosts CHs enriched in antimicrobial IRF3, p65 and CBP, and H3K4me3 in a virus-inducible fashion in infected HL (SVI; 3h, 6h). Left lower panel: The sequence architecture of this SHAe; Perfect and "half", IRF3 (green and magenta) and p65 (red and blue) TFBSs are underlined. Right panel: The *IFIH1* TSS-proximal IR//κBR-HCTFBS is meta-profiled within its endogenous genomic coordinates of residence, which hosts CHs enriched in antimicrobial IRF3, p65 and CBP in a virus-inducible fashion in infected HL (SVI; 3h, 6h). H3K4me3 modification remains stable prior to and in the course of transition from naïve to antiviral states. Right lower panel: The sequence architecture of this SHAe. Perfect and "half", IRF3 (green and magenta) and p65 (red and blue) TFBSs, are underlined. **(C)** The percentage (%) of recovery of IR-HCTFBSs, total IRF3-SHAe, and IRF3-ChIP-seq total peaks in A549, HL and NM (SVI 6h), by the "cIR-motif". **(D)** Topographic maps of IRHADs in human and mouse cells; High-resolution IGVs snapshots of *genomics* tracks illustrate the epigenetic and transcriptional states of striking examples of IRHADs in human and mouse cells. Shown are the human *HERC6*, *ADAP1*, *ZC3HAV1*, *IL6* loci and the mouse chr6:84,388,081-84,389,144; *Ccl5*; *Prdm9*; and *Ifna4* loci that host biochemically *in vivo* validated IRHADs in HL, NM, A549 and NIH/3T3. The numbers depict the "cIR-motif" instances captured. **(E)** Sequence architecture investigations of human *IFIH1* and mouse *Ifih1* TSS-proximal regions, that correspond to the human IR//κBR-HCTFBS, demonstrate in high-resolution the pattern of residence of IRF3 and NFκB TFBSs in both species.

Interestingly, the human IR//κBR-HCTFBS encompasses IRF3 and NFκB TFBSs that evolved upon the emergence of mice. These binding sites are targeted by these TFs in HL cells upon virus-infection (Figure S28B) and contribute to the functional fitness of the IR//κBR-HCTFBS, and consequently the transcriptional regulation of *IFIH1*. These results imply that the evolutionary shaping of human vrCRMs imposes the fine-tuning of critical defensive genes. Part of Supplementary Figure S28 was created in BioRender: Agelopoulos, M. (2025) https://BioRender.com/f33v950

Supplementary Figure S29. (A) RNA-seq heatmaps depict the transcriptional changes acquired in vrDTTEs NM (upper panel) and in HL (lower panel) upon virus-infection (SVI; 0h, 3h, 6h). **(B)** TEtranscripts tool analyses identified families of vruDTTEs in NM; 58 distinct families of vruDTTEs are captured stimulated e.g., MER57B1, MER20B. **(C)** Upper left panel: Venn diagram depicts common (n=586) and cell-type-specific (n=1,698 in HL; n=3,405 in NM) vruDTTEs copies in HL and NM. Upper right panel: Genomic localization assessments of vruDTTEs of NM and HL. vruDTTEs in both NM and HL are prevalently enriched across intronic regions and intergenic sequences. Middle panels: a comprehensive analysis of GOs in common vruDTTEs between HL and NM, total vruDTTEs in both cell types that show significant specificity for defensive antiviral cellular processes. GOs of common and total vruDTTEs in HL and NM demonstrate substantial specificity for defense response to virus, response to type I IFN, and innate immune response. **(D)** GOs in distal intergenic vruDTTEs of HL and NM; Additionally, distal intergenic vruDTTEs of HL were found to be correlated with defense response to virus, and type I IFN signaling pathway. In NM, distal intergenic vruDTTEs showed correlations, among others, with response to other organism and biotic stimulus, and immune response. **(E)** Binomial test followed by Wilcoxon rank sum test within the first 20 kb highlight statistically significant enrichment of *in cis* proximity between vruDTTEs and vruDEGs, in NM, when total (red line) or distal intergenic copies (black dotted line) were assessed, compared to an equal number of non-virus-upregulated TEs (black dashed line) ($p\text{-value}<2.2e^{-16}$). **(F)** Localization assessments map the distribution (%) of vruDTTEs within SEs, tEs, and safGs of the human genome in HL and NM. **(G)** Topographic maps of human of vruDTTEs. High-resolution IGVs snapshots of *genomics* tracks illustrate the epigenetic and transcriptional states of genomic loci inhabited by vruDTTEs in naïve and virus-infected Epithelial cells (HL) (SVI; 0h, 3h, 6h). Shown are: the chr9:110,495,000-110,517,887 locus that hosts a pmSE and a viSE inhabited by vruDTTEs; the *IRF1/IRF1-AS1 locus* that hosts a viSE and a IRF3/p65-SHAe and encompasses vruDTTEs; and the *DDX58* locus that hosts a SHAe very proximal to vruDTTEs. *DDX58* is also discussed in Supplementary Figure S31. Virus-inducible transcription of DTTEs is depicted in all examples. Blue horizontal lines and rectangles distinguish the coordinates of SEs as mapped by the ROSE algorithm. Orange horizontal and vertical lines, shadows and rectangles label TEs genomic coordinates. Lower panels: Zoomed snapshots of *genomics* tracks display in high resolution the generation of TEs-transcripts.

Supplementary Figure S30. (A) RNA-seq heatmaps depict the transcriptional changes acquired in vrDTTEs of MRC-5 upon virus-infection (SVI; 0h, 3h, 6h). **(B)** Upper panel: GOs applied on

vruDTTEs of MRC-5 demonstrate substantial specificity for defense response to virus, innate immune response, etc. Bottom panel: Genomic localization assessments of MRC-5 vruDTTEs highlight that they are prevalently enriched across intronic regions and intergenic sequences. **(C)** Upper panel: Intersection of the datasets derived from NM, HL, and MRC-5 highlights a “common-core” of more than 100 vruDTTEs. Bottom panel: GOs applied on the “common core” of vruDTTEs demonstrate substantial specificity for defense response to virus, response to biotic stimulus, innate immune response, etc. **(D)** Selected examples of NM, HL, and MRC-5 vruDTTEs in RNA-seq heatmaps. **(E)** GOs applied on mouse vruDTTEs identified by RNA-seq experiments in NIH/3T3 cells (SVI; 0h, 7h). The results highlight substantial specificity for defense response to virus, response to biotic stimulus, innate immune response, etc. **(F)** Upset plot demonstrates the (co)-occupation of ~600 SHAe by antimicrobial TFs and the co-activator MED1. These vrCRMs reside within the genomic loci described in Aracena et al. as marked by epigenomic features/variations and QTLs. (G) Disease enrichment analyses applied on vruDEGs associated in cis with vrCRMs/SHAe that host SNPs and other immune-related short variants.

Supplementary Figure S31. Integrative analyses of SHAe loci that are enriched in SNPs linked to Human Autoimmune diseases. (A) Upper left panel: A description of the workflow. Upper center panel: The transcriptional and *epigenomics* states of the IRF3/p65-SHAe and its flanking ±2.5 kb sequence. These genomic coordinates are encompassed within the *DDX58* genomic locus. A CH is assembled prior to and becomes enhanced upon virus-infection (SVI 0h, 3h, 6h) accompanied by the binding of the antimicrobial TFs (IRF3 and p65) and the recruitment of CBP, thus leading to the *in vivo* reconstitution of functional vrCRMs. RNA pol II travels across the gene body and transcribes its coding region, which apart from protein-coding sequences harbors multiple vruDTTEs (orange lines) that generate TEs-encoded RNAs. One autoimmune-disease-associated SNP is mapped within the above genomic coordinates, and it is linked to Psoriasis (PSO). Upper right panel: The SNP and its associated Human autoimmune disease. **(B)** Upper panel: The evolutionary origins of the SHAe, its flanking ±2.5 kb sequence, and its *in cis* associated SNP within the cohort of 100 representative vertebrate species (phylop100way); In principle, the chr9:32,523,520-32,529,189 locus of the human genome is not traced beyond mammalian species, and its sequence conservation is significantly increased from lower mammals, such as Armadillo, to primates. Middle panel: The sequence architecture of chr9:32,523,520-32,529,189 coordinates of the human genome. TFBSs for IRF1/3 (green letters) and NFκB (red letters), the IRF3/p65-SHAe (blue shadow), and the SNP (brown letter) are highlighted. Part of Supplementary Figure S31 was created in BioRender: Agelopoulos, M. (2025) https://BioRender.com/m89n154

Supplementary Figure S32. Integrative analyses of SHAe loci that are enriched in SNP and QTL linked to Human Autoimmune diseases and severity of Influenza infection. (A) Upper left panel: A description of the workflow. Upper center panel: The transcriptional and *epigenomics* states of the IRF3-SHAe and its flanking ±2.5 kb sequence. These genomic coordinates are encompassed within the *IFITM3* genomic locus and are constituents of the pmSE of the gene (blue line). A CH is

assembled prior to and becomes enhanced upon virus-infection (SVI 0h, 3h, 6h) accompanied by the binding of the antimicrobial TFs (IRF3 and p65), thus leading to the *in vivo* reconstitution of functional vrCRMs. Upper right panel: One autoimmune-disease-associated SNP that is linked to Multiple sclerosis (MS) and one QTL that is associated with the severity of Influenza infection are mapped within the above genomic coordinates. **(B)** Upper panel: The evolutionary origins of the SHAe, its flanking ±2.5 kb sequence, and its *in cis* associated SNP and QTL within the cohort of 100 representative vertebrate species (phylop100way); In principle, the chr11:318,225-323,632 locus of the human genome is traced predominantly within mammalian species, and its sequence conservation is observed from lower mammals to primates. Middle panel: The sequence architecture of chr11:318,225-323,632 coordinates of the human genome. TFBSs for IRF1/3 (green letters) and NFκB (red letters), the IRF3-SHAe (blue shadow), and the SNP and QTL (brown letters) are highlighted. Part of Supplementary Figure S32 was created in BioRender: Agelopoulos, M. (2025) https://BioRender.com/k56z599

Supplementary Figure S33. Model picture: Human cells fight *Viruses via* the virus-responsive fate of their (epi)genome which is programmed by vrCRMs of recent, old or microbial origins, and presumably works in defensive cellular responses against other pathogens.

Supplementary References

135. Afgan,E., Baker,D., Batut,B., van den Beek,M., Bouvier,D., Cech,M., Chilton,J., Clements,D., Coraor,N., Grüning,B.A., et al. (2018) The Galaxy platform for accessible, reproducible and collaborative biomedical analyses: 2018 update. *Nucleic Acids Res.,* **46**, W537–W544.
136. Kim,D., Paggi,J.M., Park,C., Bennett,C. and Salzberg,S.L. (2019) Graph-based genome alignment and genotyping with HISAT2 and HISAT-genotype. *Nat. Biotechnol.,* **37**, 907–915.
137. Wang,L., Wang,S. and Li,W. (2012) RSeQC: quality control of RNA-seq experiments. *Bioinformatics,* **28**, 2184–2185.
138. Anders,S., Pyl,P.T. and Huber,W. (2015) HTSeq--a Python framework to work with high-throughput sequencing data. *Bioinformatics,* **31**, 166–169.
139. Zhang,Z., Zhang,Y., Evans,P. and Chinwalla,A. (2017) RNA-seq 2G: online analysis of differential gene expression with comprehensive options of statistical methods. Preprint at bioRxiv,
140. Robinson,M.D., McCarthy,D.J. and Smyth,G.K. (2010) edgeR: a Bioconductor package for differential expression analysis of digital gene expression data. *Bioinformatics,* **26**, 139–140.
141. Robinson,J.T., Thorvaldsdóttir,H., Winckler,W., Guttman,M., Lander,E.S., Getz,G. and Mesirov,J.P. (2011) Integrative genomics viewer. *Nat. Biotechnol.,* **29**, 24–26.
142. Hao,Y., Stuart,T., Kowalski,M.H., Choudhary,S., Hoffman,P., Hartman,A., Srivastava,A., Molla,G., Madad,S., Fernandez-Granda,C., et al. (2024) Dictionary learning for integrative, multimodal and scalable single-cell analysis. *Nat. Biotechnol.,* **42**, 293–304.

143. Hao,Y., Hao,S., Andersen-Nissen,E., Mauck,W.M.,3rd, Zheng,S., Butler,A., Lee,M.J., Wilk,A.J., Darby,C., Zager,M., et al. (2021) Integrated analysis of multimodal single-cell data. *Cell,* **184**, 3573–3587.e29.
144. Yu,G., Wang,L.G., Han,Y. and He,Q.Y. (2012) clusterProfiler: an R package for comparing biological themes among gene clusters. *OMICS,* **16**, 284–287.
145. Supek,F., Bošnjak,M., Škunca,N. and Šmuc,T. (2011) REVIGO summarizes and visualizes long lists of gene ontology terms. *PLoS One,* **6**, e21800.
146. Langmead,B. and Salzberg,S.L. (2012). Fast gapped-read alignment with Bowtie 2. *Nat. Methods,* **9**, 357–359.
147. Li,H., Handsaker,B., Wysoker,A., Fennell,T., Ruan,J., Homer,N., Marth,G., Abecasis,G., Durbin,R. and 1000 Genome Project Data Processing Subgroup. (2009) The Sequence Alignment/Map format and SAMtools. *Bioinformatics,* **25**, 2078–2079.
148. Feng,J., Liu,T., Qin,B., Zhang,Y. and Liu,X.S. (2012) Identifying ChIP-seq enrichment using MACS. *Nat. Protoc.,* **7**, 1728–1740.
149. Amemiya,H.M., Kundaje,A. and Boyle,A.P. (2019) The ENCODE Blacklist: Identification of Problematic Regions of the Genome. *Sci. Rep.,* **9**, 9354.
150. Quinlan,A.R. and Hall,I.M. (2010) BEDTools: a flexible suite of utilities for comparing genomic features. *Bioinformatics,* **26**, 841–842.
151. Yu,G., Wang,L.G. and He,Q.Y. (2015) ChIPseeker: an R/Bioconductor package for ChIP peak annotation, comparison and visualization. *Bioinformatics,* **31**, 2382–2383.
152. McLean,C.Y., Bristor,D., Hiller,M., Clarke,S.L., Schaar,B.T., Lowe,C.B., Wenger,A.M. and Bejerano,G. (2010) GREAT improves functional interpretation of cis-regulatory regions. *Nat Biotechnol.,* **28**, 495–501.
153. McLeay,R.C. and Bailey,T.L. (2010) Motif Enrichment Analysis: a unified framework and an evaluation on ChIP data. *BMC Bioinformatics,* **11**, 165.
154. Glaser,L.V., Steiger,M., Fuchs,A., van Bömmel,A., Einfeldt,E., Chung,H.R., Vingron,M. and Meijsing,S.H. (2021) Assessing genome-wide dynamic changes in enhancer activity during early mESC differentiation by FAIRE-STARR-seq. *Nucleic Acids Res.,* **49**, 12178–12195.
155. Lex,A., Gehlenborg,N., Strobelt,H., Vuillemot,R. and Pfister,H. (2014) UpSet: Visualization of Intersecting Sets. *IEEE Trans. Vis. Comput. Graph.,* **20**, 1983–1992.
156. Peng,T., Zhai,Y., Atlasi,Y., Ter Huurne,M., Marks,H., Stunnenberg,H.G. and Megchelenbrink,W. (2020) STARR-seq identifies active, chromatin-masked, and dormant enhancers in pluripotent mouse embryonic stem cells. *Genome Biol.,* **21**, 243.
157. Madeira,F., Pearce,M., Tivey,A.R.N., Basutkar,P., Lee,J., Edbali,O., Madhusoodanan,N., Kolesnikov,A. and Lopez,R. (2022) Search and sequence analysis tools services from EMBL-EBI in 2022. *Nucleic Acids Res.,* **50**, W276–W279.
158. Sayers,E.W., Bolton,E.E., Brister,J.R., Canese,K., Chan,J., Comeau,D.C., Connor,R., Funk,K., Kelly,C., Kim,S., et al. (2022) Database resources of the national center for biotechnology information. *Nucleic Acids Res.,* **50**, D20–D26.

159. Landrum,M.J., Lee,J.M., Riley,G.R., Jang,W., Rubinstein,W.S., Church,D.M. and Maglott,D.R. (2014) ClinVar: public archive of relationships among sequence variation and human phenotype. *Nucleic Acids Res.,* **42**, D980–D985.
160. BioRender.com

Supplementary Tables Legends

Supplementary Table S1. Delineation of virus-stimulated gene expression programs in human Epithelial cells (HL) and B-lymphocytes (NM). (A) Differential gene expression analysis in naïve (mock-infected) (SVI 0h) and virus-infected (SVI 3h, 6h) HL. **(B)** Differential gene expression analysis in naïve (mock-infected) (SVI 0h) and virus-infected (SVI 3h, 6h) NM. **(C)** Normalized counts of genes among all samples of the three different time points of infection (SVI 0h, 3h, 6h) in HL (two biological replicates). **(D)** Normalized counts of genes among all samples of the three different time points of infection (SVI 0h, 3h, 6h) in NM (two biological replicates)**. (E)** Identification of common and cell-type-specific virus-stimulated genes in HL and NM. **(F)** FC-based classification of the 167 (SVI 6h) common vruDEGs in HL and NM in three distinguished clusters "C". For each gene, the cellular distribution and function are shown. **(G)** FC-based classification of the cell-type-specific vruDEGs in HL and NM resulted in distinguished clusters "C". **(H)** Determination of the basal levels of expression of vruDEGs in naïve cells (SVI 0h), based on the average normalized expression in biological replicates. **(I)** Differential gene expression analysis in naïve (mock-infected) (SVI 0h) and virus-infected (SVI 3h, 6h) MRC-5, FC-based classification of the vruDEGs in MRC-5 in three distinguished clusters "C" and shared vruDEGs with common and cell-type-specific vruDEGs in HL and NM. **(J)** Normalized counts of genes [log2(CPM)] among all samples of the three different time points of infection (SVI 0h, 3h, 6h) in MRC-5 (two biological replicates). **(K)** Differential gene expression analysis in naïve (mock-infected) (SVI 0h) and virus-infected (SVI 7h) NIH/3T3, and shared vruDEGs with common and cell-type-specific vruDEGs in HL, NM and MRC-5. **(L)** Normalized counts of genes [log2(CPM)] among all samples of the two different time points of infection (SVI 0h, 7h) in NIH/3T3 (two biological replicates). **(M)** Detailed results of the computational analysis of RNA-seq datasets retrieved from human HEK293 upon infection with Rift Valley Fever Virus (RVFV). The differential gene expression analysis and the transcriptional induction of the upregulated DEGs in clusters as well as the vruDEGs shared between human HEK293, NM, and HL are listed. **(N)** Same as in (M) for human A549 upon infection with parainfluenza virus type 3 (HPIV3). **(O)** Same as in (M) for human A549 upon infection with Respiratory Syncytial Virus (RSV). **(P)** Same as in (M) for human A549 upon infection with Dengue Virus 2 (DENV2). **(Q)** Same as in (M) for human A549 upon infection with Influenza A Virus (IAV). **(R)** Same as in (M) for human Calu-3 upon infection with Severe Acute

Respiratory Syndrome Coronavirus 2 (SARS-CoV-2). **(S)** Same as in (M) of RNA-seq datasets retrieved from Primary Human macrophages, isolated from healthy donors, upon infection with West Nile Virus (WNV). **(T)** Same as in (M) of RNA-seq datasets retrieved from human cell cultures established by upper respiratory tract specimens (EpiAirway & EpiAlveolar) upon infection with enterovirus D68 (EV-D68). **(U)** Same as in (M) of RNA-seq datasets retrieved from human $CD4^+$ T cells upon infection with the Ebola virus (EBOV). **(V)** Same as in (M) of RNA-seq datasets retrieved from human pluripotent stem cells (HPSCs)-derived airway organoids upon infection with SARS-CoV-2. **(W)** Same as in (M) of RNA-seq datasets retrieved from human pluripotent stem cells (HPSCs)-derived brain organoids upon infection with Zika Virus (ZIKV). **(X)** Same as in (M) of RNA-seq datasets retrieved from human dermal fibroblasts upon stimulation with poly(I:C). **(Y)** Same as in (M) of RNA-seq datasets retrieved from mouse embryonic fibroblasts (MEFs) upon stimulation with poly(I:C). (**Z**) Detailed results of the upregulated genes retrieved from the computational analysis of scRNA-seq datasets from human PBMCs infected with Sendai virus for 24 hours. The cell-type-characteristic upregulated genes for each distinct cell type are charted. (**AA**) Same as in (Z) for scRNA-seq datasets from human PBMCs infected with Influenza A virus (IAV) for 12 hours. (**AB**) Same as in (Z) for scRNA-seq datasets retrieved from human ileum-derived organoids infected with Human Astrovirus-1 for 16 hours. **(AC)** List of downregulated genes in at least three datasets along with the associated biological processes (GOs).

Supplementary Table S2. Epigenetics profiling of naïve and virus-infected human Epithelial cells (HL) and B-lymphocytes (NM). (A) Virus-inducible NGS peaks enriched in virus-infected HL compared to naïve HL (HL; $SVI^{6h/0h}$) derived from DNaseI-seq and ChIP-seq assays. **(B)** Virus-inducible NGS peaks enriched in virus-infected NM compared to naïve NM (NM; $SVI^{6h/0h}$) derived from DNaseI-seq and ChIP-seq assays. **(C)** Genomic coordinates of *in vivo* reconstituted CHs in naïve and virus-infected HL. CHs-associated vruDEGs are listed. The Upset plot shows the (co)-occupation of CHs by inducible IRF3, p65, and CBP for HL SVI 6h. **(D)** Genomic coordinates of *in vivo* reconstituted CHs in naïve and virus-infected NM. CHs-associated vruDEGs are listed. The Upset plot shows the (co)-occupation of CHs by inducible IRF3, p65, and CBP for NM SVI 6h. **(E)** A comprehensive table demonstrates the entire analyses from DNaseI-seq and ChIP-seq assays in NM, HL, A549, THP-1 and NIH/3T3, which includes numerical data for NGS peaks. **(F)** Comparisons of biological replicates of *epigenomics* assays (DNaseI-seq and ChIP-seq) in HL and NM. Additional information regarding the mostly enriched GO terms and TFBMs, and the number of associated vruDEGs are provided for the virus-inducible peaks obtained from the datasets selected. **(G)** Virus-inducible NGS peaks enriched in virus-infected A549 compared to naïve A549 (A549; $SVI^{6h/0h}$) derived from H3K27ac- and IRF3-ChIP-seq assays. **(H)** Virus-inducible NGS peaks enriched in virus-infected THP-1 compared to naïve THP-1 (THP-1; $SVI^{6h/0h}$) derived from H3K27ac-ChIP-seq assay.

Supplementary Table S3. Genomic coordinates of SEs and tEs in naïve and virus-infected human Epithelial cells (HL) and B-lymphocytes (NM), and (sub)classes of those. (A) SEs in naïve and virus-infected HL and NM (HL, NM; SVI 0h, 6h) were identified from MED1- and H3K27ac-

ChIP-seq datasets, using the ROSE algorithm. SEs classification according to their anatomical pattern of assembly [preassembled-decommissioned (pdSEs), preassembled-maintained (pmSEs), and virus-induced (viSEs)]. **(B)** SEs-associated expressed vruDEGs (C/I, C/II, C/III) in naïve and virus-infected HL and NM. **(C)** GOs of pdSEs in naïve HL and NM. **(D)** Genomic coordinates of subclasses of SEs distinguished according to their single or combined epigenetic marking in HL and NM. **(E)** tEs in naïve and virus-infected HL and NM (HL, NM; SVI 0h, 6h) identified from MED1- and H3K27ac-ChIP-seq datasets, using the ROSE algorithm. **(F)** The genomic coordinates of "newly-established" CHs and viSEs in HL and NM. The process validated the statistical significance of the overlap. **(G)** List of Super-enhancers (SEs) in naïve HeLa cells (SVI 0 hrs) that co-inhabit domains (Rao *et al.* Cell, 2014) with SEs-associated expressed vruDEGs in naïve HeLa cells (SVI 0 hrs).

Supplementary Table S4. *Massive-in-parallel in vivo* functional authentication by TFs-ChIP-STARR-seq assays uncovers SHAe in virus-infected human Epithelial cells (HL). (A) Genomic coordinates of the 1,949 IRF3-SHAe. Distribution of IRF3-SHAe in SEs, tEs, and safGs. IRF3-SHAe-associated vruDEGs (C/I, C/II, C/III) in virus-infected HL. **(B)** Genomic coordinates of the 1,601 p65-SHAe. Distribution of p65-SHAe in SEs, tEs, and safGs. p65-SHAe-associated vruDEGs (C/I, C/II, C/III) in virus-infected HL. **(C)** Same as in (A) and (B) for the 219 common IRF3/p65-SHAe. **(D)** Proximity analysis of vruDEGs to vrCRMs in virus-infected HeLa cells (SVI 6 hrs). **(E)** SHAe *in vivo* operation in additional human tissues challenged with SVI or alternative immunogenic and inflammatory stimuli.

Supplementary Table S5. Classification of 3,367 SHAe (IRF3 or/and p65) into repetitive and non-repetitive DNA; phylo*genomics* investigations; DNA Grammar and Syntax analyses; NIH/3T3 ChIP-seq experiments; vrDTTEs. (A) The repetitive-SHAe genomic coordinates and their evolutionary classification. List of repetitive-SHAe with DNA sequences conserved in viral genomes. **(B)** The non-repetitive-SHAe genomic coordinates and their evolutionary classification. **(C)** The 482 IR-HCTFBSs distribution within SEs, tEs, and safGs. **(D)** The 588 κBR-HCTFBSs distribution within SEs, tEs, and safGs. **(E)** The genomic coordinates of IR//κBR-HCTFBSs and their distribution in SEs, tEs, and safGs. Listed are 38 vruDEGs associated *in cis* with IR//κBR-HCTFBSs. **(F)** Genomic coordinates of IRHADs in the human (hg19), and the mouse genome (mm9). The number of hyper-motif instances, the closest gene, and the distance from TSSs (according to GREAT) are charted. **(G)** Virus-inducible NGS peaks captured in NIH/3T3 ($SVI^{6h/0h}$) derived from IRF3- and H3K27ac-ChIP-seq assays. Non-human-orthologous virus-inducible IRF3 peaks ($SVI^{6h/0h}$) hosting viral DNA segments. **(H)** Analyses of transposable elements families differential expression (HL; $SVI^{6h/0h}$). **(I)** Same as in (H) for NM. **(J)** Same as in (H) and (I) for individual copies of TEs in HL. The genomic distribution is charted for each DTTE copy. Assignment of vruDTTEs to vruDEGs (C/I, C/II, and C/III). **(K)** Same as in (J) for NM. **(L)** List of 68 SEs in virus-infected HL and 188 in virus-infected NM that embed at least one vruDTTE copy. **(M)** Genomic coordinates of vruDTTEs that overlap with cCREs in virus-infected HL. **(N)** Genomic coordinates of all SHAe associated with at least one vruDTTE and the specific genomic coordinates of 222 SHAe associated (±40 kb) with at least one vruDTTE. **(O)** Same as in (J)

for MRC-5. **(P)** Same as in (J) for NIH/3T3. **(Q)** Comprehensive scanning reveals the identification of SNPs associated with Human Autoimmune Diseases across genomic loci of SEs, and SHAe. **(R)** High-resolution depiction of representative examples illustrating the evolutionary history of SHAe from the classes of e-Primates, e-Mammals, and b-Mammals within the cohort 241 placental mammals. **(S)** Lift-over analysis of SHAe coordinates from GRCh37/hg19 to GRCh38/hg38 assembly. **(T)** Intersections with Aracena *et al.* 2024 and GOs. **(U)** Comparative intersection of the SHAe, cCREs, and SNPs/QTLs cohorts and GOs. **(V)** SEs that host CHs in naïve and virus-infected HL and NM.

Supplementary Table S6. List of materials, consumables and key resources used in the study.

Supplementary Figure S1 Common virus-upregulated DEGs

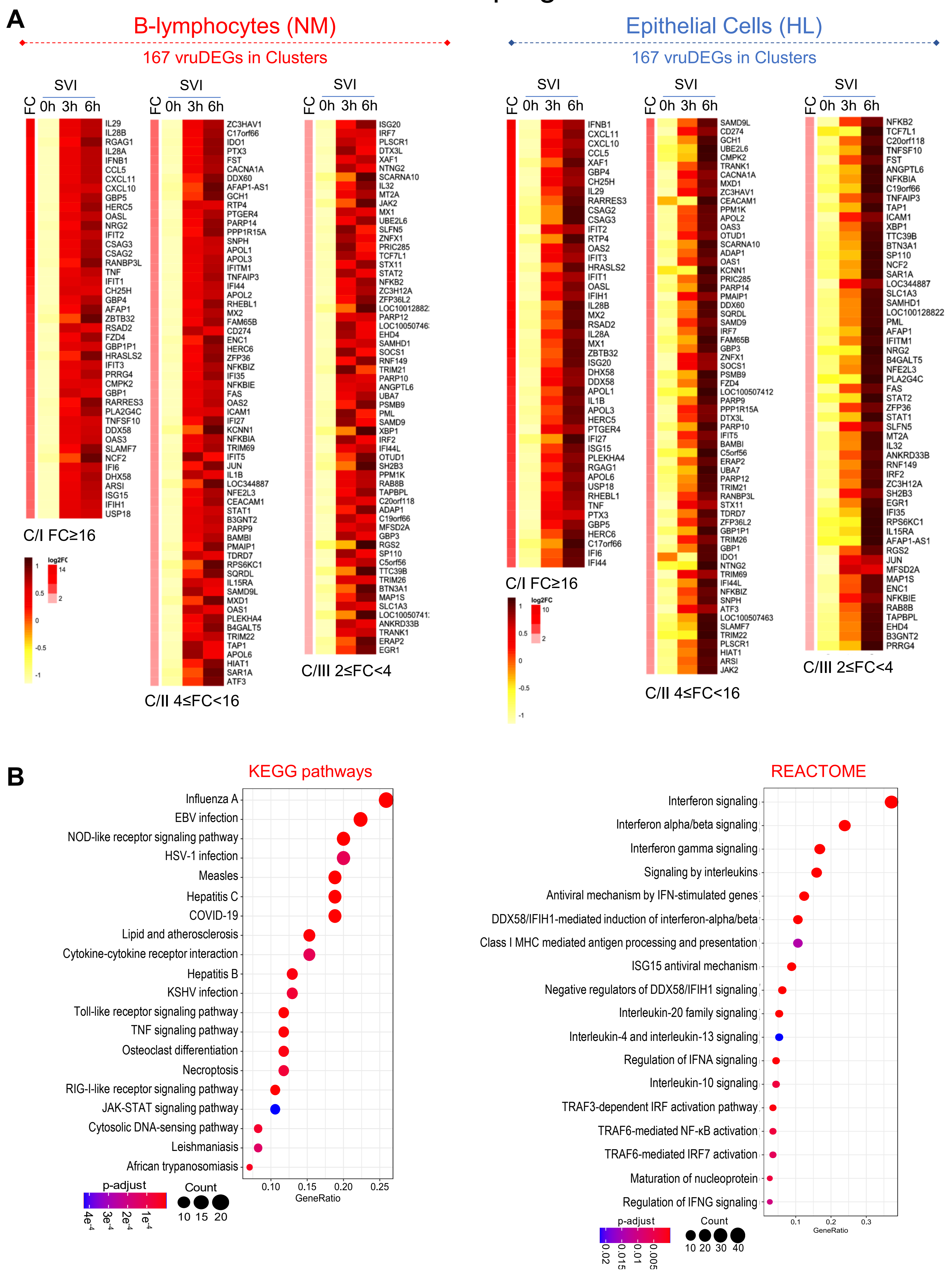

Supplementary Figure S2

Cell-type-specific virus-upregulated DEGs

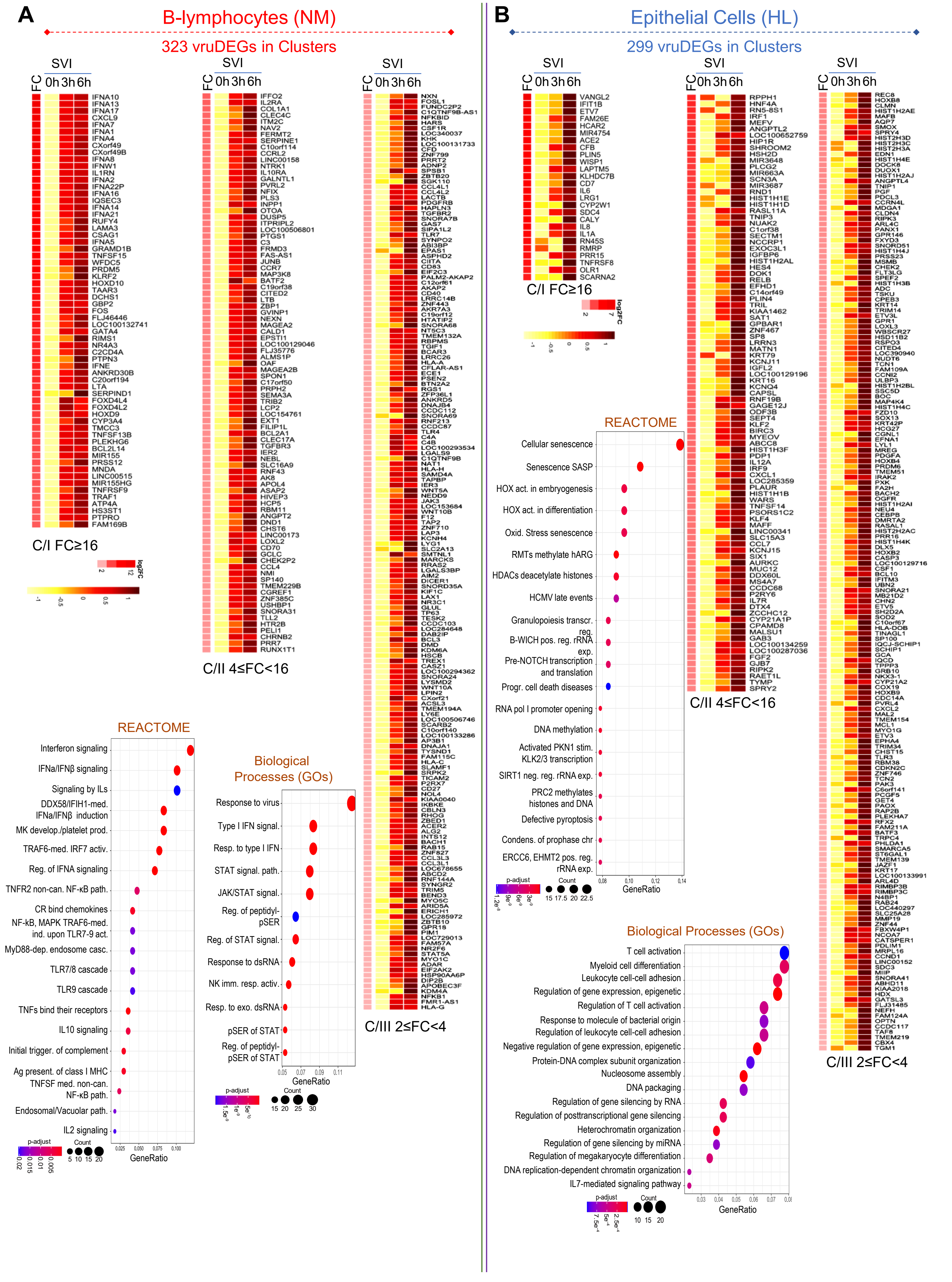

Supplementary Figure S3 Cell-type-specific virus-upregulated DEGs

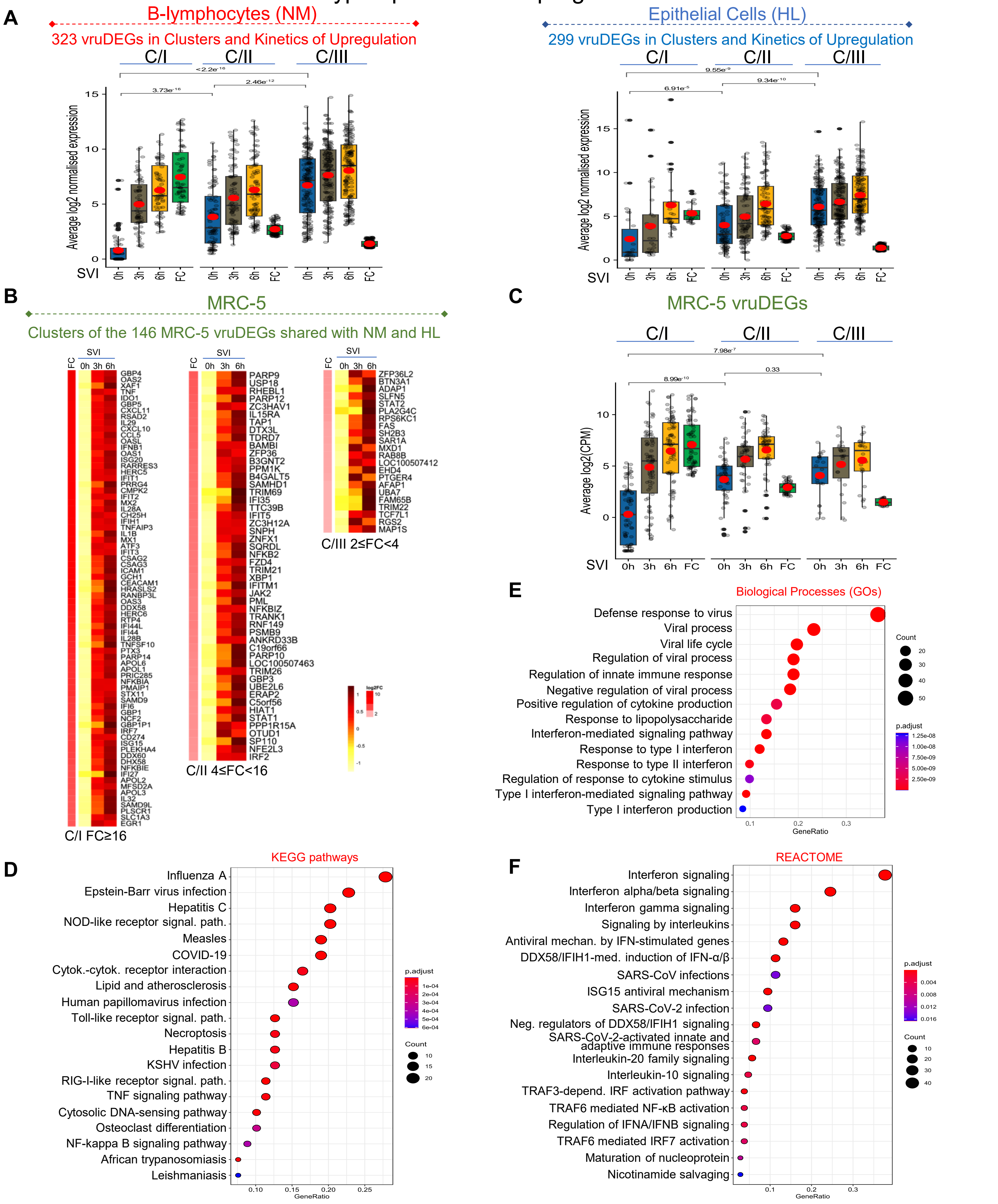

G

HeLa SV			Namalwa SV				Common	
IFI6	*ISG15*	*STAT1*	*OAS2*	*TRIM5*	*PML*	*IFIH1*	*IFI6*	*TAP1*
TRIM21	*IFIT1*	*HERC6*	*IFI6*	*APOBEC3F*	*IFITM1*	*DDX58*	*TRIM21*	*DDX58*
TRIM26	*TAP1*	*IRF9*	*TRIM21*	*STAT1*	*JAK3*		*TRIM26*	*STAT1*
SAMHD1	*DDX58*	*IRF1*	*TRIM26*	*HERC6*	*TREX1*		*SAMHD1*	*HERC6*
PML	*CASP3*	*TRIM34*	*TRIM22*	*XAF1*	*IFIT1*		*PML*	*ISG15*
IFITM1	*TRIM14*	*IFITM3*	*SAMHD1*	*ISG15*	*TAP1*		*IFITM1*	*IFIT1*

Human MRC-5 SV			
STAT1	*TRIM21*	*TRIM26*	*APOBEC3F*
IFITM3	*IRF9*	*IFITM1*	*HERC6*
TRIM22	*TRIM14*	*SAMHD1*	*TREX1*
PML	*IRF1*	*TRIM5*	
TAP1	*IFI6*	*DDX58*	
ISG15	*IFIT1*	*IFIH1*	

Supplementary Figure S4

A

	Human MRC-5 SV	Human Calu-3 SARS-CoV-2	Human A549 IAV	Human A549 RSV	Human A549 DENV2	Human A549 HPIV3
Total of NM+HL Unique vruDEGs	353	312	226	288	186	237
NM SVI 6h	240	216	157	201	120	170
HL SVI 6h	259	225	168	212	160	189

	Human Respiratory Tissues EV-D68	HEK293 RVFV	Human CD4+ T EBOV	Human Airway Organoids SARS-CoV-2	Human Brain Organoids ZIKV	Human Macrophages WNV
Total of NM+HL Unique vruDEGs	229	298	277	337	334	399
NM SVI 6h	157	202	200	242	218	290
HL SVI 6h	172	210	182	233	224	250

Human Calu-3 SARS-CoV-2		Human A549 IAV		Human A549 RSV		Human A549 DENV2	Human A549 HPIV3		Human Respirt. Tissues EV-D68		HEK293 RVFV		Human CD4+ T EBOV		Human Airway Organoids SARS-CoV-2		Human Brain Organoids ZIKV		Human Macrophages WNV	
IFI6	IFIT1	STAT1	TRIM5	STAT1	IFITM3	STAT1	STAT1	OAS2	IFIT1	IRF1	STAT1	IFI6	XAF1	TRIM21	STAT1	IFITM3	STAT1	IFIH1	IFI6	TRIM21
STAT1	IRF9	IRF1	TRIM14	TRIM14		TRIM5	TRIM21		ISG15	PML	IFITM1	ISG15	IFI6	IFITM3	IFI6	IFIH1	TRIM5	IRF1	STAT1	IRF9
IFITM3	SAMHD1	TRIM21	IRF9	TRIM21		TRIM21	PML		TAP1	TRIM14	DDX58		STAT1	TREX1	IFIT1	TRIM22	IFI6	TRIM22	TRIM14	TRIM22
ISG15	IRF1	SAMHD1	DDX58	IRF9		IRF1	IRF9		DDX58	XAF1	IFIT1		TRIM22	TRIM5	DDX58	HERC6	TRIM21	JAK3	PML	IRF1
PML	OAS2	PML	IFITM3	DDX58		IFITM3	DDX58		IFIH1	TRIM21	IRF1		OAS2	TAP1	TRIM5	SAMHD1	IFITM3		CASP3	OAS2
DDX58	TRIM5	APOBEC3F	HERC6	HERC6		APOBEC3F	HERC6		OAS2	TRIM5	IRF9		IFITM1	IRF1	TRIM21	PML	ISG15		ISG15	IFITM3
IFIH1	TRIM21	IFI6		TRIM5		IRF9	TRIM14		STAT1	TRIM22	TREX1		PML	TRIM14	IRF1	TRIM14	TRIM14		IFIH1	XAF1
TRIM14	HERC6	IFIH1		PML		HERC6	TRIM5		HERC6		SAMHD1		DDX58	IRF9	OAS2	IFITM1	HERC6		DDX58	TRIM5
APOBEC3F		ISG15		IRF1		DDX58	IRF1		APOBEC3F		PML		IFIH1	TRIM26	ISG15		IFIT1		HERC6	
IFITM1		IFITM1		SAMHD1		IFIT1	SAMHD1		IFITM3		TRIM21		ISG15	HERC6	XAF1		DDX58		APOBEC3F	
TREX1		IFIT1		IFI6			IFI6		IFITM1		HERC6		APOBEC3F	IFIT1	APOBEC3F		IRF9		IFIT1	

B

Biological Processes (GOs) in total vruDEGs

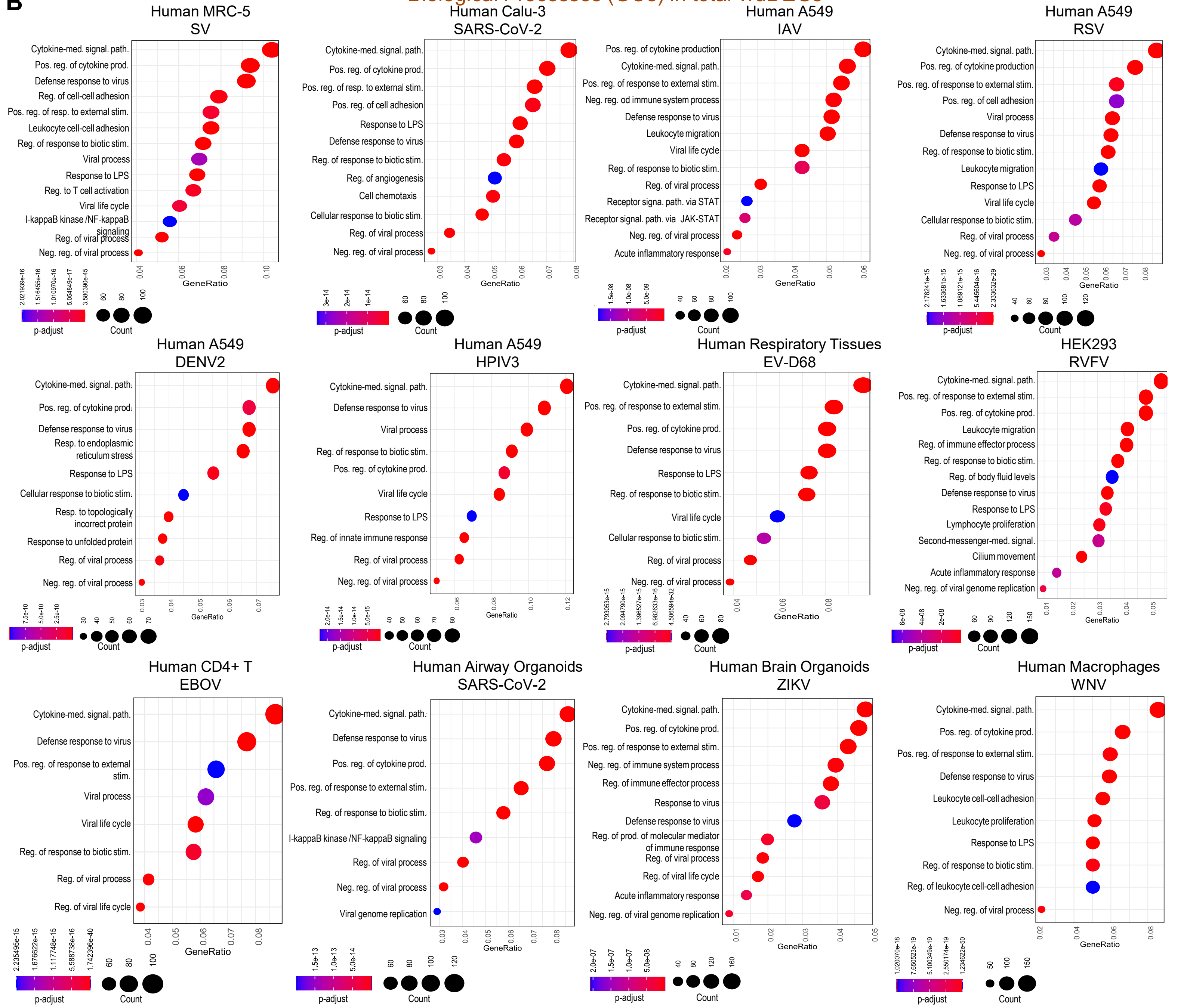

Supplementary Figure S5

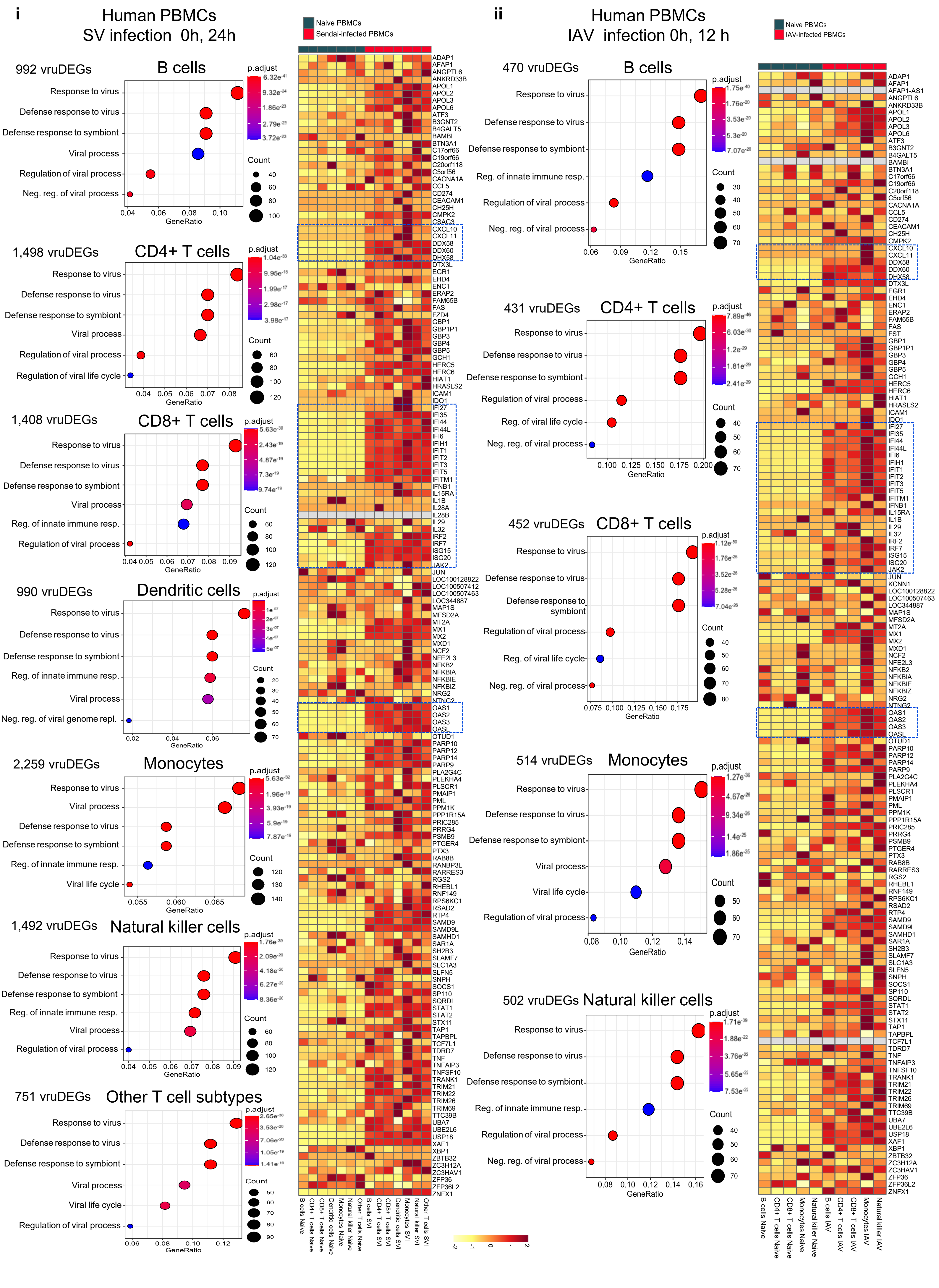

Supplementary Figure S6

A

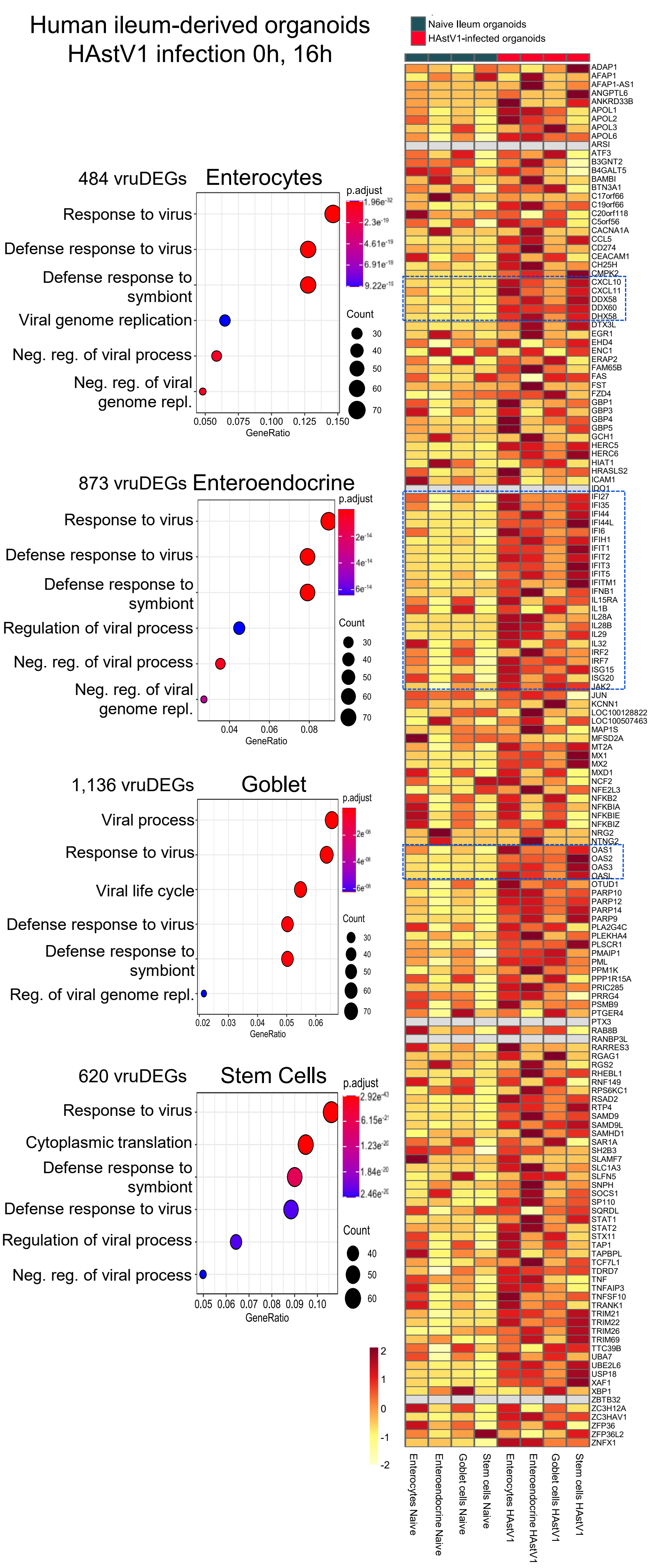

B

	Human Fibroblasts Poly(I:C)	MEFs Poly(I:C)
**Total of NM+HL Unique vruDEGs**	354	290
NM SVI 6h	234	212
HL SVI 6h	252	185

C Constitutive Immunity Molecules

**Human Fibroblasts Poly(I:C)**		**MEFs Poly(I:C)**	
IFITM3	*TRIM14*	*Samhd1*	*Xaf1*
PML	*IRF1*	*Irf1*	*Ifih1*
TRIM26	*XAF1*	*Tap1*	*Stat1*
TRIM22	*IFI6*	*Ddx58*	*Trim21*
TAP1	*TRIM21*	*Pml*	*Herc6*
SAMHD1	*ISG15*	*Trim26*	*Trim14*
TRIM5	*OAS2*	*Apobec3*	*Ifit1bl2*
IFITM1		*Irf9*	
IRF9		*Isg15*	

D Biological Processes (GOs) in total poly(I:C)-induced DEGs

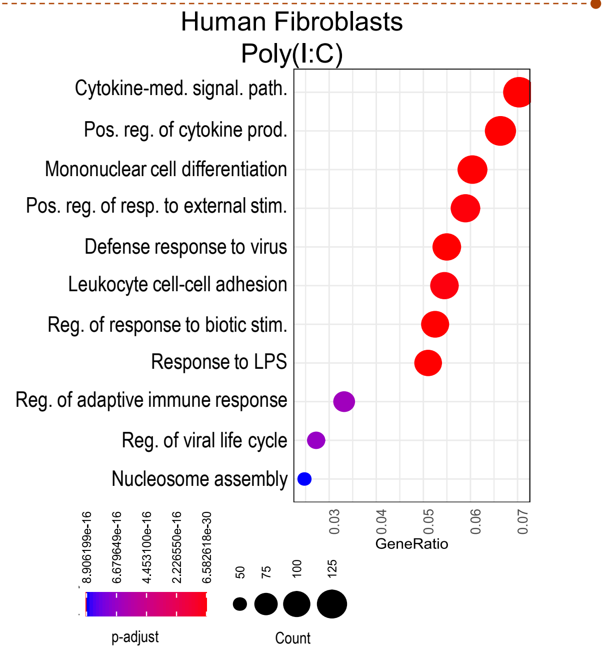

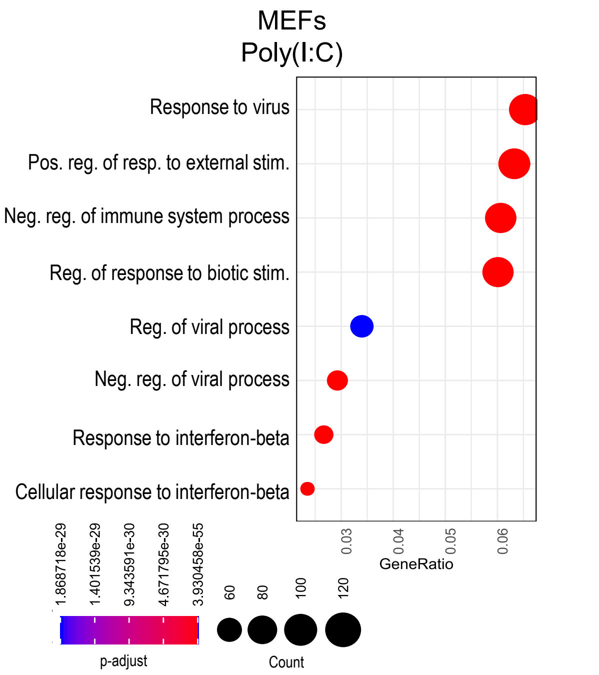

Supplementary Figure S7

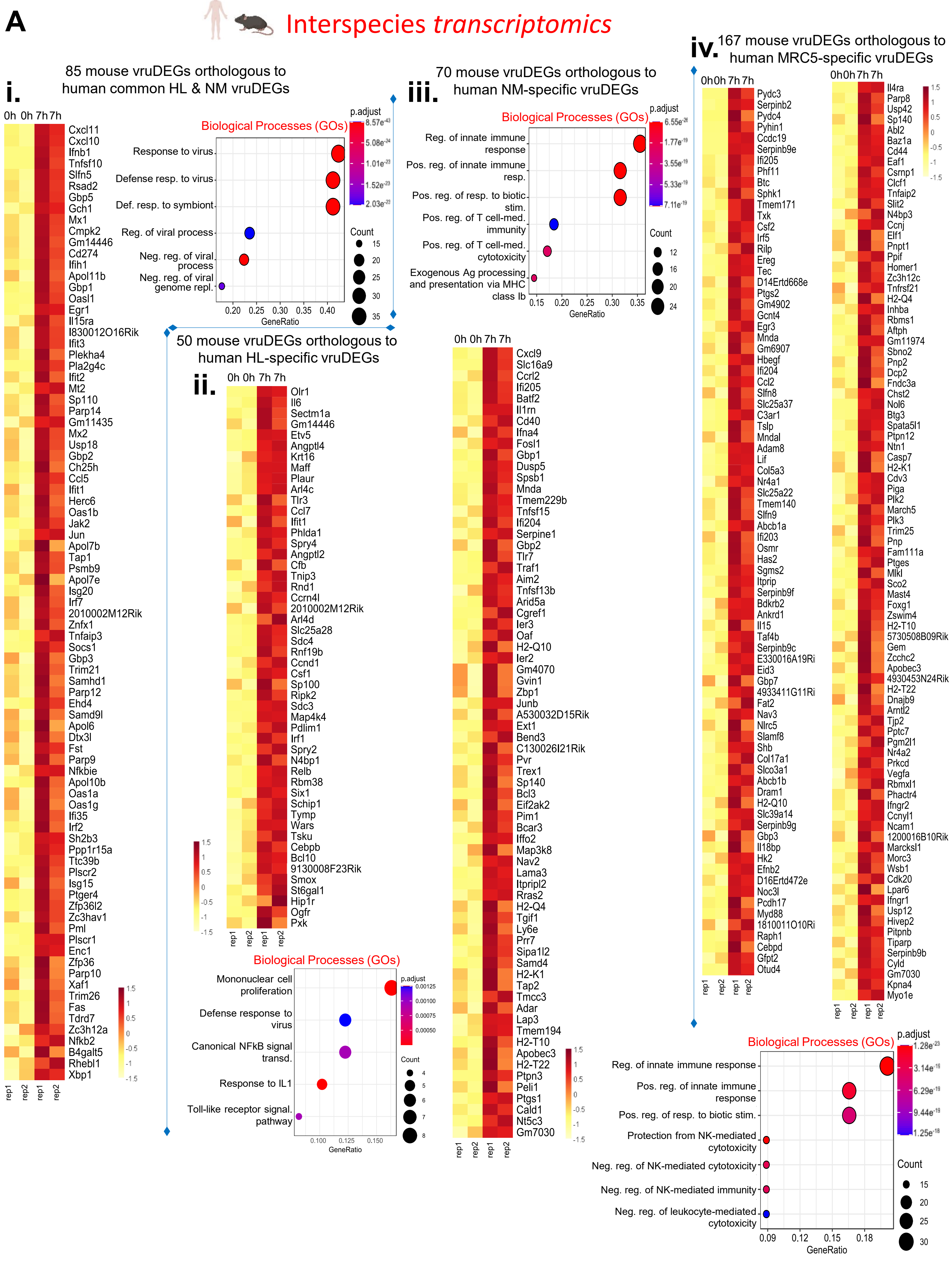

Supplementary Figure S8

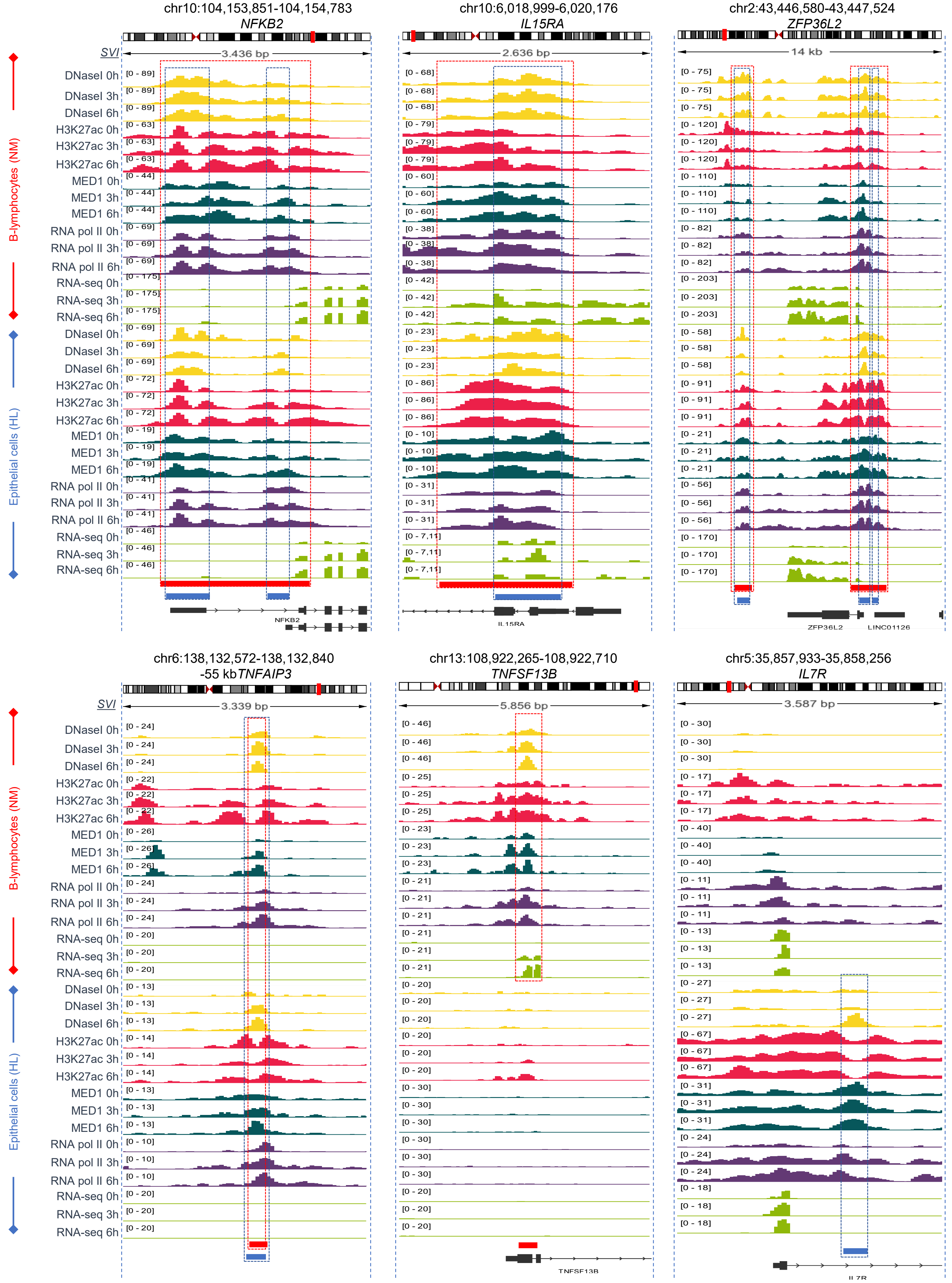

Supplementary Figure S9 *Cis*-Hotspots *in vivo* reconstitution

A

Intersections of NGS peaks	Naïve HL	Virus-infected HL	Naïve NM	Virus-infected NM
DNaseI	46,491	45,691	60,971	120,461
DNaseI/H3K27ac	32,552	30,617	22,209	30,728
DNaseI/H3K27ac/MED1	25,822	27,239	18,412	16,252
DNaseI/H3K27ac/MED1/RNA pol II *Cis*-Hotspots (CHs)	20,272	19,265	14,864	14,129
Cis-Hotspots (CHs)/IRF3	N/A	2,128	N/A	922
Cis-Hotspots (CHs)/p65	N/A	816	N/A	624
Cis-Hotspots (CHs)/CBP	N/A	1,119	N/A	1,634
Cis-Hotspots (CHs)/IRF3/p65	N/A	617	N/A	338
Cis-Hotspots (CHs)/IRF3/CBP	N/A	748	N/A	466
Cis-Hotspots (CHs)/p65/CBP	N/A	375	N/A	336
Cis-Hotspots (CHs)/IRF3/p65/CBP	N/A	364	N/A	246

B

% of CHs-Associated vruDEGs/Cluster

	Naïve HL		Virus-infected HL	
	Common	HL-specific	Common	HL-specific
C/I	38.30%	11.54%	55.32%	15.38%
C/II	53.13%	41.05%	65.63%	50.53%
C/III	71.43%	54.49%	80.36%	63.48%

% of CHs-Associated vruDEGs/Cluster

	Naïve NM		Virus-infected NM	
	Common	NM-specific	Common	NM-specific
C/I	27.91%	6.35%	41.86%	7.94%
C/II	71.43%	27.27%	73.02%	30.68%
C/III	62.30%	48.84%	59.02%	47.67%

CHs	Virus-infected HL		Virus-infected NM	
	Pre-printed CHs (SVI 0h & 6h)	Newly-established CHs (SVI only 6h)	Pre-printed CHs (SVI 0h & 6h)	Newly-established CHs (SVI only 6h)
CHs	88.19%	11.81%	89.69%	10.31%
CHs/IRF3	82.52%	17.48%	68.87%	31.13%
CHs/p65	86.76%	13.24%	73.56%	26.44%
CHs/CBP	87.31%	12.69%	85.56%	14.44%
CHs/IRF3/p65	85.41%	14.59%	67.16%	32.84%
CHs/IRF3/CBP	82.49%	17.51%	64.38%	35.62%
CHs/p65/CBP	84.80%	15.20%	69.94%	30.06%
CHs/IRF3/p65/CBP	84.34%	15.66%	65.85%	34.15%

Ci

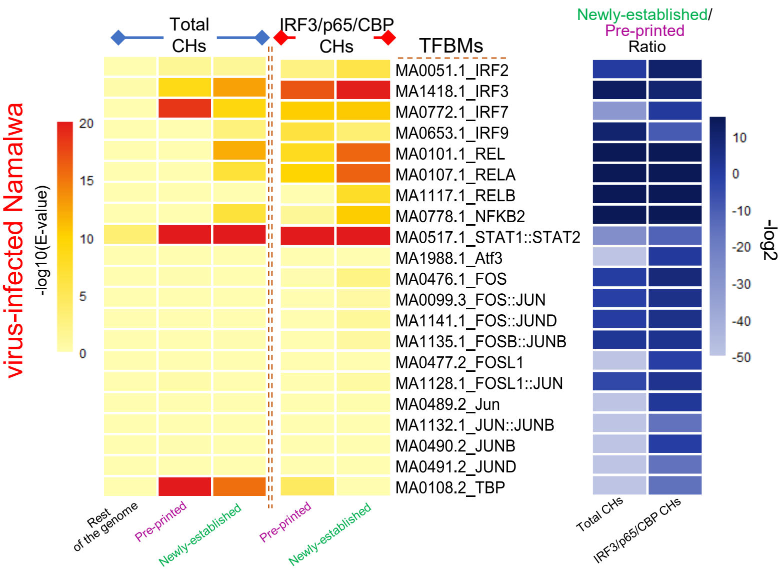

ii

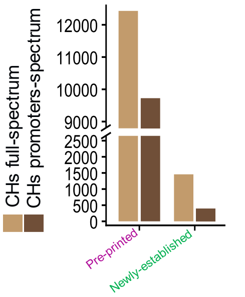

iii

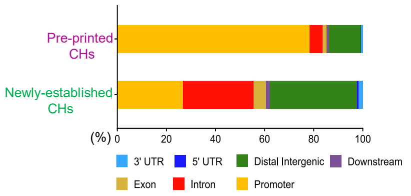

D

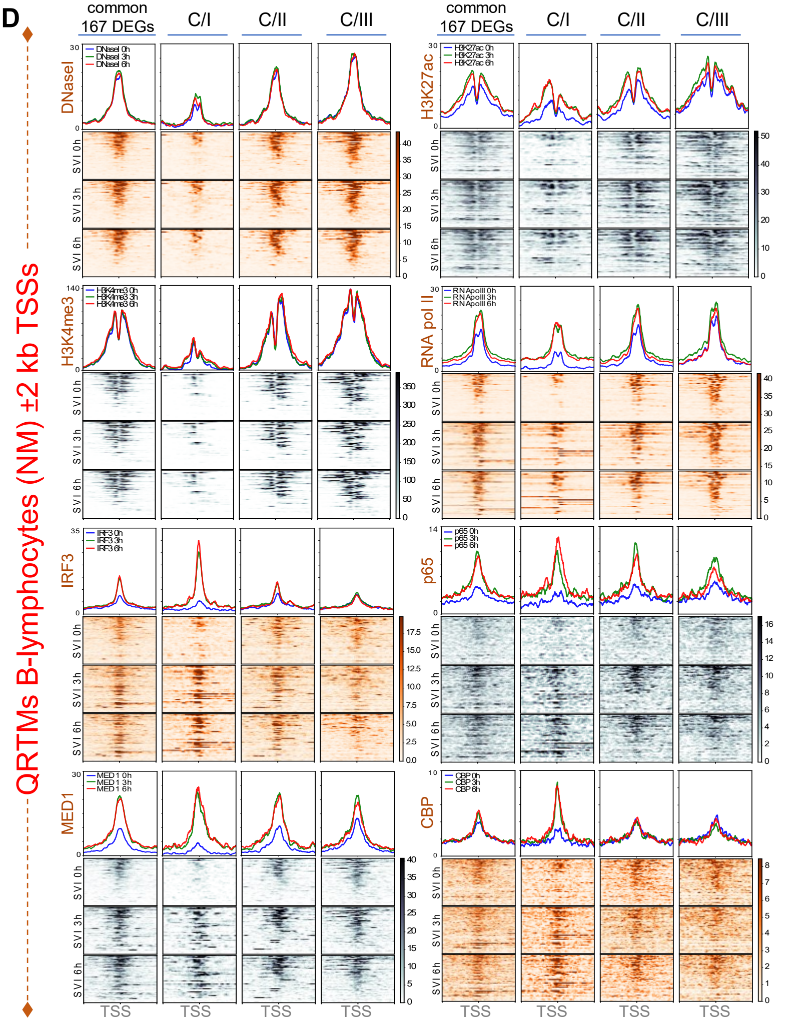

Supplementary Figure S10

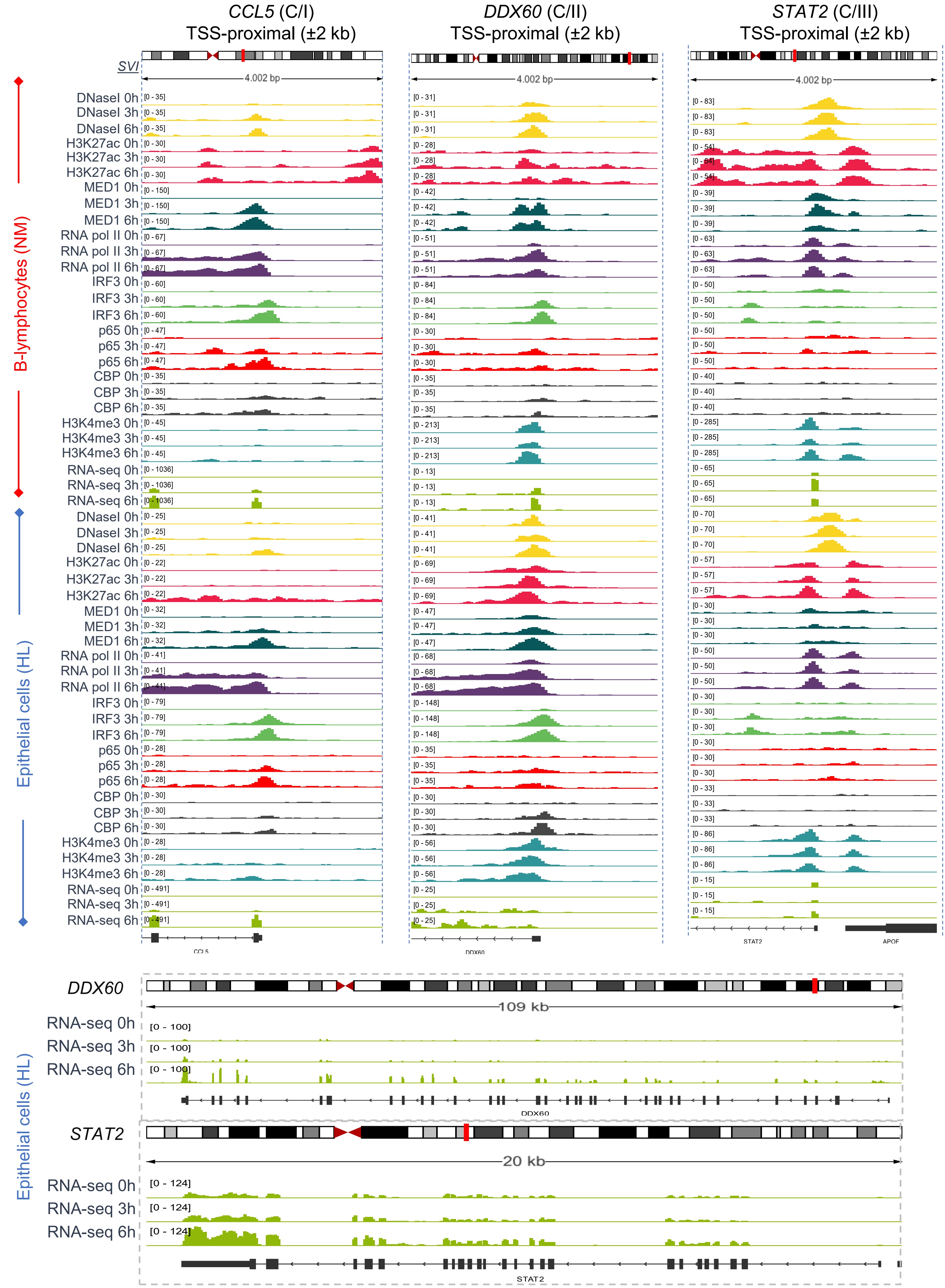

Supplementary Figure S11

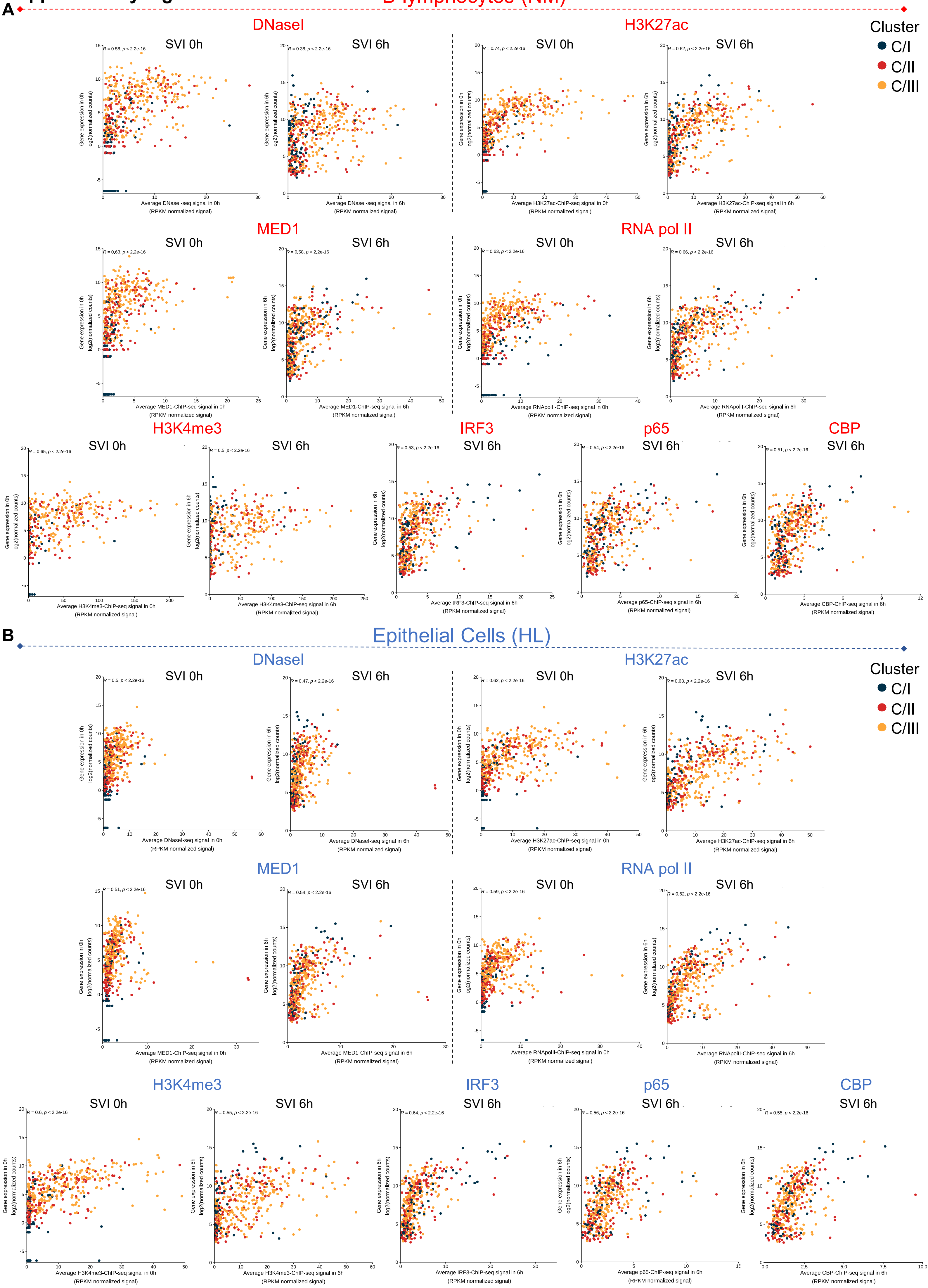

Supplementary Figure S12

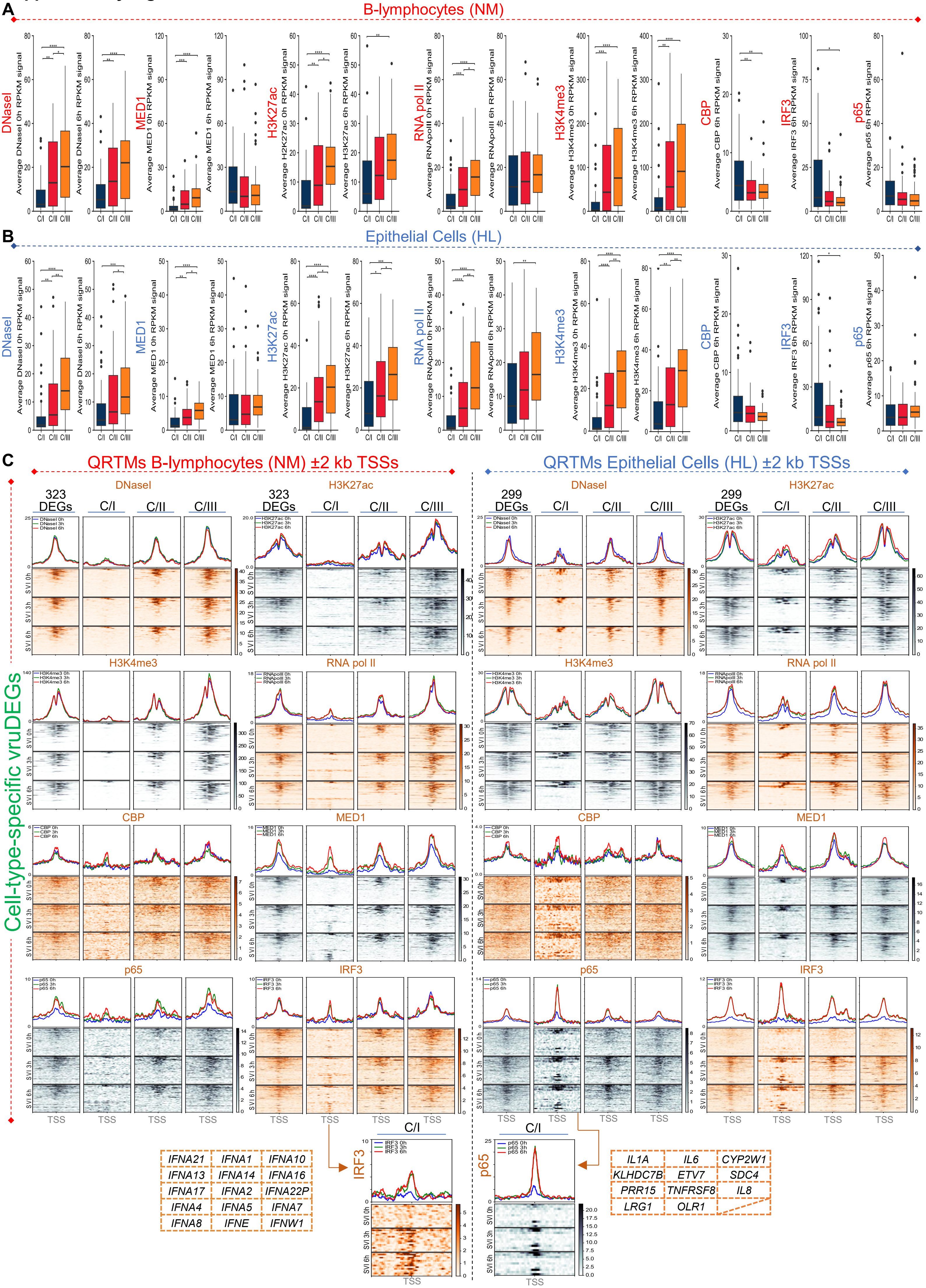

Supplementary Figure S13

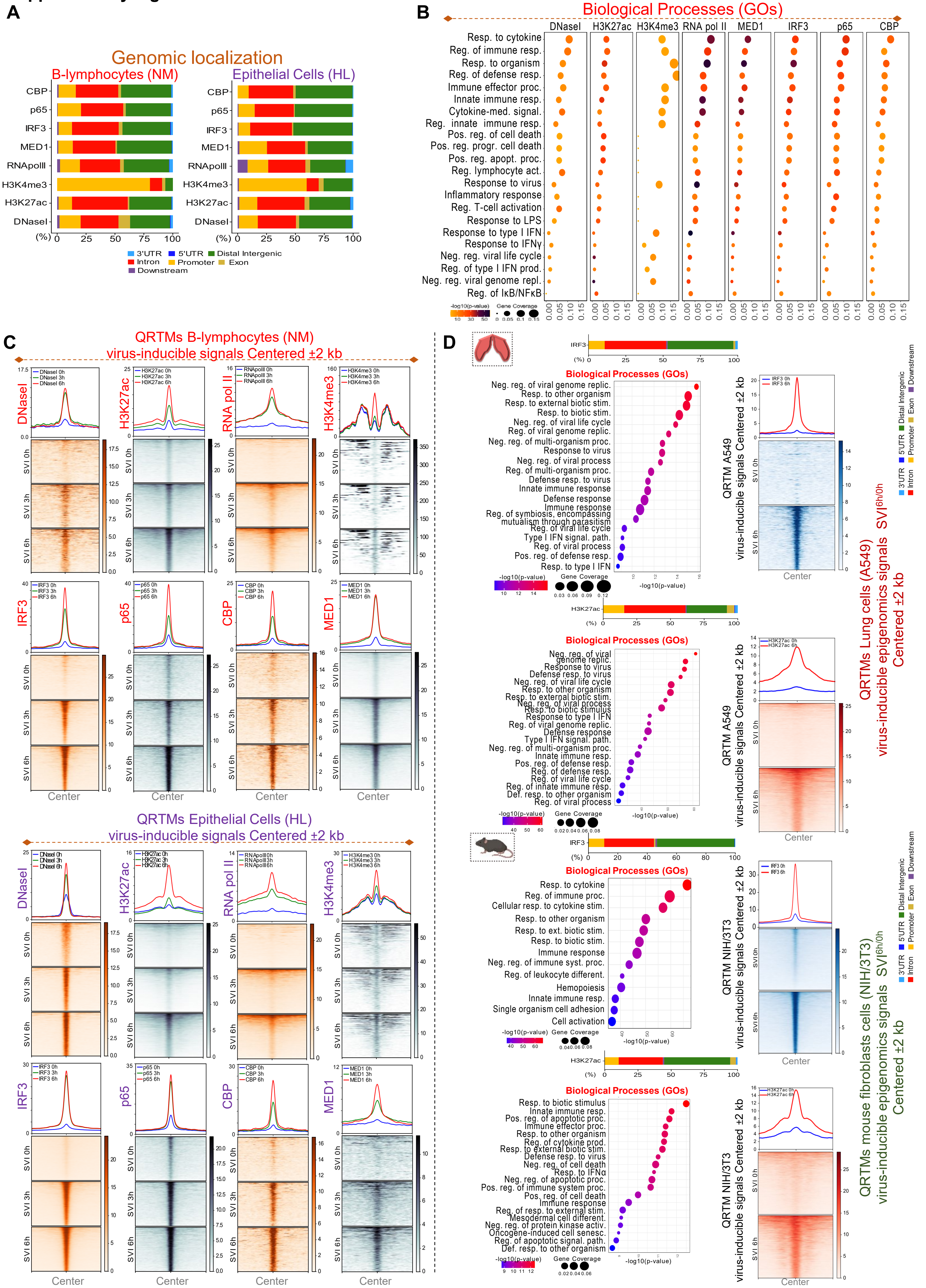

Supplementary Figure S14

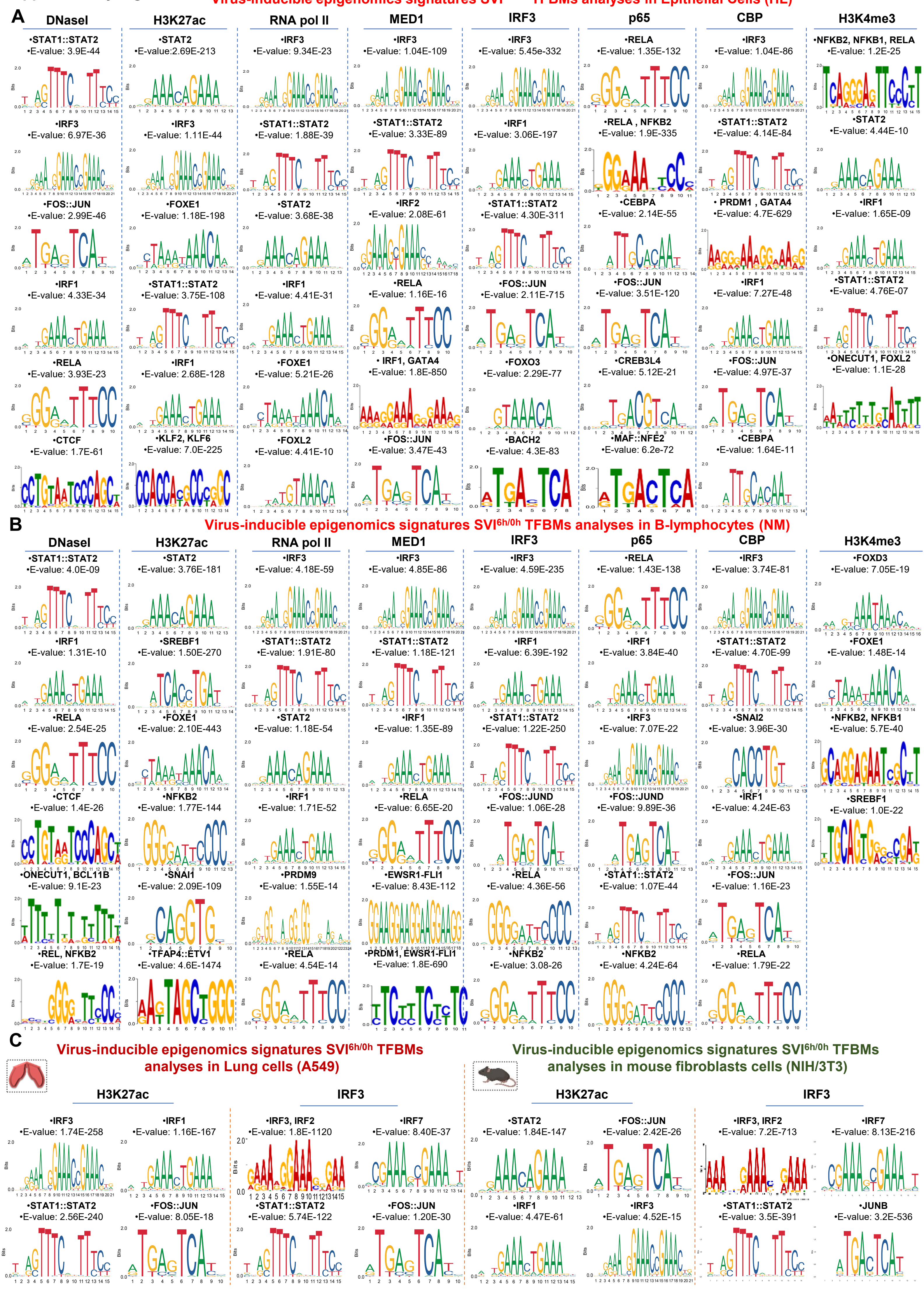

Supplementary Figure S15

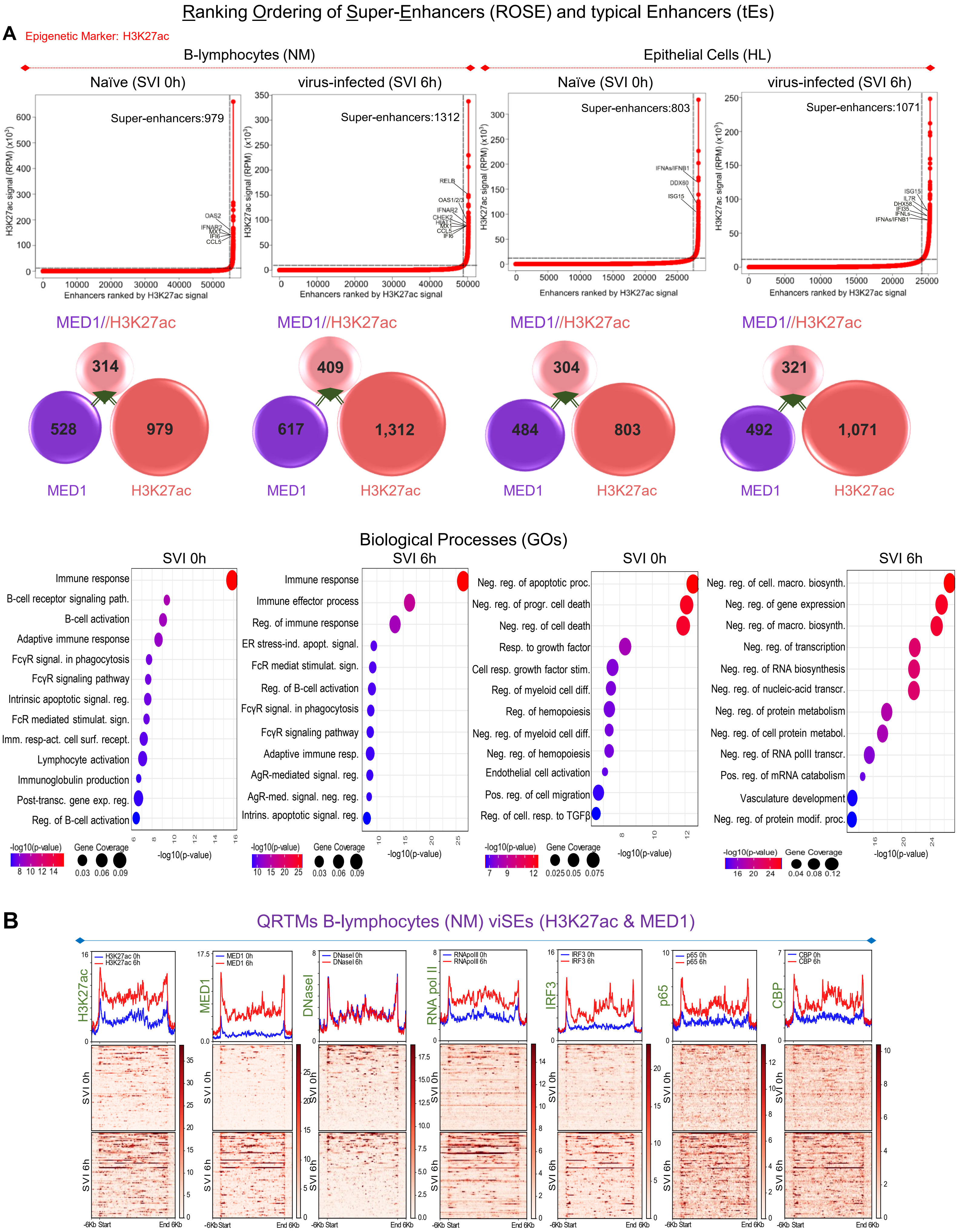

Supplementary Figure S16

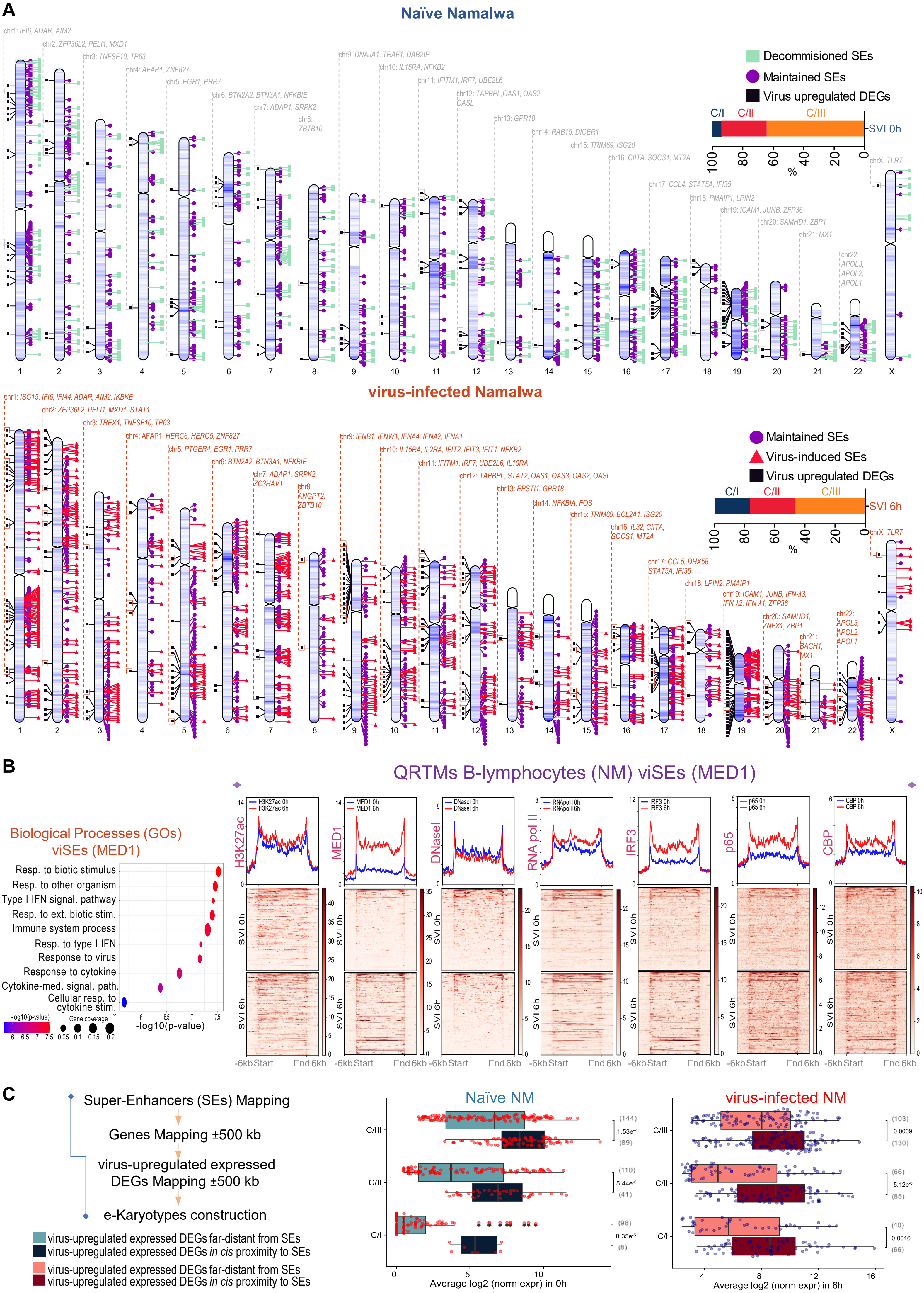

Supplementary Figure S17

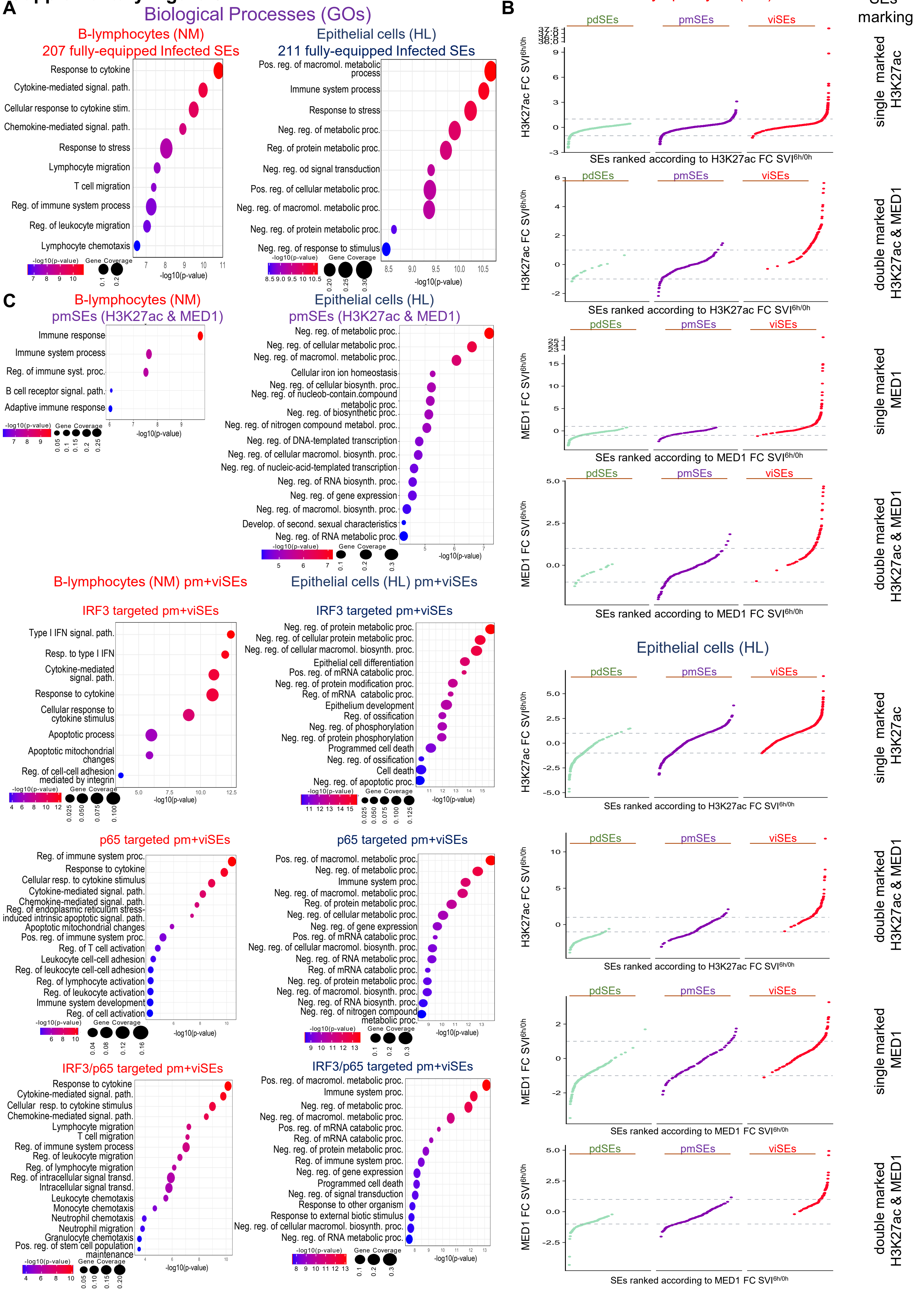

Supplementary Figure S18

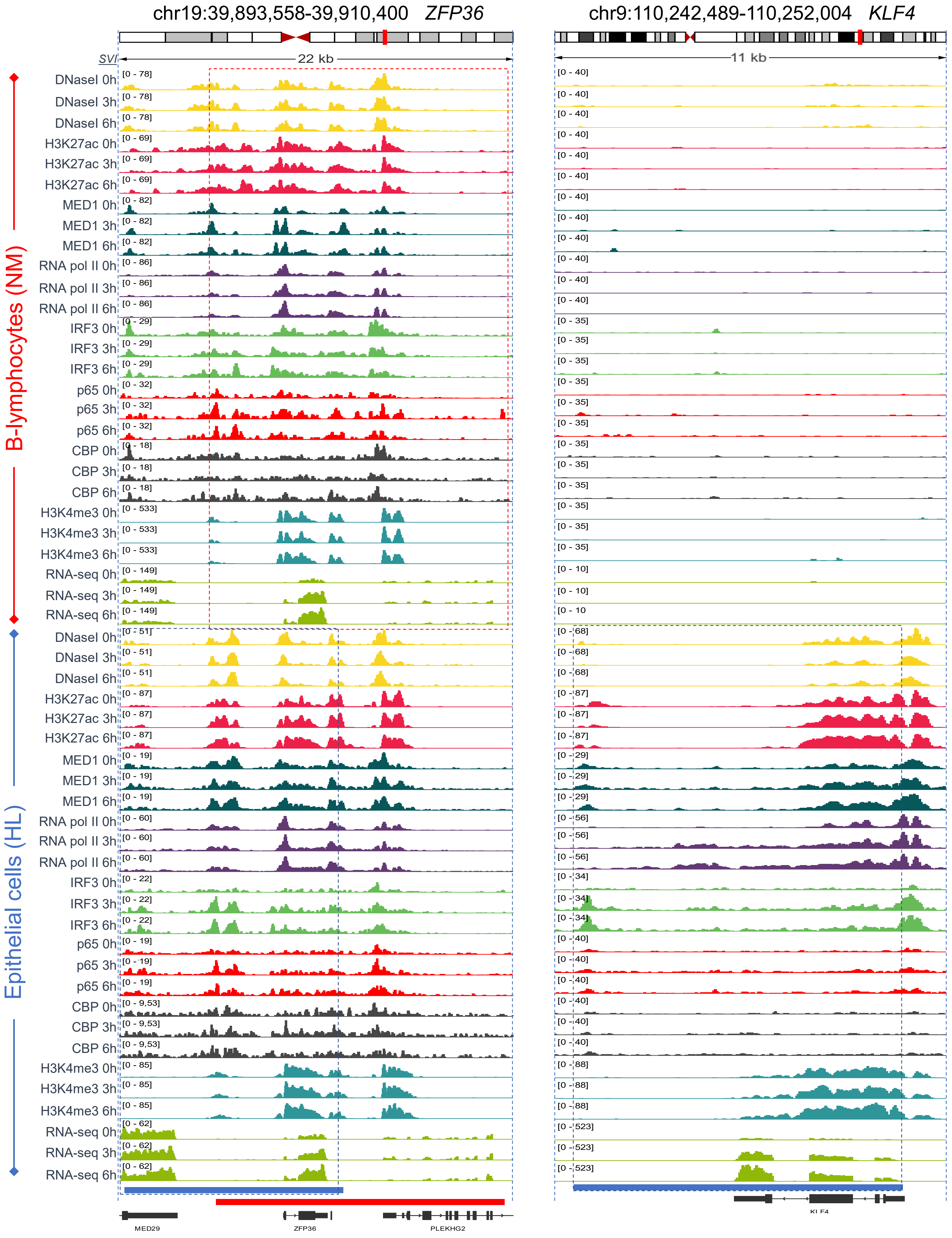

Supplementary Figure S19

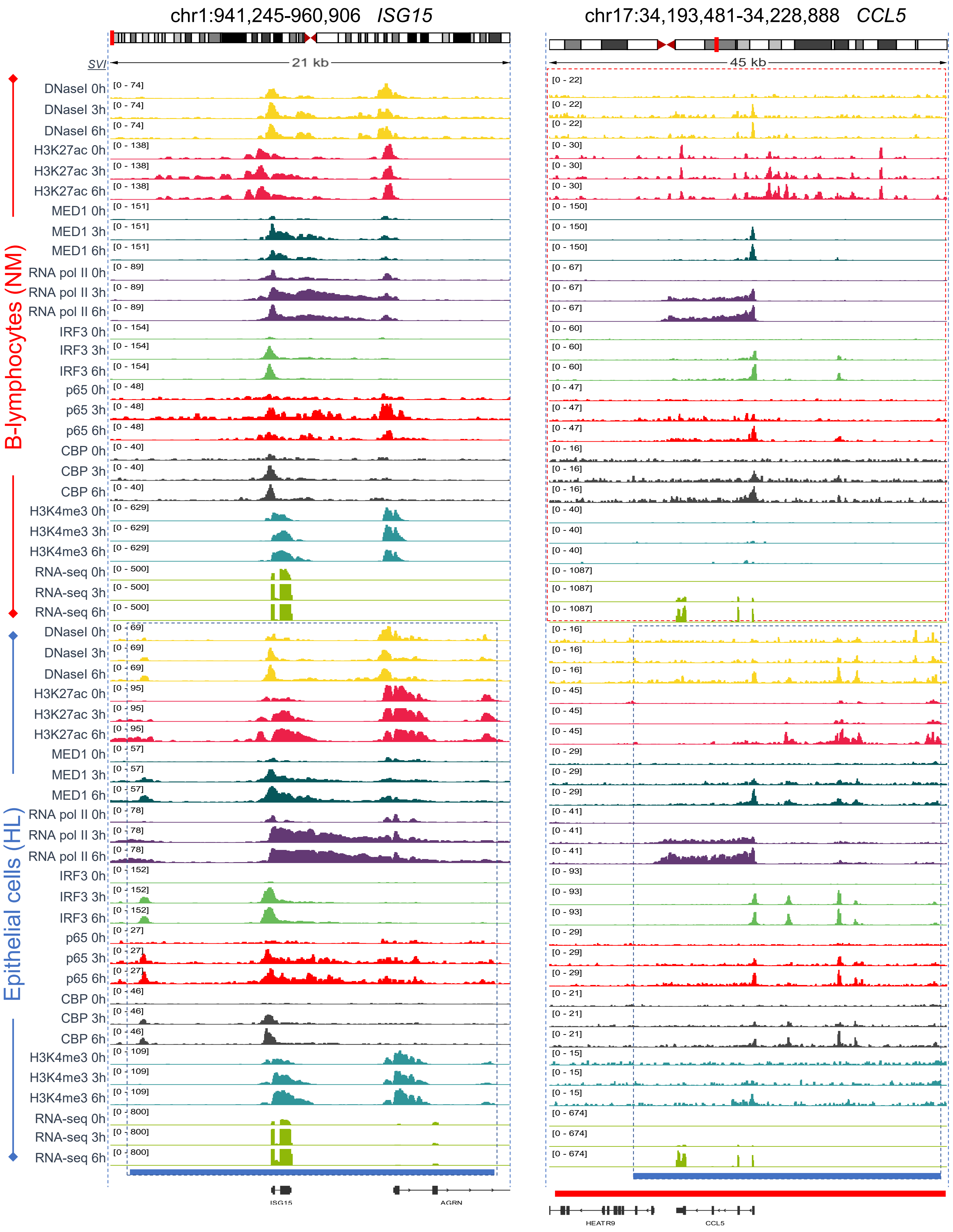

Supplementary Figure S20

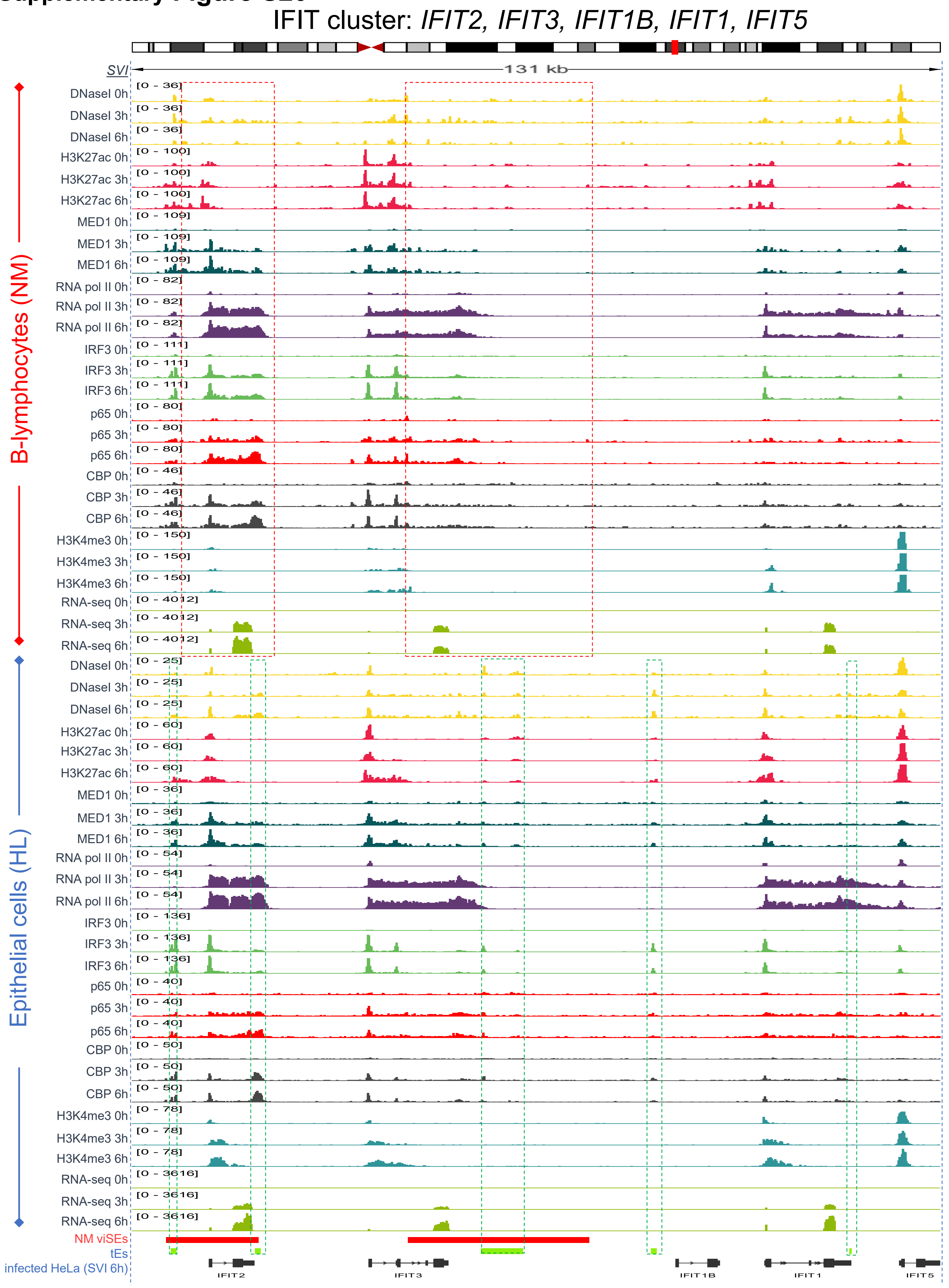

Supplementary Figure S21

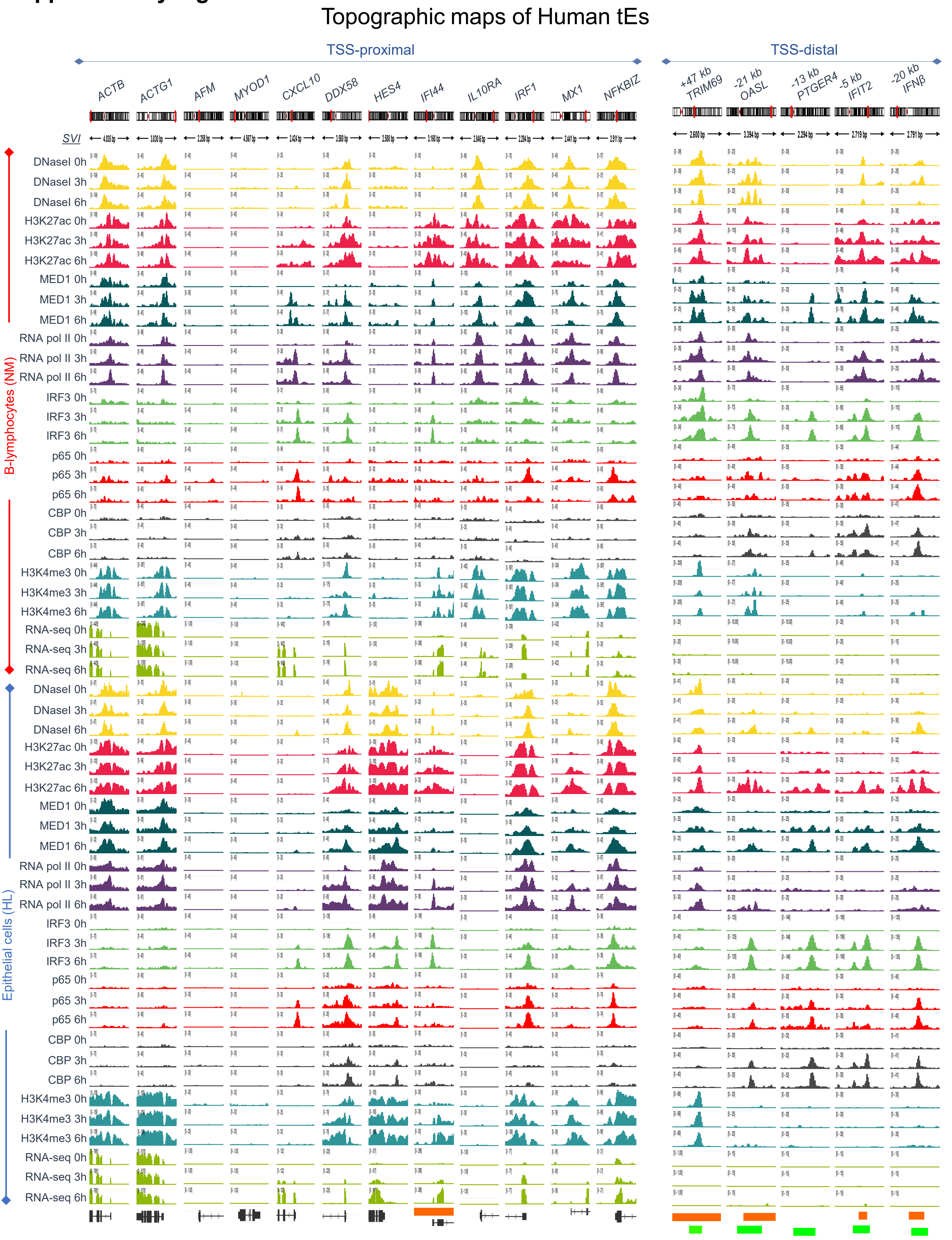

Supplementary Figure S22

A

		% of virus-upregulated DEGs/Cluster	Biological Process / Pathway	SEs-Associated DEGs
Naïve HL	C/I	12.32%	Apoptotic signaling pathway	*RELA, TP53, CFLAR, MNT, HIF1A, TNFAIP3, BCL3, TRIM32*
	C/II	25.15%	Positive regulation of viral process	*IFIT1, LARP1, CD74, VAPA*
	C/III	26.06%	Cellular Senescence	*CDKN2A/B, CDK6, ETS2, MAPKs, JUN*
Virus-induced HL	C/I	41.09%	Viral life cycle	*CCL5, ISG15, ZC3H12A, IFITM1/2/3, MX1, LY6E, IFNL3*
	C/II	52.2%	Intrinsic apoptotic pathway	*BCL3, CHEK2, IKBKE, CEBPB, PHLDA3, PMAIP1*
	C/III	52.56%	Response to virus	*ZC3H12A, ZC3HAV1, IL12A, IFNE, IFNGR1, IFNL2/3, IL6, TRIM5/11/56, STING1, STAT2, IRF7*
Naïve NM	C/I	7.54%	Activation and regulation of innate immune response	*RBM14, PLCG2, NLRC5, IFI16/35, RELA/B, AIM2, TRAFD1, CREBBP, ICAM2/3, IFNGR2, IFNAR1/2, IRF1/3/7, STAT5B*
	C/II	27.15%	Intrinsic apoptotic signaling pathway	*ENO1, NFE2L2, PARP1, BMF, MDM2, TP53, PMAIP1, HERPUD1*
	C/III	38.19%	Signaling by the B Cell Receptor	*BLNK, BLK, CD19, CD79A/B, LYN, SOS1, CARD11, PIK3CD, RELA, IKBKG, PRKCB*
Virus-induced NM	C/I	62.26%	Response to virus	*AIM2, CCL5, CXCR4, DHX58, FOSL1, FOXP3, HERC5, IFI6/16/44/44L, IFIT1/2/3/5, IFITM1/2/3, IFNAs, IFNAR1/2, IFNB1, IFNGR1/2, IFNLs, IRF1/3/5/7, ISG15, OAS1/2/3, OASL, RELA, STAT1/2*
	C/II	56.29%	Type I IFN signaling	*IFI6/35, IFIT1/2/3, IFITM1/2/3, IFNAs, IFNB1, IKBKE, IRF1/3/4/5/7/8, ISG15, JAK1, MX1, OAS1/2/3, OASL, STAT1/2, TYK2, SAMHD1, USP18*
	C/III	55.79%		

B

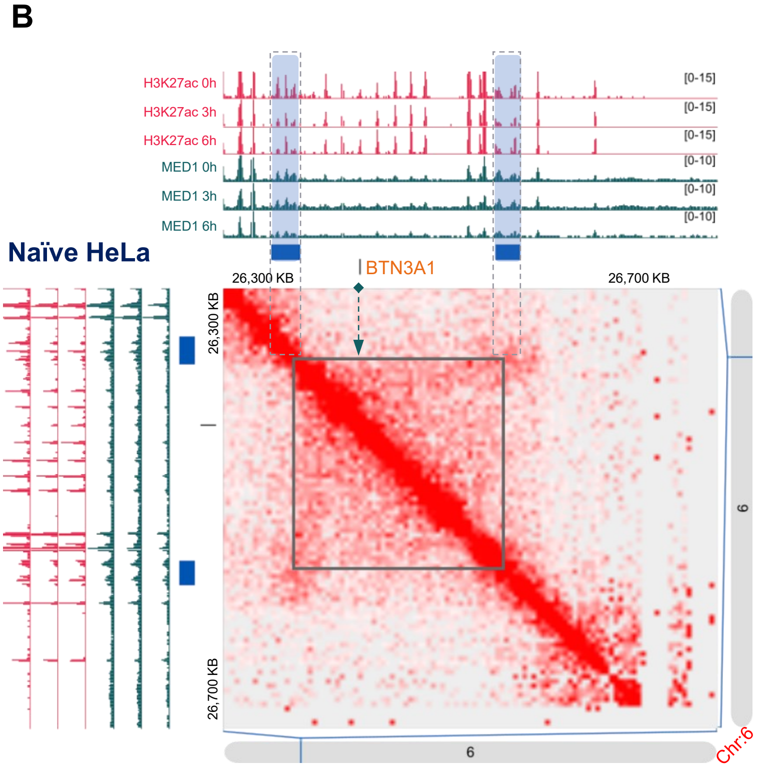

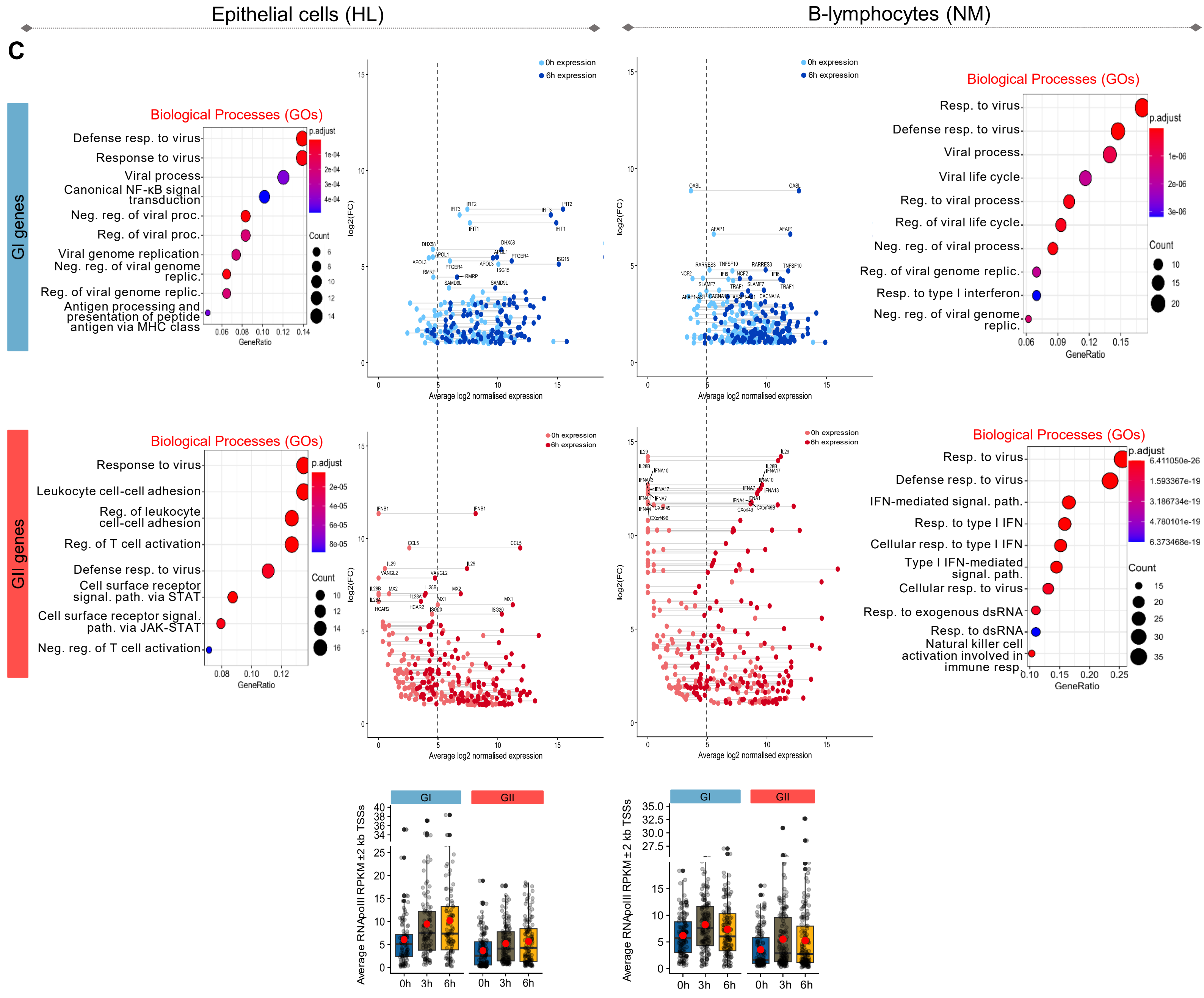

Supplementary Figure S23

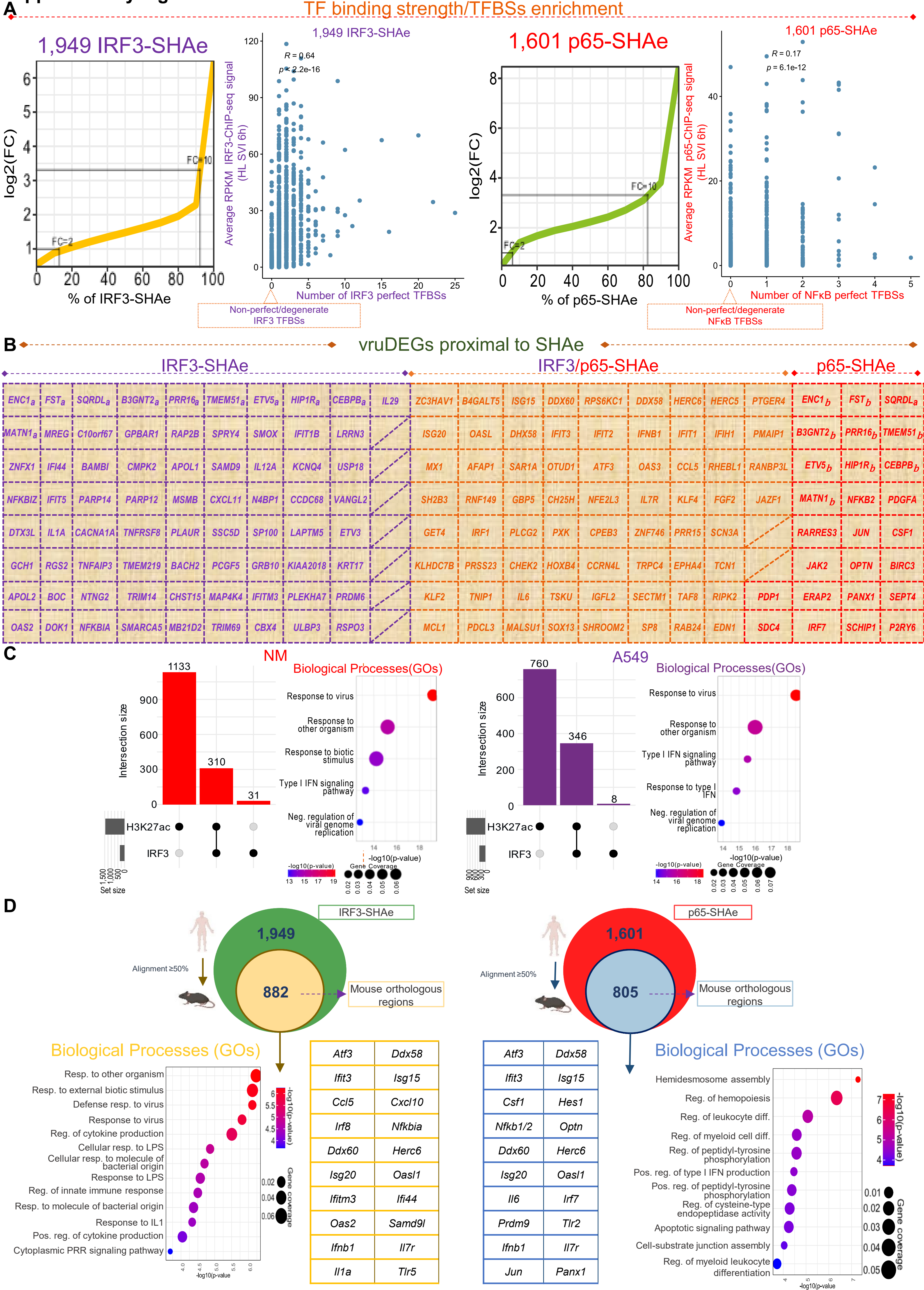

Supplementary Figure S24

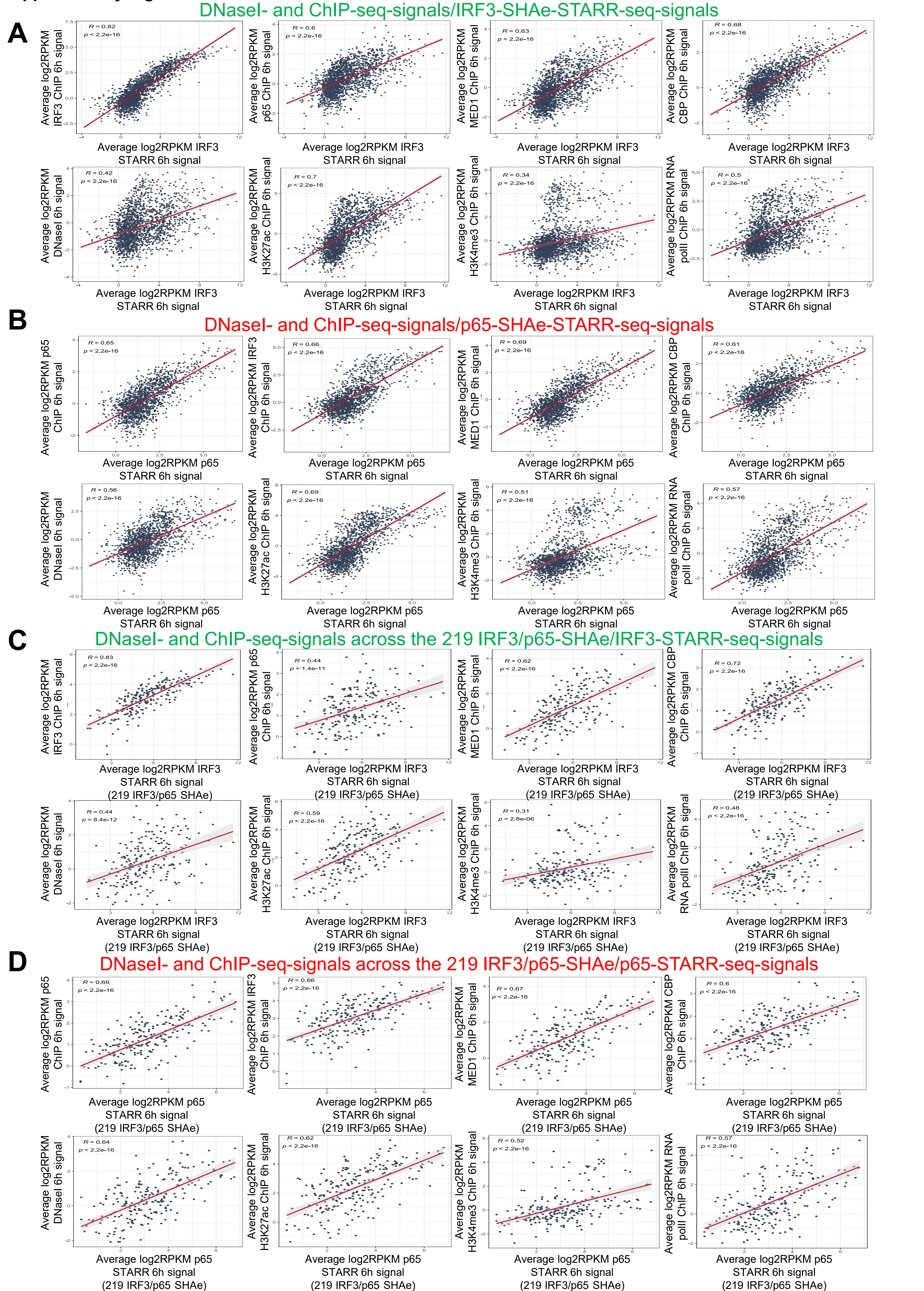

Supplementary Figure S25

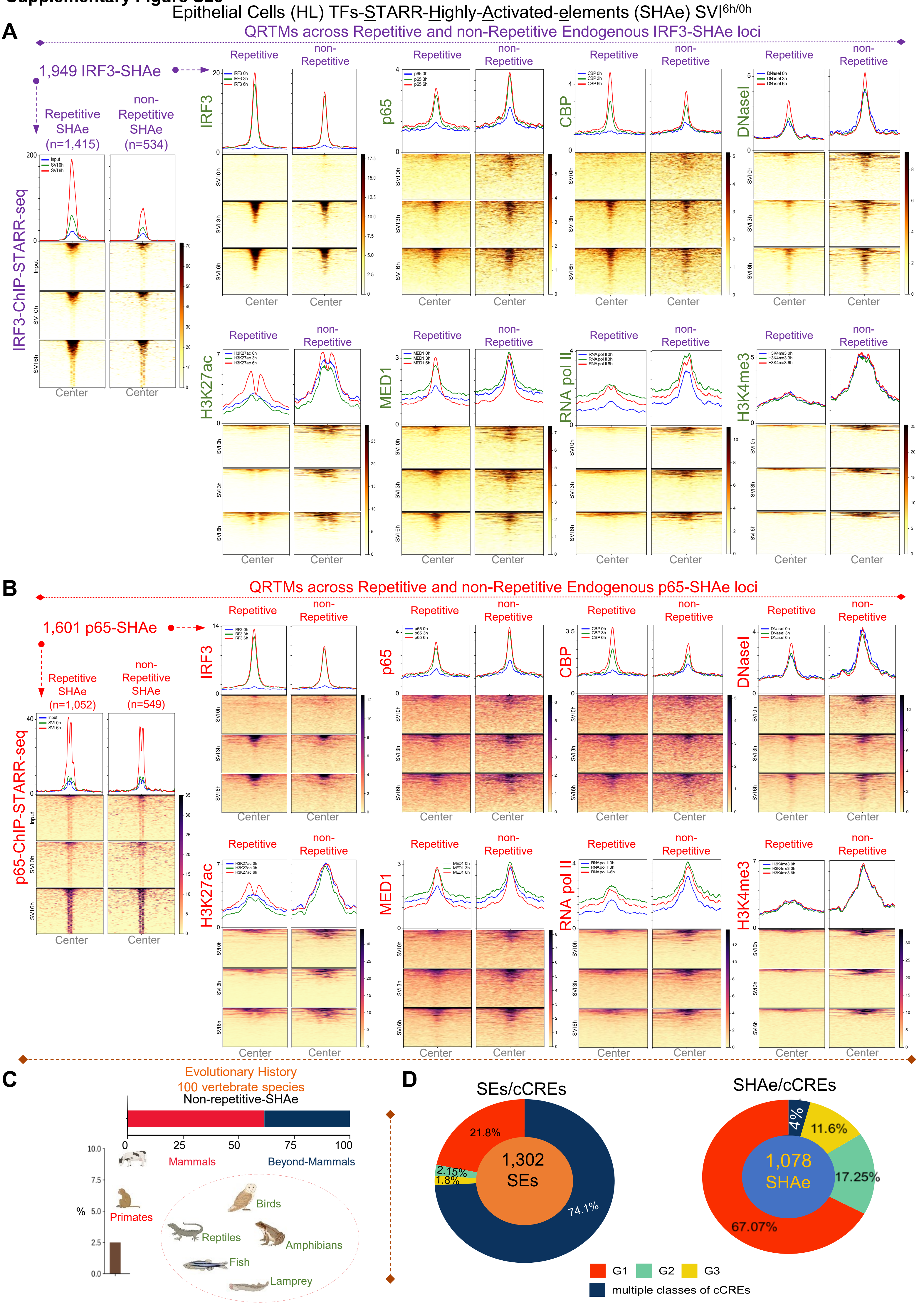

Supplementary Figure S26

A Human-Higher-Primates-enriched (HHPe-primates) SHAe

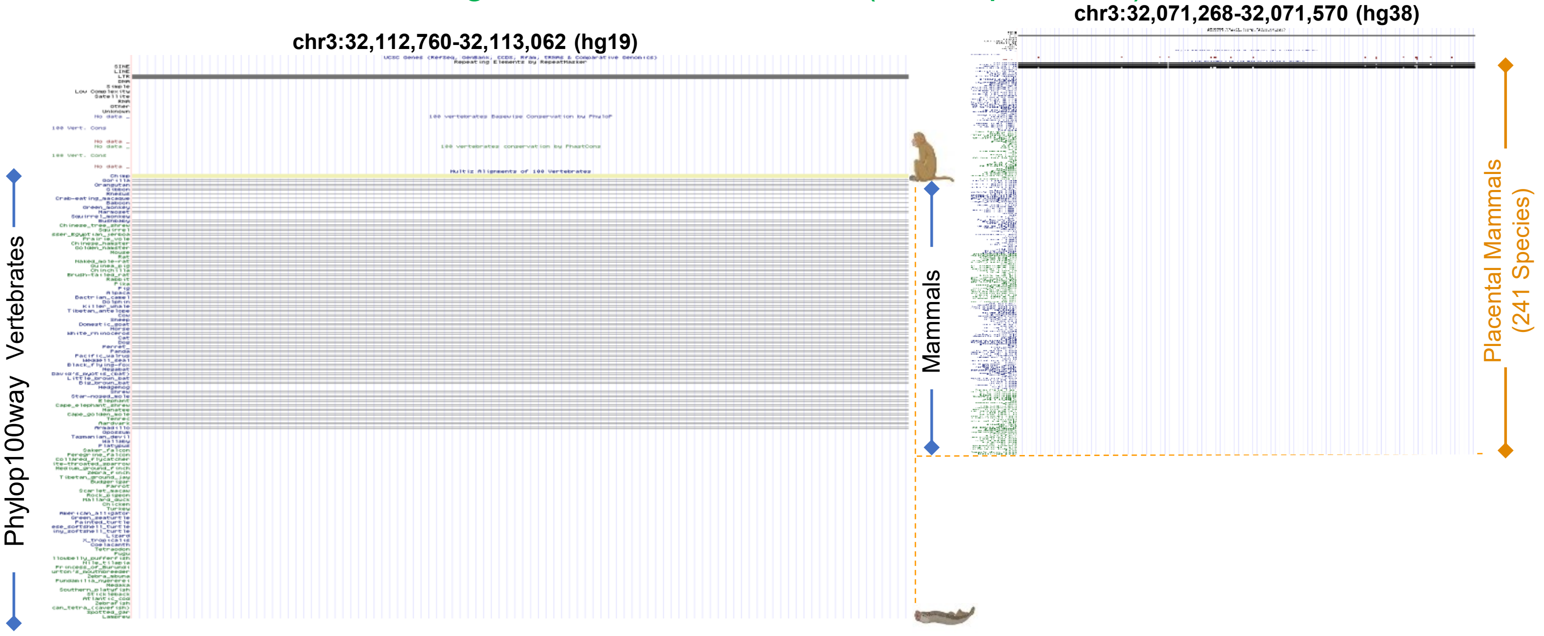

B IRF3-SHAe chr6:109,870,889-109,871,191 (302 bp) Identities:244/283 (86%), Gaps:4/283 (1%) E-value:1e-87

```
IRF3-SHAe 1      ACGGAGTTTCGCTCTGTCGCCCAGGCTGGAGCGCAGTGGCGCGATCTCGACTCACTGCAA  60
Zika virus 11153 ACGGAGTCTTGCTCTGTCGCCCAGGCTGGAGTGCAATGGTGCGATCTCAGCTCACTGCAA  11094

IRF3-SHAe 61     GCTCCGCCTCCCGGGTTCACGCCATTCTCCTGCCTCAGCCTCCCAAGTAGCTGGGACTAC  120
Zika virus 11093 CCTCTGCCTCCTGGGTTCAAGCAATTCTCCTGCCTCAGTCTCCCGAGTAGCTAGGATTAC  11034

IRF3-SHAe 121    AGGCGCGCGCCACCATGCCCGGCTAATT-TTTGTATTTTTAGTAGAGACGGGGTTTCACC  179
Zika virus 11033 A-GCGCCCGCCACCATGCCTGGCTAATTGTTTGTGTTTCTAGTAGAGACGGGGTTTCACC  10975

IRF3-SHAe 180    GTGTCAGCCAGGATGGTCTCGATCTCCTGACCTC--GTGATCCGCCCGTCTCGGCCTCCC  237
Zika virus 10974 ATGTTGGCCAAGCTGGTCTCGAACTCCTGACCTCAGGTGATCCACCTGCCTCAACCTCCC  10915

IRF3-SHAe 238    AAAGTGCTGGGATTACAGGCGTGAGCCACCGCGCCCGGCCTAG  280
Zika virus 10914 AAAGTGCTGGGATTACAGGCATGAGCCGCCACGCCCAGCCTAG  10872
```

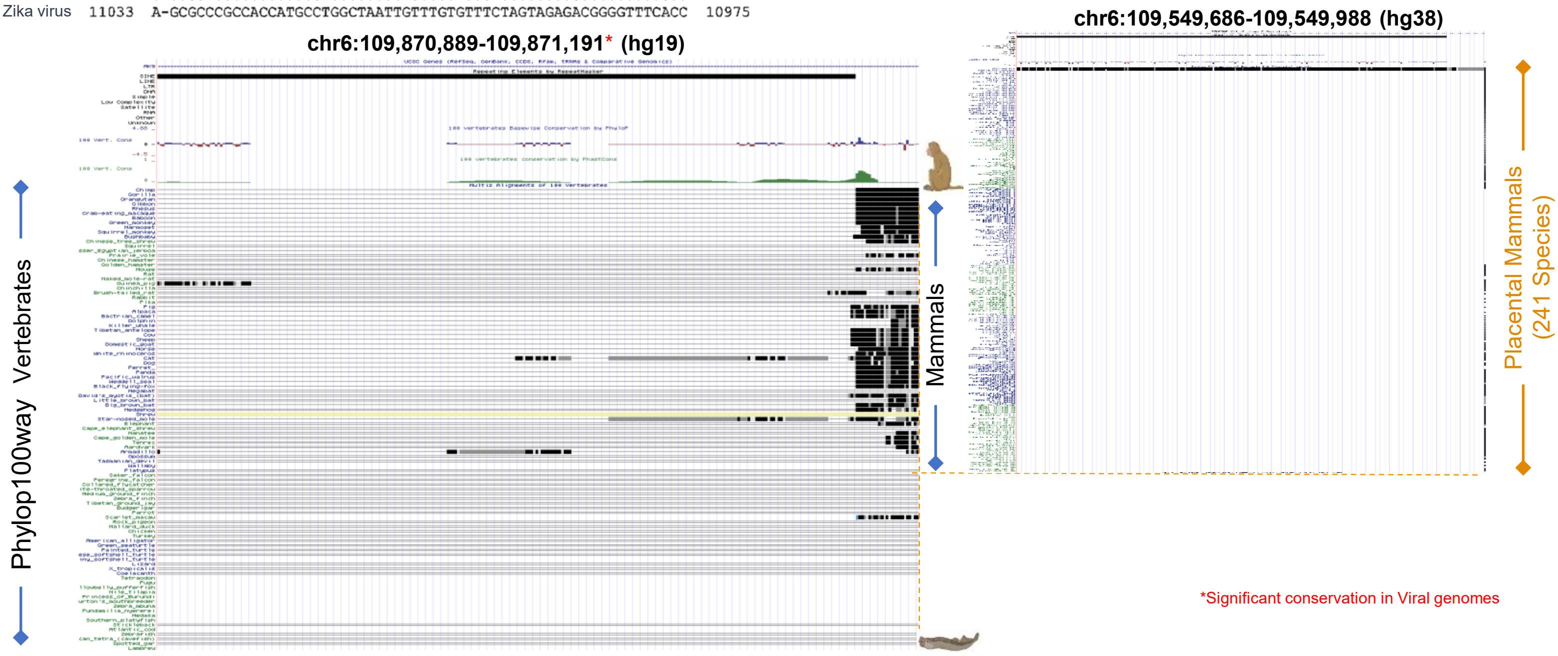

C IRF3-SHAe chr12:131,480,388-131,480,690 (302 bp) Identities:234/250 (94%), Gaps:0/250 (0%) E-value:6e-104

```
IRF3-SHAe                        53   CTGGTTTTCCTAGGCAGAGGACCCTGCGGCCTTCCGCAGTGTTTGTGTCCCTGATTACTT  112
Human endog. retrovirus K113     329  CTGGCTTTCCTAGGCAGAGGTCCCTGCGGCCTTCCGCAGTTTTTGTGTCCCTGGGTACTT  270

IRF3-SHAe                        113  GAGATTAGGGAGTGGTGATGACTCTTAACGAGCATGCTGCCTTCAAGCATCTGTTTAACA  172
Human endog. retrovirus K113     269  GAGATTAGGGAGTGGTGATGACTCTTAAGGAGCATGCTGCCTTCAAGCATCTGTTTAACA  210

IRF3-SHAe                        173  AAGCACATCTTGCACCGCCCTTAATCCATTTAACCCTGAGTGGACACAGCACATGTTTCA  232
Human endog. retrovirus K113     209  AAGCACATCCTGCACCGCCCTTAATCCATTCAACTCTGAGTTGACACAGCACATGTTTCA  150

IRF3-SHAe                        233  GAGAGCACAGGTTTGGGGGTAAGGTCATAGATCAACAGGATCCCAAGGTAGAAGAATTTT  292
Human endog. retrovirus K113     149  GAGAGCACGGGGTTGGGGGTAAGGTCATAGATTAACAGAATCTCAAGGCAGAAGAATTTT  90

IRF3-SHAe                        293  TCTTAGTACA  302
Human endog. retrovirus K113     89   TCTTAGTACA  80
```

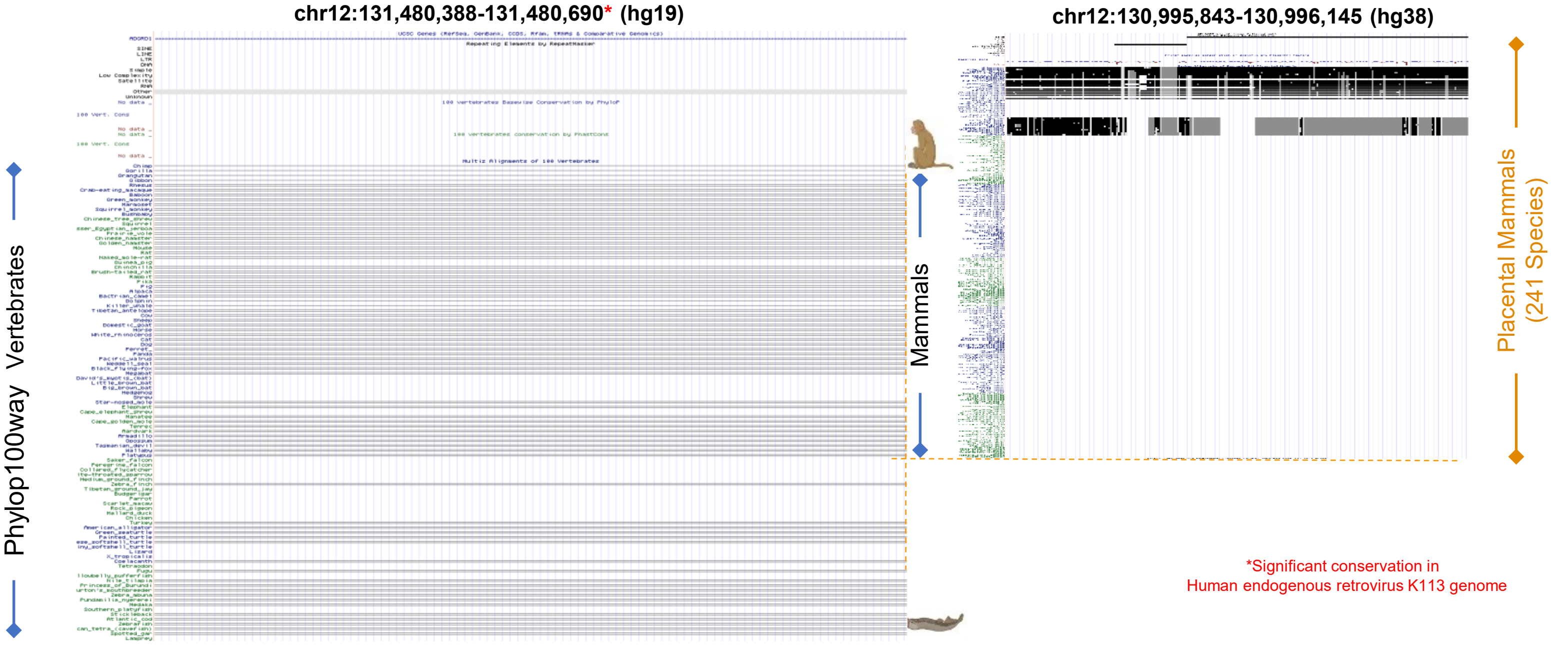

Supplementary Figure S27

A

Ly6e mouse locus chr15:74,763,723-74,764,780 (1057 bp)
Identities:440/459 (96%), Gaps:1/459 (0%) E-value:0

```
Ly6e locus mNIH/3T3  498   ATGCAAACAGCAAAAGGCTTTATTGGGAACACGGGTACCCGGGCGACTCAGTCTATCGGA  557
PreXMRV-1            8197  ATGCAAACAGCAAAAGGCTTTATTGGGAACACGGGTACCCGGGCGACTCAGTCTATCGGA  8138

Ly6e locus mNIH/3T3  558   TGACTGGCGCGCCGGGTGTGGAGTTTTTATCC-TTTTTATAGGGCTAGGGCTGGGGGGCA  616
PreXMRV-1            8137  TGACTGGCGCGCCGAGTGTGGAGTTCTTACCCCTTTTTATAGGGCTAGGACTGGGGAGCA  8078

Ly6e locus mNIH/3T3  617   AAAAGCGCGGGTACAGAAGCGAGAAGCGAGCTGATTGGTTAGTTTAAATAAGGCTTGGGG  676
PreXMRV-1            8077  AAAAGCGCGGGTACAGAAGCGAGAAGCGAGCTGATTGGTTAGTTTAAATAAGGCTTGGGG  8018

Ly6e locus mNIH/3T3  677   TATTTCCCGGTCATTTGGGGAACCTGAAACTGAGGTGGGACTTTCCAGAAACTGTTGCTG  736
PreXMRV-1            8017  TATTTCCCGGTCTTTTGGGGAACTTGAAACTGAGGTGGGACTTTCCAGAAACTGTTGTTA  7958

Ly6e locus mNIH/3T3  737   GTTTCGCTTTATCTGAGTACCATCTGTTCTTGGCCCTGAGCCGGGGCCCAGGTGCTTGAC  796
PreXMRV-1            7957  GTTTCGCTTTATCTGAGGACCATCTGTTCTTGGCCCTGAGCCGGGGCCCAGGTGCCTGAC  7898

Ly6e locus mNIH/3T3  797   CACAGATATCCTGTTTGGCCCCTGTCCCGGTATTATTCAGCCTTATTCTTTAACTAAACT  856
PreXMRV-1            7897  TACAGATATCCTGTTTGGCCCCTGTCCCAGTGTTATTCAGCCTTATTCTTTAATTAAACT  7838

Ly6e locus mNIH/3T3  857   TCCTTGTAACTTTTGAGAACTCAGCTCTGGTACTTTTTCATGCCTTGCAAAATGGCGTTA  916
PreXMRV-1            7837  TCCTTGTAACTTTTGAGAACTCAGCTCTGGTACTTTTCCATGCCTTGCAAAATGGCGTTA  7778

Ly6e locus mNIH/3T3  917   CTGCAGCTAGCTTGCTAAGCCTTATGGTGGGGTCTTTCA  955
PreXMRV-1            7777  CTGTAGCTAGCGTGCTAAGCCTTATGGTGGGGTCTTTCA  7739
```

B

Human *CHEK2* 3rd exon

IRF3/p65 SHAe chr22:29,126,259-29,126,733

Identities:139/171 (81%), Gaps:0/171 (0%)
E-value:4e-39

```
IRF3/p65 SHAe           304    AGGCCAGCCACGGTGGCTCATGCCTGTAATCCCAGCACTTTGGGAGGCCGAGGCGGGTGG  363
Baboon cytomegalovirus  65541  AGGCTGGGCATGGTGGCTCACACCTATAATCCCAGTTCTTCAGGAGGCCGAGGCGGGCGT  65600

IRF3/p65 SHAe           364    ATCACCCGAGGGCAGGAGTTCGAGACCAGCCTGGCCAACATGGTGAAACCCCATCTCTAC  423
Baboon cytomegalovirus  65601  ATCTTTTGAGCTCAGGAGTTCGACGCCAGCCTGGCCAACATGGTGAAATCCCATCTCCAC  65660

IRF3/p65 SHAe           424    TAAAAATACAAAAATTAGCCAGGCATGGTGGCATAAGCATGTAATCCCAGC  474
Baboon cytomegalovirus  65661  AAAAAATACAAAAATTAGCCAGGTGTGGTGGCACGCCCTTGTAGTCCCAGC  65711
```

Identities:155/170 (91%), Gaps:0/170 (0%)
E-value:2e-61

```
IRF3/p65 SHAe      305   GGCCAGCCACGGTGGCTCATGCCTGTAATCCCAGCACTTTGGGAGGCCGAGGCGGGTGGA  364
BeAn 58058 virus   8616  GGCCAGGCGCGGTGGCTCATGCCTGTAATCCCAGCACTTTGGGAGGCCGAGGCAGGTGGA  8557

IRF3/p65 SHAe      365   TCACCCGAGGGCAGGAGTTCGAGACCAGCCTGGCCAACATGGTGAAACCCCATCTCTACT  424
BeAn 58058 virus   8556  TCACATGAGGTCAGGAGTTCGAGACCAGCCTGGCCAACATGGTGAAACCCTCTCTCTACT  8497

IRF3/p65 SHAe      425   AAAAATACAAAAATTAGCCAGGCATGGTGGCATAAGCATGTAATCCCAGC  474
BeAn 58058 virus   8496  AAAAATACAAAAATTAGCTTGGCGTGGTGGCTTTTGCCTGTAATCCCAGC  8447
```

SHAe-encoded
&
G3 classified primate-specific exons

IFIT3	*IFITM3*	*APOL2*	*ACVR1*
PRKAB1	*RAN*	*ACSF3*	*ZNF695*

IRF3- targeted mouse DNA-sequences
Conserved in Viral genomes
Integrated within H3K27ac modified loci

chr15:74,745,778-74,800,893 *Ly6e*

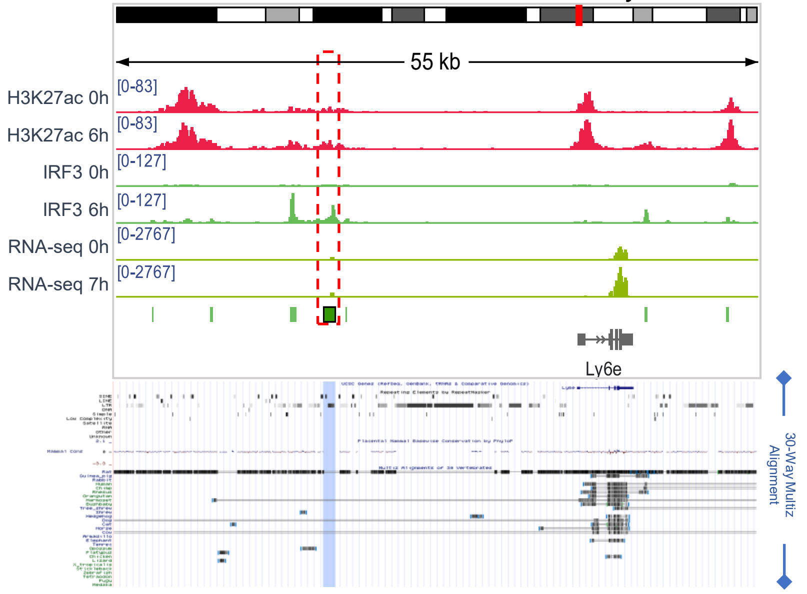

chr15:79,699,994-79,748,109 *Apobec3*

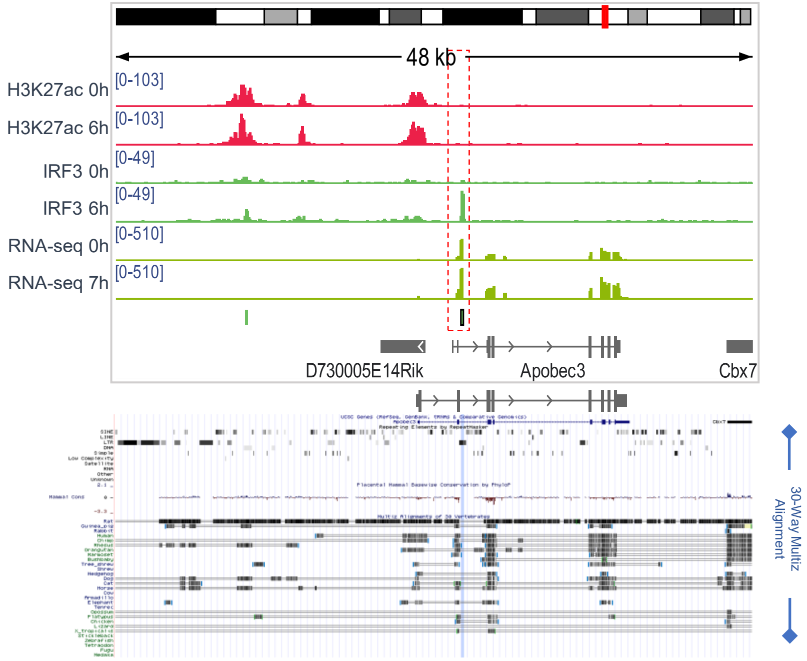

mouse DNA-sequences
Conserved in Viral genomes
w/o IRF3-targeting and H3K27ac modification

chr15:39,290,460-39,345,575 *Rims2*

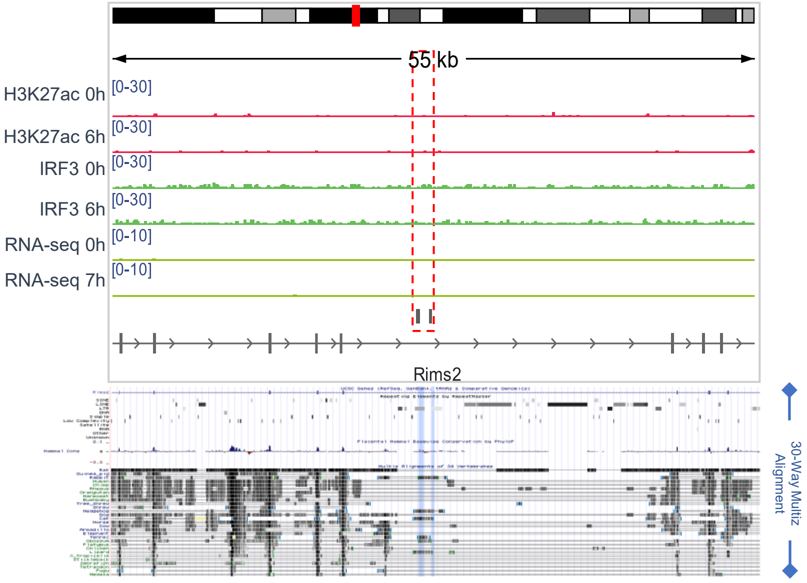

Supplementary Figure S28

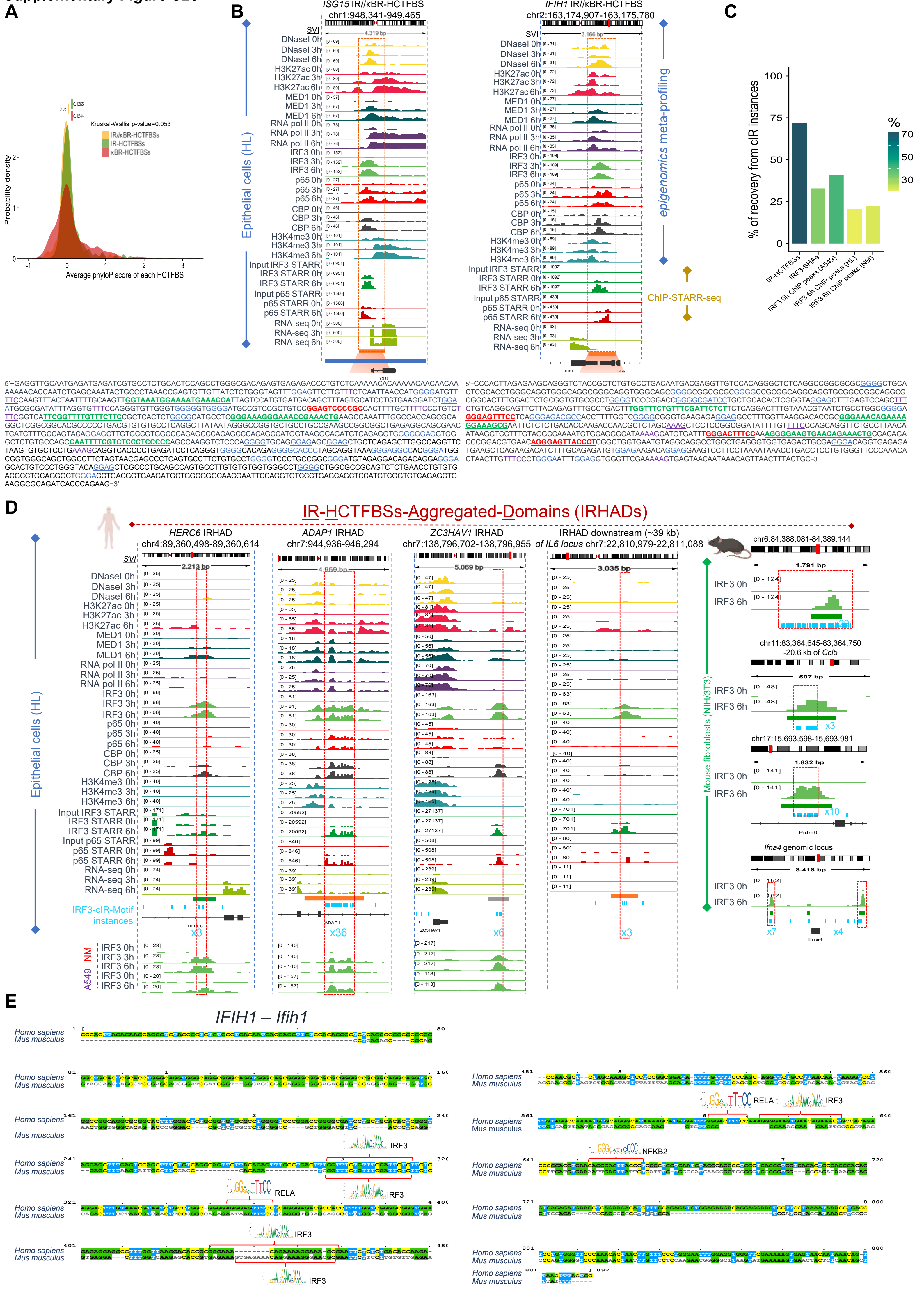

Supplementary Figure S29

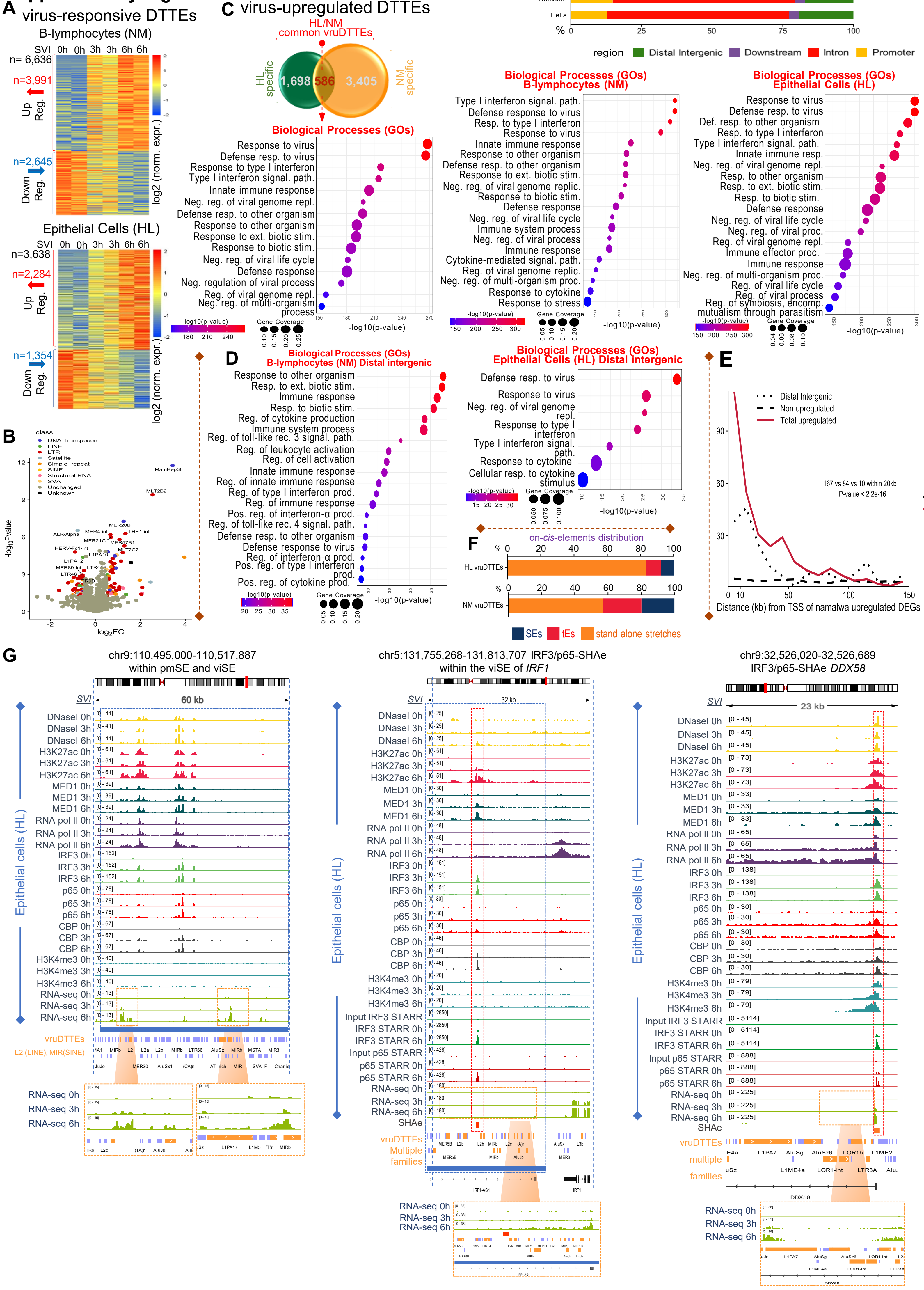

Supplementary Figure S30

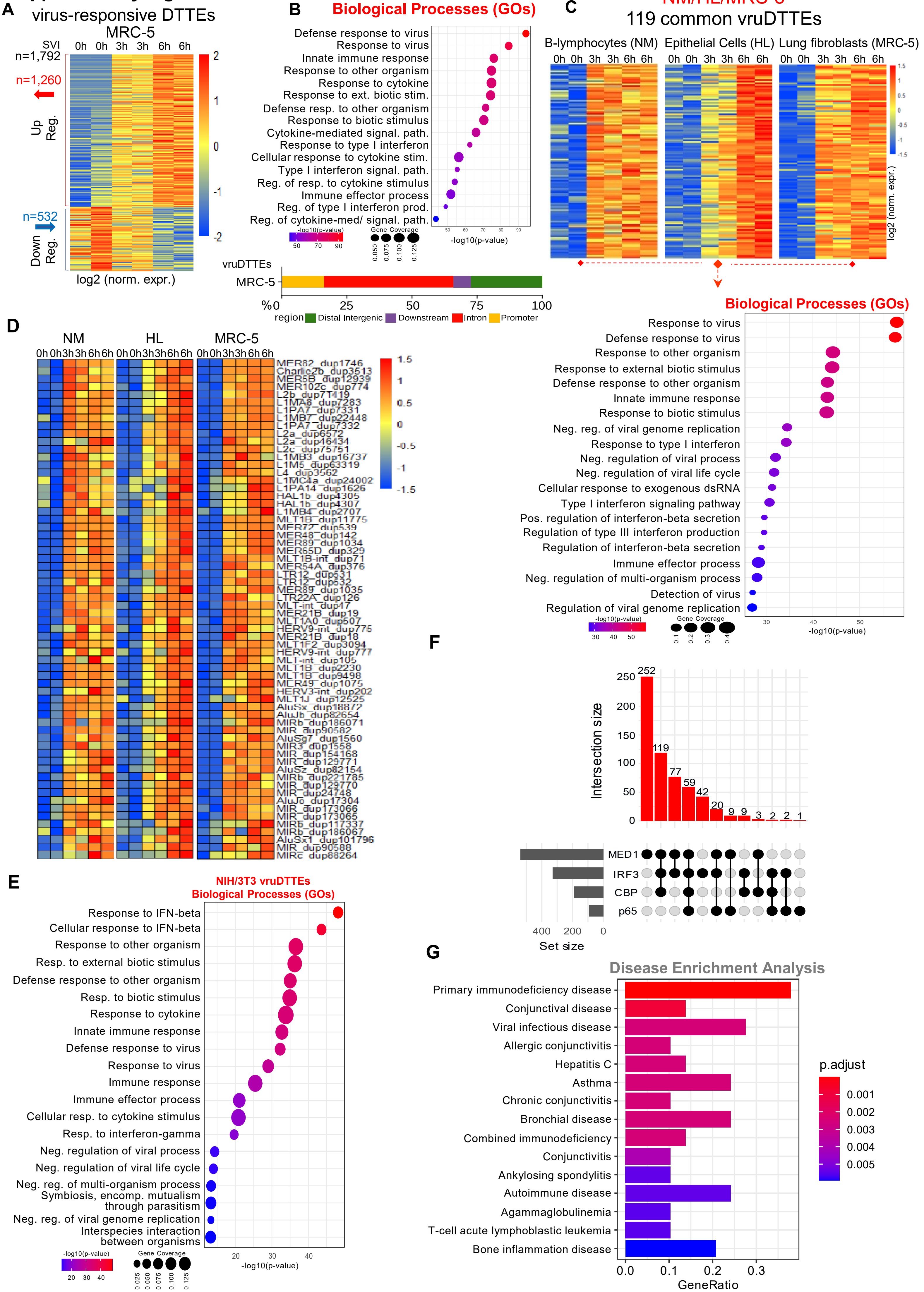

Supplementary Figure S31

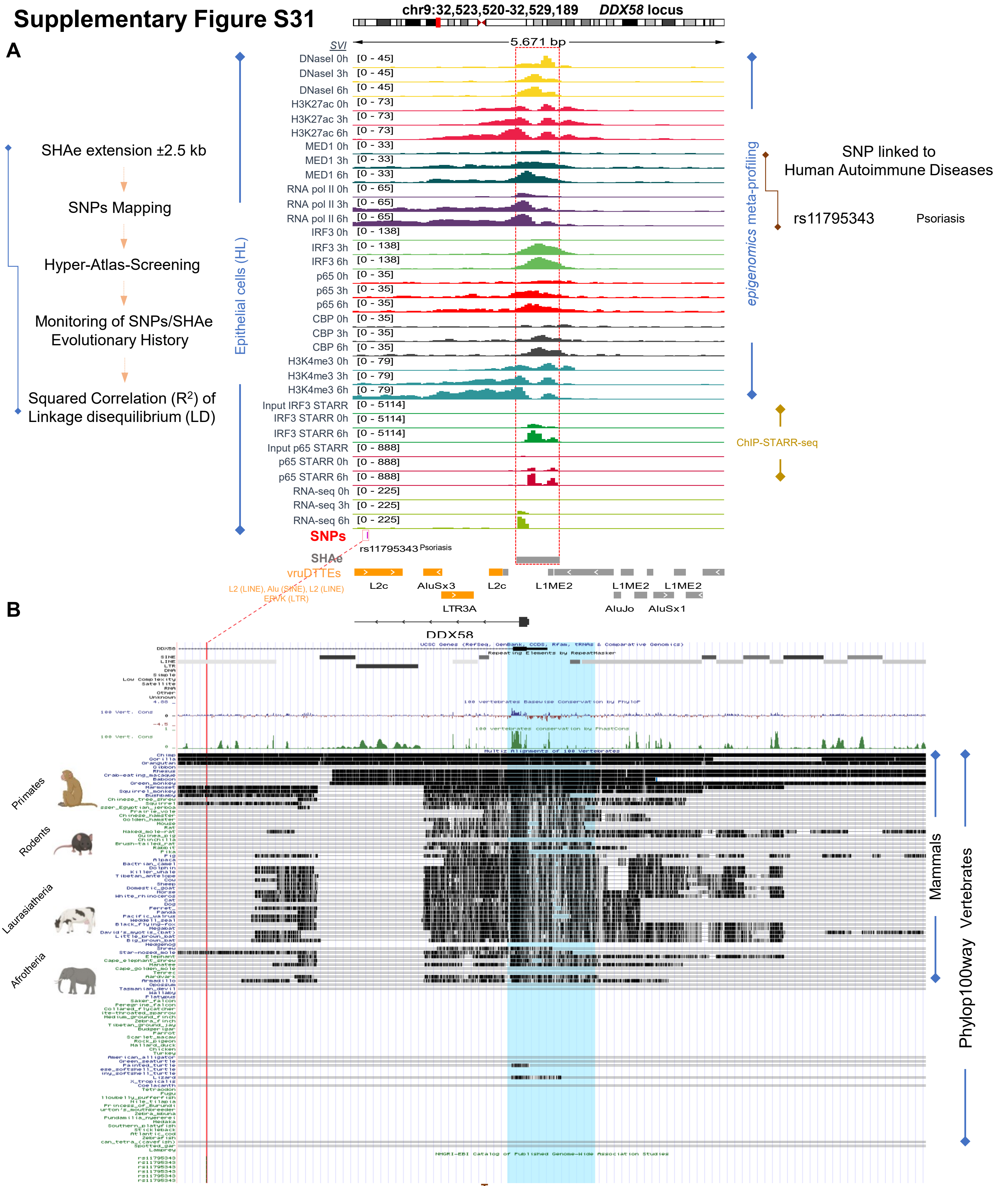

5'–…..ATCCTTGCTTCCAGTTTTTCCACTCTGCTGTCCTGGCATCATTGCATCTGCCATTCTCTCTCTCCCTGTCTTGTACCTATTCAAGCCATTCCTCAAACTGAAGTCAAAGTCGTTTTTCTACAATGCAAATTCTATTTTGGTCCCCTCTCCTTATCACATTTCAGTGGATAAAGT
CCAAAACTGTTCATATCCCACTTCTGCTTGCCTGTCAAGTTTCATCTCTCTGAACTCCTGCCTCTCTCTCCACAATGCCACCATACTAATATTTTCTGGATACCTGGAACACTCTAGTTTCTCTTTCACCTTGGACCTTTGCACATAAGGAGCCCTCTAACTGCAATTTATTGTCTCC
TTCCAGCTAATTTTTCCTCTTTCTTTAGACCTCAGTTCAAATGCCAAATATCTGGGAAGCTTTGGCTGTGCCCTTTGGACTCTAGTAGACATCCCAGCTTTTTCCATCAAAGCACTGCTGCACTTTATTATTTACTATTT…..CCAAAAACTGCAATAACATCAGGAAAGACTGGCAC
TTGGAGACTTTTAGGATTCCCAGGCCCCCTCTCATCGACTTTTTTTGTTTTTCGGTTTTTTTTTTTTTTTTTTTTTTTTTTTTTGGAGACAAGAGTCTCACGATGTCACCCAGGCTGAGTGCAGTGGTGCGATCTCACCATAACCTCTGCCTCCCTGGTTCAAGCGATTTTCATTCCT
CAGCCTCCTGAGTAGCTGGGACTACAGGAGTGCACCACTACGCCTGGCTAATTTTGTATTTTTCGGTAGAGACGGGGTTTCACCATATTGGCCAAG…..AGATCTTACCACAAACCTGGGGAGTCTGGCAGGCTCTCTGCATTGATTAAAAGAAGGGAACGAAGCAAGAGAAA
AACAAGTCCTCAATTGTTTTCCGCCGAGAAGGCGCCGCTGGAGACACTCACCCTCCCTAAACCAGGGGGCCATGTAGCTCAGGATGTAGGTAGGGTCCAGGGTCTTCCGGATATAATCCTGGAAGGCTTGCAGGCTGCGTCGCTGCTCGGTGGTCATGCCGGCCTCTGCTT
GCAGCTAGCTACGTTCCCCGCAGGCTGTGCCTCACTAGCTTTAAAGCCGGGTAGGAGGAGGGCGTGGCGAGCGGGGAAAGCAGGGATTTTCCGAGGAAATGAGGCCGGACGCGAGGCGGAGCTGCATAGGAACTTTAATAACTAAAGGGAAAATCGAAAGTGCAACTAG
CGGCGGGCGGGGTTCGCGGAGGCTGTTTTGCCTCGGGCTAGTTTCGTTTCCCTCCGCACCGAGGAAGCCCGGGAACGGAGAGGACCCCTAGGAATTGCTTTTATTTTTATGATCTTATATTTTGTTTTGCTTTTATAGCGCTAACGTTTAGACACAGTAAAATGCACACAATCT
GAGTGTGCAATTCGATGAGTTTTGTAAAGTGTACACCTGTGTACCCAGTTCTCCCACGTCGGCTGGGTGCCAGGGTAAGTCTATGTTTAGCTTTATAGGAAACCGCCACCTGTTGTCCAAAATGACTGATTACATCCAGTTTACATTCCCACCCGCAGTAGGAGAGCTCCAGTA
GATAGACACATTGGTCAGCCTTTCTTTTTTTTTTTTAATTTTAACTTCCATCTTGGTCGGTGTGAAATGGTATATTATTGTGGTTTTAATTTTAATTTCCCATATAATTAATGGTGGGCACATTTGCATGTGCTTACTGGGTCATTCATATATCTTTTGTGAAGCATCTGCTCCAATCTTTTG
CCTGACTTTGGAGTTTTTTGGTTTATGGATTTACAGGAGTTATTTATTCAGGATACAAAATTGTTAAATATATGTATTCGAAATATCTTCTTCCAGTCTGTGGCTTACCATTTCATTTTCTTAAAGGTGTCTTTTGAGGAGCAGACACTTATATTGATGCAATTTTCAGGTTTTTCTTCTGT
TTGTTGTTTTTTGTGTCCTCTTTAATAAATCTTTGCCTACCCCAAGGTCATGAAAATATTGTTTCCTTTTAGATTTATATTTTTAGCCTTTACTTCTGGGTCTATTACTATCTCAAAATAATTTTGTTGTGGCGTGAGATGGGGGGTCCAGGTTCACTTTTTCCATATTTCTAATTGTTTCA
GCACCGTTTGTTGAAAACTTTTCCTTTTGGCATTGGATAGCCTTGGGACCTTTGTAAAAAAACCAAGTCCATTGAATGTATATGTGTCTATTTCTGACTCTTGTTATATTCTACTTCCTACTACTTTGACAATACCATTGTCTCTAGGTTACTTTAGATTTCAACTAAGTCTCAAAATCAA
GTACTACTAAGTTCTCCAAATTTGTTTTTCACTGTGAAGATATTAGCTATTTGAGATTCTTTGCATTCCTTTTTTTTTTTTTTTTTTTAATTTCTTTTTTT…..TAGGTCTATAATTTCTCTCAGCAGTATTTTAGTTTTGTTTCATTTTATTTTACAGACGGAGTCTCACTATGTTGCCCAAGCTG
GAGTGCAGTGGCTATTCACAGGCACAATTATAGTGTGCTACAGCCTTGAACTCTTGGGCTCAAGGGATCCACCTGGACTCAAGCAATCCTCCTACCTCAGCCATCCAGAGTAGCTGGGACTACAGGCACGGGCCATCACACCTGGCTATTCTGAACTATTTTATGTTATTTGAT
GCTACTGTCAATGGAATTGTTTTTAAGATTTCATTTTCAAATTGTCTGTTGCTACGGTATAGAAATACAAATGATTGGCTGGGTGTGGTGGCTCACACCTGTAATCCCAGCACTTTGGGAGGCTGAGGTGGGTGGATCACCTGAGGTCAGGAGTTCGAGACCAGCCTGGCCAAC
ATGGTGAAACCTCATCTCTACTAAAAATACAAAAAATAGCCGGGCGTGGTGGTGGGCGCCTGTAATCCCATCTACTCGGGAGGCTGAGGCAGGAGAATCGCTTGAACCCAGGAAGCGGAGGGTGTGGTGAGCCGAGATTGCACCACTGCACTCCAGCCTGGGCGACAGAG
TGAGACTCCATCTCACACACACACACAAAAAAAGAAATACAAGTGATTTTTGTTTTTTGACCTTAAAACATGCAACATTGCTAACTTCACTTATTTGTTCTAGTAATTGTTCTGTAAATTCCTCTAAGTTTTCTAGATAAATAATGATGTCATTTGTGAATACATACTTTAACTTCTTCCTT
TCCAATCTTTATGC…..AAGTGGTGACAGTTGACACCATTACTTTATTGCTAATTTCAGTGGGGAAATCATCAATGTAATAATAATCACTTTAAATAATATGGTAGCTGTAAATTTTTCTT…..–3'

Supplementary Figure S32

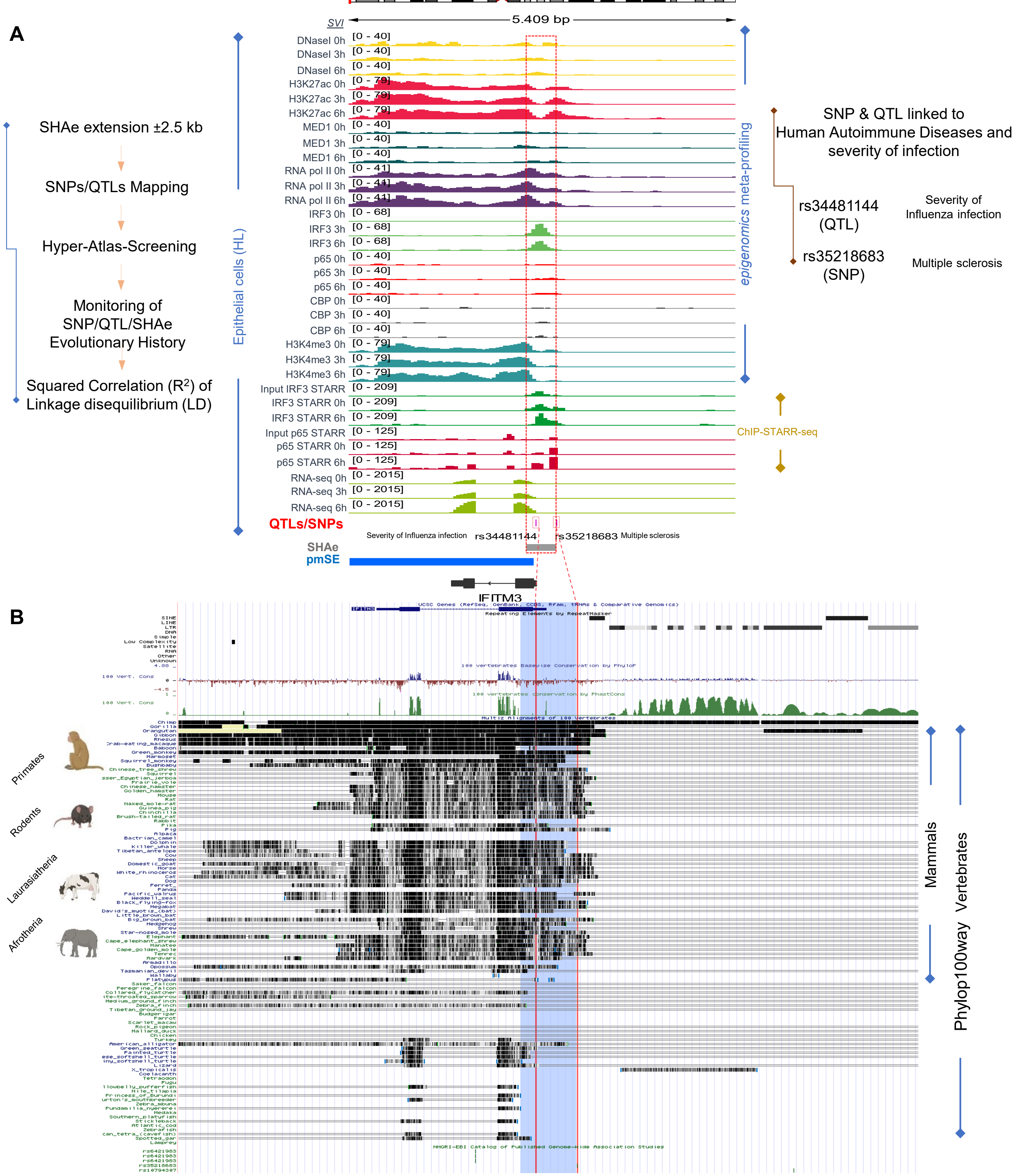

5'-.....GGTCACAGGACCGGGCTCCCAGGGTGACTCCCTGGCCACCAAGTCCTCCAGCAGGTCACTCCTGGATTCCGGCTGGACCCCCTGCCCAGGGAGGGGCCCAGGAGACGCGGAAATCGGGTCCCCCGCGGGTGAAGGGGAGGTGGGAGCCCCAGAGGAGGGCGGG
AAGGCTCGGCCGGGGACGTGCCCGAGGGGGAGGAGAGGGGCTCCCGTCCGGGTCCGGCGCTGCGGGTCTCAGGGTCCGCGTCCCGCCCACCCCGCGGGGGTCTGGGCTGCGGGCGGAGCCTCCTCCGGGACCTGCACCTGCGCCGGCCGCCGCCAGGGGCGCCGC
TGAGCACCCGCCTCCGCCGCTTCCCCCTCCCCGCCGGGCGGGGCCGCGCGCCTCCCACCCGGGACCCACTTCGGAGTTCGCCCCCTTCGGGACTCGCCTCCGGGAGACCCCCGAGGGGGAGAGGGCGCTTCCTCCGCGTCCATCGCCCTTGCCGCCCCTGGGGTGAG
AGCGCGGCTCCGGGGCTGGGGGCGGGAAAGGGTGGGGAGGGCGAGTAGATCCAGACCTGGAGTCGCGGGTGGACGGCGGGGCGGGGGCGCGCATGGCCACCCCAGCTCCCACCGCGGCCGCCCATGGGGCACCCCA.....CCTTGACCTCAGCACAGGCTGTTCTCCC
CACCATGGGGTGAAGTCACCTGTGAGTTCCCTTCTCACTTTCTGGGAGAGCCTGGGTGCCAGTCTGGAAGGTGAAGGTGTAATTTCTTACTGGGCTATAGGGAGAACTGCTCTGGGCTAGTGGATAGCCGGGGACAGACTCTGAGCCAGCCAGCCAGCCTCCTGGAGCCTCC
TCCTAAACCTGCACTGGGCAGATTCCCCAAGCCACACACACACACAGATCACACAGACAAAGCCACTGACGTGGGCACACACGGGACAGAGGTGCACACAAACTCACAGTCACAGGGACACACAAGTCCCCACCCCAGGCAGCATGTGGGCAGGTGGAGCTCCAGGCTCA
GCGGCACCCTCTGAGCATTCCCTGGGGCCATACGCACCTTCACGGAGTAGGCGAATGCTATGAAGCCCAGGCAGCAGGGGTTCATGAAGAGGGTGTTGAACAGGGACCAGACGACATGGTCGGGCACGGAGGTCTCGCTGCGGATGTGGATCACGGTGGACGTCGGGGG
AGCAGGGTTGTGGGGCGCCCCCAGCACAGCCACCTCGTGCTCCTCCTTGAGCATCTCATAGTTGGGGGGCTGGCCACTGTTGACAGGAGAGAAGAAGGTTTGGACAGTGTGATTCATGGTGTCCAGCGAAGACCAGCGG**C**GGTCGGGTTACTGGGATGGTTCTCAGTGAG
CCCTCCCTTTCCCCAGTAGTTTCGGTTTCTCAACAGTTTCCTTTTCCTGGCATTTGTCAAATCGGGATTTGGCCGGGGCCAGATGAGGGCGGAACAAATTCCTGAGGATCAAATTACTTTAGGAGAGGGAGGAAAGAGGGGCTGAGGGCTAAGAGGGGCTTCCTCTCTAGAGC
CCAAGGGAGCACAAGGCTCATCTTGTTAGTGGTGTGGGGTCCTGGAGACTTCCCCTGTGTTGGGGGTCTCCACACCCCAGGCCAGCAGCTGCAGGACC**C**CACTGTCCCGGTTCAGGGCTCCTGCCAGGCCTGCGCTGACGCCTCCACACTCCAGGCCCCACAACCGTTCC
CCCAAAGCCCTCACCTCTGCCGGGCGCCGCGGCTCACGCCTGTAATCCCAGCACTTTGGGAGGCTGAGGTGGAGGATCATGAGGTCAGGAGATCGAGACCATCCTGGCTGACACGGTGAAACCCCGTCTCCCATAACCCTCACTGTGGTGCCCAGGCAGCTGGATTATC
CCATTACCTTCACTATGGTGCCCAGGCAGCTGGATTATCCCATTACCTTCACTATGGTGCCCAGGCAGCTGGATTATCCCATTACCTTCACTATGGTGCCCAGGCAGCTGGATTATCCCATTACCTTCACTATGGTGCCCAG.....AGTGTGGTGCCCAGGCAGCTGGATTATCCC
ATAACCCTCACTGTGGTGCCCAGGCAGCTGGATTATCCCATTACCTTCACTATGGTGCCCAGGCAGCTGGATTATCCCATAACCCTCACTGTGGTGCCCAGGCAGCCGGATTATCCCATTACCTTCAGTGTGGTGCCCAGGCAGCTGGATTATCCCATAACCCTCACTGTGGT
GCCCAGGCAGCTGGATTATCCCATTACCTTCACTATGGTGCCCAGGCAGCTGGATTATCCCATTACCTTCACTATGGTGCCCAGGCAGCTGGCGTTCAGGCCTGCTCAGGCGTCTCATGACCTTCACTGTACTTCTTAGATAAAACAGAATACTTGAAGTCACTAGTTACAGAG
AACAGGAATCTATAAACTCATTCCAAAAACAAAGGAAAATTTGTTTTTTCTTCTCCCTGTGTTAAGGGAGTGCTGGGAGAGTCTCAGAGCACATTAGATAATATTATCAGGACTTTTCCTGGGTCTCAGCTGTGTCTGCTGCTGCCTCCGGGACGAGTCAGCCTGATACAGGAAAA
CCTATTTCTCTTTCTTTTTAATTTTAATTTTATTTAATTTCCTGCCTCAT .....-3'

RS ID	rs34481144	rs35218683
rs34481144	1	0.612
rs35218683	0.612	1

Supplementary Figure S33

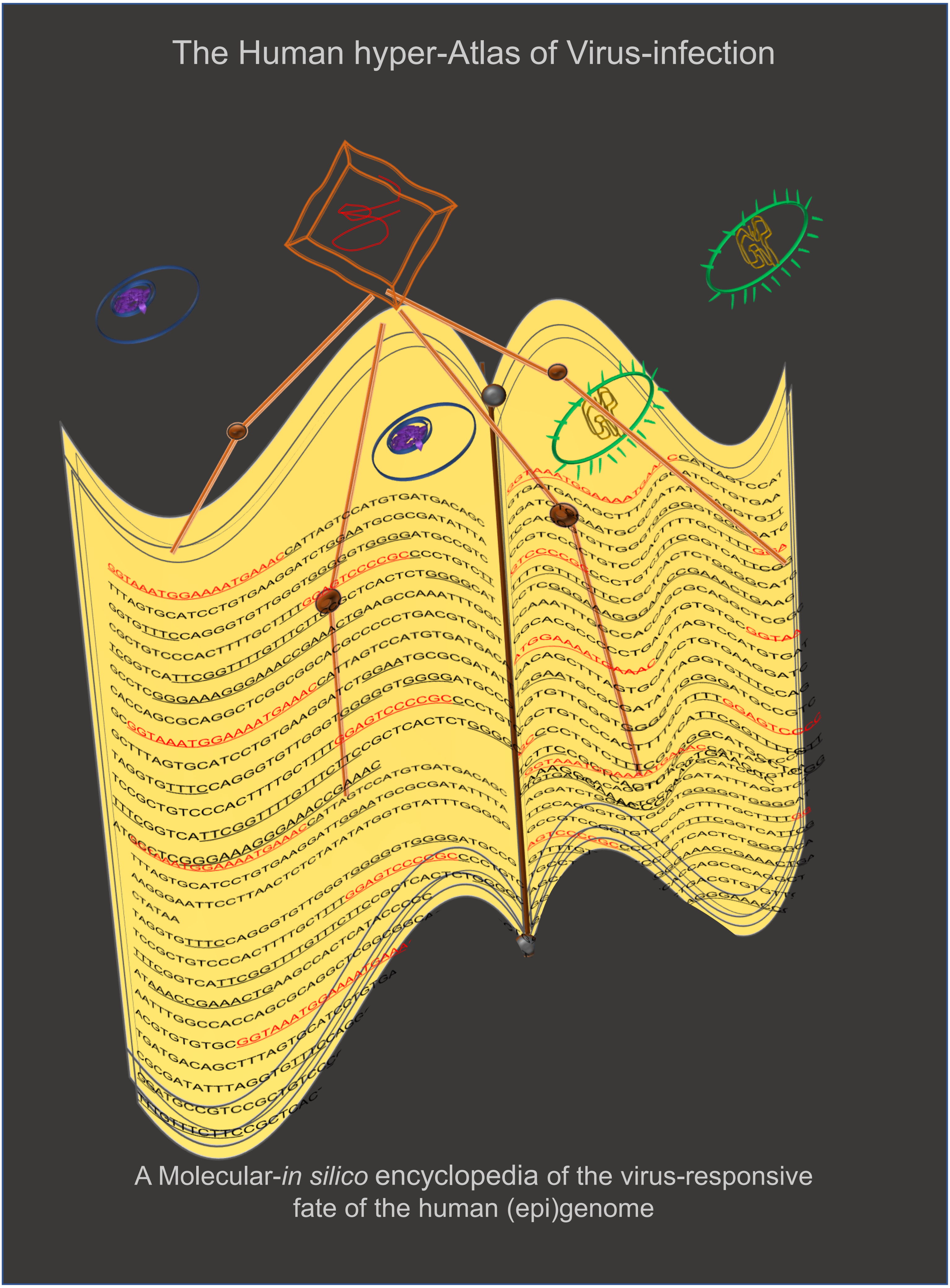

Supplement: gkaf207_Supplemental_Files [file gkaf207_supplemental_files.zip › 26_2_Supplemetary File_Combined.pdf]
